# Cost-efficiency assessments of marine monitoring methods lack rigor—a systematic mapping of literature and an end-user view on optimal cost-efficiency analysis

**DOI:** 10.1007/s10661-021-09159-y

**Published:** 2021-06-09

**Authors:** Heini Hyvärinen, Annaliina Skyttä, Susanna Jernberg, Kristian Meissner, Harri Kuosa, Laura Uusitalo

**Affiliations:** 1 Programme for Environmental Information, Finnish Environment Institute, Jyväskylä, Finland; 2 Department of Biological and Environmental Science, University of Jyväskylä, Jyväskylä, Finland; 3 Finnish Environment Institute, Programme for Environmental Information, Helsinki, Finland; 4 Finnish Environment Institute, Marine Research Center, Helsinki, Finland

**Keywords:** Cost-efficiency analysis, Cost of monitoring, Marine monitoring tool, Method performance, Method standardization

## Abstract

Global deterioration of marine ecosystems, together with increasing pressure to use them, has created a demand for new, more efficient and cost-efficient monitoring tools that enable assessing changes in the status of marine ecosystems. However, demonstrating the cost-efficiency of a monitoring method is not straightforward as there are no generally applicable guidelines. Our study provides a systematic literature mapping of methods and criteria that have been proposed or used since the year 2000 to evaluate the cost-efficiency of marine monitoring methods. We aimed to investigate these methods but discovered that examples of actual cost-efficiency assessments in literature were rare, contradicting the prevalent use of the term “cost-efficiency.” We identified five different ways to compare the cost-efficiency of a marine monitoring method: (1) the cost–benefit ratio, (2) comparative studies based on an experiment, (3) comparative studies based on a literature review, (4) comparisons with other methods based on literature, and (5) subjective comparisons with other methods based on experience or intuition. Because of the observed high frequency of insufficient cost–benefit assessments, we strongly advise that more attention is paid to the coverage of both cost and efficiency parameters when evaluating the actual cost-efficiency of novel methods. Our results emphasize the need to improve the reliability and comparability of cost-efficiency assessments. We provide guidelines for future initiatives to develop a cost-efficiency assessment framework and suggestions for more unified cost-efficiency criteria.

**Supplementary Information:**

The online version contains supplementary material available at 10.1007/s10661-021-09159-y.

## Introduction

Marine ecosystems face multiple challenges, many of which arise from human activities (e.g., Andersen et al., 2015; Crain et al., 2008; Halpern et al., 2008; HELCOM, 2018; Millenium Ecosystem Assessment, 2005). Therefore, society requires a better understanding of the state of marine ecosystems and the impacts of human activities over extensive temporal and spatial scales (Lovett et al., 2007). Along with national and international legislations such as the European directives, this leads to extensive data and information needs from multiple parts of the marine ecosystem, which require high quality and comprehensive, yet cost-efficient monitoring (Míguez et al., 2019).

Cost-efficiency of monitoring has two components: the monetary cost of the monitoring and data handling and the utility (“efficiency”) of the data. The monetary costs associated with obtaining the data are often caused by the use of platforms such as vessels and satellites, expendable costs such as laboratory chemicals, and personnel time required to process the samples. The utility of the data is the benefit that is gained from having this data and the associated increased knowledge. If the data is not useful, it will not be cost-efficient even if it can be obtained very cheaply. Increased cost-efficiency can therefore be obtained either through reduced monitoring costs or improved data utility. It becomes clear from this definition, as well as earlier work (e.g., Koski et al., 2020; Nygård et al., 2016), that conducting a comprehensive assessment of cost-efficiency of marine monitoring is often complicated, as cost information is generally difficult to obtain, and the utility of the data difficult to discern. Emerging monitoring methods which provide the potential to get new types and larger quantities of data, or data with higher temporal and/or spatial resolution compared to current methods, are often presented as cost-efficient. However, it is not always obvious how the claimed cost-efficiency has been assessed.

The aim of this paper is to provide better understanding of the state-of-the-art regarding marine monitoring methods’ cost-efficiency assessments through identifying, classifying, and discussing the arguments supporting claims of cost-efficiency of marine monitoring methods. To this end, we conducted a systematic literature mapping (James et al., 2016) of scientific literature published since the year 2000, comprising 1684 papers. In this paper, we assess how often, and in which ways the claim of cost-efficiency of a marine monitoring method is backed with data, calculations, or arguments, and what those corroborating arguments and assessment methodologies are. Finally, we provide recommendations on how to conduct more comprehensive and transparent cost-efficiency assessments.

## Methods

The systematic mapping method originated in social sciences as a response to the need to make transparent, comprehensive synthesis of the available evidence in cases where systematic reviews were not feasible, e.g., due to the open-framed nature of the research question (Clapton et al., 2009; James et al., 2016). While systematic reviews aim to answer specific, well-defined questions using results from multiple studies (James et al., 2016; Lockwood, 2017), systematic mapping is better suited to open-framed questions such as describing the state of the knowledge for a topic, discovering the actual amount of evidence and the kind of studies that have been carried out (James et al., 2016). For such questions, strict inclusion criteria such as population, interventions or exposure, and defined outcomes of interest, may be irrelevant or impossible to formulate. Systematic mapping offers a way to summarize the existing knowledge in a comprehensible and transparent manner to serve as a basis of decision-making or for further studies.

The systematic mapping protocol includes many of the same elements as a systematic review, such as the searches and reproducible reporting of the search methods and the number of articles found. We adopted the protocol for systematic mapping for environmental sciences suggested and outlined by James et al. (2016) and used CADIMA software (Cadima.info, 2019), an open access tool designed to facilitate and formalize the systematic review/mapping process. The software guides reviewers through the review process step by step. When there are several reviewers reading and selecting the articles based on decided criteria, the advantages of using the software include an inbuilt consistency check between reviewers.

**Step 1: Establishing a review team**

The core review team included marine scientists and ecologists knowledgeable in marine monitoring and the policy frameworks affecting it (the Water Framework Directive and Marine Strategy Framework Directive of the EU). In the initial literature search stage, an information specialist was consulted to maximize the efficiency of the database searches.

**Step 2: Scoping, research question, and inclusion criteria**

Our primary research question was: *What methods have been used to evaluate the cost-efficiency of marine monitoring methods since the year 2000?* As this question aims to describe the current state of the art of the cost-efficiency analysis methods, it clearly called for the use of a systematic mapping, rather than a systematic review (see also James et al., 2016). As the research question regards the occurrence of different cost-efficiency evaluation methods, it contains the key elements of a population outcome, PO question type, where (i) population = scientific articles that include a cost-efficiency assessment and (ii) outcome = a cost-efficiency assessment method.

The main focus of this research was to map the cost-efficiency estimation methods of marine monitoring; however, also other search terms such as “coastal” and “freshwater monitoring” were included, since they all share common features.

Based on the research question, the following search was defined:

TITLE-ABS-KEY (marine OR sea OR coast* OR estuar* OR freshwater OR lake) AND TITLE-ABS-KEY (monitoring OR survey) AND TITLE-ABS-KEY (“cost efficien*” OR cost-efficien* OR “cost effectiv*” OR cost-effectiv*) AND PUBYEAR > 1999

Additionally, the obtained material was reviewed to answer the question:

How is cost-efficiency of monitoring defined in these studies?

Two inclusion criteria were set for the title and abstract screening: (1) the article deals with aquatic environment monitoring and (2) the article assesses the cost-efficiency of the monitoring methods.

**Step 3: Searching for and screening the evidence**

The databases Scopus and Web of Science were queried on March 21, 2019 using the aforementioned search criteria. Our search yielded a total of 1684 articles (after duplicate removal) (see Annex 1) which were uploaded to CADIMA as RIS files. The inclusion criteria set in step 2 were used in the title and abstract screening; an article was included for the full text screening if the abstract included a mention of aquatic environment monitoring and the cost-efficiency of the monitoring method. Ten percent of the abstracts were jointly screened by the two reviewers as a confidence check. When inconsistencies between reviewers occurred, they were automatically highlighted by CADIMA allowing the reviewers to resolve the conflicts. When the confidence check gave an acceptable result (kappa value > 0.4; strength of the agreement: fair), one of the reviewers continued to screen the abstracts independently. A total of 591 articles were selected for full text reading. The full text was available for a subset of 521 papers (subset B), and an article was included if it offered some information on how the cost-efficiency assessment was made. At this review stage, further articles were excluded if they did not deal with aquatic monitoring methods even though the title and abstract had seemingly indicated so. We found 313 articles that actually included relevant knowledge about cost-efficiency assessment methods and hence contributed to the knowledge base (subset C; see Annex 2). To reduce personal bias and avoid screening out relevant studies, the reviewers revisited and discussed the screening criteria whenever their application to the full text seemed ambiguous.

**Step 4: Coding and production of the systematic database**

Information about the articles of subset C that contributed to the knowledge base is catalogued in Table 1, and the full information collected from the articles of subset C will be made available online (Annex 3). The initial screening of these 313 articles recorded bibliographic information, monitoring method(s) assessed, the monitored parameter (e.g., zooplankton), why the method was considered cost-efficient, and how or whether cost-efficiency was assessed. Our second full text screening recorded more detailed descriptions of *costs* (e.g., cost per sample or price of data) and parameters that indicated the *efficiency* of the method (e.g., number of samples needed). In the initial screening, we mostly categorized comparative methods for cost-efficiency assessments as “comparisons with other methods”. The second screening also distinguished between whether a comparative cost-efficiency assessment method was either (1) a comparative study based on an experiment or a systematic literature review or (2) a comparison with other methods based on literature or experience/intuition. Thus, we categorized comparative assessment methods either into comparisons that included a study of two or more methods or comparisons that were not based on a study.

**Table 1:** The articles of the subset C that included a cost-efficiency assessment method for a monitoring method and, thus, contributed to the knowledge base. Full references can be found at the data table (Annex 3)

2000	Kozma Törökné et al
2001	Franklin et al., Iwamoto et al.
2002	Chial and Persoone, Cooke and Schreer, Donaldson et al., Kong et al., Kvernevik et al., Munksgaard et al., Nendza, Yang et al., Lydersen et al., Mumby and Edwards
2003	Buzzelli et al., Jönsson et al., Lu et al., Smith, Thompson et al., Mueller
2004	Au, Dinsdale and Harriott, Dissanayake and Galloway, Jäger et al., Moreira et al., Moreira-Santos et al., Jones and Glegg, Mackinson et al.
2005	Anderson et al., Baldantoni et al., Farré et al., Fuhrman et al., Hodge et al., Lampadariou et al., O’Driscoll and Macaulay, Tercier-Waeber et al., Valta-Hulkkonen et al., Xu et al., Tagliapietra et al.
2006	Bell et al., Fornes et al., Kinzelman et al., Prabhudesai et al., Roelfsema et al., Davidson et al., Diana et al., Yamano et al.
2007	Aguado-Giménez et al., Alquezar and Boyd, Krstić et al., Leujak and Ormond, Mosindy and Duffy, Rotherham et al.
2008	Ding et al., Ding and Rogers, Goreau et al., Jones et al., Rotherham et al., Zappalà et al., Litaker et al., Tercier-Waeber and Taillefert
2009	Abdelzaher et al., Costa et al., Farrell et al., Kong et al., Léopold et al., Risk et al., Rundberget et al., Ryan et al., Song et al., van Overmeeren et al., Williams and Thomas, Brischoux et al., Rickerby, Wight et al.
2010	Ashraf et al., Assilzadeh et al., Bailey et al., Evans and Abdo, Kim et al., Knight-Jones et al., Knutsen et al., Murphy and Jenkins, Ruse, Santos et al., Sheehan et al., Smale, Smith et al., Waddington et al., Xu et al., Chai et al.
2011	Aarnio et al., Andrade and Renaud, Bastian et al., Bresciani et al., Descamp et al., Fairclough et al., Heblinski et al., Klečka and Boukal, Lagarde and Jaffrezic-Renault, Murray et al., Pelletier et al., Rich et al., Ruse, Rönkä et al., Seoane et al., Shin, Smale et al., van Rein et al., Byer et al., Shinohara et al.
2012	Balfour, Camino-Sánchez et al., Fernandes et al., Gera et al., Hitz et al., Hoyer et al., Ingleton and McMinn, Jackson et al., LaCommare et al., Michailova et al., Nagai and Itakura, Polak-Juszczak, Pollard, Porst et al., Qing et al., Schouten and Parisi, Solberg, Van Rein et al., Wong et al., Zhang and Hanner, Canedo-Arguelles et al., Fernandes et al.
2013	Balfour et al., Berman et al., Bourlat et al., Brodin et al., Emelogu et al., Gardner and Struthers, Gerovasileiou et al., Hicks et al., Kanninen et al., Kobryn et al., Martinis et al., Pinna et al., Ramkilowan et al., Teixeira et al., Waseem et al., Bellchambers et al., Deus and Gloaguen
2014	Delparte et al., Fairclough et al., Mallet and Pelletier, Malley and Williams, Martinez-Haro et al., Puhr et al., Ruiz et al., Silva et al., Stringell et al., Turner et al., Unsworth et al., Vianna et al., Xu et al., Clemento et al., Gray et al., Ozsoy-Cicek, Rishworth et al.
2015	Bonino et al., Allan et al., Assoumani et al., Borker et al., Bramburger et al., Castellote et al., Cragg et al., Embling et al., Ewing and Frusher, Johnston et al., Koenig and Stallings, Kopf et al., Mancini et al., Miya et al., Noyer et al., Rajamani and Marsh, Sevilla et al., Southwell and Emmerson, Stern et al., Van Lancker and Baeye, Williams et al., Xu et al., Zeh et al., Codiga, Melo et al., Souza and Barros, Thomsen and Willerslev
2016	Abdullah et al., Aylagas et al., Bennett et al., Bui et al., Cheng et al., Danovaro et al., Evans et al., Hedley et al., Kalaji et al., Keskin et al., Kuzukiran et al., Lanzén et al., Le Reste et al., Lintern et al., Martinez-Haro et al., Mazurkiewicz et al., Minchin et al., Neto et al., Ouyang et al., Porst et al., Romagnan et al., Strindberg et al., Sun and Fine, Turner et al., Watson and Huntington, Ventura et al., Vilmi et al., Wood et al., Yamanaka and Minamoto, Yoon et al., Zhou et al., Booth, Aykanat et al., Boman et al.
2017	Aylesworth et al., Begliomini et al., Beisiegel et al., Bellanger and Levrel, Cahalane et al., Clayton and Dennison, Guzman and Condit, Huang et al., Jiang et al., Karczewski et al., Kotilainen and Kaskela, Koydemir et al., Lanzén et al., Laran et al., Lembke et al., Minamoto et al., Misra and Balaji, Mortensen et al., Moxley et al., Pergent et al., Pirotta et al., Qi et al., Ransome et al., Sánchez-Gendriz and Padovese, Sykora-Bodie et al., Terán-Baamonde et al., Trasviña-Moreno et al., Watson et al., Yin et al., Amin et al., Bosch et al., Collins et al., Goetze et al.
2018	Atkinson et al., Aubert et al., Bartholomew et al., Bevilacqua et al., Bian et al., Boldt et al., Braulik et al., Carpio et al., Chambault et al., Colefax et al., Currie et al., Davies et al., Flynn et al., Gallo et al., Gordoa et al., Göröcs et al., Harper et al., Hering et al., Karatayev et al., Latini and Petrere Júnior, Leonardo et al., Lim et al., Melnik et al., Perini et al., Piermattei et al., Pitois et al., Schaeffer et al., Schmidt et al., Stahr and Knudsen, Toma et al., Turemis et al., Varkitzi et al., Warren et al., Warren-Myers et al., Vasimalai et al., Ventura et al., Wittwer et al., Yamanaka et al., Florisson et al., Jouvet et al., Popescu and Iordan, Stoeck et al.
2019	Dong et al., Gill et al., Harper et al., Hulley et al., Jeunen et al., Li et al., Molognoni et al., Siegenthaler et al., Slimani et al., Claire et al., Mahmood et al.

**Step 5: Describing the findings**

We analyzed and described the methods of cost-efficiency assessment found in the articles in subset C.

## Results

### The number of studies that include a cost-efficiency assessment of a monitoring method

**Fig. 1:**
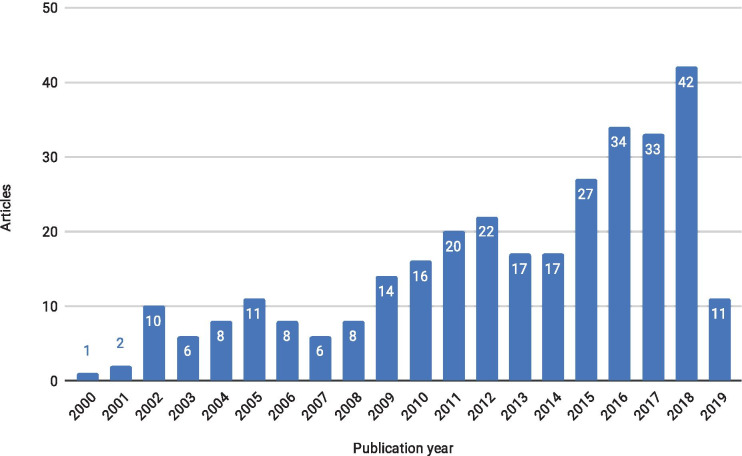
The number of articles including a cost-efficiency assessment method per publication year (2019 until March 21, 2019)

A total of 313 out of 1684 papers from the literature search were found to include some assessment of cost-efficiency of a monitoring method. We observed a clear, steadily increasing temporal trend with 21 times more articles published in 2018 than in 2001 (Fig. 1).

The increase in the number of cost-efficiency analyses in marine monitoring may partially reflect the impact of the adoption of the European legislature, which requires more rigorous monitoring, i.e., the Water Framework Directive (WFD; EC, 2019) and the Marine Strategy Framework Directive (MSFD; EC, 2008). Roughly a sixth of the studies (subset C) referenced either the WFD, MSFD, or HELCOM (Baltic Marine Environment Protection Commission) common objectives, i.e., to assess the current status of aquatic ecosystems, compare it to the good ecological state, and if needed, to restore the good ecological status. Many of these articles emphasize that to achieve these objectives, novel, cost-efficient monitoring methods are required.

### Justifications for cost-efficiency

**Fig. 2:**
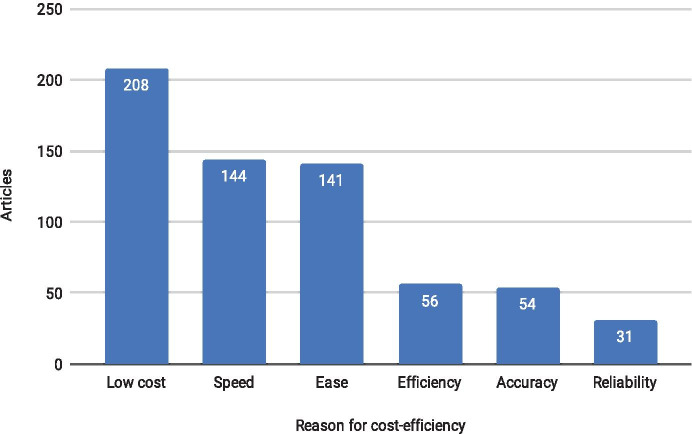
The most frequently given reasons for the cost-efficiency of monitoring methods in literature. There is overlap between different categories as a method can be, e.g., both rapid and low-cost

Monitoring methods are presented as cost-efficient based either on their cost, speed, ease, efficiency, accuracy, or reliability (Fig. 2).

Assessment of costs

Most of the reviewed studies (66%) described the proposed monitoring method as less costly than current methods. However, most of these articles (67%) did not offer any information on the financial costs of the method. Only a fifth of the included articles provided any detailed cost information for the presented method or its relative cost compared to another method. Even fewer articles provided cost comparisons that reported monetary cost for more than one method. Thus, it can be concluded that the authors of such comparisons either (1) were aware of the financial expenses related to the compared methods and chose not to include them in the study or (2) based the cost-efficiency assessment on an intuition about the assumed costs of other methods.

In the studies that argued that the method is cost-efficient based on its cost compared to other methods, the focus was often on other issues, and the cost-efficiency was mentioned only in passing. Costs were sometimes equated to labor costs, e.g., the time spent on the collection and processing of data (e.g., Flynn et al., 2019) or to logistical costs calculated as person days (e.g., Stringel et al., 2014).

Some studies did not mention the financial cost of the method at all, but the cost-efficiency assessment was based only on the superior attributes of the novel method, such as faster operation or better detection capabilities, implying either reduced costs of monitoring or increased data utility. Time-efficiency, i.e., reduced time required to collect and analyze the data, is also essentially a cost-related argument, although if the data comes in a different form from the traditional monitoring data, it may also be more efficient information-wise.

Assessment of efficiency

Many of the studies dubbed a method efficient but entirely failed to define efficiency. Efficiency can be related to the necessary number of samples or the number of indicators that can be assessed utilizing the method as well as to the usefulness of data provided by the method. Even inexpensive and rapid monitoring methods are not necessarily cost-efficient if the obtained data cannot be used in a meaningful way. However, a few studies considered the scientific gain (e.g., Descamp et al., 2011) or added scientific value (e.g., Brodin et al., 2013; Mackinson et al., 2004) in itself a benefit of the proposed method.

The effectiveness of proposed monitoring methods was justified either by listing the advantages of the method over other methods or without comparisons. Qualities that are presented as an advantage compared to other methods include better, more sensitive detection capability over other methods, the ability to monitor new parameters, and the removal of a disadvantage evident with other methods (e.g., Mackinson et al., 2004).

### Ways to compare cost-efficiency with other monitoring methods

We identified five distinct methods to compare the cost-efficiency of monitoring methods: (1) cost–benefit ratio, (2) comparative studies based on an experiment, (3) comparative studies based on a literature review, (4) comparisons on literature, and (5) subjective comparisons based on experience or intuition. Often articles used more than one assessment method, for example, a cost–benefit ratio was generally coupled with a comparative study based on an experiment.

Very few of the studied articles provided a precise definition for the cost-efficiency of a monitoring method. Souza and Barros (2015) defined cost-efficiency of a method as demanding the least effort and expense to obtain reliable results. Rotherham et al. (2007) defined cost-efficiency as optimal levels of spatial and temporal replication given restrictions of time, money, or both. The most extensive definition of cost-efficiency given by Hering et al. (2018) includes factors such as the monetary cost of sample processing, cost and availability of facilities, training needs, speed of processing, sensitivity, and precision. However, often cost-efficiency was defined indirectly by stating the criteria that were used for the cost-efficiency assessment, e.g., Nendza (2002) determined cost-efficiency of the method by summing cost categories (personnel costs, required equipment and materials) and relating them to the relevance and representativeness of the method.

#### Cost–benefit ratio

Cost–benefit ratios apply a mathematical formula that consists of certain cost and benefit parameters. For instance, Souza and Barros (2015) obtained a cost–benefit ratio by calculating the precision and the cost for each method so that lower cost and higher precision generated a small value indicating a better cost–benefit ratio*.*

#### Comparative studies based on an experiment

The cost-efficiency argument was sometimes based on a systematic comparison of two or more methods. This was done either by conducting an experiment comparing the methods or through a literature review. The more common were comparative studies based on an experiment, which applied the methods and compared their costs and/or ability to measure a parameter efficiently. The methods selected for comparison included either all existing methods or only the most commonly used ones to monitor a certain parameter. However, the inclusion criteria used for selecting methods for the comparisons were rarely explained.

Some articles dedicated a section for cost–benefit analysis of the compared methods. The most cost-efficient method was determined based on predetermined criteria, e.g., sampling effort, accuracy, and cost per sample. Despite the term “cost–benefit analysis,” the method is essentially the same as for other comparative studies that were based on an experiment. Bennett et al. (2016) used the term “cost–benefit optimization” for determining the most cost-efficient method through calculating the staff hours required to complete the survey.

#### Comparative studies based on a literature review

The cost-efficiency assessments were also sometimes the result of a comparative study that sought to present current knowledge on the available methods. Such articles did not report original experiments or present novel methods but determined the most cost-efficient method by reviewing previous studies (e.g., Mallet et al., 2014; Ryan et al., 2009; Wong et al., 2012). These literature reviews were the least common of all assessment methods.

#### Comparisons based on literature

In addition to a literature review, we identified another, more common, category of literature-based comparison method that justified the cost-efficiency assessment by citing previous studies. This kind of comparison was often done in a brief, non-systematic way, and was thus essentially different from a comprehensive literature review. The former often provided a brief reference to “current methods” with a citation to previous studies that included a description of the disadvantages of the methods. Some of these articles focused on describing the inadequacy of current methods and then presented a new method as implicitly more cost-efficient. Such comparisons were often vague and lacked citations to other studies but were sometimes supported by other studies that described current methods and their drawbacks.

#### Subjective comparisons based on experience/intuition

Comparisons with other methods were sometimes also made subjectively without any formal citations to other studies. It is reasonable to assume that such articles based the comparison on the authors’ prior experience with the methods or the authors’ intuition about the assumed costs and benefits of the method. In most of such articles, the reasons for the cost-efficiency of a monitoring method were not clearly defined but, rather, were indirectly implied. In some of these articles, the comparison with other methods was a reference to “current” or “traditional” methods that were deemed costly or laborious and therefore not cost-efficient. Some authors argued that new, cost-efficient methods were needed and presented a new method that was argued to be less costly and time-consuming. While such subjective indirect comparisons lacked a formal assessment of costs and benefits of the method, they often claimed that the new method would constitute an improvement over previous ones.

### Monitoring methods

The monitoring methods in this study included a wide variety of tasks. Twenty-seven percent of the methods were remote sensing techniques; 20% sampling procedures; 13% genomics (eDNA metabarcoding, qPCR); 13% other laboratory methods; 13% biological methods; 11% devices, sensors, or systems; 8% underwater cameras; and 4% citizen science. This division of methods serves as an approximate categorization as a method can, for example, be both remote sensing and utilize an underwater camera. Some of the categories also entailed a broad range of methods, e.g., biological methods included bioassays, biosensors, bioindicators and taxonomy surrogates, and remote sensing included satellites, unmanned vehicles, and automated monitoring.

**Fig. 3:**
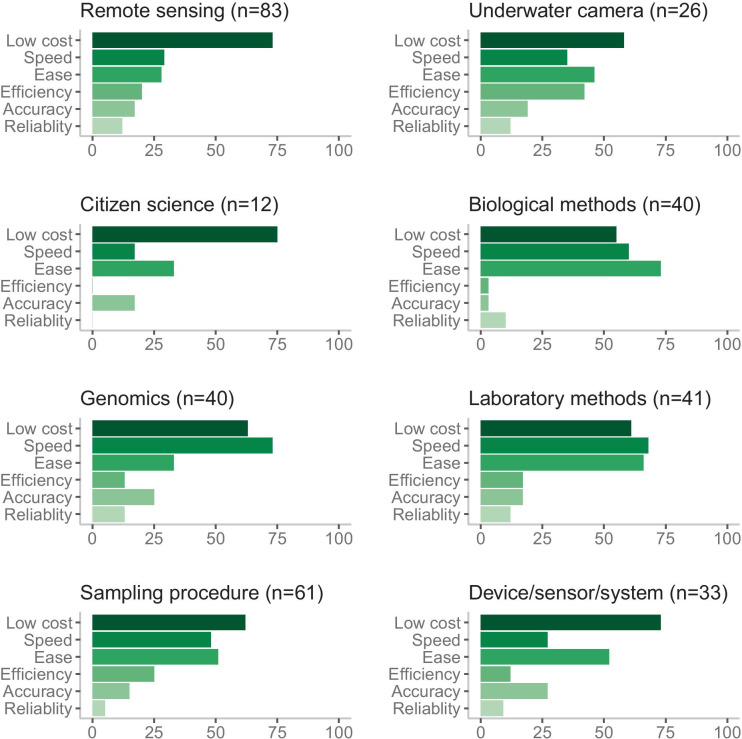
Justifications for cost-efficiency in different monitoring method categories

The justifications for dubbing a method cost-efficient varied across the method categories (Fig. 3). None of the 12 citizen science articles based their cost-efficiency arguments on efficiency or reliability while underwater camera methods were most often described as efficient and genomics as reliable. Cost was the most prevalent reason for cost-efficiency in most categories, with the exception of biological and laboratory methods which had more mentions of speed and ease and genomics which had a greater emphasis on speed than cost.

## Discussion

This literature mapping revealed that while the concept of cost-efficiency is used increasingly often in scientific literature discussing marine monitoring (Fig. 1), there are only a few examples of rigorous cost-efficiency assessment. The observed insufficiencies in the cost-efficiency assessments applied to all five cost-efficiency assessment methods found in this study. Articles that combined method comparison studies with cost-efficiency ratios were generally most transparent in their cost-efficiency arguments—the cost-efficiency parameters were stated clearly and there was no ambiguity concerning which method or methods the novel method was compared to. Notable examples of comprehensive cost assessments can be found in the studies by Harper et al. (2018) and Hulley et al. (2019). Harper et al. (2018) evaluated the cost and investigator effort (time spent) for both of the compared methods and presented them in a picture depicting the methodological steps with corresponding costs. Similarly, Hulley et al. (2019) provided the costs and time spent per sample, specifying costs and prep and run time for each component of the methodology.

Even though cost-efficiency assessment, understood strictly, by definition entails both the costs and benefits associated with obtaining the data, many studies based their cost-efficiency argument only on one of these components, either using the term cost-effectiveness as a synonym for “low cost” or claiming that the improved quality (such as accuracy) of the collected data means the method is cost-efficient. While these cheaper or more accurate methods may indeed be more cost-efficient than the established methods they are compared to, this can only be concluded after considering the effectiveness part of the monitoring, i.e., the usefulness of the collected data. Therefore, it is easy to show that collecting similar data than what is being used already but with lower costs is more cost-effective, but if the newly proposed method yields data that is different from the currently used data, it also has to be shown that it is useful for its intended purpose such as environmental management. Improved data quality or quantity is useful only if it has the potential to improve the decisions made based on the data. Therefore, the effectiveness argument also needs to show that the improved data quality is useful to the end-users of the data. Multiple factors complicate the assessment of costs and effectiveness of marine monitoring. On the costs side, the relationship between the cost-per-sample and information-per-sample is crucial—for monitoring requiring a high number of samples to gain useful information, lower unit cost per sample is needed for the same cost-efficiency. Also, the relationship between investment and operating costs also needs to take into account the expected amount of information gained during the investment’s life span. The efficiency of the obtained data, on the other hand, depends crucially on the needs of the end user, who may again be bound by laws and agreements to assess certain parameters, spatial or temporal scales. This ties the cost-effectiveness of monitoring tightly to the local legislature as well as to the technological and economic considerations.

In cases where the established monitoring is proposed to be replaced by a new, more cost-efficient method, method comparison studies can provide critical information about method performance and the comparability of collected data. Ideally, such comparative studies should also provide exact descriptions of cost and efficiency derived from a comparative experiment or a systematic literature review. In this review, most articles did not include any monetary assessment, and those that did often only covered some component (e.g., equipment or labor costs) of the method instead of all costs related to method implementation. This may at least partly be explained by the sheer difficulty of calculating the costs of a method in their entirety. Similarly, Nygård et al. (2016) found that data on monitoring costs are difficult to obtain and that calculating the exact costs even of the different established components of marine monitoring programs is often complicated.

While method comparison studies are essential for the assessment of cost-efficiency of a method, they are not applicable when a method is applied to monitor an entirely new parameter. In such a case, the cost-efficiency of a monitoring method is relative to the usefulness of the obtained data and the costs to acquire such data. High costs may therefore be justifiable when a method is utilized to measure a new, necessary type of data that cannot be obtained by other means. Novel methods may also lack counterparts in previous methods that would allow a method comparison requisite for a cost-efficiency assessment. The present study may therefore not represent such novel monitoring methods, posing a possible limitation to the study.

## Proposal for an improved cost-effectiveness assessment framework

This study shows that there is no established methodology for assessing cost-efficiency of marine monitoring methods. To aid managers’ work of selecting the best and most cost-efficient methods to their monitoring programs, the comparability and transparency of cost-efficiency assessment need to be improved. Therefore, we propose a cost-effectiveness assessment framework that can be applied to present the cost-effectiveness of a newly proposed monitoring method. The framework is comprehensive, but showing even part of these components, and clearly indicating which information is missing, would be useful.

Cost-efficiency assessments should entail descriptions of both the expenses and benefits of the method. These descriptions should be presented for both the novel method and the method or methods that it is compared to. Further, descriptions of costs and benefits of the methods should ideally cover the entire process chain of the methodology, e.g., from sampling and analysis to data management. We recommend the implementation of cost per sample or cost per survey site as comparable assessment criteria for the cost of the method. In addition to the cost of the method, the spatial and temporal coverage in relation to the end-users’ needs regarding the specific parameters should be evaluated for both the novel method and methods it is compared to.

When calculating the cost of the method we propose considering all the costs associated with the method, including (i) price of the data (if bought from third parties); (ii) equipment cost: the price of the equipment divided by the expected number of samples processed by the equipment during its lifetime, cost of expendable supplies per sample; (iii) personnel costs needed to process one sample; (iv) time spent on sampling and data management; and (v) other costs: transport of samples, training the personnel, keeping up their proficiency, maintaining a website for citizen observations, etc., as well as (vi) possible savings if the method could replace another, currently used monitoring method (Table 2). The cost of infrastructure related to monitoring, such as satellites, laboratory facilities, or vessels of opportunity have not been included into this framework, as they usually exist regardless of any individual monitoring method and dividing their costs to specific methods would be both difficult and intractable.

**Table 2:** A proposal for unified cost criteria to be used in the cost-efficiency assessments of novel monitoring methods

Cost factor	Definition
Type I: Price of data	Price of data if bought from third parties
Type II: Equipment and supplies	Cost of equipment, cost of expendable supplies per sample
Type III: Personnel	Personnel costs needed to process one sample or a study site
Type IV: Time	Time spent on sampling and data management
Type V: Other costs	E.g., transport of samples, training the personnel and keeping up their proficiency, maintaining a website for citizen science observations
Type VI: Savings	Savings gained by replacing an existing method Required analysis: worktime (% compared with the present method), required skills (compared with the present method) and total costs (compared with the present method)

As with the cost assessment, we propose the application of a comprehensive set of criteria to determine the efficiency of a method, including (i) the amount of yearly samples needed for an accurate assessment, (ii) the number of indicators that can be assessed using this method, (iii) the level of confidence in the obtained data, and (iv) evaluation of how well the obtained data fits the requirements (Table 3).

**Table 3:** A proposal for unified efficiency criteria to be used in the cost-efficiency assessments of novel monitoring methods

Efficiency factor	Definition
Type I: Number of samples needed	Aim: keeping the representativeness at an acceptable level compared to the present method Required analysis: the minimum amount of samples/data based on documented sources
Type II: The number of indicators that can be assessed	Does the method allow monitoring of several indicators at once?
Type III: The level of confidence in the obtained data	Aim: keeping the reliability at an acceptable and stable level compared to the present method Required analysis: accuracy and consistency of the data based, e.g., on the technology readiness level (TRL)
Type IV: Value of data	How well does the data fit the requirements of an indicator compared with the present method? Ultimately, the true value of monitoring data comes from the benefits gained from its use. Thus, even a rapid and low-cost method is not truly cost-efficient if the data is not representative of the environmental factor that is being monitored.

This information will give a fair and transparent picture of the costs and efficiency of the data obtained using any monitoring method and allows alternative methods to be compared. Even if the assessed monitoring method addresses a newly monitored parameter, and no alternative methods exist, the proposed framework will give a comprehensive picture of the costs and effectiveness of the gathered data.

It should be recognized that carrying out a comprehensive cost-efficiency assessment may be beyond the scope of studies presenting a novel monitoring method. As concluded by Nygård et al. (2016), costs of monitoring are often difficult to obtain. An assessment of method efficiency is arguably even more complex. However, this should not discourage researchers from engaging in assessing the cost-efficiency of novel methods, as even a partial assessment, with a fair description of its shortcomings, is more informative than no assessment. Future studies are needed to expand and test the recommendations for evaluating cost-efficiency presented in this study. As marine monitoring entails a very wide range of monitoring tasks, technologies, and objectives, task and objective-specific assessment frameworks may be needed.

## Supplementary Information

Below is the link to the electronic supplementary material.

Supplementary file1 (DOCX 247 KB)

Supplementary file2 (DOCX 55 KB)

Supplementary file3 (XLSX 210 KB)

## Data Availability

The following supplementary material will be made available online: i) Annex 1: a reference list of the 1684 articles yielded by the Scopus and Web of Science search on 21 March 2019 (articles of the subset A), ii) Annex 2: a reference list of the 313 articles that included a cost-efficiency assessment method and thus, contributed to the knowledge base (articles of the subset C) and iii) Annex 3: a data table that includes full information collected from the articles of the subset C.
