# Supplementary material for: Cost-efficiency assessments of marine monitoring methods lack rigor—a systematic mapping of literature and an end-user view on optimal cost-efficiency analysis
Source: Environ Monit Assess. 2021 Jun 9;193(7):400. doi: 10.1007/s10661-021-09159-y (PMC8187199; doi:10.1007/s10661-021-09159-y)
Supplement: Supplementary file 1 — Supplementary file1 (DOCX 247 KB) [file 10661_2021_9159_MOESM1_ESM.docx]

Annex 1. Scopus and Web of Science databases were queried via CADIMA on March 21^st^ 2019. The search yielded a total of 1684 articles.

1. 2nd International Conference on Materials Engineering and Automatic Control, ICMEAC 2013. 2013. Applied Mechanics and Materials.
2. 7th International Conference on River Basin Management, RBM 2013, 2013. WIT Transactions on Ecology and the Environment 172.
3. Aarnio, K., Mattila, J., Bonsdorff, E., 2011. Comparison of different sampling strategies in monitoring zoobenthos and classification of archipelago areas. Boreal Environment Research 16, 395–406.
4. Abayazid, H., 2019. Changes in a Coastal Lake Dynamic System and Potential Restoration, Handbook of Environmental Chemistry. https://doi.org/10.1007/698_2017_104
5. Abdelzaher, A.M., Solo-Gabriele, H.M., Palmer, C.J., Scott, T.M., 2009. Simultaneous concentration of enterococci and coliphage from marine waters using a dual layer filtration system. Journal of Environmental Quality 38, 2468–2473. https://doi.org/10.2134/jeq2008.0488
6. Abdulgader, H.A., Kochkodan, V., Hilal, N., 2013. Hybrid ion exchange - Pressure driven membrane processes in water treatment: A review. Separation and Purification Technology 116, 253–264. https://doi.org/10.1016/j.seppur.2013.05.052
7. Abdullah, A.L., Anscelly, A.A., Mohamed, J., Yasin, Z., 2016. Conservation of pulau payar marine park and optical remote sensing models. Kemanusiaan 23, 79–107.
8. Abebe, Y., Bitew, M., Ayenew, T., Alo, C., Cherinet, A., Dadi, M., 2018. Morphometric change detection of Lake Hawassa in the Ethiopian Rift Valley. Water (Switzerland) 10. https://doi.org/10.3390/w10050625
9. Aboulghar, M., 2010. Coasting: What is the cost? Current Women’s Health Reviews 6, 239–244. https://doi.org/10.2174/157340410792007145
10. Abramic, A., Martínez-Alzamora, N., González del Rio Rams, J., Barrachina, T., Polo, J.F., 2014. New methodology for analysing and increasing the cost-efficiency of environmental monitoring networks. Marine Pollution Bulletin 86, 161–173. https://doi.org/10.1016/j.marpolbul.2014.07.029
11. Abreu, I.O., Monteiro, C., Rocha, A.C.S., Reis-Henriques, M.A., Teixeira, C., Pires Basto, M.C., Ferreira, M., Almeida, C.M.R., Oliva-Teles, L., Guimarães, L., 2018. Data for the analysis of interactive multibiomarker responses of a marine crustacean to long-term exposure to aquatic contaminants. Data in Brief 21, 386–394. https://doi.org/10.1016/j.dib.2018.09.055
12. Absametov, M., Kan, S., Kurmangaliyeva, S., Asanov, Y., 2015. THE ROLE OF “EURASIA” CHANNEL IN THE DEVELOPMENT OF TRANSIT POTENTIAL REPUBLIC OF KAZAKHSTAN. Bulletin of the National Academy of Sciences of the Republic of Kazakhstan 68-+.
13. Acevo-Herrera, R., Aguasca, A., Bosch-Lluis, X., Camps, A., Martínez-Fernández, J., Sánchez-Martín, N., Pérez-Gutiérrez, C., 2010. Design and first results of an UAV-borne L-band radiometer for multiple monitoring purposes. Remote Sensing 2, 1662–1679. https://doi.org/10.3390/rs2071662
14. Adams, K., Pennock, N., Phelps, B., Rose, W., Peters, M., 2007. Anesthesia services outside of the operating room. Pediatric nursing 33, 232, 234, 236–2.
15. Adams, V.M., Pressey, R.L., Stoeckl, N., 2012. Estimating land and conservation management costs: The first step in designing a stewardship program for the Northern Territory. Biological Conservation 148, 44–53. https://doi.org/10.1016/j.biocon.2012.01.064
16. Agostinho, M., Moreira-Santos, M., Ribeiro, R., 2012. A freshwater amphipod toxicity test based on postexposure feeding and the population consumption inhibitory concentration. Chemosphere 87, 43–48. https://doi.org/10.1016/j.chemosphere.2011.11.052
17. Aguado-Giménez, F., Marín, A., Montoya, S., Marín-Guirao, L., Piedecausa, A., García-García, B., 2007. Comparison between some procedures for monitoring offshore cage culture in western Mediterranean Sea: Sampling methods and impact indicators in soft substrata. Aquaculture 271, 357–370. https://doi.org/10.1016/j.aquaculture.2007.03.026
18. Ahkola, H., Herve, S., Knuutinen, J., 2014. Study of different Chemcatcher configurations in the monitoring of nonylphenol ethoxylates and nonylphenol in aquatic environment. Environmental Science and Pollution Research 21, 9182–9192. https://doi.org/10.1007/s11356-014-2828-5
19. Ahmad, A., Dada, A.C., Usup, G., Heng, L.Y., 2013. Validation of the Enterococci indicator for bacteriological quality monitoring of beaches in Malaysia using a multivariate approach. Springerplus 2. https://doi.org/10.1186/2193-1801-2-425
20. Ahmad, M., Ali, M., Imran, M., Hashem, Z., Anas, S.M., Hussain, S., Khan, A.S., Yousuf, B.M., 2019. Remotely operated underwater vehicle (ROV) using wireless communication protocol over a floating unit, Advances in Intelligent Systems and Computing. https://doi.org/10.1007/978-3-030-02683-7_66
21. Ahmed, M., Abdelmohsen, K., 2018. Quantifying Modern Recharge and Depletion Rates of the Nubian Aquifer in Egypt. Surveys in Geophysics 39, 729–751. https://doi.org/10.1007/s10712-018-9465-3
22. Ahnert, A., Schriever, G., 2001. Response of abyssal copepoda Harpacticoida (crustacea) and other meiobenthos to an artificial disturbance and its bearing on future mining for polymetallic nodules. Deep-Sea Research Part II: Topical Studies in Oceanography 48, 3779–3794. https://doi.org/10.1016/S0967-0645(01)00067-4
23. Ai, J., Gao, W., Gao, Z., Shi, R., Zhang, C., Liu, C., 2016. Integrating pan-sharpening and classifier ensemble techniques to map an invasive plant (Spartina alterniflora) in an estuarine wetland using Landsat 8 imagery. Journal of Applied Remote Sensing 10. https://doi.org/10.1117/1.JRS.10.026001
24. Ainslie, M.A., 2013. Neglect of bandwidth of Odontocetes echo location clicks biases propagation loss and single hydrophone population estimates. Journal of the Acoustical Society of America 134, 3506–3512. https://doi.org/10.1121/1.4823804
25. Ainsworth, C., 2008. Ferryboxes begin to make waves. Science 322, 1627–1629. https://doi.org/10.1126/science.322.5908.1627
26. Ait-Khaled, N., Auregan, G., Bencharif, N., Camara, L.M., Dagli, E., Djankine, K., Keita, B., Ky, C., Mahi, S., Ngoran, K., Pham, D.L., Sow, O., Yousser, M., Zidouni, N., Enarson, D.A., 2000. Affordability of inhaled corticosteroids as a potential barrier to treatment of asthma in some developing countries. International Journal of Tuberculosis and Lung Disease 4, 268–271.
27. Akbar, T.A., Hassan, Q.K., Achari, G., 2011. A Methodology for Clustering Lakes in Alberta on the basis of Water Quality Parameters. Clean - Soil, Air, Water 39, 916–924. https://doi.org/10.1002/clen.201100050
28. Akimbekov, N.S., Yernazarova, A.K., Tastambek, K.T., Abdieva, G.Z., Ualieva, P.S., Kaiyrmanova, G.K., Djansugurova, L.B., Zhubanova, A.A., 2017. Microbial load as ecotoxicological assessment of heavy metals presence in soil samples from the Kazakhstan part of the caspian sea. Eurasian Chemico-Technological Journal 19, 335–340. https://doi.org/10.18321/ectj681
29. Akumu, A.O., English, M., Scott, J.A.G., Griffiths, U.K., 2007. Economic evaluation of delivering Haemophilus influenzae type b vaccine in routine immunization services in Kenya. Bulletin of the World Health Organization 85, 511–518. https://doi.org/10.2471/BLT.06.034686
30. Alahuhta, J., Luukinoja, J., Tukiainen, H., Hjort, J., 2016. Importance of spatial scale in structuring emergent lake vegetation across environmental gradients and scales: GIS-based approach. Ecological Indicators 60, 1164–1172. https://doi.org/10.1016/j.ecolind.2015.08.045
31. Alendal, G., 2017. Cost efficient environmental survey paths for detecting continuous tracer discharges. Journal of Geophysical Research-Oceans 122, 5458–5467. https://doi.org/10.1002/2016jc012655
32. Ali, A., Lobinske, R.J., Leckel, R.J., Carandang, N., Mazumdar, A., 2008. Population survey and control of Chironomidae (Diptera) in wetlands in northeast florida, USA. Florida Entomologist 91, 446–452. https://doi.org/10.1653/0015-4040(2008)91[446:psacoc]2.0.co;2
33. Alkan, R.M., Saka, M.H., Ozulu, İ.M., İlçi, V., 2017. Kinematic precise point positioning using GPS and GLONASS measurements in marine environments. Measurement: Journal of the International Measurement Confederation 109, 36–43. https://doi.org/10.1016/j.measurement.2017.05.054
34. Alkotaini, B., Tinucci, S.L., Robertson, S.J., Hasan, K., Minteer, S.D., Grattieri, M., 2018. Alginate-Encapsulated Bacteria for the Treatment of Hypersaline Solutions in Microbial Fuel Cells. Chembiochem 19, 1162–1169. https://doi.org/10.1002/cbic.201800142
35. Allan, M.G., Hamilton, D.P., Hicks, B., Brabyn, L., 2015. Empirical and semi-analytical chlorophyll a algorithms for multi-temporal monitoring of New Zealand lakes using Landsat. Environmental Monitoring and Assessment 187. https://doi.org/10.1007/s10661-015-4585-4
36. Allender, S., Millar, L., Hovmand, P., Bell, C., Moodie, M., Carter, R., Swinburn, B., Strugnell, C., Lowe, J., De La Haye, K., Orellana, L., Morgan, S., 2016. Whole of systems trial of prevention strategies for childhood obesity: WHO STOPS childhood obesity. International Journal of Environmental Research and Public Health 13. https://doi.org/10.3390/ijerph13111143
37. Al-Nabulsi, K.M., Rizk, T.Y., Al-Abbas, F.M., Dias, O.C., 2016. Sea water cooler tubes corrosion and leaks due to microbiologically induced corrosion, NACE - International Corrosion Conference Series.
38. Alquezar, R., Boyd, W., 2007. Development of rapid, cost effective coral survey techniques: Tools for management and conservation planning. Journal of Coastal Conservation 11, 105–119. https://doi.org/10.1007/s11852-008-0011-1
39. Al-Shawaf, T., Grudzinskas, J.G., 2003. Prevention and treatment of ovarian hyperstimulation syndrome. Best Practice & Research in Clinical Obstetrics & Gynaecology 17, 249–261. https://doi.org/10.1053/ybeog.2003.349
40. Alvarez-Guerra, M., Viguri, J.R., Casado-Martínez, M.C., DelValls, T.A., 2007. Sediment quality assessment and dredged material management in Spain: Part II, analysis of action levels for dredged material management and application to the Bay of Cádiz. Integrated environmental assessment and management 3, 539–551. https://doi.org/10.1897/IEAM_2007-016.1
41. Amde, M., Liu, J.F., Tan, Z.Q., Bekana, D., 2016. Ionic liquid-based zinc oxide nanofluid for vortex assisted liquid liquid microextraction of inorganic mercury in environmental waters prior to cold vapor atomic fluorescence spectroscopic detection. Talanta 149, 341–346. https://doi.org/10.1016/j.talanta.2015.12.004
42. Amin, R., Richards, B.L., Misa, W., Taylor, J.C., Miller, D.R., Rollo, A.K., Demarke, C., Singh, H., Young, G.C., Childress, J., Ossolinski, J.E., Reardon, R.T., Koyanagi, K.H., 2017. The Modular Optical Underwater Survey System. Sensors 17. https://doi.org/10.3390/s17102309
43. Amoako-Atta, S., Kuntu-Mensah, P., 2007. A GIS data modeling approach to port ballast water bioinvasion. Surveying and Land Information Science 67, 43–50.
44. Anastasopoulos, D., Kolios, S., Stylios, C., 2011. How will Greek ports become green ports? Geo-Eco-Marina 17, 73–80.
45. Andersen, M., Juhler, S., Tang, L., 2015. Implementation of new test system for optimizing the performance of chemical mitigation strategies against MIC in pipelines, NACE - International Corrosion Conference Series.
46. Anderson, J.W., Hartwell, S.I., Hameed, M.J., 2005. Regional comparisons of coastal sediment contamination detected by a biomarker (P450 HRGS; EPA Method 4425). Environmental Science and Technology 39, 17–23. https://doi.org/10.1021/es049283b
47. Anderson, L.G., Chapman, J.K., Escontrela, D., Gough, C.L.A., 2017. The role of conservation volunteers in the detection, monitoring and management of invasive alien lionfish. Management of Biological Invasions 8, 589–598. https://doi.org/10.3391/mbi.2017.8.4.14
48. Anderson, T.J., Nichol, S.L., Syms, C., Przeslawski, R., Harris, P.T., 2011. Deep-sea bio-physical variables as surrogates for biological assemblages, an example from the Lord Howe Rise. Deep-Sea Research Part II: Topical Studies in Oceanography 58, 979–991. https://doi.org/10.1016/j.dsr2.2010.10.053
49. Andrade, H., Renaud, P.E., 2011. Polychaete/amphipod ratio as an indicator of environmental impact related to offshore oil and gas production along the Norwegian continental shelf. Marine Pollution Bulletin 62, 2836–2844. https://doi.org/10.1016/j.marpolbul.2011.08.032
50. Andrews, J.E., Burgess, D., Cave, R.R., Coombes, E.G., Jickells, T.D., Parkes, D.J., Turner, R.K., 2006. Biogeochemical value of managed realignment, Humber estuary, UK. Science of the Total Environment 371, 19–30. https://doi.org/10.1016/j.scitotenv.2006.08.021
51. Anitha, A., Rajesh, M., 2017. Programmable logic controller based control of tidal power plant. Journal of Advanced Research in Dynamical and Control Systems 9.
52. Anon, 2000a. Four-component data for imaging, lithology prediction, and fluid discrimination. JPT, Journal of Petroleum Technology 52, 24–25.
53. Anon, 2000b. Sea Oracle AUV under development. Journal of Offshore Technology 8, 24–25.
54. Anthony, E.J., Vanhee, S., Ruz, M.H., 2007. An assessment of the impact of experimental brushwood fences on foredune sand accumulation based on digital elelvation models. Ecological Engineering 31, 41–46. https://doi.org/10.1016/j.ecoleng.2007.05.005
55. Arashiro, L.T., Montero, N., Ferrer, I., Acién, F.G., Gómez, C., Garfí, M., 2018. Life cycle assessment of high rate algal ponds for wastewater treatment and resource recovery. Science of the Total Environment 622–623, 1118–1130. https://doi.org/10.1016/j.scitotenv.2017.12.051
56. Ardalan, A.A., Ahmadi-Givi, F., Rezvani, M.H., 2013. Comparison of attitude determination based on four and three onboard GNSS antennae, case study: Hydrographic applications. Journal of the Earth and Space Physics 39, 123–131.
57. Armagan, B., Besli, N., Ucar, D., 2016. An approach for sustainable management of the balikligol lakes, Turkey, in: Plants, Pollutants and Remediation. pp. 371–379. https://doi.org/10.1007/978-94-017-7194-8_18
58. Armitage, A.R., Ho, C.K., Madrid, E.N., Bell, M.T., Quigg, A., 2014. The influence of habitat construction technique on the ecological characteristics of a restored brackish marsh. Ecological Engineering 62, 33–42. https://doi.org/10.1016/j.ecoleng.2013.10.021
59. Armstrong, D.P., Perrott, J.K., Castro, I., 2001. Estimating impacts of poison operations using mark-recapture analysis: Hihi (Notiomystis cincta) on Mokoia Island. New Zealand Journal of Ecology 25, 49–54.
60. Armstrong, M.J., Witthames, P.R., 2012. Developments in understanding of fecundity of fish stocks in relation to egg production methods for estimating spawning stock biomass. Fisheries Research 117–118, 35–47. https://doi.org/10.1016/j.fishres.2010.12.028
61. Armstrong, S., Ai, Y., 2013. Qualified soundings from interferometric bathymetry. Sea Technology 54, 35–38.
62. Arnold, W.R., Warren-Hicks, W.J., 2007. Probability-based estimates of site-specific copper water quality criteria for the Chesapeake Bay, USA. Integrated environmental assessment and management 3, 101–117. https://doi.org/10.1897/1551-3793(2007)3[101:PEOSCW]2.0.CO;2
63. Artemiadou, V., Lazaridou, M., 2005. Evaluation score and interpretation index for the ecological quality of running waters in central and northern Hellas. Environmental Monitoring and Assessment 110, 1–40. https://doi.org/10.1007/s10661-005-6289-7
64. Arthur, N., 2005. Development, implementation a application of a Condition Based Maintenance assessment tool for the UK Oil and Gas Industry. International Journal of COMADEM 8, 2–9.
65. Asaf, L., Negaoker, N., Alon, T., Laronne, J., Al Khateeb, N., 2006. Transboundary stream restoration in Israel and the Palestinian Authority, NATO Security through Science Series C: Environmental Security. https://doi.org/10.1007/978-1-4020-5986-5_13
66. Asami, S., 2005. Applications of motor-powered paragliders. Sea Technology 46, 57–58.
67. Ashraf, S., Brabyn, L., Hicks, B.J., Collier, K., 2010. Satellite remote sensing for mapping vegetation in New Zealand freshwater environments: A review. New Zealand Geographer 66, 33–43. https://doi.org/10.1111/j.1745-7939.2010.01168.x
68. Assilzadeh, H., Levy, J.K., Wang, X., Gao, Y., Zhong, Z., 2010. Geosensing systems engineering for ocean security and sustainable coastal zone management. Journal of Systems Science and Systems Engineering 19, 22–35. https://doi.org/10.1007/s11518-010-5123-0
69. Assoumani, A., Coquery, M., Liger, L., Mazzella, N., Margoum, C., 2015. Field application of passive SBSE for the monitoring of pesticides in surface waters. Environmental Science and Pollution Research 22, 3997–4008. https://doi.org/10.1007/s11356-014-3590-4
70. Atalah, J., Crowe, T.P., 2012. Nutrient enrichment and variation in community structure on rocky shores: The potential of molluscan assemblages for biomonitoring. Estuarine Coastal and Shelf Science 99, 162–170. https://doi.org/10.1016/j.ecss.2011.12.034
71. Atkinson, J., Esteves, L.S., Williams, J.W., McCann, D.L., Bell, P.S., 2018. The Application of X-Band Radar for Characterization of Nearshore Dynamics on a Mixed Sand and Gravel Beach. Journal of Coastal Research 85, 281–285. https://doi.org/10.2112/SI85-057.1
72. Au, D.W.T., 2004. The application of histo-cytopathological biomarkers in marine pollution monitoring: A review. Marine Pollution Bulletin 48, 817–834. https://doi.org/10.1016/j.marpolbul.2004.02.032
73. Aubert, A., Antajan, E., Lynam, C., Pitois, S., Pliru, A., Vaz, S., Thibault, D., 2018. No more reason for ignoring gelatinous zooplankton in ecosystem assessment and marine management: Concrete cost-effective methodology during routine fishery trawl surveys. Marine Policy 89, 100–108. https://doi.org/10.1016/j.marpol.2017.12.010
74. Aubin, J., Tocqueville, A., Kaushik, S.J., 2011. Characterisation of waste output from flow-through trout farms in France: comparison of nutrient mass-balance modelling and hydrological methods. Aquatic Living Resources 24, 63–70. https://doi.org/10.1051/alr/2011008
75. Avelar, S., Tokarczyk, P., 2014. Analysis of land use and land cover change in a coastal area of Rio de Janeiro using high-resolution remotely sensed data. Journal of Applied Remote Sensing 8. https://doi.org/10.1117/1.JRS.8.083631
76. Avery-Gomm, S., O’Hara, P.D., Kleine, L., Bowes, V., Wilson, L.K., Barry, K.L., 2012. Northern fulmars as biological monitors of trends of plastic pollution in the eastern North Pacific. Marine Pollution Bulletin 64, 1776–1781. https://doi.org/10.1016/j.marpolbul.2012.04.017
77. Aykanat, T., Lindqvist, M., Pritchard, V.L., Primmer, C.R., 2016. From population genomics to conservation and management: a workflow for targeted analysis of markers identified using genome-wide approaches in Atlantic salmon Salmo salar. Journal of Fish Biology 89, 2658–2679. https://doi.org/10.1111/jfb.13149
78. Aylagas, E., Borja, Á., Irigoien, X., Rodríguez-Ezpeleta, N., 2016. Benchmarking DNA metabarcoding for biodiversity-based monitoring and assessment. Frontiers in Marine Science 3. https://doi.org/10.3389/fmars.2016.00096
79. Aylesworth, L., Phoonsawat, R., Suvanachai, P., Vincent, A.C.J., 2017. Generating spatial data for marine conservation and management. Biodiversity and Conservation 26, 383–399. https://doi.org/10.1007/s10531-016-1248-x
80. Azemard, S., Vassileva, E., 2015. Determination of methylmercury in marine biota samples with advanced mercury analyzer: Method validation. Food Chemistry 176, 367–375. https://doi.org/10.1016/j.foodchem.2014.12.085
81. Azevedo-Linhares, M., Freire, C.A., 2015. Evaluation of impacted Brazilian estuaries using the native oyster Crassostrea rhizophorae: Branchial carbonic anhydrase as a biomarker. Ecotoxicology and Environmental Safety 122, 483–489. https://doi.org/10.1016/j.ecoenv.2015.09.027
82. Aziz, A.A., Phinn, S., Dargusch, P., Omar, H., Arjasakusuma, S., 2015. Assessing the potential applications of Landsat image archive in the ecological monitoring and management of a production mangrove forest in Malaysia. Wetlands Ecology and Management 23, 1049–1066. https://doi.org/10.1007/s11273-015-9443-1
83. Babcock, D.A., Wawrik, B., Paul, J.H., McGuinness, L., Kerkhof, L.J., 2007. Rapid screening of a large insert BAC library for specific 16S rRNA genes using TRFLP. Journal of Microbiological Methods 71, 156–161. https://doi.org/10.1016/j.mimet.2007.07.015
84. Baeté, C., 2015. ICCP retrofit challenges for an offshore jacket complex, NACE - International Corrosion Conference Series.
85. Bagi, A., Soelberg, S.D., Furlong, C.E., Baussant, T., 2018. Implementing morpholino-based nucleic acid sensing on a portable surface plasmon resonance instrument for future application in environmental monitoring. Sensors (Switzerland) 18. https://doi.org/10.3390/s18103259
86. Bailey, D.E., Bulleit, K.A., 2002. Defining “adverse environmental impact” and making paragraph 316(b) decisions: a fisheries management approach. TheScientificWorldJournal 2 Suppl 1, 147–168.
87. Bailey, H., Clay, G., Coates, E.A., Lusseau, D., Senior, B., Thompson, P.M., 2010. Using T-PODs to assess variations in the occurrence of coastal bottlenose dolphins and harbour porpoises. Aquatic Conservation: Marine and Freshwater Ecosystems 20, 150–158. https://doi.org/10.1002/aqc.1060
88. Bailey, J.E., Calhoun, A.J.K., 2008. Comparison of three physical management techniques for controlling variable-leaf milfoil in Maine Lakes. Journal of Aquatic Plant Management 46, 163–167.
89. Baker Jr, M.S., Oeschger, I., 2009. Description and initial evaluation of a text message based reporting method for marine recreational anglers. Marine and Coastal Fisheries 1, 143–154. https://doi.org/10.1577/C08-042.1
90. Baker, C.M., Hodgson, J.C., Tartaglia, E., Clarke, R.H., 2017. Modelling tropical fire ant (Solenopsis geminata) dynamics and detection to inform an eradication project. Biological Invasions 19, 2959–2970. https://doi.org/10.1007/s10530-017-1499-9
91. Baker, E.T., Walker, S.L., Embley, R.W., de Ronde, C.E.J., 2012. High-Resolution Hydrothermal Mapping of Brothers Caldera, Kermadec Arc. Economic Geology 107, 1583–1593. https://doi.org/10.2113/econgeo.107.8.1583
92. Baker, L.F., Artym, K.J., Swanson, H.K., 2017. Optimal sampling methods for modelling the occupancy of Arctic grayling (Thymallus arcticus) in the Canadian Barrenlands. Canadian Journal of Fisheries and Aquatic Sciences 74, 1564–1574. https://doi.org/10.1139/cjfas-2016-0429
93. Baker, R.A., Culver, T.B., 2010. Locating nested monitoring wells to reduce model uncertainty for management of a multilayer coastal aquifer. Journal of Hydrologic Engineering 15, 763–771. https://doi.org/10.1061/(ASCE)HE.1943-5584.0000247
94. Baldantoni, D., Maisto, G., Bartoli, G., Alfani, A., 2005. Analyses of three native aquatic plant species to assess spatial gradients of lake trace element contamination. Aquatic Botany 83, 48–60. https://doi.org/10.1016/j.aquabot.2005.05.006
95. Baldera, A., Hanson, D.A., Kraft, B., 2018. Selecting indicators to monitor outcomes across projects and multiple restoration programs in the Gulf of Mexico. Ecological Indicators 89, 559–571. https://doi.org/10.1016/j.ecolind.2018.01.025
96. Balfour, C.A., 2012. Cost-effective remote data acquisition and instrumentation management for oceanographic and environmental monitoring applications. Journal of Operational Oceanography 5, 41–52. https://doi.org/10.1080/1755876X.2012.11020137
97. Balfour, C.A., Howarth, M.J., Jones, D.S., Doyle, T., 2013. The design and development of an irish sea passenger-ferry-based oceanographic measurement system. Journal of Atmospheric and Oceanic Technology 30, 1226–1239. https://doi.org/10.1175/JTECH-D-12-00223.1
98. Ballouard, J.M., Bonnet, X., Gravier, C., Ausanneau, M., Caron, S., 2016. Artificial water ponds and camera trapping of tortoises, and other vertebrates, in a dry Mediterranean landscape. Wildlife Research 43, 533–543. https://doi.org/10.1071/WR16035
99. Bandeira, J.V., Salim, L.H., Bomtempo, V.L., Moreira, R.M., Brisset, P., Hughes, C.E., Pant, H.J., Thereska, J., Wörman, A., 2014. Nuclear techniques for monitoring sediment dynamics in the coastal zone, in: Engineering Geology for Society and Territory - Volume 4: Marine and Coastal Processes. pp. 151–155. https://doi.org/10.1007/978-3-319-08660-6_28
100. Banerjee, R., Srivastava, P.K., 2013. Reconstruction of contested landscape: Detecting land cover transformation hosting cultural heritage sites from Central India using remote sensing. Land Use Policy 34, 193–203. https://doi.org/10.1016/j.landusepol.2013.03.005
101. Banting, M., Wonnacott, R., 2002. Turbidity curtains secure a contaminated environment. Geotechnical Fabrics Report 20, 18–23.
102. Bardosh, K., Waiswa, C., Welburn, S.C., 2013. Conflict of interest: Use of pyrethroids and amidines against tsetse and ticks in zoonotic sleeping sickness endemic areas of Uganda. Parasites and Vectors 6. https://doi.org/10.1186/1756-3305-6-204
103. Barr, N.G., Dudley, B.D., Rogers, K.M., Cornelisen, C.D., 2013. Broad-scale patterns of tissue-delta N-15 and tissue-N indices in frondose Ulva spp.; Developing a national baseline indicator of nitrogen-loading for coastal New Zealand. Marine Pollution Bulletin 67, 203–216. https://doi.org/10.1016/j.marpolbul.2012.11.033
104. Barr, N.G., Dudley, B.D., Rogers, K.M., Cornelisen, C.D., 2013. Broad-scale patterns of tissue-δ15N and tissue-N indices in frondose Ulva spp.; Developing a national baseline indicator of nitrogen-loading for coastal New Zealand. Marine Pollution Bulletin 67, 203–216. https://doi.org/10.1016/j.marpolbul.2012.11.033
105. Barrio Froján, C.R.S., Cooper, K.M., Bolam, S.G., 2016. Towards an integrated approach to marine benthic monitoring. Marine Pollution Bulletin 104, 20–28. https://doi.org/10.1016/j.marpolbul.2016.01.054
106. Barry, J., Maxwell, D., Jennings, S., Walker, D., Murray, J., 2017. Emon: an R-package to support the design of marine ecological and environmental studies, surveys and monitoring programmes. Methods in Ecology and Evolution 8, 1342–1346. https://doi.org/10.1111/2041-210X.12748
107. Bartholomew, D.C., Mangel, J.C., Alfaro-Shigueto, J., Pingo, S., Jimenez, A., Godley, B.J., 2018. Remote electronic monitoring as a potential alternative to on-board observers in small-scale fisheries. Biological Conservation 219, 35–45. https://doi.org/10.1016/j.biocon.2018.01.003
108. Bartley, D.M., Bell, J.D., 2008. Restocking, stock enhancement, and sea ranching: Arenas of progress. Reviews in Fisheries Science 16, 357–365. https://doi.org/10.1080/10641260701678058
109. Barton, D.N., Andersen, T., Bergland, O., Engebretsen, A., Jannicke Moe, S., Orderud, G.I., Tominaga, K., Romstad, E., Vogt, R.D., 2016. Eutropia: Integrated valuation of lake eutrophication abatement decisions using a bayesian belief network, in: Handbook of Applied System Science. pp. 297–320. https://doi.org/10.4324/9781315748771
110. Barton, M.B., Litvin, S.Y., Vollenweider, J.J., Heintz, R.A., Norcross, B.L., Boswell, K.M., 2019. Experimental determination of tissue turnover rates and trophic discrimination factors for stable carbon and nitrogen isotopes of Arctic Sculpin (Myoxocephalus scorpioides): A common Arctic nearshore fish. Journal of Experimental Marine Biology and Ecology 511, 60–67. https://doi.org/10.1016/j.jembe.2018.11.005
111. Bassil, K.L., Sanborn, M., Lopez, R., Orris, P., 2015. Integrating environmental and human health databases in the great lakes basin: Themes, challenges and future directions. International Journal of Environmental Research and Public Health 12, 3600–3614. https://doi.org/10.3390/ijerph120403600
112. Bastian, T., Haberlin, D., Purcell, J.E., Hays, G.C., Davenport, J., McAllen, R., Doyle, T.K., 2011. Large-scale sampling reveals the spatio-temporal distributions of the jellyfish Aurelia aurita and Cyanea capillata in the Irish Sea. Marine Biology 158, 2639–2652. https://doi.org/10.1007/s00227-011-1762-z
113. Bastos, A.C., Prodana, M., Abrantes, N., Keizer, J.J., Soares, A.M.V.M., Loureiro, S., 2014. Potential risk of biochar-amended soil to aquatic systems: an evaluation based on aquatic bioassays. Ecotoxicology 23, 1784–1793. https://doi.org/10.1007/s10646-014-1344-1
114. Bastviken, D., Sundgren, I., Natchimuthu, S., Reyier, H., Galfalk, M., 2015. Technical Note: Cost-efficient approaches to measure carbon dioxide (CO2) fluxes and concentrations in terrestrial and aquatic environments using mini loggers. Biogeosciences 12, 3849–3859. https://doi.org/10.5194/bg-12-3849-2015
115. Bastviken, D., Sundgren, I., Natchimuthu, S., Reyier, H., Gälfalk, M., 2015. Technical Note: Cost-efficient approaches to measure carbon dioxide (CO2) fluxes and concentrations in terrestrial and aquatic environments using mini loggers. Biogeosciences 12, 3849–3859. https://doi.org/10.5194/bg-12-3849-2015
116. Bateman, I.J., Deflandre-Vlandas, A., Fezzi, C., Hadley, D., Hutchins, M., Lovett, A., Posen, P., Rigby, D., 2007. WFD related agricultural nitrate and phosphate leaching reduction options: Cost estimates derived from farm level survey data & A cost-effectiveness assessment for the Derwent catchment, Working Paper - Centre for Social and Economic Research on the Global Environment.
117. Bates, C.R., Phillips, D.R., Grimm, R., Lynn, H., 2001. The seismic evaluation of a naturally fractured tight gas sand reservoir in the Wind River Basin, Wyoming. Petroleum Geoscience 7, 35–44. https://doi.org/10.1144/petgeo.7.1.35
118. Battistone, M.J., Barker, A.M., Grotzke, M.P., Beck, J.P., Lawrence, P., Cannon, G.W., 2016a. “Mini-Residency” in Musculoskeletal Care: a National Continuing Professional Development Program for Primary Care Providers. Journal of General Internal Medicine 31, 1301–1307. https://doi.org/10.1007/s11606-016-3773-4
119. Battistone, M.J., Barker, A.M., Lawrence, P., Grotzke, M.P., Cannon, G.W., 2016b. Mini-Residency in Musculoskeletal Care: An Interprofessional, Mixed-Methods Educational Initiative for Primary Care Providers. Arthritis Care and Research 68, 275–279. https://doi.org/10.1002/acr.22644
120. Baum, M., Schmitt, M., Wagner, M., Westerlage, C., 2008. Geochemical and microbiological surface investigation in northern Germany indicates interesting hydrocarbon potential. Erdoel Erdgas Kohle 124, OG10–OG14.
121. Baumstark, R., Dixon, B., Carlson, P., Palandro, D., Kolasa, K., 2013. Alternative spatially enhanced integrative techniques for mapping seagrass in Florida’s marine ecosystem. International Journal of Remote Sensing 34, 1248–1264. https://doi.org/10.1080/01431161.2012.721941
122. Bavusi, M., Bianca, M., Izzi, F., Di Leo, P., Parisi, S., Pulice, I., Schiattarella, M., 2015. Methods and Technologies for the Cultural Heritages in Basilicata region: A case-study from the coastal belt of Metapontum. Rendiconti Online Societa Geologica Italiana 34, 101–106. https://doi.org/10.3301/ROL.2015.46
123. Becker, N., Lavee, D., Tavor, T., 2012. Desalinate or divert? Coastal non-market values as a decision tool for an integrated water management policy: The case of the Jordan River basin. Ocean and Coastal Management 64, 27–36. https://doi.org/10.1016/j.ocecoaman.2012.04.008
124. Becker, P., Barringer, C., Marelli, D.C., 2008. Thirty years of sea ranching Manila clams (Venerupis philippinarum): Successful techniques and lessons learned. Reviews in Fisheries Science 16, 44–50. https://doi.org/10.1080/10641260701790259
125. Becker, P.H., 2003. Chapter 19 Biomonitoring with birds, Trace Metals and other Contaminants in the Environment. https://doi.org/10.1016/S0927-5215(03)80149-2
126. Becker, R.H., Sultan, M.I., Boyer, G.L., Twiss, M.R., Konopko, E., 2009. Mapping cyanobacterial blooms in the Great Lakes using MODIS. Journal of Great Lakes Research 35, 447–453. https://doi.org/10.1016/j.jglr.2009.05.007
127. Begliomini, F.N., Maciel, D.C., de Almeida, S.M., Abessa, D.M., Maranho, L.A., Pereira, C.D.S., Yogui, G.T., Zanardi-Lamardo, E., Castro, Í.B., 2017. Shell alterations in limpets as putative biomarkers for multi-impacted coastal areas. Environmental Pollution 226, 494–503. https://doi.org/10.1016/j.envpol.2017.04.045
128. Behn, K., Becker, M., Burghof, S., Moseler, B.M., Willy, D.K., Alvarez, M., 2018. Using vegetation attributes to rapidly assess degradation of East African wetlands. Ecological Indicators 89, 250–259. https://doi.org/10.1016/j.ecolind.2018.02.017
129. Beisiegel, K., Darr, A., Gogina, M., Zettler, M.L., 2017. Benefits and shortcomings of non-destructive benthic imagery for monitoring hard-bottom habitats. Marine Pollution Bulletin 121, 5–15. https://doi.org/10.1016/j.marpolbul.2017.04.009
130. Beketov, M.A., Foit, K., Schäfer, R.B., Schriever, C.A., Sacchi, A., Capri, E., Biggs, J., Wells, C., Liess, M., 2009. SPEAR indicates pesticide effects in streams - Comparative use of species- and family-level biomonitoring data. Environmental Pollution 157, 1841–1848. https://doi.org/10.1016/j.envpol.2009.01.021
131. Bekkby, T., Nilsson, H.C., Olsgard, F., Rygg, B., Isachsen, P.E., Isæus, M., 2008. Identifying soft sediments at sea using GIS-modelled predictor variables and Sediment Profile Image (SPI) measured response variables. Estuarine, Coastal and Shelf Science 79, 631–636. https://doi.org/10.1016/j.ecss.2008.06.005
132. Bell, C.D., Solomon, J.L., Blumenthal, J.M., Austin, T.J., Ebanks-petrie, G., Broderick, A.C., Godley, B.J., 2007. Monitoring and conservation of critically reduced marine turtle nesting populations: Lessons from the Cayman Islands. Animal Conservation 10, 39–47. https://doi.org/10.1111/j.1469-1795.2006.00068.x
133. Bell, J.J., Burton, M., Bullimore, B., Newman, P.B., Lock, K., 2006. Morphological monitoring of subtidal sponge assemblages. Marine Ecology Progress Series 311, 79–91. https://doi.org/10.3354/meps311079
134. Bellanger, M., Levrel, H., 2017. A cost-effectiveness analysis of alternative survey methods used for the monitoring of marine recreational fishing in France. Ocean and Coastal Management 138, 19–28. https://doi.org/10.1016/j.ocecoaman.2017.01.007
135. Bellas, J., Nieto, Ó., Beiras, R., 2011. Integrative assessment of coastal pollution: Development and evaluation of sediment quality criteria from chemical contamination and ecotoxicological data. Continental Shelf Research 31, 448–456. https://doi.org/10.1016/j.csr.2010.04.012
136. Bellchambers, L.M., Evans, S.N., Meeuwig, J.J., 2013. Assessing the effectiveness of two methods of habitat characterisation for understanding species habitat relationships, using the western rock lobster (Panulirus cygnus George). Fisheries Research 139, 5–10. https://doi.org/10.1016/j.fishres.2012.09.021
137. Bellezza Quater, P., Grimaccia, F., Masini, A., 2014. Airborne unmanned monitoring system for coastal erosion assessment, in: Engineering Geology for Society and Territory - Volume 4: Marine and Coastal Processes. pp. 115–120. https://doi.org/10.1007/978-3-319-08660-6_22
138. Bellier, E., Grøtan, V., Engen, S., Schartau, A.K., Diserud, O.H., Finstad, A.G., 2012. Combining counts and incidence data: An efficient approach for estimating the log-normal species abundance distribution and diversity indices. Oecologia 170, 477–488. https://doi.org/10.1007/s00442-012-2311-2
139. Ben-Dor, E., 2002. Quantitative remote sensing of soil properties, Advances in Agronomy.
140. Benetti, D.D., Benetti, G.I., Rivera, J.A., Sardenberg, B., O’Hanlon, B., 2010. Site selection criteria for open ocean aquaculture. Marine Technology Society Journal 44, 22–35. https://doi.org/10.4031/MTSJ.44.3.11
141. Bennett, K., Wilson, S.K., Shedrawi, G., McLean, D.L., Langlois, T.J., 2016. Can diver operated stereo-video surveys for fish be used to collect meaningful data on benthic coral reef communities? Limnology and Oceanography: Methods 14, 874–885. https://doi.org/10.1002/lom3.10141
142. Berezina, N.A., Gubelit, Y.I., Polyak, Y.M., Sharov, A.N., Kudryavtseva, V.A., Lubimtsev, V.A., Petukhov, V.A., Shigaeva, T.D., 2017. An integrated approach to the assessment of the eastern Gulf of Finland health: A case study of coastal habitats. Journal of Marine Systems 171, 159–171. https://doi.org/10.1016/j.jmarsys.2016.08.013
143. Bergman, P.S., Hansen, M.J., Nate, N.A., 2011. Relationship between Electrofishing Catch Rate and Adult Trout Abundance in Wisconsin Streams. North American Journal of Fisheries Management 31, 952–961. https://doi.org/10.1080/02755947.2011.635239
144. Berkelmans, R., Hendee, J.C., Marshall, P.A., Ridd, P.V., Orpin, A.R., Irvine, D., 2002. Automatic weather stations: Tools for managing and monitoring potential impacts to coral reefs. Marine Technology Society Journal 36, 29–38. https://doi.org/10.4031/002533202787914250
145. Berman, J., Burton, M., Gibbs, R., Lock, K., Newman, P., Jones, J., Bell, J., 2013. Testing the suitability of a morphological monitoring approach for identifying temporal variability in a temperate sponge assemblage. Journal for Nature Conservation 21, 173–182. https://doi.org/10.1016/j.jnc.2012.12.003
146. Berry, O., Sarre, S.D., Farrington, L., Aitken, N., 2007. Faecal DNA detection of invasive species: The case of feral foxes in Tasmania. Wildlife Research 34, 1–7. https://doi.org/10.1071/WR06082
147. Bevilacqua, S., Claudet, J., Terlizzi, A., 2013. Best practicable aggregation of species: A step forward for species surrogacy in environmental assessment and monitoring. Ecology and Evolution 3, 3780–3793. https://doi.org/10.1002/ece3.715
148. Bevilacqua, S., Mistri, M., Terlizzi, A., Munari, C., 2018. Assessing the effectiveness of surrogates for species over time: Evidence from decadal monitoring of a Mediterranean transitional water ecosystem. Marine Pollution Bulletin 131, 507–514. https://doi.org/10.1016/j.marpolbul.2018.04.047
149. Bhat, S., Motz, L.H., Pathak, C., Kuebler, L., 2015. Geostatistics-based groundwater-level monitoring network design and its application to the Upper Floridan aquifer, USA. Environmental Monitoring and Assessment 187. https://doi.org/10.1007/s10661-014-4183-x
150. Bhattacharyya, N., Hou, A., 2013. A pentaplex PCR assay for detection and characterization of Vibrio vulnificus and Vibrio parahaemolyticus isolates. Letters in Applied Microbiology 57, 233–240. https://doi.org/10.1111/lam.12101
151. Bhugeloo, A., Peerbhay, K., Ramdhani, S., Sershen, 2018. Assessing the Trade-Offs of SPOT7 Imagery for Monitoring Natural Forest Canopy Intactness. Forests 9. https://doi.org/10.3390/f9120781
152. Bian, X., Shao, Y., Wang, S., Tian, W., Wang, X., Zhang, C., 2018. Shallow Water Depth Retrieval from Multitemporal Sentinel-1 SAR Data. IEEE Journal of Selected Topics in Applied Earth Observations and Remote Sensing 11, 2991–3000. https://doi.org/10.1109/JSTARS.2018.2851845
153. Bianchin, M., Smith, L., Beckie, R., 2015. Freeze shoe sampler for the collection of hyporheic zone sediments and porewater. Groundwater 53, 328–334. https://doi.org/10.1111/gwat.12195
154. Bidmead, P., 2014. Designed for seismic. Offshore Engineer 39, 66–67.
155. Bignert, A., Eriksson, U., Nyberg, E., Miller, A., Danielsson, S., 2014. Consequences of using pooled versus individual samples for designing environmental monitoring sampling strategies. Chemosphere 94, 177–182. https://doi.org/10.1016/j.chemosphere.2013.09.096
156. Bigongiari, N., Cipriani, L.E., Pranzini, E., Renzi, M., Vitale, G., 2015. Assessing shelf aggregate environmental compatibility and suitability for beach nourishment: A case study for Tuscany (Italy). Marine Pollution Bulletin 93, 183–193. https://doi.org/10.1016/j.marpolbul.2015.01.021
157. Bindra, N., Dubey, B., Dutta, A., 2015. Technological and life cycle assessment of organics processing odour control technologies. Science of the Total Environment 527–528, 401–412. https://doi.org/10.1016/j.scitotenv.2015.05.023
158. Bird, C., Bell, P., Sinclair, A., 2017. Filling in the white ribbon: Radar-based nearshore hydrographic monitoring. Hydro International 21, 19–21.
159. Bisack, K.D., Magnusson, G., 2014. Measuring the Economic Value of Increased Precision in Scientific Estimates of Marine Mammal Abundance and Bycatch: Harbor Porpoise Phocoena phocoena in the Northeast U.S. Gill-Net Fishery. North American Journal of Fisheries Management 34, 311–321. https://doi.org/10.1080/02755947.2013.869281
160. Bisanzio, D., Mutuku, F., LaBeaud, A.D., Mungai, P.L., Muinde, J., Busaidy, H., Mukoko, D., King, C.H., Kitron, U., 2015. Use of prospective hospital surveillance data to define spatiotemporal heterogeneity of malaria risk in coastal Kenya. Malaria Journal 14. https://doi.org/10.1186/s12936-015-1006-7
161. Blanco, J., 2005. Borehole seismic: What does it bring to the understanding of the reservoir?, Petroleum Geology Conference Proceedings. https://doi.org/10.1144/0061471
162. Blicher-Mathiesen, G., Andersen, H.E., Carstensen, J., Børgesen, C.D., Hasler, B., Windolf, J., 2014. Reprint of “Mapping of nitrogen risk areas.” Agriculture, Ecosystems and Environment 198, 83–93. https://doi.org/10.1016/j.agee.2014.06.031
163. Blomberg, A.E.A., Sæbø, T.O., Hansen, R.E., Pedersen, R.B., Austeng, A., 2017. Automatic Detection of Marine Gas Seeps Using an Interferometric Sidescan Sonar. IEEE Journal of Oceanic Engineering 42, 590–602. https://doi.org/10.1109/JOE.2016.2592559
164. Boehme, L., Lovell, P., Biuw, M., Roquet, F., Nicholson, J., Thorpe, S.E., Meredith, M.P., Fedak, M., 2009. Technical note: Animal-borne CTD-Satellite Relay Data Loggers for real-time oceanographic data collection. Ocean Science 5, 685–695. https://doi.org/10.5194/os-5-685-2009
165. Boldt, J.L., Williams, K., Rooper, C.N., Towler, R.H., Gauthier, S., 2018. Development of stereo camera methodologies to improve pelagic fish biomass estimates and inform ecosystem management in marine waters. Fisheries Research 198, 66–77. https://doi.org/10.1016/j.fishres.2017.10.013
166. Boman, E.M., De Graaf, M., Nagelkerke, L.A.J., Van Rijn, J., Schlochtern, M.M.Z., Smaal, A., 2016. UNDERWATER TOWED VIDEO: A NOVEL METHOD TO ESTIMATE DENSITIES OF QUEEN CONCH (LOBATUS GIGAS; STROMBIDAE) ACROSS ITS DEPTH RANGE. Journal of Shellfish Research 35, 493–498. https://doi.org/10.2983/035.035.0222
167. Bonachela, S., Juan, M., Casas, J.J., Fuentes-Rodríguez, F., Gallego, I., Elorrieta, M.A., 2013. Pond management and water quality for drip irrigation in Mediterranean intensive horticultural systems. Irrigation Science 31, 769–780. https://doi.org/10.1007/s00271-012-0361-1
168. Bonino, G., Burlando, M., De Gaetano, P., Solari, G., Carmisciano, C., Iafolla, L., 2015. Sea state monitoring and simulation in the “wind, ports, and sea” project, in: Towards Green Marine Technology and Transport. pp. 875–882. https://doi.org/10.1201/b18855
169. Booth, C.G., 2016. Challenge of using passive acoustic monitoring in high-energy environments: UK tidal environments and other case studies, Advances in Experimental Medicine and Biology. https://doi.org/10.1007/978-1-4939-2981-8_12
170. Borja, Á., Elliott, M., 2013. Marine monitoring during an economic crisis: The cure is worse than the disease. Marine Pollution Bulletin 68, 1–3. https://doi.org/10.1016/j.marpolbul.2013.01.041
171. Borja, A., Elliott, M., Snelgrove, P.V.R., Austen, M.C., Berg, T., Cochrane, S., Carstensen, J., Danovaro, R., Greenstreet, S., Heiskanen, A.S., Lynam, C.P., Mea, M., Newton, A., Patrício, J., Uusitalo, L., Uyarra, M.C., Wilson, C., 2016. Bridging the gap between policy and science in assessing the health status of marine ecosystems. Frontiers in Marine Science 3. https://doi.org/10.3389/fmars.2016.00175
172. Borker, A.L., Halbert, P., McKown, M.W., Tershy, B.R., Croll, D.A., 2015. A comparison of automated and traditional monitoring techniques for marbled murrelets using passive acoustic sensors. Wildlife Society Bulletin 39, 813–818. https://doi.org/10.1002/wsb.608
173. Borzo, P., 2001. Riding the tides to information integration and improved performance. Water Engineering and Management 148, 21–26.
174. Bosch, N.E., Goncalves, J.M.S., Erzini, K., Tuya, F., 2017. “How” and “what” matters: Sampling method affects biodiversity estimates of reef fishes. Ecology and Evolution 7, 4891–4906. https://doi.org/10.1002/ece3.2979
175. Boukalová, Z., Beneš, V., Veselý, L., 2011. Application of a geophysical monitoring system on the tidal and salt exposed embankments in the Humber estuary, UK. WIT Transactions on Ecology and the Environment 146, 71–82. https://doi.org/10.2495/RM110071
176. Bourlat, S.J., Borja, A., Gilbert, J., Taylor, M.I., Davies, N., Weisberg, S.B., Griffith, J.F., Lettieri, T., Field, D., Benzie, J., Glöckner, F.O., Rodríguez-Ezpeleta, N., Faith, D.P., Bean, T.P., Obst, M., 2013. Genomics in marine monitoring: New opportunities for assessing marine health status. Marine Pollution Bulletin 74, 19–31. https://doi.org/10.1016/j.marpolbul.2013.05.042
177. Bouwmeester, H., Abele, S., Manyong, V.M., Legg, C., Mwangi, M., Nakato, V., Coyne, D., Sonder, K., 2010. The potential benefits of gis techniques in disease and pest control: An example based on a regional project in central Africa, Acta Horticulturae. https://doi.org/10.17660/ActaHortic.2010.879.34
178. Bowen, A.D., Yoerger, D.R., Taylor, C., McCabe, R., Howland, J., Gomez-Ibanez, D., Kinsey, J.C., Heintz, M., McDonald, G., Peters, D., Young, C., Buescher, J., Fletcher, B., Whitcomb, L.L., Martin, S.C., Webster, S.E., Jakuba, M., 2009. The Nereus hybrid underwater robotic vehicle. Underwater Technology 28, 79–89. https://doi.org/10.3723/ut.28.079
179. Bowen, R.E., Depledge, M.H., 2006. Rapid Assessment of Marine Pollution (RAMP). Marine Pollution Bulletin 53, 631–639. https://doi.org/10.1016/j.marpolbul.2006.09.002
180. Boylen, C.W., Howe, E.A., Bartkowski, J.S., Eichler, L.W., 2004. Augmentation of a long-term monitoring program for lake george, ny by citizen volunteers. Lake and Reservoir Management 20, 121–129. https://doi.org/10.1080/07438140409354356
181. Bradshaw, C.J.A., Field, I.C., Bowman, D.M.J.S., Haynes, C., Brook, B.W., 2007. Current and future threats from non-indigenous animal species in northern Australia: A spotlight on World Heritage Area Kakadu National Park. Wildlife Research 34, 419–436. https://doi.org/10.1071/WR06056
182. Bramburger, A.J., Stephen Brown, R., Haley, J., Ridal, J.J., 2015. A new, automated rapid fluorometric method for the detection of Escherichia coli in recreational waters. Journal of Great Lakes Research 41, 298–302. https://doi.org/10.1016/j.jglr.2014.12.008
183. Branchini, S., Pensa, F., Neri, P., Tonucci, B.M., Mattielli, L., Collavo, A., Sillingardi, M.E., Piccinetti, C., Zaccanti, F., Goffredo, S., 2015. Using a citizen science program to monitor coral reef biodiversity through space and time. Biodiversity and Conservation 24, 319–336. https://doi.org/10.1007/s10531-014-0810-7
184. Brandl, S.J., Casey, J.M., Knowlton, N., Duffy, J.E., 2017. Marine dock pilings foster diverse, native cryptobenthic fish assemblages across bioregions. Ecology and Evolution 7, 7069–7079. https://doi.org/10.1002/ece3.3288
185. Braulik, G.T., Kasuga, M., Wittich, A., Kiszka, J.J., MacCaulay, J., Gillespie, D., Gordon, J., Said, S.S., Hammond, P.S., 2018. Cetacean rapid assessment: An approach to fill knowledge gaps and target conservation across large data deficient areas. Aquatic Conservation: Marine and Freshwater Ecosystems 28, 216–230. https://doi.org/10.1002/aqc.2833
186. Braun, D.C., Reynolds, J.D., Patterson, D.A., 2015. Using watershed characteristics to inform cost-effective stream temperature monitoring. Aquatic Ecology 49, 373–388. https://doi.org/10.1007/s10452-015-9531-6
187. Bresciani, M., Stroppiana, D., Odermatt, D., Morabito, G., Giardino, C., 2011. Assessing remotely sensed chlorophyll-a for the implementation of the Water Framework Directive in European perialpine lakes. Science of the Total Environment 409, 3083–3091. https://doi.org/10.1016/j.scitotenv.2011.05.001
188. Bridging the gap, 2005. . Planet Earth 11.
189. Bried, J.T., Hager, B.J., Hunt, P.D., Fox, J.N., Jensen, H.J., Vowels, K.M., 2012. Bias of reduced-effort community surveys for adult Odonata of lentic waters. Insect Conservation and Diversity 5, 213–222. https://doi.org/10.1111/j.1752-4598.2011.00156.x
190. Bright, K.D., Smith, P.M., 2002. Perceptions of new and established waterfront materials by U.S. marine decision makers. Wood and Fiber Science 34, 186–204.
191. Brigolin, D., Pranovi, F., Kholeif, S., Abdelsalam, K., Pastres, R., 2016. Interactions of cage aquaculture in Nile Delta lakes: Insights from field data and models. Regional Studies in Marine Science 7, 129–135. https://doi.org/10.1016/j.rsma.2016.06.002
192. Brischoux, F., Bonnet, X., Legagneux, P., 2009. Are sea snakes pertinent bio-indicators for coral reefs? a comparison between species and sites. Marine Biology 156, 1985–1992. https://doi.org/10.1007/s00227-009-1229-7
193. Britton, J., 2017. Ageing subsea pipelines external corrosion management, NACE - International Corrosion Conference Series.
194. Brockmann, C., Stelzer, K., 2008. Optical remote sensing of intertidal flats, in: Remote Sensing of the European Seas. pp. 117–128. https://doi.org/10.1007/978-1-4020-6772-3_9
195. Brodin, Y., Ejdung, G., Strandberg, J., Lyrholm, T., 2013. Improving environmental and biodiversity monitoring in the Baltic Sea using DNA barcoding of Chironomidae (Diptera). Molecular Ecology Resources 13, 996–1004. https://doi.org/10.1111/1755-0998.12053
196. Broszeit, S., Beaumont, N.J., Uyarra, M.C., Heiskanen, A.S., Frost, M., Somerfield, P.J., Rossberg, A.G., Teixeira, H., Austen, M.C., 2017. What can indicators of good environmental status tell us about ecosystem services?: Reducing efforts and increasing cost-effectiveness by reapplying biodiversity indicator data. Ecological Indicators 81, 409–442. https://doi.org/10.1016/j.ecolind.2017.05.057
197. Brown, A.J., Bollini, A.M., Craighead, L.W., Astin, M.C., Norrholm, S.D., Bradley, B., 2014. Self-Monitoring of Reexperiencing Symptoms: A Randomized Trial. Journal of Traumatic Stress 27, 519–525. https://doi.org/10.1002/jts.21950
198. Brown, A.M., Bejder, L., Pollock, K.H., Allen, S.J., 2016. Site-specific assessments of the abundance of three inshore dolphin species to inform conservation and management. Frontiers in Marine Science 3. https://doi.org/10.3389/fmars.2016.00004
199. Brown, C.J., Todd, B.J., Kostylev, V.E., Pickrill, R.A., 2011. Image-based classification of multibeam sonar backscatter data for objective surficial sediment mapping of Georges Bank, Canada. Continental Shelf Research 31, S110–S119. https://doi.org/10.1016/j.csr.2010.02.009
200. Brownjohn, J.M.W., Moyo, P., Omenzetter, P., Lu, Y., 2003. Assessment of highway bridge upgrading by dynamic testing and finite-element model updating. Journal of Bridge Engineering 8, 162–172. https://doi.org/10.1061/(ASCE)1084-0702(2003)8:3(162)
201. Brumovský, M., Bečanová, J., Kohoutek, J., Thomas, H., Petersen, W., Sørensen, K., Sáňka, O., Nizzetto, L., 2016. Exploring the occurrence and distribution of contaminants of emerging concern through unmanned sampling from ships of opportunity in the North Sea. Journal of Marine Systems 162, 47–56. https://doi.org/10.1016/j.jmarsys.2016.03.004
202. Bryant, I.D., 2002. Well-bore reservoir evaluation technologies to optimize field revitalization. Petroleum Geoscience 8, 339–348. https://doi.org/10.1144/petgeo.8.4.339
203. Bryhn, A.C., Jiménez, A., Mateos, A., Ríos-Insua, S., 2009. Multi-attribute analysis of trophic state and waterfowl management in Ringkøbing Fjord, Denmark. Journal of Environmental Management 90, 2568–2577. https://doi.org/10.1016/j.jenvman.2009.01.017
204. Brämick, U., Diekmann, M., Lemcke, R., Mehner, T., 2008. Assessing shifts in fish assemblages of German large lakes by literature data and commercial catch statistics. Fundamental and Applied Limnology 171, 87–103. https://doi.org/10.1127/1863-9135/2008/0171-0087
205. Buck, E.H., 2012. Marine dead zones: Understanding the problem, in: Poisoning and Acidification of the Earth’s Oceans. pp. 9–24.
206. Bui, M.P.N., Brockgreitens, J., Ahmed, S., Abbas, A., 2016. Dual detection of nitrate and mercury in water using disposable electrochemical sensors. Biosensors and Bioelectronics 85, 280–286. https://doi.org/10.1016/j.bios.2016.05.017
207. Bulgarelli, B., Djavidnia, S., 2012. On MODIS retrieval of oil spill spectral properties in the marine environment. IEEE Geoscience and Remote Sensing Letters 9, 398–402. https://doi.org/10.1109/LGRS.2011.2169647
208. Bunnell, F.L., Dunsworth, B.G., 2004. Making adaptive management for biodiversity work - The example of Weyerhaeuser in coastal British Columbia. Forestry Chronicle 80, 37–43. https://doi.org/10.5558/tfc80037-1
209. Burge, P., 2010. Condition monitoring vital for safety. Motor Ship 91, 26–27.
210. Burger, J., Gochfeld, M., 2001. On developing bioindicators for human and ecological health. Environmental Monitoring and Assessment 66, 23–46. https://doi.org/10.1023/a:1026476030728
211. Burger, J., Gochfeld, M., 2006. Locational differences in heavy metals and metalloids in Pacific Blue Mussels Mytilus [edulis] trossulus from Adak Island in the Aleutian Chain, Alaska. Science of the Total Environment 368, 937–950. https://doi.org/10.1016/j.scitotenv.2006.04.022
212. Burrows, R., Ali, K.H.M., Tickell, R.G., Hedges, T.S., Pearson, H.W., Mara, D.D., 2001. Marine-based waste stabilisation ponds: An evaluation of the hydraulic viability, Water Science and Technology.
213. Bush, S.L., Santos, G.M., Xu, X., Southon, J.R., Thiagarajan, N., Hines, S.K., Adkins, J.F., 2013. Simple, rapid, and cost effective: A screening method for 14C analysis of small carbonate samples. Radiocarbon 55, 631–640. https://doi.org/10.2458/azu_js_rc.55.16192
214. Bush, S.L., Santos, G.M., Xu, X.M., Southon, J.R., Thiagarajan, N., Hines, S.K., Adkins, J.F., 2013. SIMPLE, RAPID, AND COST EFFECTIVE: A SCREENING METHOD FOR C-14 ANALYSIS OF SMALL CARBONATE SAMPLES. Radiocarbon 55, 631–640. https://doi.org/10.1017/s0033822200057787
215. Buss, D.F., Borges, E.L., 2008. Application of rapid bioassessment protocols (RBP) for benthic macroinvertebrates in Brazil: Comparison between sampling techniques and mesh sizes. Neotropical Entomology 37, 288–295. https://doi.org/10.1590/S1519-566X2008000300007
216. Bustos, E., Báez, W.A., Norini, G., Chiodi, A.L., Groppell, G., Arnosio, J.M., 2017. Using optical imagery data for lithological mapping of composite volcanoes in high arid puna plateau. Tuzgle volcano case study. Revista de la Asociacion Geologica Argentina 74, 357–371.
217. Buzzelli, C.P., Ramus, J., Paerl, H.W., 2003. Ferry-based monitoring of surface water quality in North Carolina estuaries. Estuaries 26, 975–984. https://doi.org/10.1007/BF02803356
218. Byer, J.D., Struger, J., Sverko, E., Klawunn, P., Todd, A., 2011. Spatial and seasonal variations in atrazine and metolachlor surface water concentrations in Ontario (Canada) using ELISA. Chemosphere 82, 1155–1160. https://doi.org/10.1016/j.chemosphere.2010.12.054
219. Bürkli, A., Sieber, N., Seppälä, K., Jokela, J., 2017. Comparing direct and indirect selfing rate estimates: when are population-structure estimates reliable? Heredity 118, 525–533. https://doi.org/10.1038/hdy.2017.1
220. Bäck, S., Ekebom, J., Kangas, P., 2002. A proposal for a long-term baseline phytobenthos monitoring programme for the finnish baltic coastal waters: Monitoring submerged rocky shore vegetation. Environmental Monitoring and Assessment 79, 13–27. https://doi.org/10.1023/A:1020095220957
221. Cabral-Oliveira, J., Bevilacqua, S., Terlizzi, A., Pardal, M.A., 2014. Are eulittoral assemblages suitable for detecting the effects of sewage discharges in Atlantic and Mediterranean coastal areas? Italian Journal of Zoology 81, 584–592. https://doi.org/10.1080/11250003.2014.947336
222. Cachon, T., Frykman, O., Innes, J.F., Lascelles, B.D.X., Okumura, M., Sousa, P., Staffieri, F., Steagall, P.V., Van Ryssen, B., Group, C.D., 2018. Face validity of a proposed tool for staging canine osteoarthritis: Canine OsteoArthritis Staging Tool (COAST). Veterinary Journal 235, 1–8. https://doi.org/10.1016/j.tvjl.2018.02.017
223. Caffrey, J.M., Murrell, M.C., Amacker, K.S., Harper, J.W., Phipps, S., Woodrey, M.S., 2014. Seasonal and Inter-annual Patterns in Primary Production, Respiration, and Net Ecosystem Metabolism in Three Estuaries in the Northeast Gulf of Mexico. Estuaries and Coasts 37, 222–241. https://doi.org/10.1007/s12237-013-9701-5
224. Cahalane, C., Hanafin, J., Monteys, X., 2017. Improving satellite-derived bathymetry. Hydro International 21, 16–19.
225. Cai, H., Sun, Y., 2007. Management of marine cage aquaculture: Environmental carrying capacity method based on dry feed conversion rate. Environmental Science and Pollution Research 14, 463–469. https://doi.org/10.1065/espr2007.05.423
226. Cain, A., Morgan, J.T., Brooks, N., 2011. Mercury policy in the Great Lakes states: Past successes and future opportunities. Ecotoxicology 20, 1500–1511. https://doi.org/10.1007/s10646-011-0764-4
227. Calambokidis, J., Barlow, J., 2004. Abundance of blue and humpback whales in the eastern north pacific estimated by capture-recapture and line-transect methods. Marine Mammal Science 20, 63–85. https://doi.org/10.1111/j.1748-7692.2004.tb01141.x
228. Calderbank, B., 2002. Minimising offshore survey product liability. Hydro International 6, 22–25.
229. Calgua, B., Fumian, T., Rusiñol, M., Rodriguez-Manzano, J., Mbayed, V.A., Bofill-Mas, S., Miagostovich, M., Girones, R., 2013. Detection and quantification of classic and emerging viruses by skimmed-milk flocculation and PCR in river water from two geographical areas. Water Research 47, 2797–2810. https://doi.org/10.1016/j.watres.2013.02.043
230. Call, K.A., Hardy, J.T., Wallin, D.O., 2003. Coral reef habitat discrimination using multivariate spectral analysis and satellite remote sensing. International Journal of Remote Sensing 24, 2627–2639. https://doi.org/10.1080/0143116031000066990
231. Camilleri, S., De Giglio, M., Stecchi, F., Pérez-Hurtado, A., 2017. Land use and land cover change analysis in predominantly man-made coastal wetlands: towards a methodological framework. Wetlands Ecology and Management 25, 23–43. https://doi.org/10.1007/s11273-016-9500-4
232. Camino-Sánchez, F.J., Zafra-Gómez, A., Oliver-Rodríguez, B., Ruiz-Naranjo, I., Ruiz-García, J., Vílchez, J.L., 2012. Validation of a method for the determination of tributyltin in seawater by stir bar sorptive extraction-liquid chromatography tandem mass spectrometry. Journal of Chromatography A 1263, 14–20. https://doi.org/10.1016/j.chroma.2012.09.018
233. Campbell, H.A., Beyer, H.L., Dennis, T.E., Dwyer, R.G., Forester, J.D., Fukuda, Y., Lynch, C., Hindell, M.A., Menke, N., Morales, J.M., Richardson, C., Rodgers, E., Taylor, G., Watts, M.E., Westcott, D.A., 2015. Finding our way: On the sharing and reuse of animal telemetry data in Australasia. Science of the Total Environment 534, 79–84. https://doi.org/10.1016/j.scitotenv.2015.01.089
234. Campbell, M.L., Gould, B., Hewitt, C.L., 2007. Survey evaluations to assess marine bioinvasions. Marine Pollution Bulletin 55, 360–378. https://doi.org/10.1016/j.marpolbul.2007.01.015
235. Canal-Vergés, P., Petersen, J.K., Rasmussen, E.K., Erichsen, A., Flindt, M.R., 2016. Validating GIS tool to assess eelgrass potential recovery in the Limfjorden (Denmark). Ecological Modelling 338, 135–148. https://doi.org/10.1016/j.ecolmodel.2016.04.023
236. Canedo-Arguelles, M., Boix, D., Sanchez-Millaruelo, N., Sala, J., Caiola, N., Nebra, A., Rieradevall, M., 2012. A rapid bioassessment tool for the evaluation of the water quality of transitional waters. Estuarine Coastal and Shelf Science 111, 129–138. https://doi.org/10.1016/j.ecss.2012.07.001
237. Canfield, D.E., Jr., Brown, C.D., Bachmann, R.W., Hoyer, M.V., 2002. Volunteer lake monitoring: Testing the reliability of data collected by the Florida LAKEWATCH Program. Lake and Reservoir Management 18, 1–9. https://doi.org/10.1080/07438140209353924
238. Cao, B., Zhao, J., Yang, P., Lv, Z., Liu, X., Min, G., 2018. 3-d multiobjective deployment of an industrial wireless sensor network for maritime applications utilizing a distributed parallel algorithm. IEEE Transactions on Industrial Informatics 14, 5487–5495. https://doi.org/10.1109/TII.2018.2803758
239. Capela, R., Raimundo, J., Santos, M.M., Caetano, M., Micaelo, C., Vale, C., Guimarães, L., Reis-Henriques, M.A., 2016. The use of biomarkers as integrative tools for transitional water bodies monitoring in the Water Framework Directive context - A holistic approach in Minho river transitional waters. Science of the Total Environment 539, 85–96. https://doi.org/10.1016/j.scitotenv.2015.08.113
240. Capelle, P., Matthews, P., 2008. “Intelligent infill” for cost effective 3D seismic marine acquisitions, SEG Technical Program Expanded Abstracts. https://doi.org/10.1190/1.3054774
241. Caporal, M., Blacquière, G., Davydenko, M., 2018. Broadband imaging via direct inversion of blended dispersed source array data. Geophysical Prospecting 66, 942–953. https://doi.org/10.1111/1365-2478.12584
242. Caramanna, G., Voltattorni, N., Mercedes Maroto-Valer, M., 2011. Is Panarea Island (Italy) a valid and cost-effective natural laboratory for the development of detection and monitoring techniques for submarine CO 2 seepage? Greenhouse Gases: Science and Technology 1, 200–210. https://doi.org/10.1002/ghg.28
243. Carballeira, C., De Orte, M.R., Viana, I.G., Carballeira, A., 2012. Implementation of a minimal set of biological tests to assess the ecotoxic effects of effluents from land-based marine fish farms. Ecotoxicology and Environmental Safety 78, 148–161. https://doi.org/10.1016/j.ecoenv.2011.11.022
244. Carew, M.E., Kellar, C.R., Pettigrove, V.J., Hoffmann, A.A., 2018. Can high-throughput sequencing detect macroinvertebrate diversity for routine monitoring of an urban river? Ecological Indicators 85, 440–450. https://doi.org/10.1016/j.ecolind.2017.11.002
245. Carew, M.E., Nichols, S.J., Batovska, J., St Clair, R., Murphy, N.P., Blacket, M.J., Shackleton, M.E., 2017. A DNA barcode database of Australia’s freshwater macroinvertebrate fauna. Marine and Freshwater Research 68, 1788–1802. https://doi.org/10.1071/MF16304
246. Carletti, A., De Leo, G.A., Ferrari, I., 2006. A preliminary coastal wetland assessment procedure: Designing and testing an environmental sustainability index for Mediterranean lagoons. Chemistry and Ecology 22, S15–S35. https://doi.org/10.1080/02757540600572578
247. Carniel, S., Beldowski, J., Cumming, A., 2017. Review&Forecast: Munitions in the sea: Time for global action. Sea Technology 58, 37–39.
248. Caroni, R., Irvine, K., 2010. The potential of zooplankton communities for ecological assessment of lakes: Redundant concept or political oversight? Biology and Environment 110, 35–53. https://doi.org/10.3318/BIOE.2010.110.1.35
249. Carpio, J.N., Nalunat, J.C., Bañares, J.C.T., Fernando, A.R., Jurado, J.A.O., Bayang, S.J.N., Dizon, C.F., Valerio, C.M.G., Marasigan, J.S., 2018. A cost-effective fish pond monitoring and warning system using thermal probe. International Journal of Simulation: Systems, Science and Technology 19, 14.1-14.4. https://doi.org/10.5013/IJSSST.a.19.03.14
250. Carstensen, J., Dahl, K., Henriksen, P., Hjorth, M., Josefson, A., Krause-Jensen, D., 2012. Coastal Monitoring Programs, in: Treatise on Estuarine and Coastal Science. pp. 175–206. https://doi.org/10.1016/B978-0-12-374711-2.00712-9
251. Caruso, G., De Pasquale, F., Mita, D.G., Micale, V., 2016. Digestive enzymatic patterns as possible biomarkers of endocrine disruption in the red mullet (Mullus barbatus): A preliminary investigation. Marine Pollution Bulletin 105, 37–42. https://doi.org/10.1016/j.marpolbul.2016.03.005
252. Carvalho, L., Miller, C.A., Scott, E.M., Codd, G.A., Davies, P.S., Tyler, A.N., 2011. Cyanobacterial blooms: Statistical models describing risk factors for national-scale lake assessment and lake management. Science of the Total Environment 409, 5353–5358. https://doi.org/10.1016/j.scitotenv.2011.09.030
253. Casoli, E., Ventura, D., Cutroneo, L., Capello, M., Jona-Lasinio, G., Rinaldi, R., Criscoli, A., Belluscio, A., Ardizzone, G.D., 2017. Assessment of the impact of salvaging the Costa Concordia wreck on the deep coralligenous habitats. Ecological Indicators 80, 124–134. https://doi.org/10.1016/j.ecolind.2017.04.058
254. Castellote, M., Brotons, J.M., Chicote, C., Gazo, M., Cerdà, M., 2015. Long-term acoustic monitoring of bottlenose dolphins, Tursiops truncatus, in marine protected areas in the Spanish Mediterranean Sea. Ocean and Coastal Management 113, 54–66. https://doi.org/10.1016/j.ocecoaman.2015.05.017
255. Castendyk, D.N., Balistrieri, L.S., Gammons, C., Tucci, N., 2015. Modeling and management of pit lake water chemistry 2: Case studies. Applied Geochemistry 57, 289–307. https://doi.org/10.1016/j.apgeochem.2014.09.003
256. Catherine, A., Selma, M., Mouillot, D., Troussellier, M., Bernard, C., 2016. Patterns and multi-scale drivers of phytoplankton species richness in temperate peri-urban lakes. Science of the Total Environment 559, 74–83. https://doi.org/10.1016/j.scitotenv.2016.03.179
257. Cavallo, M., Torras, X., Mascaró, O., Ballesteros, E., 2016. Effect of temporal and spatial variability on the classification of the Ecological Quality Status using the CARLIT Index. Marine Pollution Bulletin 102, 122–127. https://doi.org/10.1016/j.marpolbul.2015.11.047
258. Cecchi, E., Gennaro, P., Piazzi, L., Ricevuto, E., Serena, F., 2014. Development of a new biotic index for ecological status assessment of Italian coastal waters based on coralligenous macroalgal assemblages. European Journal of Phycology 49, 298–312. https://doi.org/10.1080/09670262.2014.918657
259. Cefali, M.E., Ballesteros, E., Riera, J.L., Chappuis, E., Terradas, M., Mariani, S., Cebrian, E., 2018. The optimal sampling design for littoral habitats modelling: A case study from the north-western Mediterranean. PLoS ONE 13. https://doi.org/10.1371/journal.pone.0197234
260. Cerup-Simonsen, B., De Kat, J.O., Jakobsen, O.G., Pedersen, L.R., Petersen, J.B., Posborg, T., 2010. An integrated approach towards cost-effective operation of ships with reduced GHG emissions. Transactions - Society of Naval Architects and Marine Engineers 118, 381–396.
261. Cha, Y.H., Jo, C.H., Suh, J.H., 2003. Water bottom seismic refraction survey for engineering applications. Geosystem Engineering 6, 40–45. https://doi.org/10.1080/12269328.2003.10541203
262. Chai, F., Wang, C.A., Wang, T.T., Li, L., Su, Z.M., 2010. Colorimetric Detection of Pb2+ Using Glutathione Functionalized Gold Nanoparticles. Acs Applied Materials & Interfaces 2, 1466–1470. https://doi.org/10.1021/am100107k
263. Chaitanya, A.V.S., Lengaigne, M., Vialard, J., Gopalakrishna, V.V., Durand, F., Kranthikumar, C., Amritash, S., Suneel, V., Papa, F., Ravichandran, M., 2014. SALINITY MEASUREMENTS COLLECTED BY FISHERMEN REVEAL A “RIVER IN THE SEA” FLOWING ALONG THE EASTERN COAST OF INDIA. Bulletin of the American Meteorological Society 95, 1897-+. https://doi.org/10.1175/bams-d-12-00243.1
264. Chambault, P., Vandeperre, F., Machete, M., Lagoa, J.C., Pham, C.K., 2018. Distribution and composition of floating macro litter off the Azores archipelago and Madeira (NE Atlantic) using opportunistic surveys. Marine Environmental Research 141, 225–232. https://doi.org/10.1016/j.marenvres.2018.09.015
265. Chan, J.K.Y., Man, Y.B., Xing, G.H., Wu, S.C., Murphy, M.B., Xu, Y., Wong, M.H., 2013. Dietary exposure to polychlorinated dibenzo-p-dioxins and dibenzofurans via fish consumption and dioxin-like activity in fish determined by H4IIE-luc bioassay. Science of the Total Environment 463–464, 1192–1200. https://doi.org/10.1016/j.scitotenv.2012.07.099
266. Chance, T.S., Northcutt, J.G., 2000. The huggin 3000 AUV. Sea Technology 41, 10–14.
267. Chang, N.B., Yeh, S.C., Chang, C.H., 2011. Optimal expansion of a coastal wastewater treatment and ocean outfall system under uncertainty (II): optimisation analysis. Civil Engineering and Environmental Systems 28, 39–59. https://doi.org/10.1080/10286600903243138
268. Chang, S.J., 2001. Design and preliminary test on the integration of weather data in ECDIS for marine navigation. Journal of Marine Science and Technology 9, 21–24.
269. Chang, Y.H., Scrimshaw, M.D., Lester, J.N., 2004. Quantifying uncertainties in the assessment of sediment quality: Statistical criteria and guidelines for sediment quality assessments. Environmental Technology 25, 247–259. https://doi.org/10.1080/09593330409355458
270. Chaparro, M.A.E., Nunez, H., Lirio, J.M., Gogorza, C.S.G., Sinito, A.M., 2007. Magnetic screening and heavy metal pollution studies in soils from Marambio Station, Antarctica. Antarctic Science 19, 379–393. https://doi.org/10.1017/s0954102007000454
271. Charles, E., Boude, J.P., Murray, A., Paquotte, P., 2003. Coastal fishing: Resource’s enhancement and preservation. Ocean and Coastal Management 46, 421–437. https://doi.org/10.1016/S0964-5691(03)00016-4
272. Chaussard, E., Kerosky, S., 2016. Characterization of black sand mining activities and their environmental impacts in the philippines using remote sensing. Remote Sensing 8. https://doi.org/10.3390/rs8020100
273. Chavan, P.V., Dennett, K.E., Marchand, E.A., Gustin, M.S., 2007. Evaluation of small-scale constructed wetland for water quality and Hg transformation. Journal of Hazardous Materials 149, 543–547. https://doi.org/10.1016/j.jhazmat.2007.06.077
274. Cheaitani, A., Laurila, T., 2012. Latest trends in corrosion control for new reinforced concrete structures and a new concept for intelligent structures, NACE - International Corrosion Conference Series.
275. Chebud, Y., Naja, G.M., Rivero, R.G., Melesse, A.M., 2012. Water Quality Monitoring Using Remote Sensing and an Artificial Neural Network. Water Air and Soil Pollution 223, 4875–4887. https://doi.org/10.1007/s11270-012-1243-0
276. Chen, J., Mao, Z., Zhang, H., Wu, J., Chen, X., Pan, D., 2007. Analysis on coral reefs mapping using SPOT5 data at the Dongsha Atoll. Acta Oceanologica Sinica 26, 26–35.
277. Chen, W.Y., Jou, L.J., Chen, S.H., Liao, C.M., 2012. A real-time biomonitoring system to detect arsenic toxicity by valve movement in freshwater clam Corbicula fluminea. Ecotoxicology 21, 1177–1187. https://doi.org/10.1007/s10646-012-0872-9
278. Chen, Y., Gillieson, D., 2009. Evaluation of Landsat TM vegetation indices for estimating vegetation cover on semi-arid rangelands: A case study from Australia. Canadian Journal of Remote Sensing 35, 435–446. https://doi.org/10.5589/m09-037
279. Chen, Y., Zhang, L., Xu, C., Vaidyanathan, S., 2016. Dissolved inorganic carbon speciation in aquatic environments and its application to monitor algal carbon uptake. Science of the Total Environment 541, 1282–1295. https://doi.org/10.1016/j.scitotenv.2015.10.025
280. Cheng, C., Chen, H.Y., Wu, C.S., Meena, J.S., Simon, T., Ko, F.H., 2016. A highly sensitive and selective cyanide detection using a gold nanoparticle-based dual fluorescence-colorimetric sensor with a wide concentration range. Sensors and Actuators, B: Chemical 227, 283–290. https://doi.org/10.1016/j.snb.2015.12.057
281. Cheng, Y.W., Hillier, L.K., 2011. Use of pacific oyster Crassostrea gigas (Thunberg, 1793) shell to collect wild Juvenile sea cucumber Parastichopus californicus (Stimpson, 1857). Journal of Shellfish Research 30, 65–69. https://doi.org/10.2983/035.030.0110
282. Chesshyre, M., 2009. Deliberating on decom. Offshore Engineer 34.
283. Chial, B., Persoone, G., 2002. Cyst-based toxicity tests XIII - Development of a short chronic sediment toxicity test with the ostracod crustacean Heterocypris incongruens: Methodology and precision. Environmental Toxicology 17, 528–532. https://doi.org/10.1002/tox.10086
284. Chial, B., Persoone, G., 2002. Cyst-based toxicity tests XIII - Development of a short chronic sediment toxicity test with the ostracod crustacean Heterocypris incongruents: Methodology and precision. Environmental Toxicology 17, 528–532. https://doi.org/10.1002/tox.10086
285. Chion, C., Turgeon, S., Cantin, G., Michaud, R., Menard, N., Lesage, V., Parrott, L., Beaufils, P., Clermont, Y., Gravel, C., 2018. A voluntary conservation agreement reduces the risks of lethal collisions between ships and whales in the St. Lawrence Estuary (Quebec, Canada): From co-construction to monitoring compliance and assessing effectiveness. Plos One 13. https://doi.org/10.1371/journal.pone.0202560
286. Chion, C., Turgeon, S., Cantin, G., Michaud, R., Ménard, N., Lesage, V., Parrott, L., Beaufils, P., Clermont, Y., Gravel, C., 2018. A voluntary conservation agreement reduces the risks of lethal collisions between ships and whales in the St. Lawrence Estuary (Québec, Canada): From co-construction to monitoring compliance and assessing effectiveness. PLoS ONE 13. https://doi.org/10.1371/journal.pone.0202560
287. Chirayath, V., Earle, S.A., 2016. Drones that see through waves – preliminary results from airborne fluid lensing for centimetre-scale aquatic conservation. Aquatic Conservation: Marine and Freshwater Ecosystems 26, 237–250. https://doi.org/10.1002/aqc.2654
288. Chittenden, C.M., Fauchald, P., Rikardsen, A.H., 2013. Important open-ocean areas for northern Atlantic salmon (Salmo salar) - as estimated using a simple ambient-temperature approach. Canadian Journal of Fisheries and Aquatic Sciences 70, 101–104. https://doi.org/10.1139/cjfas-2012-0215
289. Chiu, J.M.Y., Degger, N., Leung, J.Y.S., Po, B.H.K., Zheng, G.J., Richardson, B.J., Lau, T.C., Wu, R.S.S., 2016. A novel approach for estimating the removal efficiencies of endocrine disrupting chemicals and heavy metals in wastewater treatment processes. Marine Pollution Bulletin 112, 53–57. https://doi.org/10.1016/j.marpolbul.2016.08.043
290. Choi, J.Y., Hong, G.H., Ra, K., Kim, K.T., Kim, K., 2014. Magnetic characteristics of sediment grains concurrently contaminated with TBT and metals near a shipyard in Busan, Korea. Marine Pollution Bulletin 85, 679–685. https://doi.org/10.1016/j.marpolbul.2014.03.029
291. Chow, J., 2015. Spatially Explicit Evaluation of Local Extractive Benefits from Mangrove Plantations in Bangladesh. Journal of Sustainable Forestry 34, 651–681. https://doi.org/10.1080/10549811.2015.1036454
292. Chowdhury, O.R., Kim, H.G., Lee, M., Shin, C., Cho, Y., Park, J., 2016. Novel wave energy harvesting system for ocean sensor network applications. International Journal of Control and Automation 9, 93–102. https://doi.org/10.14257/ijca.2016.9.2.10
293. Christie, K.S., Gilbert, S.L., Brown, C.L., Hatfield, M., Hanson, L., 2016. Unmanned aircraft systems in wildlife research: current and future applications of a transformative technology. Frontiers in Ecology and the Environment 14, 242–252. https://doi.org/10.1002/fee.1281
294. Church, R.A., Warren, D.J., 2002. Autonomous underwater vehicles: The latest tool for archaeological investigations. Marine Technology Society Journal 36, 45–50. https://doi.org/10.4031/002533202787913431
295. Cid, A., Prado, R., Rioboo, C., Suárez-Bregua, P., Herrero, C., 2013. Use of microalgae as biological indicators of pollution: Looking for new relevant cytotoxicity endpoints, in: Microalgae: Biotechnology, Microbiology and Energy. pp. 311–324.
296. Cigna, F., Banks, V.J., Donald, A.W., Donohue, S., Graham, C., Hughes, D., McKinley, J.M., Parker, K., 2017. Mapping ground instability in areas of geotechnical infrastructure using satellite InSAR and small UAV surveying: A case study in Northern Ireland. Geosciences (Switzerland) 7. https://doi.org/10.3390/geosciences7030051
297. Claire, K., Nathalie, C.M., Noelle, B., Frank, D., 2019. Optimizing cost-efficiency of long term monitoring programs by using for spatially balanced sampling designs: The case of manila clams in Arcachon bay. Ecological Informatics 49, 32–39. https://doi.org/10.1016/j.ecoinf.2018.11.005
298. Claire, K., Nathalie, C.M., Noëlle, B., Frank, D., 2019. Optimizing cost-efficiency of long term monitoring programs by using spatially balanced sampling designs: The case of manila clams in Arcachon bay. Ecological Informatics 49, 32–39. https://doi.org/10.1016/j.ecoinf.2018.11.005
299. Clarkson, C.E., 2011. Applicability of ptilochronology as a conservation tool in waterbird studies. Ecological Indicators 11, 1707–1709. https://doi.org/10.1016/j.ecolind.2011.04.019
300. Claudet, J., García-Charton, J.A., Lenfant, P., 2011. Combined effects of levels of protection and environmental variables at different spatial resolutions on fish assemblages in a marine protected area. Conservation Biology 25, 105–114. https://doi.org/10.1111/j.1523-1739.2010.01586.x
301. Clayton, L., Dennison, G., 2017. Inexpensive video drop-camera for surveying sensitive benthic habitats: Applications from glass sponge (Hexactinellida) reefs in Howe Sound, British Columbia. Canadian Field-Naturalist 131, 46–54. https://doi.org/10.22621/cfn.v131i1.1783
302. Clemento, A.J., Crandall, E.D., Garza, J.C., 2014. Evaluation of a single nucleotide polymorphism baseline for genetic stock identification of Chinook Salmon (Oncorhynchus tshawytscha) in the California Current large marine ecosystem. Fishery Bulletin 112, 112–130. https://doi.org/10.7755/fb.112.2-3.2
303. Clua, E., Brena, P.F., Lecasble, C., Ghnassia, R., Chauvet, C., 2011. Prevalence and proposal for cost-effective management of the ciguatera risk in the Noumea fish market, New Caledonia (South Pacific). Toxicon 58, 591–601. https://doi.org/10.1016/j.toxicon.2011.08.020
304. Codiga, D.L., 2015. A Marine Autonomous Surface Craft for Long-Duration, Spatially Explicit, Multidisciplinary Water Column Sampling in Coastal and Estuarine Systems. Journal of Atmospheric and Oceanic Technology 32, 627–641. https://doi.org/10.1175/jtech-d-14-00171.1
305. Colefax, A.P., Butcher, P.A., Kelaher, B.P., 2018. The potential for unmanned aerial vehicles (UAVs) to conduct marine fauna surveys in place of manned aircraft. ICES Journal of Marine Science 75, 1–8. https://doi.org/10.1093/icesjms/fsx100
306. Coleman, A.M., Diefenderfer, H.L., Ward, D.L., Borde, A.B., 2015. A spatially based area-time inundation index model developed to assess habitat opportunity in tidal-fluvial wetlands and restoration sites. Ecological Engineering 82, 624–642. https://doi.org/10.1016/j.ecoleng.2015.05.006
307. Colenutt, A., Mason, T., Cocuccio, A., Kinnear, R., Parker, D., 2013. Nearshore substrate and marine habitat mapping to inform marine policy and coastal management. Journal of Coastal Research 1509–1514. https://doi.org/10.2112/SI65-255
308. Collas, F.P.L., Breedveld, S.K.D., Matthews, J., van der Velde, G., Leuven, R.S.E.W., 2017. Invasion biology and risk assessment of the recently introduced chinese mystery snail, Bellamya (Cipangopaludina) chinensis (Gray, 1834), in the rhine and meuse river basins in Western Europe. Aquatic Invasions 12, 275–286. https://doi.org/10.3391/ai.2017.12.3.02
309. Collin, A., Hench, J.L., Pastol, Y., Planes, S., Thiault, L., Schmitt, R.J., Holbrook, S.J., Davies, N., Troyer, M., 2018. High resolution topobathymetry using a Pleiades-1 triplet: Moorea Island in 3D. Remote Sensing of Environment 208, 109–119. https://doi.org/10.1016/j.rse.2018.02.015
310. Collins, S.F., Diana, M.J., Butler, S.E., Wahl, D.H., 2017. A Comparison of Sampling Gears for Capturing Juvenile Silver Carp in River-Floodplain Ecosystems. North American Journal of Fisheries Management 37, 94–100. https://doi.org/10.1080/02755947.2016.1240121
311. Colón-Cruz, L., Kristofco, L., Crooke-Rosado, J., Acevedo, A., Torrado, A., Brooks, B.W., Sosa, M.A., Behra, M., 2018. Alterations of larval photo-dependent swimming responses (PDR): New endpoints for rapid and diagnostic screening of aquatic contamination. Ecotoxicology and Environmental Safety 147, 670–680. https://doi.org/10.1016/j.ecoenv.2017.09.018
312. Cominelli, S., Moulins, A., Rosso, M., Tepsich, P., 2016. Fin whale seasonal trends in the Pelagos Sanctuary, Mediterranean Sea. Journal of Wildlife Management 80, 490–499. https://doi.org/10.1002/jwmg.1027
313. Connor, L., Matson, R., Kelly, F.L., 2017. Length-weight relationships for common freshwater fish species in irish lakes and rivers. Biology and Environment 117B. https://doi.org/10.3318/BIOE.2017.07
314. Constable, A.J., 2011. Lessons from CCAMLR on the implementation of the ecosystem approach to managing fisheries. Fish and Fisheries 12, 138–151. https://doi.org/10.1111/j.1467-2979.2011.00410.x
315. Conte, G., Scaradozzi, D., Mannocchi, D., Raspa, P., Panebianco, L., Screpanti, L., 2018. Development and Experimental Tests of a ROS Multi-agent Structure for Autonomous Surface Vehicles. Journal of Intelligent and Robotic Systems: Theory and Applications 92, 705–718. https://doi.org/10.1007/s10846-017-0700-9
316. Cooke, S.J., Schreer, J.F., 2002. Determination of fish community composition in the untempered regions of a thermal effluent canal-the efficacy of a fixed underwater videography system. Environmental Monitoring and Assessment 73, 109–129. https://doi.org/10.1023/A:1013099430900
317. Cooper, B.A., Raphael, M.G., Mack, D.E., 2001. Radar-based monitoring of marbled murrelets. Condor 103, 219–229. https://doi.org/10.1650/0010-5422(2001)103[0219:rbmomm]2.0.co;2
318. Cooper, D.A., Ekström, M., 2005. Applicability of the PEMS technique for simplified NO X monitoring on board ships. Atmospheric Environment 39, 127–137. https://doi.org/10.1016/j.atmosenv.2004.09.019
319. Cooper, H., McMurray, A., Ward, L., Connor, M., 2015. Implementing patient-centred care in the context of an integrated care program. International Journal of Care Coordination 18, 72–77. https://doi.org/10.1177/2053434516639336
320. Cooper, K.M., 2013. Setting limits for acceptable change in sediment particle size composition: Testing a new approach to managing marine aggregate dredging. Marine Pollution Bulletin 73, 86–97. https://doi.org/10.1016/j.marpolbul.2013.05.034
321. Corbi, H., Riquelme, A., Megias-Banos, C., Abellan, A., 2018. 3-D Morphological Change Analysis of a Beach with Seagrass Berm Using a Terrestrial Laser Scanner. Isprs International Journal of Geo-Information 7. https://doi.org/10.3390/ijgi7070234
322. Corfield, S.J., Young, J.M., 2006. Unmanned surface vehicles - game changing technology for naval operations, in: Advances in Unmanned Marine Vehicles. pp. 311–328. https://doi.org/10.1049/PBCE069E_ch15
323. Correia, A.M., Tepsich, P., Rosso, M., Caldeira, R., Sousa-Pinto, I., 2015. Cetacean occurrence and spatial distribution: Habitat modelling for offshore waters in the Portuguese EEZ (NE Atlantic). Journal of Marine Systems 143, 73–85. https://doi.org/10.1016/j.jmarsys.2014.10.016
324. Corrosion 2013 Expo: Savcor Group, Ltd, 2013. . Materials Performance 52, 157.
325. Corrosion risk assessment and planned maintenance for corrosion control of onshore and offshore field in Adriatic sea, 2000. . Metallurgia Italiana 92, 27.
326. Costa, B.M., Battista, T.A., Pittman, S.J., 2009. Comparative evaluation of airborne LiDAR and ship-based multibeam SoNAR bathymetry and intensity for mapping coral reef ecosystems. Remote Sensing of Environment 113, 1082–1100. https://doi.org/10.1016/j.rse.2009.01.015
327. Costa, P.R., Costa, S.T., Braga, A.C., Rodrigues, S.M., Vale, P., 2017. Relevance and challenges in monitoring marine biotoxins in non-bivalve vectors. Food Control 76, 24–33. https://doi.org/10.1016/j.foodcont.2016.12.038
328. Costa-Dias, S., Sousa, R., Antunes, C., 2010. Ecological quality assessment of the lower Lima Estuary. Marine Pollution Bulletin 61, 234–239. https://doi.org/10.1016/j.marpolbul.2010.02.019
329. Côté, I.M., Gill, J.A., Gardner, T.A., Watkinson, A.R., 2005. Measuring coral reef decline through meta-analyses. Philosophical Transactions of the Royal Society B: Biological Sciences 360, 385–395. https://doi.org/10.1098/rstb.2004.1591
330. Cottle, D., Eckard, R., Bray, S., Sullivan, M., 2016. An evaluation of carbon offset supplementation options for beef production systems on coastal speargrass in central Queensland, Australia. Animal Production Science 56, 385–392. https://doi.org/10.1071/AN15446
331. Courtemanche, D.A., Whoriskey Jr, F.G., Bujold, V., Curry, R.A., 2005. A nonlethal approach using strontium in scales to distinguish periods of marine and freshwater residency of anadromous species. Canadian Journal of Fisheries and Aquatic Sciences 62, 2443–2449. https://doi.org/10.1139/f05-162
332. Cousins, L.J., Cousins, M.S., Gardiner, T., Underwood, G.J.C., 2017. Factors influencing the initial establishment of salt marsh vegetation on engineered sea wall terraces in south east England. Ocean & Coastal Management 143, 96–104. https://doi.org/10.1016/j.ocecoaman.2016.11.010
333. Cragg, J.L., Burger, A.E., Piatt, J.F., 2015. Testing the effectiveness of automated acoustic sensors for monitoring vocal activity of marbled murrelets Brachyramphus marmoratus. Marine Ornithology 43, 151–160.
334. Cressey, D., 2011. Ocean conservation: Uncertain sanctuary. Nature 480, 166–167. https://doi.org/10.1038/480166a
335. Cresswell, A.J., Sanderson, D.C.W., 2012. Evaluating airborne and ground based gamma spectrometry methods for detecting particulate radioactivity in the environment: A case study of Irish Sea beaches. Science of the Total Environment 437, 285–296. https://doi.org/10.1016/j.scitotenv.2012.08.064
336. Cristina, S., Icely, J., Costa Goela, P., Angel DelValls, T., Newton, A., 2015. Using remote sensing as a support to the implementation of the European Marine Strategy Framework Directive in SW Portugal. Continental Shelf Research 108, 169–177. https://doi.org/10.1016/j.csr.2015.03.011
337. Cronin, M.F., Meinig, C., Sabine, C.L., Ichikawa, H., Tomita, H., 2008. Surface Mooring Network in the Kuroshio Extension. Ieee Systems Journal 2, 424–430. https://doi.org/10.1109/jsyst.2008.925982
338. Crowther, J., Kay, D., Wyer, M.D., 2001. Relationships between microbial water quality and environmental conditions in coastal recreational waters: The Fylde coast, UK. Water Research 35, 4029–4038. https://doi.org/10.1016/S0043-1354(01)00123-3
339. Cui, W., Wang, F., Pan, B., Hu, Y., Du, Q., 2015. Issues to be solved in the design, manufacture and maintenance of a full ocean depth manned cabin, in: Advances in Engineering Research. pp. 1–29.
340. Culver, C.S., Schroeter, S.C., Page, H.M., Dugan, J.E., 2010. Essential fishery information for trap-based fisheries: Development of a framework for collaborative data collection. Marine and Coastal Fisheries 2, 98–114. https://doi.org/10.1577/C09-007.1
341. Cunha, I., Neuparth, T., Moreira, S., Santos, M.M., Reis-Henriques, M.A., 2014. Management of contaminated marine marketable resources after oil and HNS spills in Europe. Journal of Environmental Management 135, 36–44. https://doi.org/10.1016/j.jenvman.2013.12.032
342. Currie, J.J., Stack, S.H., Kaufman, G.D., 2018. Conservation and education through ecotourism: Using citizen science to monitor cetaceans in the four-island region of Maui, Hawaii. Tourism in Marine Environments 13, 65–71. https://doi.org/10.3727/154427318X15270394903273
343. Dabbous, S.A., Scott, D.B., 2012. SHORT-TERM MONITORING OF HALIFAX HARBOUR (NOVA SCOTIA, CANADA) POLLUTION REMEDIATION USING BENTHONIC FORAMINIFERA AS PROXIES. Journal of Foraminiferal Research 42, 187–205. https://doi.org/10.2113/gsjfr.42.3.187
344. Dagallier, G., Laitinen, A.I., Malartre, F., Van Campenhout, I.P.A.M., Veeken, P.C.H., 2000. Ground penetrating radar application in a shallow marine Oxfordian limestone sequence located on the eastern flank of the Paris Basin, NE France. Sedimentary Geology 130, 149–165. https://doi.org/10.1016/S0037-0738(99)00105-0
345. Dale, A.W., Regnier, P., Van Cappellen, P., Fossing, H., Jensen, J.B., Jørgensen, B.B., 2009. Remote quantification of methane fluxes in gassy marine sediments through seismic survey. Geology 37, 235–238. https://doi.org/10.1130/G25323A.1
346. Dalu, T., Froneman, P.W., 2016. Diatom-based water quality monitoring in southern Africa: challenges and future prospects. Water Sa 42, 551–559. https://doi.org/10.4314/wsa.v42i4.05
347. Dampier, J.E.E., Bell, F.W., St-Amour, M., Pitt, D.G., Luckai, N.J., 2006. Cutting versus herbicides: Tenth-year volume and release cost-effectiveness of sub-boreal conifer plantations. Forestry Chronicle 82, 521–528. https://doi.org/10.5558/tfc82521-4
348. Danovaro, R., Carugati, L., Berzano, M., Cahill, A.E., Carvalho, S., Chenuil, A., Corinaldesi, C., Cristina, S., David, R., Dell’Anno, A., Dzhembekova, N., Garcés, E., Gasol, J.M., Goela, P., Féral, J.P., Ferrera, I., Forster, R.M., Kurekin, A.A., Rastelli, E., Marinova, V., Miller, P.I., Moncheva, S., Newton, A., Pearman, J.K., Pitois, S.G., Reñé, A., Rodríguez-Ezpeleta, N., Saggiomo, V., Simis, S.G.H., Stefanova, K., Wilson, C., Martire, M.L., Greco, S., Cochrane, S.K.J., Mangoni, O., Borja, A., 2016. Implementing and innovating marine monitoring approaches for assessing marine environmental status. Frontiers in Marine Science 3. https://doi.org/10.3389/fmars.2016.00213
349. Daokun, M., Ding, Q., Li, D., Zhao, L., 2010. Wireless sensor network for continuous monitoring water quality in aquaculture farm. Sensor Letters 8, 109–113. https://doi.org/10.1166/s1.2010.1210
350. Darling, E.S., Graham, N.A.J., Januchowski-Hartley, F.A., Nash, K.L., Pratchett, M.S., Wilson, S.K., 2017. Relationships between structural complexity, coral traits, and reef fish assemblages. Coral Reefs 36, 561–575. https://doi.org/10.1007/s00338-017-1539-z
351. Darling, J.A., Tepolt, C.K., 2008. Highly sensitive detection of invasive shore crab (Carcinus maenas and Carcinus aestuarii) larvae in mixed plankton samples using polymerase chain reaction and restriction fragment length polymorphisms (PCR-RFLP). Aquatic Invasions 3, 141–152. https://doi.org/10.3391/ai.2008.3.2.4
352. Darzi-Naftchali, A., Karandish, F., Asgari, A., 2017. Diagnosing Drainage Problems in Coastal Areas Using Machine-Learning and Geostatistical Models. Irrigation and Drainage 66, 428–438. https://doi.org/10.1002/ird.2107
353. Dauvin, J.C., Gomez Gesteira, J.L., Salvande Fraga, M., 2003. Taxonomic sufficiency: An overview of its use in the monitoring of sublittoral benthic communities after oil spills. Marine Pollution Bulletin 46, 552–555. https://doi.org/10.1016/S0025-326X(03)00033-X
354. Davidson, M.A., Aarninkhof, S.G.J., Van Koningsveld, M., Holman, R.A., 2006. Developing coastal video monitoring systems in support of coastal zone management. Journal of Coastal Research 49–56.
355. Davies, C.H., Ajani, P., Armbrecht, L., Atkins, N., Baird, M.E., Beard, J., Bonham, P., Burford, M., Clementson, L., Coad, P., Crawford, C., Dela-Cruz, J., Doblin, M.A., Edgar, S., Eriksen, R., Everett, J.D., Furnas, M., Harrison, D.P., Hassler, C., Henschke, N., Hoenner, X., Ingleton, T., Jameson, I., Keesing, J., Leterme, S.C., James McLaughlin, M., Miller, M., Moffatt, D., Moss, A., Nayar, S., Patten, N.L., Patten, R., Pausina, S.A., Proctor, R., Raes, E., Robb, M., Rothlisberg, P., Saeck, E.A., Scanes, P., Suthers, I.M., Swadling, K.M., Talbot, S., Thompson, P., Thomson, P.G., Uribe-Palomino, J., Van Ruth, P., Waite, A.M., Wright, S., Richardson, A.J., 2018. A database of chlorophyll a in Australian waters. Scientific Data 5. https://doi.org/10.1038/sdata.2018.18
356. Davis Jr, R.A., Wang, P., Silverman, B.R., 2000. Comparison of the performance of three adjacent and differently constructed beach nourishment projects on the Gulf Peninsula of Florida. Journal of Coastal Research 16, 396–407.
357. Day-Lewis, F.D., Slater, L.D., Robinson, J., Johnson, C.D., Terry, N., Werkema, D., 2017. An overview of geophysical technologies appropriate for characterization and monitoring at fractured-rock sites. Journal of Environmental Management 204, 709–720. https://doi.org/10.1016/j.jenvman.2017.04.033
358. De Battisti, C., Marciano, S., Magnabosco, C., Busato, S., Arcangeli, G., Cattoli, G., 2014. Pyrosequencing as a tool for rapid fish species identification and commercial fraud detection. Journal of Agricultural and Food Chemistry 62, 198–205. https://doi.org/10.1021/jf403545m
359. De Eyto, E., Irvine, K., 2007. Assessing the status of shallow lakes using an additive model of biomass size spectra. Aquatic Conservation-Marine and Freshwater Ecosystems 17, 724–736. https://doi.org/10.1002/aqc.801
360. de Groot, J., Campbell, M., Ashley, M., Rodwell, L., 2014. Investigating the co-existence of fisheries and offshore renewable energy in the UK: Identification of a mitigation agenda for fishing effort displacement. Ocean and Coastal Management 102, 7–18. https://doi.org/10.1016/j.ocecoaman.2014.08.013
361. De Jesús-Crespo, R., Ramirez, A., 2011. The use of a Stream Visual Assessment Protocol to determine ecosystem integrity in an urban watershed in Puerto Rico. Physics and Chemistry of the Earth 36, 560–566. https://doi.org/10.1016/j.pce.2010.11.007
362. de Jonge, V.N., 2007. Toward the application of ecological concepts in EU coastal water management. Marine Pollution Bulletin 55, 407–414. https://doi.org/10.1016/j.marpolbul.2007.09.014
363. de Jonge, V.N., Elliott, M., Brauer, V.S., 2006. Marine monitoring: Its shortcomings and mismatch with the EU Water Framework Directive’s objectives. Marine Pollution Bulletin 53, 5–19. https://doi.org/10.1016/j.marpolbul.2005.11.026
364. de Juan, S., Hewitt, J., Thrush, S., Freeman, D., 2015. Standardising the assessment of Functional Integrity in benthic ecosystems. Journal of Sea Research 98, 33–41. https://doi.org/10.1016/j.seares.2014.06.001
365. De Lange, M., 2015. The need for a survey strategy: Managing UXO risk in offshore and renewables projects. Hydro International 19, 16–19.
366. de Orte, M.R., Carballeira, C., Viana, I.G., Carballeira, A., 2013. Assessing the toxicity of chemical compounds associated with marine land-based fish farms: The use of mini-scale microalgal toxicity tests. Chemistry and Ecology 29, 554–563. https://doi.org/10.1080/02757540.2013.790381
367. de Paz, J.M., Sánchez, J., Visconti, F., 2006. Combined use of GIS and environmental indicators for assessment of chemical, physical and biological soil degradation in a Spanish Mediterranean region. Journal of Environmental Management 79, 150–162. https://doi.org/10.1016/j.jenvman.2005.06.002
368. de Sá, N.C., Castro, P., Carvalho, S., Marchante, E., López-Núñez, F.A., Marchante, H., 2018. Mapping the flowering of an invasive plant using unmanned aerial vehicles: Is there potential for biocontrol monitoring? Frontiers in Plant Science 9. https://doi.org/10.3389/fpls.2018.00293
369. De Ventura, L., Kopp, K., Seppälä, K., Jokela, J., 2017. Tracing the quagga mussel invasion along the Rhine river system using eDNA markers: Early detection and surveillance of invasive zebra and quagga mussels. Management of Biological Invasions 8, 101–112. https://doi.org/10.3391/mbi.2017.8.1.10
370. Defeo, O., Lercari, D., 2004. Testing taxonomic resolution levels for ecological monitoring in sandy beach macrobenthic communities. Aquatic Conservation: Marine and Freshwater Ecosystems 14, 65–74. https://doi.org/10.1002/aqc.594
371. Dehaut, A., Cassone, A.L., Frère, L., Hermabessiere, L., Himber, C., Rinnert, E., Rivière, G., Lambert, C., Soudant, P., Huvet, A., Duflos, G., Paul-Pont, I., 2016. Microplastics in seafood: Benchmark protocol for their extraction and characterization. Environmental Pollution 215, 223–233. https://doi.org/10.1016/j.envpol.2016.05.018
372. Dekker, A., Mount, R., Jordan, A., 2007. Satellite and airborne imagery including aerial photography for benthic habitat mapping, Special Paper - Geological Association of Canada.
373. Del Brutto, O.H., Mera, R.M., Farfán, R., Castillo, P.R., 2014a. Cerebrovascular correlates of sleep disorders - Rational and protocol of a door-to-door survey in rural coastal ecuador. Journal of Stroke and Cerebrovascular Diseases 23, 1030–1039. https://doi.org/10.1016/j.jstrokecerebrovasdis.2013.08.020
374. Del Brutto, O.H., Peñaherrera, E., Ochoa, E., Santamaría, M., Zambrano, M., Del Brutto, V.J., 2014b. Door-to-door survey of cardiovascular health, stroke, and ischemic heart disease in rural coastal Ecuador - the Atahualpa Project: Methodology and operational definitions. International Journal of Stroke 9, 367–371. https://doi.org/10.1111/ijs.12030
375. Del Brutto, O.H., Zambrano, M., Peñaherrera, E., Montalván, M., Pow-Chon-Long, F., Tettamanti, D., 2013. Prevalence of the metabolic syndrome and its correlation with the cardiovascular health status in stroke- and ischemic heart disease-free Ecuadorian natives/mestizos aged ≥40 years living in Atahualpa: A population-based study. Diabetes and Metabolic Syndrome: Clinical Research and Reviews 7, 218–222. https://doi.org/10.1016/j.dsx.2013.10.006
376. Del Vecchio, S., Fantinato, E., Silan, G., Buffa, G., 2019. Trade-offs between sampling effort and data quality in habitat monitoring. Biodiversity and Conservation 28, 55–73. https://doi.org/10.1007/s10531-018-1636-5
377. de-la-Ossa-Carretero, J.A., Del-Pilar-Ruso, Y., Loya-Fernández, A., Ferrero-Vicente, L.M., Marco-Méndez, C., Martinez-Garcia, E., Giménez-Casalduero, F., Sánchez-Lizaso, J.L., 2016. Bioindicators as metrics for environmental monitoring of desalination plant discharges. Marine Pollution Bulletin 103, 313–318. https://doi.org/10.1016/j.marpolbul.2015.12.023
378. Dell, J.T., Hobday, A.J., 2008. School-based indicators of tuna population status. ICES Journal of Marine Science 65, 612–622. https://doi.org/10.1093/icesjms/fsn032
379. Delparte, D.M., Belt, M., Nishioka, C., Turner, N., Richardson, R.T., Ericksen, T., 2014. Monitoring tropical alpine lake levels in a culturally sensitive environment utilizing 3D technological approaches. Arctic, Antarctic, and Alpine Research 46, 709–718. https://doi.org/10.1657/1938-4246-46.4.709
380. Dent, D., 2007. Environmental geophysics mapping salinity and water resources. International Journal of Applied Earth Observation and Geoinformation 9, 130–136. https://doi.org/10.1016/j.jag.2006.09.005
381. Dente, M.G., Riccardo, F., Nacca, G., Ranghiasci, A., Escadafal, C., Gaayeb, L., Jiménez-Clavero, M.A., Manuguerra, J.C., Picard, M., Fernández-Pinero, J., Pérez-Ramírez, E., Robert, V., Victoir, K., Declich, S., 2018. Strengthening preparedness for arbovirus infections in mediterranean and black sea countries: A conceptual framework to assess integrated surveillance in the context of the one health strategy. International Journal of Environmental Research and Public Health 15. https://doi.org/10.3390/ijerph15030489
382. Deribe, K., Brooker, S.J., Pullan, R.L., Hailu, A., Enquselassie, F., Reithinger, R., Newport, M., Davey, G., 2013. Spatial Distribution of Podoconiosis in Relation to Environmental Factors in Ethiopia: A Historical Review. PLoS ONE 8. https://doi.org/10.1371/journal.pone.0068330
383. Descamp, P., Holon, F., Ballesta, L., Guilbert, A., Guillot, M., Boissery, P., Raimondino, V., Deter, J., 2011. Fast and easy method for seagrass monitoring: Application of acoustic telemetry to precision mapping of Posidonia oceanica beds. Marine Pollution Bulletin 62, 284–292. https://doi.org/10.1016/j.marpolbul.2010.10.012
384. Desholm, M., 2009. Avian sensitivity to mortality: Prioritising migratory bird species for assessment at proposed wind farms. Journal of Environmental Management 90, 2672–2679. https://doi.org/10.1016/j.jenvman.2009.02.005
385. Desimone, S.A., 2013. Restoration and Science: A Practitioner/Scientist’s View from Rare Habitat Restoration at a Southern California Preserve. Restoration Ecology 21, 149–152. https://doi.org/10.1111/j.1526-100X.2012.00923.x
386. Després-Einspenner, M.L., Howe, E.J., Drapeau, P., Kühl, H.S., 2017. An empirical evaluation of camera trapping and spatially explicit capture-recapture models for estimating chimpanzee density. American Journal of Primatology 79. https://doi.org/10.1002/ajp.22647
387. Detsis, E., Brodsky, Y., Knudtson, P., Cuba, M., Fuqua, H., Szalai, B., 2012. Project Catch: A space based solution to combat illegal, unreported and unregulated fishing: Part I: Vessel monitoring system. Acta Astronautica 80, 114–123. https://doi.org/10.1016/j.actaastro.2012.06.009
388. Deus, D., Gloaguen, R., 2013. Remote Sensing Analysis of Lake Dynamics in Semi-Arid Regions: Implication for Water Resource Management. Lake Manyara, East African Rift, Northern Tanzania. Water 5, 698–727. https://doi.org/10.3390/w5020698
389. Dewez, T.J.B., Yart, S., Thuon, Y., Pannet, P., Plat, E., 2017. Towards cavity-collapse hazard maps with Zeb-Revo handheld laser scanner point clouds. Photogrammetric Record 32, 354–376. https://doi.org/10.1111/phor.12223
390. Dey, M.M., Rab, M.A., Paraguas, F.J., Bhatta, R., Alam, M.F., Koeshendrajana, S., Ahmed, M., 2005. Status and economics of freshwater aquaculture in selected countries of Asia. Aquaculture Economics and Management 9, 11–37. https://doi.org/10.1080/13657300590961609
391. Diaba, K.S., Felix, K., 2015. FARMERS’ COMPARATIVE USE ASSESSMENT OF WIND AND ELECTRIC PUMP FOR IRRIGATION. Present Environment and Sustainable Development 9, 17–24. https://doi.org/10.1515/pesd-2015-0022
392. Diana, C.M., Jonas, J.L., Claramunt, R.M., Fitzsimons, J.D., Marsden, J.E., 2006. A comparison of methods for sampling round goby in rocky littoral areas. North American Journal of Fisheries Management 26, 514–522. https://doi.org/10.1577/mo5-049.1
393. Dierberg, F.E., DeBusk, T.A., 2008. Particulate phosphorus transformations in south Florida stormwater treatment areas used for Everglades protection. Ecological Engineering 34, 100–115. https://doi.org/10.1016/j.ecoleng.2008.07.013
394. Din, A.S., Kadir, H.A., Arshad, M.R., Hariri, M.H.M., 2015. Malaysian integrated ocean observation system (MIOOS) buoy. Jurnal Teknologi 74, 41–49. https://doi.org/10.11113/jt.v74.4807
395. Ding, Y., Garcia, C.D., Rogers, K.R., 2008. Poly(dimethylsiloxane) microchip electrophoresis with contactless conductivity detection for measurement of chemical warfare agent degradation products. Analytical Letters 41, 335–350. https://doi.org/10.1080/00032710701792943
396. Ding, Y., Rogers, K., 2008. Measurement of nitrogen mustard degradation products by poly(dimethylsiloxane) microchip electrophoresis with contactless conductivity detection. Electroanalysis 20, 2192–2198. https://doi.org/10.1002/elan.200804320
397. Dingwen, Z., Songyu, L., Wenjun, H., Guangyin, D., 2013. A Combined Dry Jet Mixing-Prefabricated Vertical Drain Method for Soft Ground Improvement: A Case Study. Marine Georesources and Geotechnology 31, 332–347. https://doi.org/10.1080/1064119X.2012.680679
398. Dinsdale, E.A., Harriott, V.J., 2004. Assessing Anchor Damage on Coral Reefs: A Case Study in Selection of Environmental Indicators. Environmental Management 33, 126–139. https://doi.org/10.1007/s00267-003-3056-9
399. Dissanayake, A., Galloway, T.S., 2004. Evaluation of fixed wavelength fluorescence and synchronous fluorescence spectrophotometry as a biomonitoring tool of environmental contamination. Marine Environmental Research 58, 281–285. https://doi.org/10.1016/j.marenvres.2004.03.072
400. Djodjic, F., Spännar, M., 2012. Identification of critical source areas for erosion and phosphorus losses in small agricultural catchment in central Sweden. Acta Agriculturae Scandinavica Section B: Soil and Plant Science 62, 229–240. https://doi.org/10.1080/09064710.2012.704389
401. Doering, O.C., Ribaudo, M., Diaz-Hermelo, F., Heimlich, R., Hitzhusen, F., Howard, C., Kazmierczak, R., Lee, J., Libby, L., Milon, W., Peters, M., Prato, A., 2001. Economic analysis as a basis for large-scale nitrogen control decisions: reducing nitrogen loads to the Gulf of Mexico. TheScientificWorldJournal 1 Suppl 2, 968–975.
402. Donaldson, K.A., Griffin, D.W., Paul, J.H., 2002. Detection, quantitation and identification of enteroviruses from surface waters and sponge tissue from the Florida Keys using real-time RT-PCR. Water Research 36, 2505–2514. https://doi.org/10.1016/S0043-1354(01)00479-1
403. Dong, W., Wang, F., Fang, M., Wu, J., Wang, S., Li, M., Yang, J., Chernick, M., Hinton, D.E., Pei, D.S., Chen, H., Zheng, N., Mu, J., Xie, L., Dong, W., 2019. Use of biological detection methods to assess dioxin-like compounds in sediments of Bohai Bay, China. Ecotoxicology and Environmental Safety 173, 339–346. https://doi.org/10.1016/j.ecoenv.2019.01.116
404. Donohoe, A., Lacour, G., McCluskey, P., Diamond, D., McCaul, M., 2018. Development of a cost-effective sensing platform for monitoring phosphate in natural waters. Chemosensors 6. https://doi.org/10.3390/chemosensors6040057
405. Doody, T.M., Lewis, M., Benyon, R.G., Byrne, G., 2014. A method to map riparian exotic vegetation (Salix spp.) area to inform water resource management. Hydrological Processes 28, 3809–3823. https://doi.org/10.1002/hyp.9916
406. Dorman, S.R., Harvey, E.S., Newman, S.J., 2012. Bait effects in sampling coral reef fish assemblages with stereo-BRUVs. PLoS ONE 7. https://doi.org/10.1371/journal.pone.0041538
407. Dougherty, M.M., Larson, E.R., Renshaw, M.A., Gantz, C.A., Egan, S.P., Erickson, D.M., Lodge, D.M., 2016. Environmental DNA (eDNA) detects the invasive rusty crayfish Orconectes rusticus at low abundances. Journal of Applied Ecology 53, 722–732. https://doi.org/10.1111/1365-2664.12621
408. Douvere, F., Ehler, C.N., 2011. The importance of monitoring and evaluation in adaptive maritime spatial planning. Journal of Coastal Conservation 15, 305–311. https://doi.org/10.1007/s11852-010-0100-9
409. Downey, J.T., 2017. The Reliability of Using Surface Data for Seriation. Advances in Archaeological Practice 5, 26–43. https://doi.org/10.1017/aap.2016.2
410. Dubose, B., 2016. Assessment, prioritization help slow corrosion at water treatment plant. Materials Performance 55, 12–14 and 17–19.
411. Duerdoth, C.P., Arnold, A., Murphy, J.F., Naden, P.S., Scarlett, P., Collins, A.L., Sear, D.A., Jones, J.I., 2015. Assessment of a rapid method for quantitative reach-scale estimates of deposited fine sediment in rivers. Geomorphology 230, 37–50. https://doi.org/10.1016/j.geomorph.2014.11.003
412. Duey, R., 2010. Time-lapse breaks north sea bounds. Hart’s E and P.
413. Duveiller, G., Defourny, P., Desclée, B., Mayaux, P., 2008. Deforestation in Central Africa: Estimates at regional, national and landscape levels by advanced processing of systematically-distributed Landsat extracts. Remote Sensing of Environment 112, 1969–1981. https://doi.org/10.1016/j.rse.2007.07.026
414. Dysthe, J.C., Rodgers, T., Franklin, T.W., Carim, K.J., Young, M.K., McKelvey, K.S., Mock, K.E., Schwartz, M.K., 2018. Repurposing environmental DNA samples—detecting the western pearlshell (Margaritifera falcata) as a proof of concept. Ecology and Evolution 8, 2659–2670. https://doi.org/10.1002/ece3.3898
415. Earll, R.C., Williams, A.T., Simmons, S.L., Tudor, D.T., 2000. Aquatic litter, management and prevention - the role of measurement. Journal of Coastal Conservation 6, 67–78. https://doi.org/10.1007/BF02730470
416. Echenique-Subiabre, I., Dalle, C., Duval, C., Heath, M.W., Couté, A., Wood, S.A., Humbert, J.F., Quiblier, C., 2016. Application of a spectrofluorimetric tool (bbe BenthoTorch) for monitoring potentially toxic benthic cyanobacteria in rivers. Water Research 101, 341–350. https://doi.org/10.1016/j.watres.2016.05.081
417. Eckstein, J.W., 2010. Biomarkers in Parkinsons disease: A venture capitalists perspective. Biomarkers in Medicine 4, 731–735. https://doi.org/10.2217/bmm.10.91
418. Edenborn, H.M., Brickett, L.A., 2002. Determination of manganese stability in a constructed wetland sediment using redox gel probes. Geomicrobiology Journal 19, 485–504. https://doi.org/10.1080/01490450290098450
419. Edge, K.J., Dafforn, K.A., Simpson, S.L., Roach, A.C., Johnston, E.L., 2014. A biomarker of contaminant exposure is effective in large scale assessment of ten estuaries. Chemosphere 100, 16–26. https://doi.org/10.1016/j.chemosphere.2014.01.001
420. Edwards, A.C., Sinclair, A.H., Domburg, P., 2003. Identification, designation and formulation of an action plan for a nitrate vulnerable zone: A case study of the Ythan catchment, NE Scotland. European Journal of Agronomy 20, 165–172. https://doi.org/10.1016/S1161-0301(03)00065-0
421. Edwards, H.H., Martin, J., Deutsch, C.J., Muller, R.G., Koslovsky, S.M., Smith, A.J., Barlas, M.E., 2016. Influence of Manatees’ Diving on Their Risk of Collision with Watercraft. Plos One 11. https://doi.org/10.1371/journal.pone.0151450
422. Ehmann, K., Kelleher, C., Condon, L.E., 2019. Monitoring turbidity from above: Deploying small unoccupied aerial vehicles to image in-stream turbidity. Hydrological Processes 33, 1013–1021. https://doi.org/10.1002/hyp.13372
423. Eich, M., Bonnin-Pascual, F., Garcia-Fidalgo, E., Ortiz, A., Bruzzone, G., Koveos, Y., Kirchner, F., 2014. A robot application for marine vessel inspection. Journal of Field Robotics 31, 319–341. https://doi.org/10.1002/rob.21498
424. Eisele, T.P., Silumbe, K., Finn, T., Chalwe, V., Kamuliwo, M., Hamainza, B., Moonga, H., Bennett, A., Yukich, J., Keating, J., Steketee, R.W., Miller, J.M., 2015. Assessing the effectiveness of household-level focal mass drug administration and community-wide mass drug administration for reducing malaria parasite infection prevalence and incidence in Southern Province, Zambia: Study protocol for a community randomized controlled trial. Trials 16. https://doi.org/10.1186/s13063-015-0862-3
425. Elkadiri, R., Sultan, M., Youssef, A.M., Elbayoumi, T., Chase, R., Bulkhi, A.B., Al-Katheeri, M.M., 2014. A Remote sensing-based approach for debris-flow susceptibility assessment using artificial neural networks and logistic regression modeling. IEEE Journal of Selected Topics in Applied Earth Observations and Remote Sensing 7, 4818–4835. https://doi.org/10.1109/JSTARS.2014.2337273
426. Elliott, L., Russello, M.A., 2018. SNP panels for differentiating advanced-generation hybrid classes in recently diverged stocks: A sensitivity analysis to inform monitoring of sockeye salmon re-stocking programs. Fisheries Research 208, 339–345. https://doi.org/10.1016/j.fishres.2018.09.001
427. Elliott, M., 2002. The role of the DPSIR approach and conceptual models in marine environmental management: An example for offshore wind power. Marine Pollution Bulletin 44, iii–vii. https://doi.org/10.1016/S0025-326X(02)00146-7
428. Ellison, R.M., Bendis, B., 2007. Keeping constant watch on harmful algal blooms. Sea Technology 48, 10–13.
429. Ellison, R.M., Slocum, D.B., 2009. High spatial resolution mapping of water quality and bathymetry. Sea Technology 50, 69–72.
430. Elwany, M.H.S., 2011. Characteristics, restoration, and enhancement of southern California lagoons. Journal of Coastal Research 246–255. https://doi.org/10.2112/SI59-026.1
431. Emami, K., Askari, V., Ullrich, M., Mohinudeen, K., Anil, A.C., Khandeparker, L., Burgess, J.G., Mesbahi, E., 2012. Characterization of bacteria in Ballast water using MALDI-TOF mass spectrometry. PLoS ONE 7. https://doi.org/10.1371/journal.pone.0038515
432. Embling, C.B., Walters, A.E.M., Dolman, S.J., 2015. How much effort is enough? The power of citizen science to monitor trends in coastal cetacean species. Global Ecology and Conservation 3, 867–877. https://doi.org/10.1016/j.gecco.2015.04.003
433. Emelogu, E.S., Pollard, P., Robinson, C.D., Smedes, F., Webster, L., Oliver, I.W., McKenzie, C., Seiler, T.B., Hollert, H., Moffat, C.F., 2013. Investigating the significance of dissolved organic contaminants in aquatic environments: Coupling passive sampling with in vitro bioassays. Chemosphere 90, 210–219. https://doi.org/10.1016/j.chemosphere.2012.06.041
434. Emmrich, M., Winfield, I.J., Guillard, J., Rustadbakken, A., Vergès, C., Volta, P., Jeppesen, E., Lauridsen, T.L., Brucet, S., Holmgren, K., Argillier, C., Mehner, T., 2012. Strong correspondence between gillnet catch per unit effort and hydroacoustically derived fish biomass in stratified lakes. Freshwater Biology 57, 2436–2448. https://doi.org/10.1111/fwb.12022
435. Englander, J., 2009. The international seaKeepers society: A decade of innovation and progress. Sea Technology 50, 34–35.
436. Engler, E., Gewies, S., Banyś, P., Grunewald, E., 2018. Trajectory-based multimodal transport management for resilient transportation. Transport Problems 13, 81–96. https://doi.org/10.21307/tp.2018.13.1.8
437. Environmental management of port dredging, 2001. . Asia Pacific Shipping 2, 37.
438. Erbas, C., Tuncer Cetin, F., Yilmaz, B., Akagunduz, E., Kabak, Y., Bulca, A., 2012. Open and interoperable maritime surveillance framework set to improve sea-borders control, Communications in Computer and Information Science. https://doi.org/10.1007/978-3-642-33161-9_53
439. Erftemeijer, P.L.A., Robin Lewis Iii, R.R., 2006. Environmental impacts of dredging on seagrasses: A review. Marine Pollution Bulletin 52, 1553–1572. https://doi.org/10.1016/j.marpolbul.2006.09.006
440. Eriander, L., Infantes, E., Olofsson, M., Olsen, J.L., Moksnes, P.O., 2016. Assessing methods for restoration of eelgrass (Zostera marina L.) in a cold temperate region. Journal of Experimental Marine Biology and Ecology 479, 76–88. https://doi.org/10.1016/j.jembe.2016.03.005
441. Eriksrud, M., Langhammer, J., Nakstad, H., 2009. Towards the optical oil field, SEG Technical Program Expanded Abstracts.
442. Erkkilä, A., Kalliola, R., 2004. Patterns and dynamics of coastal waters in multi-temporal satellite images: Support to water quality monitoring in the Archipelago Sea, Finland. Estuarine, Coastal and Shelf Science 60, 165–177. https://doi.org/10.1016/j.ecss.2003.11.024
443. Ernst, B., Manríquez, P., Orensanz, J.M., Roa, R., Chamorro, J., Parada, C., 2010. Strengthening of a traditional territorial tenure system through protagonism in monitoring activities by lobster fishermen from the Juan Fernández islands, Chile. Bulletin of Marine Science 86, 315–338.
444. Eskelson, B.N.I., Anderson, P.D., Temesgen, H., 2013. Modeling Relative Humidity in Headwater Forests Using Correlation with Air Temperature. Northwest Science 87, 40–58. https://doi.org/10.3955/046.087.0104
445. Esser, D., 2002. METS - The tool for pipeline inspection: Previous leakage detection methods have been unsatisfying; the MK3 version of METS helps to remove these problems. Sea Technology 43, 51–52.
446. Estes, J.A., Tinker, M.T., Bodkin, J.L., 2010. Using Ecological Function to Develop Recovery Criteria for Depleted Species: Sea Otters and Kelp Forests in the Aleutian Archipelago. Conservation Biology 24, 852–860. https://doi.org/10.1111/j.1523-1739.2009.01428.x
447. Evans, G., Truebe, M., Hanek, G., 2011. Monitoring report of frost heave on warm lake road, Transportation Research Record. https://doi.org/10.3141/2204-31
448. Evans, L.J., Jones, T.H., Pang, K., Saimin, S., Goossens, B., 2016. Spatial ecology of estuarine crocodile (Crocodylus porosus)nesting in a fragmented landscape. Sensors (Switzerland) 16. https://doi.org/10.3390/s16091527
449. Evans, S.N., Abdo, D.A., 2010. A cost-effective technique for measuring relative water movement for studies of benthic organisms. Marine and Freshwater Research 61, 1327–1335. https://doi.org/10.1071/MF10007
450. Ewing, G., Frusher, S., 2015. New puerulus collector design suitable for fishery-dependent settlement monitoring. ICES Journal of Marine Science 72, i225–i231. https://doi.org/10.1093/icesjms/fsv038
451. Fabian, V., Costa Silva, C.B., Silva Filho, J.A.P., 2000. Operational perspective of oil-in-water monitoring offshore. Boletim Tecnico da PETROBRAS 43, 120–128.
452. Fagbeja, M.A., Hill, J.L., Chatterton, T.J., Longhurst, J.W.S., 2015. A GIS-based assessment of the suitability of SCIAMACHY satellite sensor measurements for estimating reliable CO concentrations in a low-latitude climate. Environmental Monitoring and Assessment 187. https://doi.org/10.1007/s10661-014-4227-2
453. Fairclough, D.V., Brown, J.I., Carlish, B.J., Crisafulli, B.M., Keay, I.S., 2014. Breathing life into fisheries stock assessments with citizen science. Scientific Reports 4. https://doi.org/10.1038/srep07249
454. Fairclough, D.V., Edmonds, J.S., Lenanton, R.C.J., Jackson, G., Keay, I.S., Crisafulli, B.M., Newman, S.J., 2011. Rapid and cost-effective assessment of connectivity among assemblages of Choerodon rubescens (Labridae), using laser ablation ICP-MS of sagittal otoliths. Journal of Experimental Marine Biology and Ecology 403, 46–53. https://doi.org/10.1016/j.jembe.2011.04.005
455. Farré, M., Brix, R., Barceló, D., 2005. Screening water for pollutants using biological techniques under European Union funding during the last 10 years. TrAC - Trends in Analytical Chemistry 24, 532–545. https://doi.org/10.1016/j.trac.2005.03.008
456. Farré, M., Rodriguez-Mozaz, S., de Alda, M.L., Barceló, D., Hansen, P.D., 2009. Biosensors for environmental monitoring at global scale and the EU level, Handbook of Environmental Chemistry, Volume 5: Water Pollution. https://doi.org/10.1007/978-3-540-36253-1_1
457. Farrell, A., Glick, M., 2000. Natural gas as a marine propulsion fuel Energy and environmental benefits in urban ferry service, Transportation Research Record. https://doi.org/10.3141/1738-09
458. Farrell, E.D., Clarke, M.W., Mariani, S., 2009. A simple genetic identification method for Northeast Atlantic smoothhound sharks (Mustelus spp.). ICES Journal of Marine Science 66, 561–565. https://doi.org/10.1093/icesjms/fsn218
459. Fauvelle, V., Belles, A., Budzinski, H., Mazzella, N., Plus, M., 2018. Simulated conservative tracer as a proxy for S-metolachlor concentration predictions compared to POCIS measurements in Arcachon Bay. Marine Pollution Bulletin 133, 423–427. https://doi.org/10.1016/j.marpolbul.2018.06.005
460. Fauzan, M.A., Kumara, I.S.W., Yogyantoro, R., Suwardana, S., Fadhilah, N., Nurmalasari, I., Apriyani, S., Wicaksono, P., 2017. Assessing the capability of sentinel-2A data for mapping seagrass percent cover in Jerowaru, East Lombok. Indonesian Journal of Geography 49, 195–203. https://doi.org/10.22146/ijg.28407
461. Fawcett, A., Bernard, S., Pitcher, G.C., Probyn, T.A., Du Randt, A., 2006. Real-time monitoring of harmful algal blooms in the southern Benguela. African Journal of Marine Science 28, 257–260. https://doi.org/10.2989/18142320609504158
462. Ferdin, M.E., Kvitek, R.G., Bretz, C.K., Powell, C.L., Doucette, G.J., Lefebvre, K.A., Coale, S., Silver, M.W., 2002. Emerita analoga (Stimpson) - Possible new indicator species for the phycotoxin domoic acid in California coastal waters. Toxicon 40, 1259–1265. https://doi.org/10.1016/S0041-0101(02)00129-0
463. Fernandes, M., Benger, S., Sharma, S.K., Gaylard, S., Kildea, T., Hoare, S., Braley, M., Irving, A.D., 2012. The use of delta N-15 signatures of translocated macroalgae to map coastal nutrient plumes: improving species selection and spatial analysis of metropolitan datasets. Journal of Environmental Monitoring 14, 2399–2410. https://doi.org/10.1039/c2em10997b
464. Fernandes, M., Benger, S., Sharma, S.K., Gaylard, S., Kildea, T., Hoare, S., Braley, M., Irving, A.D., 2012. The use of δ15N signatures of translocated macroalgae to map coastal nutrient plumes: Improving species selection and spatial analysis of metropolitan datasets. Journal of Environmental Monitoring 14, 2399–2410. https://doi.org/10.1039/c2em10997b
465. Ferraro, S.P., Cole, F.A., Olsen, A.R., 2006. A more cost-effective EMAP benthic macrofaunal sampling protocol. Environmental Monitoring and Assessment 116, 275–290. https://doi.org/10.1007/s10661-006-7360-8
466. Ferrera, I., Giner, C.R., Reñé, A., Camp, J., Massana, R., Gasol, J.M., Garcés, E., 2016. Evaluation of alternative high-throughput sequencing methodologies for the monitoring of marine picoplanktonic biodiversity based on rRNA gene amplicons. Frontiers in Marine Science 3. https://doi.org/10.3389/fmars.2016.00147
467. Fetscher, A.E., Stancheva, R., Kociolek, J.P., Sheath, R.G., Stein, E.D., Mazor, R.D., Ode, P.R., Busse, L.B., 2014. Development and comparison of stream indices of biotic integrity using diatoms vs. non-diatom algae vs. a combination. Journal of Applied Phycology 26, 433–450. https://doi.org/10.1007/s10811-013-0088-2
468. Fezzi, C., Hutchins, M., Rigby, D., Bateman, I.J., Posen, P., Hadley, D., Deflandre-Vlandas, A., 2008. Integrated assessment of water framework directive nitrate reduction measures, Working Paper - Centre for Social and Economic Research on the Global Environment.
469. Fidalgo, F., Santos, A., Pimenta, S., Marques, J., Honrado, J., 2014. Regional Environmental Gradients Influence Ecophysiological Responses of Dominant Coastal Dune Plants to Changes in Local Conditions. Journal of Coastal Research 30, 893–903. https://doi.org/10.2112/jcoastres-d-11-00161.1
470. Filgueira, R., Chapman, J.M., Suski, C.D., Cooke, S.J., 2016. The influence of watershed land use cover on stream fish diversity and size-at-age of a generalist fish. Ecological Indicators 60, 248–257. https://doi.org/10.1016/j.ecolind.2015.06.006
471. Filgueira, R., Guyondet, T., Comeau, L.A., Grant, J., 2014. Physiological indices as indicators of ecosystem status in shellfish aquaculture sites. Ecological Indicators 39, 134–143. https://doi.org/10.1016/j.ecolind.2013.12.006
472. Filimon, M.A., Codiga, D.L., 2016. An AIS-based site planning method to help minimize collision risk during marine autonomous surface craft deployments. Journal of Atmospheric and Oceanic Technology 33, 1251–1255. https://doi.org/10.1175/JTECH-D-16-0052.1
473. Filipe, J.A.N., Cobb, R.C., Meentemeyer, R.K., Lee, C.A., Valachovic, Y.S., Cook, A.R., Rizzo, D.M., Gilligan, C.A., 2012. Landscape Epidemiology and Control of Pathogens with Cryptic and Long-Distance Dispersal: Sudden Oak Death in Northern Californian Forests. Plos Computational Biology 8. https://doi.org/10.1371/journal.pcbi.1002328
474. Filippino, K.C., Mulholland, M.R., Bott, C.B., 2015. Phycoremediation strategies for rapid tertiary nutrient removal in a waste stream. Algal Research-Biomass Biofuels and Bioproducts 11, 125–133. https://doi.org/10.1016/j.algal.2015.06.011
475. Finn, P.G., Udy, N.S., Baltais, S.J., Price, K., Coles, L., 2010. Assessing the quality of seagrass data collected by community volunteers in Moreton Bay Marine Park, Australia. Environmental Conservation 37, 83–89. https://doi.org/10.1017/S0376892910000251
476. Finnegan, W., Goggins, J., 2013. Numerical modeling to aid in the structural health monitoring of wave energy converters, Key Engineering Materials. https://doi.org/10.4028/www.scientific.net/KEM.569-570.595
477. Fisher, R., Walshe, T., Bessell-Browne, P., Jones, R., 2018. Accounting for environmental uncertainty in the management of dredging impacts using probabilistic dose–response relationships and thresholds. Journal of Applied Ecology 55, 415–425. https://doi.org/10.1111/1365-2664.12936
478. Fitch, J.E., Crowe, T.P., 2010. Effective methods for assessing ecological quality in intertidal soft-sediment habitats. Marine Pollution Bulletin 60, 1726–1733. https://doi.org/10.1016/j.marpolbul.2010.06.027
479. Flint, J., Flint, M., Limpus, C.J., Mills, P., 2017. Status of marine turtle rehabilitation in Queensland. PeerJ 2017. https://doi.org/10.7717/peerj.3132
480. Flint, J., Flint, M., Limpus, C.J., Mills, P.C., 2015. Trends in Marine Turtle Strandings along the East Queensland, Australia Coast, between 1996 and 2013. Journal of Marine Biology 2015. https://doi.org/10.1155/2015/848923
481. Florisson, J.H., Tweedley, J.R., Walker, T.H.E., Chaplin, J.A., 2018. Reef vision: A citizen science program for monitoring the fish faunas of artificial reefs. Fisheries Research 206, 296–308. https://doi.org/10.1016/j.fishres.2018.05.006
482. Flynn, D.J.H., Lynch, T.P., Barrett, N.S., Wong, L.S.C., Devine, C., Hughes, D., 2018. Gigapixel big data movies provide cost-effective seascape scale direct measurements of open-access coastal human use such as recreational fisheries. Ecology and Evolution 8, 9372–9383. https://doi.org/10.1002/ece3.4301
483. Foan, L., Ricoul, F., Vignoud, S., 2015. A novel microfluidic device for fast extraction of polycyclic aromatic hydrocarbons (PAHs) from environmental waters - comparison with stir-bar sorptive extraction (SBSE). International Journal of Environmental Analytical Chemistry 95, 1171–1185. https://doi.org/10.1080/03067319.2014.994617
484. Fontana, P.M., 2007. Exploration comes full-circle. Hart’s E and P.
485. Forcada, J., 2000. Can population surveys show if the Mediterranean monk seal colony at Cap Blanc is declining in abundance? Journal of Applied Ecology 37, 171–181. https://doi.org/10.1046/j.1365-2664.2000.00482.x
486. Ford, J., Rose, C.E., 2000. Characterizing small subbasins: A case study from coastal Oregon. Environmental Monitoring and Assessment 64, 359–377. https://doi.org/10.1023/a:1006451420945
487. Forget, G., Bagliniere, J.L., Marchand, F., Richard, A., Nevoux, M., 2018. A new method to estimate habitat potential for Atlantic salmon (Salmo salar): predicting the influence of dam removal on the Selune River (France) as a case study. Ices Journal of Marine Science 75, 2172–2181. https://doi.org/10.1093/icesjms/fsy089
488. Fornes, A., Basterretxea, G., Orfila, A., Jordi, A., Alvarez, A., Tintore, J., 2006. Mapping Posidonia oceanica from IKONOS. ISPRS Journal of Photogrammetry and Remote Sensing 60, 315–322. https://doi.org/10.1016/j.isprsjprs.2006.04.002
489. Forster, D.L., Rausch, J.N., 2002. Evaluating agricultural nonpoint-source pollution programs in two Lake Erie tributaries. Journal of Environmental Quality 31, 24–31.
490. Fox, H.E., Barnes, M.D., Ahmadia, G.N., Kao, G., Glew, L., Haisfield, K., Hidayat, N.I., Huffard, C.L., Katz, L., Mangubhai, S., Purwanto, 2017. Generating actionable data for evidence-based conservation: The global center of marine biodiversity as a case study. Biological Conservation 210, 299–309. https://doi.org/10.1016/j.biocon.2017.04.025
491. Francy, D.S., Stelzer, E.A., Brady, A.M.G., Huitger, C., Bushon, R.N., Ip, H.S., Ware, M.W., Villegas, E.N., Gallardo, V., Lindquist, H.D.A., 2013. Comparison of Filters for Concentrating Microbial Indicators and Pathogens in Lake Water Samples. Applied and Environmental Microbiology 79, 1342–1352. https://doi.org/10.1128/aem.03117-12
492. Franklin, N.M., Adams, M.S., Stauber, J.L., Lim, R.P., 2001. Development of an improved rapid enzyme inhibition bioassay with marine and freshwater microalgae using flow cytometry. Archives of Environmental Contamination and Toxicology 40, 469–480. https://doi.org/10.1007/s002440010199
493. Freidman, B.L., Camenzuli, D., Lackie, M., 2014. Locating an ice-covered Antarctic landfill using ground magnetometry. Antarctic Science 26, 361–368. https://doi.org/10.1017/S0954102013000953
494. Friberg, N., Bonada, N., Bradley, D.C., Dunbar, M.J., Edwards, F.K., Grey, J., Hayes, R.B., Hildrew, A.G., Lamouroux, N., Trimmer, M., Woodward, G., 2011. Biomonitoring of Human Impacts in Freshwater Ecosystems: The Good, the Bad and the Ugly, in: Woodward, G. (Ed.), Adv. Ecol. Res., Advances in Ecological Research. Elsevier Academic Press Inc, San Diego, pp. 1–68. https://doi.org/10.1016/b978-0-12-374794-5.00001-8
495. Friedman, E.S., Rosenbaum, M.A., Lee, A.W., Lipson, D.A., Land, B.R., Angenent, L.T., 2012. A cost-effective and field-ready potentiostat that poises subsurface electrodes to monitor bacterial respiration. Biosensors and Bioelectronics 32, 309–313. https://doi.org/10.1016/j.bios.2011.12.013
496. Frohlich, R.K., Urish, D.W., 2002. The use of geoelectrics and test wells for the assessment of groundwater quality of a coastal industrial site. Journal of Applied Geophysics 50, 261–278. https://doi.org/10.1016/S0926-9851(02)00146-5
497. FSI: Setting a new and innovative course for the future, 2003. . Sea Technology 44, 17–22.
498. Fuentes, S., Méndez, V., Aguila, P., Seeger, M., 2014. Bioremediation of petroleum hydrocarbons: Catabolic genes, microbial communities, and applications. Applied Microbiology and Biotechnology 98, 4781–4794. https://doi.org/10.1007/s00253-014-5684-9
499. Fuhrman, J.A., Liang, X., Noble, R.T., 2005. Rapid detection of enteroviruses in small volumes of natural waters by real-time quantitative reverse transcriptase PCR. Applied and Environmental Microbiology 71, 4523–4530. https://doi.org/10.1128/AEM.71.8.4523-4530.2005
500. Funaki, M., Higashino, S.I., Sakanaka, S., Iwata, N., Nakamura, N., Hirasawa, N., Obara, N., Kuwabara, M., 2014. Small unmanned aerial vehicles for aeromagnetic surveys and their flights in the South Shetland Islands, Antarctica. Polar Science 8, 342–356. https://doi.org/10.1016/j.polar.2014.07.001
501. Furlani, S., Cucchi, F., Odorico, R., 2010. A new method to study microtopographical changes in the intertidal zone: one year of TMEM measurements on a limestone removable rock slab (RRS). Zeitschrift Fur Geomorphologie 54, 137–151. https://doi.org/10.1127/0372-8854/2010/0054s2-0008
502. Gade, M., Kohlus, J., Kost, C., 2017. SAR imaging of archaeological sites on intertidal flats in the German Wadden Sea. Geosciences (Switzerland) 7. https://doi.org/10.3390/geosciences7040105
503. Gaese, H., Grauvogl, H., 2007. Comparative feasibility study on the provision of water for an irrigation scheme within the Lake Nasser Development Project in Upper Egypt. Agrartechnische Forschung-Agricultural Engineering Research 13, 77–87.
504. Gal, Y., Browne, M., Lane, C., 2014. Long-term automated monitoring of nearshore wave height from digital video. IEEE Transactions on Geoscience and Remote Sensing 52, 3412–3420. https://doi.org/10.1109/TGRS.2013.2272790
505. Gallagher, S.P., Gallagher, C.M., 2005. Discrimination of Chinook salmon, coho salmon, and steelhead redds and evaluation of the use of redd data for estimating escapement in several unregulated streams in northern California. North American Journal of Fisheries Management 25, 284–300. https://doi.org/10.1577/M04-016.1
506. Gallo, A., Boni, R., Tosti, E., 2018. Sperm viability assessment in marine invertebrates by fluorescent staining and spectrofluorimetry: A promising tool for assessing marine pollution impact. Ecotoxicology and Environmental Safety 147, 407–412. https://doi.org/10.1016/j.ecoenv.2017.07.069
507. Galloway, T.S., Brown, R.J., Browne, M.A., Dissanayake, A., Lowe, D., Depledge, M.H., Jones, M.B., 2006. The ECOMAN project: A novel approach to defining sustainable ecosystem function. Marine Pollution Bulletin 53, 186–194. https://doi.org/10.1016/j.marpolbul.2005.09.036
508. Galloway, T.S., Brown, R.J., Browne, M.A., Dissanayake, A., Lowe, D., Jones, M.B., Depledge, M.H., 2004. Ecosystem management bioindicators: The ECOMAN project - A multi-biomarker approach to ecosystem management. Marine Environmental Research 58, 233–237. https://doi.org/10.1016/j.marenvres.2004.03.064
509. Galluzzi, L., Bertozzini, E., Del Campo, A., Penna, A., Bruce, I.J., Magnani, M., 2006. Capture probe conjugated to paramagnetic nanoparticles for purification of Alexandrium species (Dinophyceae) DNA from environmental samples. Journal of Applied Microbiology 101, 36–43. https://doi.org/10.1111/j.1365-2672.2006.02952.x
510. Gao, J., Shi, H., Dai, Z., Mei, X., 2015. Variations of sediment toxicity in a tidal Estuary: A case study of the South Passage, Changjiang (Yangtze) Estuary. Chemosphere 128, 7–13. https://doi.org/10.1016/j.chemosphere.2015.01.007
511. Gardner, J.P.A., Struthers, C.D., 2013. Comparisons among survey methodologies to test for abundance and size of a highly targeted fish species. Journal of Fish Biology 82, 242–262. https://doi.org/10.1111/j.1095-8649.2012.03478.x
512. Gardner, R., Pennie, K., 2005. Ken Pennie. Aerospace International 32, 30–31.
513. Garmendia, M., Revilla, M., Zarauz, L., 2013. Testing the usefulness of a simple automatic method for particles abundance and size determination to derive cost-effective biological indicators in large monitoring networks. Hydrobiologia 704, 231–252. https://doi.org/10.1007/s10750-012-1400-x
514. Gaucherand, S., Schwoertzig, E., Clement, J.C., Johnson, B., Quétier, F., 2015. The Cultural Dimensions of Freshwater Wetland Assessments: Lessons Learned from the Application of US Rapid Assessment Methods in France. Environmental Management 56, 245–259. https://doi.org/10.1007/s00267-015-0487-z
515. Gawarkiewicz, G., Malek Mercer, A., 2019. Partnering with fishing fleets to monitor ocean conditions. Annual Review of Marine Science 11, 391–411. https://doi.org/10.1146/annurev-marine-010318-095201
516. Gavin, M.C., Solomon, J.N., Blank, S.G., 2010. Measuring and Monitoring Illegal Use of Natural Resources. Conservation Biology 24, 89–100. https://doi.org/10.1111/j.1523-1739.2009.01387.x
517. Gavrilescu, M., Demnerová, K., Aamand, J., Agathos, S., Fava, F., 2015. Emerging pollutants in the environment: Present and future challenges in biomonitoring, ecological risks and bioremediation. New Biotechnology 32, 147–156. https://doi.org/10.1016/j.nbt.2014.01.001
518. Geder, J.D., Ramamurti, R., Sandberg, W.C., Palmisano, J., Pruessner, M., Ratna, B., 2014. A 3-D unsteady hydrodynamics design methodology for underwater vehicles and appendages. Transactions - Society of Naval Architects and Marine Engineers 122, 161–176.
519. Gera, A., Alcoverro, T., Mascaró, O., Pérez, M., Romero, J., 2012. Exploring the utility of Posidonia oceanica chlorophyll fluorescence as an indicator of water quality within the European Water Framework Directive. Environmental Monitoring and Assessment 184, 3675–3686. https://doi.org/10.1007/s10661-011-2215-3
520. Gerovasileiou, V., Trygonis, V., Sini, M., Koutsoubas, D., Voultsiadou, E., 2013. Three-dimensional mapping of marine caves using a handheld echosounder. Marine Ecology Progress Series 486, 13–22. https://doi.org/10.3354/meps10374
521. Gerwing, T.G., Plate, E., 2019. Effectiveness of nutrient enhancement as a remediation or compensation strategy of salmonid fisheries in culturally oligotrophic lakes and streams in temperate climates. Restoration Ecology 27, 279–288. https://doi.org/10.1111/rec.12909
522. Geske, J., Berghout, N., van den Broek, M., 2015. Cost-effective balance between CO2 vessel and pipeline transport. Part I – Impact of optimally sized vessels and fleets. International Journal of Greenhouse Gas Control 36, 175–188. https://doi.org/10.1016/j.ijggc.2015.01.026
523. Gharachorlou, A., Ramezanianpour, A.A., 2010. Durability of Concrete Cylinder Specimens Strengthened With FRP Laminates under Penetration of Chloride Ions. International Journal of Civil Engineering 8, 327–336.
524. Ghosh, A.R., Mondal, S., Kole, D., 2018. Environmental impact assessment: A case study on East Kolkata Wetlands, in: Wastewater Management Through Aquaculture. pp. 285–303. https://doi.org/10.1007/978-981-10-7248-2_15
525. Giesy, J.P., Hilscherova, K., Jones, P.D., Kannan, K., Machala, M., 2002. Cell bioassays for detection of aryl hydrocarbon (AhR) and estrogen receptor (ER) mediated activity in environmental samples. Marine Pollution Bulletin 45, 3–16. https://doi.org/10.1016/S0025-326X(02)00097-8
526. Gil, A., Yu, Q., Lobo, A., Lourenco, P., Silva, L., Calado, H., 2011. Assessing the effectiveness of high resolution satellite imagery for vegetation mapping in small islands protected areas. Journal of Coastal Research 1663–1667.
527. Gilby, B.L., Olds, A.D., Peterson, C.H., Connolly, R.M., Voss, C.M., Bishop, M.J., Elliott, M., Grabowski, J.H., Ortodossi, N.L., Schlacher, T.A., 2018. Maximizing the benefits of oyster reef restoration for finfish and their fisheries. Fish and Fisheries 19, 931–947. https://doi.org/10.1111/faf.12301
528. Giles, H., 2008. Using Bayesian networks to examine consistent trends in fish farm benthic impact studies. Aquaculture 274, 181–195. https://doi.org/10.1016/j.aquaculture.2007.11.020
529. Gill, D.A., Oxenford, H.A., Turner, R.A., Schuhmann, P.W., 2019. Making the most of data-poor fisheries: Low cost mapping of small island fisheries to inform policy. Marine Policy 101, 198–207. https://doi.org/10.1016/j.marpol.2017.10.040
530. Gilligan, J.J., Otway, N.M., 2011. Comparison of dorsal and pectoral fin denticles for grey nurse, great white, and six whaler sharks from east Australian waters. Journal and Proceedings of the Royal Society of New South Wales 144, 66–82.
531. Giltrap, M., Macken, A., Davoren, M., Minchin, D., McGovern, E., Foley, B., Strand, J., McHugh, B., 2009. Use of caged Nucella Lapillus and Crassostrea Gigas to monitor tributyltin-induced bioeffects in Irish coastal waters. Environmental Toxicology and Chemistry 28, 1671–1678. https://doi.org/10.1897/08-384.1
532. Giri, R.K.K.V., Mandla, V.R., 2017. Study and evaluation of carbon sequestration using remote sensing and GIS: A review on various techniques. International Journal of Civil Engineering and Technology 8, 287–300.
533. Giziakis, C., Christodoulou, A., 2012. Environmental awareness and practice concerning maritime air emissions: The case of the Greek shipping industry. Maritime Policy and Management 39, 315–330. https://doi.org/10.1080/03088839.2012.671543
534. Gladstone, W., Lindfield, S., Coleman, M., Kelaher, B., 2012. Optimisation of baited remote underwater video sampling designs for estuarine fish assemblages. Journal of Experimental Marine Biology and Ecology 429, 28–35. https://doi.org/10.1016/j.jembe.2012.06.013
535. Glaholt, R.D., 2008. Investigation of the Potential Effects of Marine Pipelines on Dungeness Crab Movement and Benthic Ecology, in: Environment Concerns in Rights-of-Way Management 8th International Symposium. pp. 679–692. https://doi.org/10.1016/B978-044453223-7.50076-9
536. Goes, M., Goni, G., Dong, S., 2015. An optimal XBT-based monitoring system for the South Atlantic meridional overturning circulation at 34°S. Journal of Geophysical Research: Oceans 120, 161–181. https://doi.org/10.1002/2014JC010202
537. Goes, M., Goni, G., Dong, S.F., 2015. An optimal XBT-based monitoring system for the South Atlantic meridional overturning circulation at 34 degrees S. Journal of Geophysical Research-Oceans 120, 161–181. https://doi.org/10.1002/2014jc010202
538. Goetze, J.S., Januchowski-Hartley, F.A., Claudet, J., Langlois, T.J., Wilson, S.K., Jupiter, S.D., 2017. Fish wariness is a more sensitive indicator to changes in fishing pressure than abundance, length or biomass. Ecological Applications 27, 1178–1189. https://doi.org/10.1002/eap.1511
539. Gonzalez-Mirelis, G., Lindegarth, M., Sköld, M., 2014. Using vessel monitoring system data to improve systematic conservation planning of a multiple-use marine protected area, the Kosterhavet National Park (Sweden). Ambio 43, 162–174. https://doi.org/10.1007/s13280-013-0413-7
540. Goodman, C.A., Mutemi, W.M., Baya, E.K., Willetts, A., Marsh, V., 2006. The cost-effectiveness of improving malaria home management: shopkeeper training in rural Kenya. Health Policy and Planning 21, 275–288. https://doi.org/10.1093/heapol/czl011
541. Gordoa, A., Boada, J., García-Rubies, A., Sagué, O., 2018. Free-diving underwater fish photography contests: A complementary tool for assessing littoral fish communities. Scientia Marina 82, 95–106. https://doi.org/10.3989/scimar.04781.14A
542. Gordoa, A., Dedeu, A.L., Boada, J., 2019. Recreational fishing in Spain: First national estimates of fisher population size, fishing activity and fisher social profile. Fisheries Research 211, 1–12. https://doi.org/10.1016/j.fishres.2018.10.026
543. Goreau, T.J., Fisher, T., Perez, F., Lockhart, K., Hibbert, M., Lewin, A., 2008. Turks and Caicos Islands 2006 coral reef assessment: Large-scale environmental and ecological interactions and their management implications. Revista de Biologia Tropical 56, 25–49.
544. Goring, D.G., 2006. Models for correcting hydrographic surveys. Sea Technology 47, 31–37.
545. Gormley, K.S.G., McWhinnie, L.H., Porter, J.S., Hull, A.D., Fernandes, T.F., Sanderson, W.G., 2014. Can management effort be predicted for marine protected areas? New considerations for network design. Marine Policy 47, 138–146. https://doi.org/10.1016/j.marpol.2014.01.021
546. Goto, R., Geco, W., Kragh, E., 2009. Minimizing noise with corrected seismic: Rough seas introduce unwanted noise in 4-D surveys. New techniques help keep things quiet. Hart’s E and P.
547. Gouin, T., Harner, T., Blanchard, P., Mackay, D., 2005. Passive and active air samplers as complementary methods for investigating persistent organic pollutants in the Great Lakes Basin. Environmental Science and Technology 39, 9115–9122. https://doi.org/10.1021/es051397f
548. Grattan, S.K.T., Taylor, S.E., Basheer, P.M.A., Sun, T., Grattan, K.T.V., 2011. Sensors systems, especially fibre optic sensors in structural monitoring applications in concrete: An overview, Lecture Notes in Electrical Engineering. https://doi.org/10.1007/978-3-642-21099-0_15
549. Gray, C.A., Johnson, D.D., Reynolds, D., Rotherham, D., 2014. Development of rapid sampling procedures for an exploited bivalve in the swash zone on exposed ocean beaches. Fisheries Research 154, 205–212. https://doi.org/10.1016/j.fishres.2014.02.027
550. Gredzens, C., Marsh, H., Fuentes, M.M.P.B., Limpus, C.J., Shimada, T., Hamann, M., 2014. Satellite tracking of sympatric marine megafauna can inform the biological basis for species co-management. PLoS ONE 9. https://doi.org/10.1371/journal.pone.0098944
551. Greenberg, S., Godin, T., 2015. A Tool Supporting the Extraction of Angling Effort Data from Remote Camera Images. Fisheries 40, 276–287. https://doi.org/10.1080/03632415.2015.1038380
552. Gregalis, K.C., Schlenker, L.S., Drymon, J.M., Mareska, J.F., Powers, S.P., 2012. Evaluating the Performance of Vertical Longlines to Survey Reef Fish Populations in the Northern Gulf of Mexico. Transactions of the American Fisheries Society 141, 1453–1464. https://doi.org/10.1080/00028487.2012.703154
553. Gresham, R.M., 2009. Tilting at wind turbines. Tribology and Lubrication Technology 65, 38–39.
554. Griffiths, C.A., Patterson, T.A., Blanchard, J.L., Righton, D.A., Wright, S.R., Pitchford, J.W., Blackwell, P.G., 2018. Scaling marine fish movement behavior from individuals to populations. Ecology and Evolution 8, 7031–7043. https://doi.org/10.1002/ece3.4223
555. Griffiths, S.P., 2012. Recreational catch composition, catch rates, effort and expenditure in a specialised land-based pelagic game fish fishery. Fisheries Research 127–128, 40–44. https://doi.org/10.1016/j.fishres.2012.04.009
556. Grigorakis, K., Dimogianopoulos, D., 2010. Cost-effective and nondestructive textural assessment of fish freshness via system identification principles. Journal of Texture Studies 41, 492–510. https://doi.org/10.1111/j.1745-4603.2010.00238.x
557. Grion, S., Barsch, J., Kostov, C., Ronen, S., 2000. Good data in bad weather: Can quantity make up for quality?, SEG Technical Program Expanded Abstracts. https://doi.org/10.1190/1.1815663
558. Gröcke, D.R., Racionero-Gómez, B., Marschalek, J.W., Greenwell, H.C., 2017. Translocation of isotopically distinct macroalgae: A route to low-cost biomonitoring? Chemosphere 184, 1175–1185. https://doi.org/10.1016/j.chemosphere.2017.06.082
559. Gunerken, E., D’Hondt, E., Eppink, M., Elst, K., Wijffels, R., 2017. Flow cytometry to estimate the cell disruption yield and biomass release of Chlorella sp during bead milling. Algal Research-Biomass Biofuels and Bioproducts 25, 25–31. https://doi.org/10.1016/j.algal.2017.04.033
560. Gunes, K., Tuncsiper, B., Drizo, A., Masi, F., Ayaz, S., Tufekci, H., 2016. Constructed and riverine wetlands design considerations for domestic and agricultural diffuse pollution treatment—a case study from Turkey. Desalination and Water Treatment 57, 11988–11998. https://doi.org/10.1080/19443994.2015.1048534
561. Gustafson, L.L., Remmenga, M.D., Gardner, I.A., Hartman, K.H., Creekmore, L.H., Goodwin, A.E., Whaley, J.E., Warg, J.V., Gardner, S.L., Scott, A.E., 2014. Viral hemorrhagic septicemia IVb status in the United States: Inferences from surveillance activities and regional context. Preventive Veterinary Medicine 114, 174–187. https://doi.org/10.1016/j.prevetmed.2014.02.011
562. Guyot, A., Ostergaard, K.T., Fan, J., Santini, N.S., Lockington, D.A., 2015. Xylem hydraulic properties in subtropical coniferous trees influence radial patterns of sap flow: implications for whole tree transpiration estimates using sap flow sensors. Trees - Structure and Function 29, 961–972. https://doi.org/10.1007/s00468-014-1144-5
563. Guzinski, R., Kass, S., Huber, S., Bauer-Gottwein, P., Jensen, I.H., Naeimi, V., Doubkova, M., Walli, A., Tottrup, C., 2014. Enabling the use of earth observation data for integrated water resource management in africa with the water observation and information system. Remote Sensing 6, 7819–7839. https://doi.org/10.3390/rs6087819
564. Guzman, H.M., Condit, R., 2017. Abundance of manatees in Panama estimated from side-scan sonar. Wildlife Society Bulletin 41, 556–565. https://doi.org/10.1002/wsb.793
565. Gӧrӧcs, Z., Tamamitsu, M., Bianco, V., Wolf, P., Roy, S., Shindo, K., Yanny, K., Wu, Y., Koydemir, H.C., Rivenson, Y., Ozcan, A., 2018. A deep learning-enabled portable imaging flow cytometer for cost-effective, high-throughput, and label-free analysis of natural water samples. Light: Science and Applications 7. https://doi.org/10.1038/s41377-018-0067-0
566. Haaken, K., Furman, A., Weisbrod, N., Kemna, A., 2016. Time-lapse electrical imaging of water infiltration in the context of soil aquifer treatment. Vadose Zone Journal 15. https://doi.org/10.2136/vzj2016.04.0028
567. Haas, A.F., Guibert, M., Foerschner, A., Co, T., Calhoun, S., George, E., Hatay, M., Dinsdale, E., Sandin, S.A., Smith, J.E., Vermeij, M.J., Felts, B., Dustan, P., Salamon, P., Rohwer, F., 2015. Can we measure beauty? Computational evaluation of coral reef aesthetics. PeerJ 2015. https://doi.org/10.7717/peerj.1390
568. Hagen, P.E., Børhaue, E., Midtgaard, Ø., 2010. Pipeline inspection with interferometric SAS. Sea Technology 51, 37–40.
569. Halkos, G.E., Galani, G.K., 2014. Cost-Effectiveness analysis in reducing nutrient loading in baltic and black seas: A review. Journal of Environmental Management and Tourism 5, 29–52. https://doi.org/10.14505/jemt.v5.1(9).03
570. Halse, A.K., Schlabach, M., Sweetman, A., Jones, K.C., Breivik, K., 2012. Using passive air samplers to assess local sources versus long range atmospheric transport of POPs. Journal of Environmental Monitoring 14, 2580–2590. https://doi.org/10.1039/c2em30378g
571. Hamin, E.M., Abunnasr, Y., Dilthey, M.R., Judge, P.K., Kenney, M.A., Kirshen, P., Sheahan, T.C., DeGroot, D.J., Ryan, R.L., McAdoo, B.G., Nurse, L., Buxton, J.A., Sutton-Grier, A.E., Albright, E.A., Marin, M.A., Fricke, R., 2018. Pathways to coastal resiliency: The Adaptive Gradients Framework. Sustainability (Switzerland) 10. https://doi.org/10.3390/su10082629
572. Hanowski, J.M., Danz, N.P., Howe, R.W., Regal, R.R., Niemi, G.J., 2007. Considerations for monitoring breeding birds in Great Lakes coastal wetlands. Journal of Great Lakes Research 33, 245–252. https://doi.org/10.3394/0380-1330(2007)33[245:CFMBBI]2.0.CO;2
573. Hansen, A.G., Brendsdal, A., Arnesen, D.S., Morris, M., 2003. Optimizing an aggressive drilling program at the statfjord field. JPT, Journal of Petroleum Technology 55, 39–40.
574. Hansen, B.K., Bekkevold, D., Clausen, L.W., Nielsen, E.E., 2018. The sceptical optimist: challenges and perspectives for the application of environmental DNA in marine fisheries. Fish and Fisheries 19, 751–768. https://doi.org/10.1111/faf.12286
575. Hapke, C., Richmond, B., 2000. Monitoring beach morphology changes using small-format aerial photography and digital softcopy photogrammetry. Environmental Geosciences 7, 32–37. https://doi.org/10.1046/j.1526-0984.2000.71001.x
576. Happold, J.R., Brunhart, I., Schwermer, H., Stärk, K.D.C., 2008. Surveillance of H5 avian influenza virus in wild birds found dead. Avian Diseases 52, 100–105. https://doi.org/10.1637/8021-051407-Reg
577. Harasti, D., Malcolm, H., Gallen, C., Coleman, M.A., Jordan, A., Knott, N.A., 2015. Appropriate set times to represent patterns of rocky reef fishes using baited video. Journal of Experimental Marine Biology and Ecology 463, 173–180. https://doi.org/10.1016/j.jembe.2014.12.003
578. Harley, M.D., Turner, I.L., Short, A.D., Ranasinghe, R., 2011. Assessment and integration of conventional, RTK-GPS and image-derived beach survey methods for daily to decadal coastal monitoring. Coastal Engineering 58, 194–205. https://doi.org/10.1016/j.coastaleng.2010.09.006
579. Harner, T., Rauert, C., Muir, D., Schuster, J.K., Hsu, Y.M., Zhang, L.M., Marson, G., Watson, J.G., Ahad, J., Cho, S., Jariyasopit, N., Kirk, J., Korosi, J., Landis, M.S., Martin, J.W., Zhang, Y.F., Fernie, K., Wentworth, G.R., Wnorowski, A., Dabek, E., Charland, J.P., Pauli, B., Wania, F., Galarneau, E., Cheng, I., Makar, P., Whaley, C., Chow, J.C., Wang, X.L., 2018. Air synthesis review: polycyclic aromatic compounds in the oil sands region. Environmental Reviews 26, 430–468. https://doi.org/10.1139/er-2018-0039
580. Harper, L.R., Buxton, A.S., Rees, H.C., Bruce, K., Brys, R., Halfmaerten, D., Read, D.S., Watson, H.V., Sayer, C.D., Jones, E.P., Priestley, V., Mächler, E., Múrria, C., Garcés-Pastor, S., Medupin, C., Burgess, K., Benson, G., Boonham, N., Griffiths, R.A., Lawson Handley, L., Hänfling, B., 2019. Prospects and challenges of environmental DNA (eDNA) monitoring in freshwater ponds. Hydrobiologia 826, 25–41. https://doi.org/10.1007/s10750-018-3750-5
581. Harper, L.R., Lawson Handley, L., Hahn, C., Boonham, N., Rees, H.C., Gough, K.C., Lewis, E., Adams, I.P., Brotherton, P., Phillips, S., Hänfling, B., 2018. Needle in a haystack? A comparison of eDNA metabarcoding and targeted qPCR for detection of the great crested newt (Triturus cristatus). Ecology and Evolution 8, 6330–6341. https://doi.org/10.1002/ece3.4013
582. Harrington, C., 2006. US military commanders seek low-cost satellite capability to aid missions. Jane’s Defence Weekly.
583. Hart, R.C., 2001. Two calanoids, two lakes, and a decade or two. An updated record and evaluation of occurrence and periodicity of Tropodiaptomus spectabilis and Metadiaptomus meridianus (Copepoda: Calanoida), and alternative stable states in two cascading impoundments. Hydrobiologia 453–454, 269–283. https://doi.org/10.1023/A:1013157109927
584. Hartill, B.W., Payne, G.W., Rush, N., Bian, R., 2016. Bridging the temporal gap: Continuous and cost-effective monitoring of dynamic recreational fisheries by web cameras and creel surveys. Fisheries Research 183, 488–497. https://doi.org/10.1016/j.fishres.2016.06.002
585. Haseler, M., Schernewski, G., Balciunas, A., Sabaliauskaite, V., 2018. Monitoring methods for large micro- and meso-litter and applications at Baltic beaches. Journal of Coastal Conservation 22, 27–50. https://doi.org/10.1007/s11852-017-0497-5
586. Hashim, R., Kamali, B., Tamin, N.M., Zakaria, R., 2010. An integrated approach to coastal rehabilitation: Mangrove restoration in Sungai Haji Dorani, Malaysia. Estuarine, Coastal and Shelf Science 86, 118–124. https://doi.org/10.1016/j.ecss.2009.10.021
587. Hassall, M., Lane, S.J., 2001. Effects of varying rates of autumn fertilizer applications to pastures in eastern England on feeding sites selection by brent geese Branta b. bernicla. Agriculture Ecosystems & Environment 86, 203–209. https://doi.org/10.1016/s0167-8809(00)00282-6
588. Hassan, M., Abdelhamid, M., Nassef, O.A., Abdel Harith, M., 2018. Spectrochemical Analytical Follow up of Phytoremediation of Oil-Contaminated Soil. Soil and Sediment Contamination 27, 485–500. https://doi.org/10.1080/15320383.2018.1485631
589. Hassett, B., Palmer, M., Bernhardt, E., Smith, S., Carr, J., Hart, D., 2005. Restoring watersheds project by project: Trends in Chesapeake Bay tributary restoration. Frontiers in Ecology and the Environment 3, 259–267. https://doi.org/10.1890/1540-9295(2005)003[0259:RWPBPT]2.0.CO;2
590. Hayes, K.R., Cannon, R., Neil, K., Inglis, G., 2005. Sensitivity and cost considerations for the detection and eradication of marine pests in ports. Marine Pollution Bulletin 50, 823–834. https://doi.org/10.1016/j.marpolbul.2005.02.032
591. He, Y.L., McLaughlin, S., Lo, J.S.H., Shi, C., Lenos, J., Vincelli, A., 2014. Radio frequency identification (RFID) based corrosion monitoring sensors Part 2 - Application and testing of coating materials. Corrosion Engineering Science and Technology 49, 695–704. https://doi.org/10.1179/1743278214Y.0000000212
592. Heblinski, J., Schmieder, K., Heege, T., Agyemang, T.K., Sayadyan, H., Vardanyan, L., 2011. High-resolution satellite remote sensing of littoral vegetation of Lake Sevan (Armenia) as a basis for monitoring and assessment. Hydrobiologia 661, 97–111. https://doi.org/10.1007/s10750-010-0466-6
593. Hedley, J.D., Roelfsema, C.M., Chollett, I., Harborne, A.R., Heron, S.F., Weeks, S.J., Skirving, W.J., Strong, A.E., Mark Eakin, C., Christensen, T.R.L., Ticzon, V., Bejarano, S., Mumby, P.J., 2016. Remote sensing of coral reefs for monitoring and management: A review. Remote Sensing 8. https://doi.org/10.3390/rs8020118
594. Hegg, D., Giroir, T., Ellenberg, U., Seddon, P.J., 2012. Yellow-eyed Penguin (Megadyptes antipodes) as a case study to assess the reliability of nest counts. Journal of Ornithology 153, 457–466. https://doi.org/10.1007/s10336-011-0761-7
595. Heidtke, T., Hartig, J.H., Zarull, M.A., Yu, B., 2006. PCB levels and trends within the Detroit River-Western Lake Erie basin: A historical perspective of ecosystem monitoring. Environmental Monitoring and Assessment 112, 23–33. https://doi.org/10.1007/s10661-006-0212-8
596. Heinrich, C., Feldens, P., Schwarzer, K., 2017. Highly dynamic biological seabed alterations revealed by side scan sonar tracking of Lanice conchilega beds offshore the island of Sylt (German Bight). Geo-Marine Letters 37, 289–303. https://doi.org/10.1007/s00367-016-0477-z
597. Helland, I.P., Uglem, I., Jansen, P.A., Diserud, O.H., Bjørn, P.A., Finstad, B., 2015. Statistical and ecological challenges of monitoring parasitic salmon lice infestations in wild salmonid fish stocks. Aquaculture Environment Interactions 7, 267–280. https://doi.org/10.3354/AEI00155
598. Helle, I., Ahtiainen, H., Luoma, E., Hänninen, M., Kuikka, S., 2015. A probabilistic approach for a cost-benefit analysis of oil spill management under uncertainty: A Bayesian network model for the Gulf of Finland. Journal of Environmental Management 158, 122–132. https://doi.org/10.1016/j.jenvman.2015.04.042
599. Henderson, J., Pizarro, O., Johnson-Roberson, M., Mahon, I., 2013. Mapping submerged archaeological sites using stereo-vision photogrammetry. International Journal of Nautical Archaeology 42, 243–256. https://doi.org/10.1111/1095-9270.12016
600. Herbort, A.F., Schuhen, K., 2017. A concept for the removal of microplastics from the marine environment with innovative host-guest relationships. Environmental Science and Pollution Research 24, 11061–11065. https://doi.org/10.1007/s11356-016-7216-x
601. Hering, D., Borja, A., Jones, J.I., Pont, D., Boets, P., Bouchez, A., Bruce, K., Drakare, S., Hänfling, B., Kahlert, M., Leese, F., Meissner, K., Mergen, P., Reyjol, Y., Segurado, P., Vogler, A., Kelly, M., 2018. Implementation options for DNA-based identification into ecological status assessment under the European Water Framework Directive. Water Research 138, 192–205. https://doi.org/10.1016/j.watres.2018.03.003
602. Herle, S., Becker, R., Blankenbach, J., 2018. IoT and Sensor Web technologies enabling real-time monitoring of hydraulic constructions. Wasserwirtschaft 108, 34–39. https://doi.org/10.1007/s35147-018-0203-z
603. Herle, S., Becker, R., Blankenbach, J., 2018. LoT and Sensor Web technologies enabling real-time monitoring of hydraulic constructions. WasserWirtschaft 108, 34–39. https://doi.org/10.1007/s35147-018-0203-z
604. Hermoso, V., Januchowski-Hartley, S.R., Linke, S., 2015. Systematic planning of disconnection to enhance conservation success in a modified world. Science of the Total Environment 536, 1038–1044. https://doi.org/10.1016/j.scitotenv.2015.07.120
605. Hermoso, V., Pantus, F., Olley, J., Linke, S., Mugodo, J., Lea, P., 2012. Systematic planning for river rehabilitation: Integrating multiple ecological and economic objectives in complex decisions. Freshwater Biology 57, 1–9. https://doi.org/10.1111/j.1365-2427.2011.02693.x
606. Hernández-Carrasco, I., Solabarrieta, L., Rubio, A., Esnaola, G., Reyes, E., Orfila, A., 2018. Impact of HF radar current gap-filling methodologies on the Lagrangian assessment of coastal dynamics. Ocean Science 14, 827–847. https://doi.org/10.5194/os-14-827-2018
607. Hernandez-Paniagua, I.Y., Ramirez-Vargas, R., Ramos-Gomez, M.S., Dendooven, L., Avelar-Gonzalez, F.J., Thalasso, F., 2014. Greenhouse gas emissions from stabilization ponds in subtropical climate. Environmental Technology (United Kingdom) 35, 727–734. https://doi.org/10.1080/09593330.2013.848910
608. Heron, S.F., Liu, G., Rauenzahn, J.L., Christensen, T.R.L., Skirving, W.J., Burgess, T.F.R., Eakin, C.M., Morgan, J.A., 2014. Improvements to and continuity of operational global thermal stress monitoring for coral bleaching. Journal of Operational Oceanography 7, 3–11. https://doi.org/10.1080/1755876X.2014.11020154
609. Hicks, B.J., Stichbury, G.A., Brabyn, L.K., Allan, M.G., Ashraf, S., 2013. Hindcasting water clarity from Landsat satellite images of unmonitored shallow lakes in the Waikato region, New Zealand. Environmental Monitoring and Assessment 185, 7245–7261. https://doi.org/10.1007/s10661-013-3098-2
610. High-accuracy surveying for Ministry of Defence, 2009. . GEO: connexion 8, 32–34.
611. Hill, B.H., Elonen, C.M., Herlihy, A.T., Jicha, T.M., Serenbetz, G., 2018. Microbial ecoenzyme stoichiometry, nutrient limitation, and organic matter decomposition in wetlands of the conterminous United States. Wetlands Ecology and Management 26, 425–439. https://doi.org/10.1007/s11273-017-9584-5
612. Hilton, A.E., Bausell, J.T., Kudela, R.M., 2018. Quantification of Polychlorinated Biphenyl (PCB) Concentration in San Francisco Bay Using Satellite Imagery. Remote Sensing 10. https://doi.org/10.3390/rs10071110
613. Hitz, G., Pomerleau, F., Garneau, M.E., Pradalier, C., Posch, T., Pernthaler, J., Siegwart, R.Y., 2012. Autonomous inland water monitoring: Design and application of a surface vessel. IEEE Robotics and Automation Magazine 19, 62–72. https://doi.org/10.1109/MRA.2011.2181771
614. Hjellvik, V., Godø, O.R., Tjøstheim, D., 2004. Decomposing and explaining the variability of bottom trawl survey data from the Barents Sea. Sarsia 89, 196–210. https://doi.org/10.1080/00364820410006376
615. Hjerppe, T., Taskinen, A., Kotamäki, N., Malve, O., Kettunen, J., 2017. Probabilistic Evaluation of Ecological and Economic Objectives of River Basin Management Reveals a Potential Flaw in the Goal Setting of the EU Water Framework Directive. Environmental Management 59, 584–593. https://doi.org/10.1007/s00267-016-0806-z
616. Hjorth, M., Haller, R., Dahllöf, I., 2006. The use of 14C tracer technique to assess the functional response of zooplankton community grazing to toxic impact. Marine Environmental Research 61, 339–351. https://doi.org/10.1016/j.marenvres.2005.11.003
617. Hodge, J., Longstaff, B., Steven, A., Thornton, P., Ellis, P., McKelvie, I., 2005. Rapid underway profiling of water quality in Queensland estuaries. Marine Pollution Bulletin 51, 113–118. https://doi.org/10.1016/j.marpolbul.2004.10.043
618. Hodlur, G.K., Dhakate, R., Andrade, R., 2006. Correlation of vertical electrical sounding and borehole-log data for delineation of saltwater and freshwater aquifers. Geophysics 71, G11–G20. https://doi.org/10.1190/1.2169847
619. Hoenicke, R., Davis, J.A., Gunther, A., Mumley, T.E., Abu-Saba, K., Taberski, K., 2003. Effective application of monitoring information: The case of San Francisco Bay. Environmental Monitoring and Assessment 81, 15–25. https://doi.org/10.1023/A:1021344117229
620. Hoffman, E., Lyons, J., Boxall, J., Robertson, C., Lake, C.B., Walker, T.R., 2017. Spatiotemporal assessment (quarter century) of pulp mill metal(loid) contaminated sediment to inform remediation decisions. Environmental Monitoring and Assessment 189. https://doi.org/10.1007/s10661-017-5952-0
621. Holmes, P.M., 2008. Optimal ground preparation treatments for restoring lowland Sand Fynbos vegetation on old fields. South African Journal of Botany 74, 33–40. https://doi.org/10.1016/j.sajb.2007.08.005
622. Holopainen, S., Arzel, C., Elmberg, J., Fox, A.D., Guillemain, M., Gunnarsson, G., Nummi, P., Sjöberg, K., Väänänen, V.M., Alhainen, M., Pöysä, H., 2018. Sustainable management of migratory European ducks: Finding model species. Wildlife Biology 2018. https://doi.org/10.2981/wlb.00336
623. Hong, T., Fisk, W.J., 2010. Assessment of energy savings potential from the use of demand controlled ventilation in general office spaces in California. Building Simulation 3, 117–124. https://doi.org/10.1007/s12273-010-0001-8
624. Honrado, J., Vicente, J., Lomba, A., Alves, P., Macedo, J.A., Henriques, R., Granja, H., Caldas, F.B., 2010. Fine-scale patterns of vegetation assembly in the monitoring of changes in coastal sand-dune landscapes. Web Ecology 10, 1–14. https://doi.org/10.5194/we-10-1-2010
625. Horan, R.D., Lupi, F., 2005. Tradeable risk permits to prevent alien species into future introductions of invasive the Great Lakes. Ecological Economics 52, 289–304. https://doi.org/10.1016/j.ecolecon.2004.06.018
626. Horan, R.D., Lupi, F., 2005. Tradeable risk permits to prevent future introductions of invasive alien species into the Great Lakes. Ecological Economics 52, 289–304. https://doi.org/10.1016/j.ecolecon.2004.06.018
627. Howard, M., Harding, C., Stoughton, D., 2007. Rich azimuth marine seismic, a cost effective approach to better subsalt images. First Break 25, 63–68.
628. Howarth, R.W., 2005. The development of policy approaches for reducing nitrogen pollution to coastal waters of the USA. Science in China. Series C, Life sciences / Chinese Academy of Sciences 48 Spec No, 791–806.
629. Howe, B.M., Miller, J.H., 2004. Acoustic sensing for ocean research. Marine Technology Society Journal 38, 144–154. https://doi.org/10.4031/002533204787522811
630. Hoyer, M.V., Wellendorf, N., Frydenborg, R., Bartlett, D., Canfield Jr, D.E., 2012. A comparison between professionally (Florida department of environmental protection) and volunteer (Florida LAKEWATCH) collected trophic state chemistry data in Florida. Lake and Reservoir Management 28, 277–281. https://doi.org/10.1080/07438141.2012.736016
631. Hu, B., Zhao, R., Chen, S., Zhou, Y., Jin, B., Li, Y., Shi, Z., 2018. Heavy metal pollution delineation based on uncertainty in a coastal industrial city in the yangtze river delta, China. International Journal of Environmental Research and Public Health 15. https://doi.org/10.3390/ijerph15040710
632. Hu, X.L., Bao, Y.F., Hu, J.J., Liu, Y.Y., Yin, D.Q., 2017. Occurrence of 25 pharmaceuticals in Taihu Lake and their removal from two urban drinking water treatment plants and a constructed wetland. Environmental Science and Pollution Research 24, 14889–14902. https://doi.org/10.1007/s11356-017-8830-y
633. Huang, J., Bennett, W.W., Teasdale, P.R., Kankanamge, N.R., Welsh, D.T., 2017. A modified DGT technique for the simultaneous measurement of dissolved inorganic nitrogen and phosphorus in freshwaters. Analytica Chimica Acta 988, 17–26. https://doi.org/10.1016/j.aca.2017.08.024
634. Huang, W., Murray, C., Kraus, N., Rosati, J., 2003. Development of a regional neural network for coastal water level predictions. Ocean Engineering 30, 2275–2295. https://doi.org/10.1016/S0029-8018(03)00083-0
635. Huang, Y.C., Tang, P.K., Lee, Y.C., Lin, Y.R., 2011. Back-propagation neural network on characterization of groundwater quality in Pingtung Champaign, Taiwan, Applied Mechanics and Materials. https://doi.org/10.4028/www.scientific.net/AMM.58-60.2456
636. Hubler, S., Huff, D.D., Edwards, P., Pan, Y.D., 2016. The Biological Sediment Tolerance Index: Assessing fine sediments conditions in Oregon streams using macroinvertebrates. Ecological Indicators 67, 132–145. https://doi.org/10.1016/j.ecolind.2016.02.009
637. Hudson, H.R., Harding, J.S., 2004. Drainage management in New Zealand: A review of existing activities and alternative management practices, Science for Conservation.
638. Hughes, K.A., Ashton, G.V., 2017. Breaking the ice: the introduction of biofouling organisms to Antarctica on vessel hulls. Aquatic Conservation: Marine and Freshwater Ecosystems 27, 158–164. https://doi.org/10.1002/aqc.2625
639. Hughes, R.M., Herlihy, A.T., Gerth, W.J., Pan, Y., 2012. Estimating vertebrate, benthic macroinvertebrate, and diatom taxa richness in raftable pacific northwest rivers for bioassessment purposes. Environmental Monitoring and Assessment 184, 3185–3198. https://doi.org/10.1007/s10661-011-2181-9
640. Huikuri, P., Salonen, L., 2000. Removal of uranium from Finnish groundwaters in domestic use with a strong base anion resin. Journal of Radioanalytical and Nuclear Chemistry 245, 385–393. https://doi.org/10.1023/a:1006787111010
641. Hulley, E.N., Taylor, N.D.J., Zarnke, A.M., Somers, C.M., Manzon, R.G., Wilson, J.Y., Boreham, D.R., 2018. DNA barcoding vs. morphological identification of larval fish and embryos in Lake Huron: Advantages to a molecular approach. Journal of Great Lakes Research 44, 1110–1116. https://doi.org/10.1016/j.jglr.2018.07.013
642. Hulley, E.N., Tharmalingam, S., Zarnke, A., Boreham, D.R., 2019. Development and validation of probe-based multiplex real-time PCR assays for the rapid and accurate detection of freshwater fish species. PLoS ONE 14. https://doi.org/10.1371/journal.pone.0210165
643. Humphrey, H.E.B., Joseph, J.C., Pandya, J.R., Sweeney, A.M., Gasior, D.M., McCaffrey, R.J., Schantz, S.L., 2000. PCB congener profile in the serum of humans consuming Great Lakes fish. Environmental Health Perspectives 108, 167–172. https://doi.org/10.1289/ehp.00108167
644. Hunt, J., 2006. Re-injecting gases. Turbomachinery International 47, 22–25.
645. Hunt, T.L., Scarborough, H., Giri, K., Douglas, J.W., Jones, P., 2017. Assessing the cost-effectiveness of a fish stocking program in a culture-based recreational fishery. Fisheries Research 186, 468–477. https://doi.org/10.1016/j.fishres.2016.09.003
646. Huse, S.M., Dethlefsen, L., Huber, J.A., Welch, D.M., Relman, D.A., Sogin, M.L., 2008. Exploring microbial diversity and taxonomy using SSU rRNA hypervariable tag sequencing. PLoS Genetics 4. https://doi.org/10.1371/journal.pgen.1000255
647. Huspeni, T.C., Hechinger, R.F., Lafferty, K.D., 2004. Trematode parasites as estuarine indicators: Opportunities, applications, and comparisons with conventional community approaches, in: Estuarine Indicators. pp. 297–314. https://doi.org/10.1201/9781420038187
648. Hussain, N.A.A., Ali, S.S.A., Saad, M.N.M., Ovinis, M., Nordin, N., Adil, S.H., 2017. Underactuated nonlinear adaptive control approach using U-model incorporated with RBFNN for multivariable underwater glider control parameters. Indian Journal of Geo-Marine Sciences 46, 2482–2492.
649. Hutchins, M., Fezzi, C., Bateman, I., Posen, P., Deflandre-Vlandas, A., 2009. Cost-effective mitigation of diffuse pollution: Setting criteria for river basin management at multiple locations. Environmental Management 44, 256–267. https://doi.org/10.1007/s00267-009-9306-8
650. Hutchinson, T.H., 2002. Reproductive and developmental effects of endocrine disrupters in invertebrates: In vitro and in vivo approaches. Toxicology Letters 131, 75–81. https://doi.org/10.1016/S0378-4274(02)00046-2
651. Hutchinson, T.H., Lyons, B.P., Thain, J.E., Law, R.J., 2013. Evaluating legacy contaminants and emerging chemicals in marine environments using adverse outcome pathways and biological effects-directed analysis. Marine Pollution Bulletin 74, 517–525. https://doi.org/10.1016/j.marpolbul.2013.06.012
652. Hutchison, K.D., 2003. Applications of MODIS satellite data and products for monitoring air quality in the state of Texas. Atmospheric Environment 37, 2403–2412. https://doi.org/10.1016/S1352-2310(03)00128-6
653. Huveneers, C., Luo, K., Otway, N.M., Harcourt, R.G., 2009. Assessing the distribution and relative abundance of wobbegong sharks (Orectolobidae) in New South Wales, Australia, using recreational scuba-divers. Aquatic Living Resources 22, 255–264. https://doi.org/10.1051/alr/2009046
654. Hyder, K., Townhill, B., Anderson, L.G., Delany, J., Pinnegar, J.K., 2015. Can citizen science contribute to the evidence-base that underpins marine policy? Marine Policy 59, 112–120. https://doi.org/10.1016/j.marpol.2015.04.022
655. Ibrahim, A.N., Mabuchi, Y., Murakami, M., 2005. Remote sensing algorithms for monitoring eutrophication in Ishizuchi storm water reservoir in Kochi Prefecture, Japan. Hydrological Sciences Journal 50, 525–542. https://doi.org/10.1623/hysj.50.3.525.65024
656. Imani, S., Niksokhan, M.H., Jamshidi, S., Abbaspour, K.C., 2017. Discharge permit market and farm management nexus: an approach for eutrophication control in small basins with low-income farmers. Environmental Monitoring and Assessment 189. https://doi.org/10.1007/s10661-017-6066-4
657. Inazaki, T., 2004. High-resolution seismic reflection surveying at paved areas using an S-wave type Land Streamer. Exploration Geophysics 35, 1–6. https://doi.org/10.1071/EG04001
658. Indraratna, B., Balasubramaniam, A.S., Poulos, H., Rujikiatkamjorn, C., Ameratunga, J., 2013. Performance and prediction of marine clay treated with vacuum and surcharge consolidation at port of brisbane. Australian Geomechanics Journal 48, 161–180.
659. Indraratna, B., Rujikiatkamjorn, C., Ameratunga, J., Boyle, P., 2011. Performance and prediction of vacuum combined surcharge consolidation at port of brisbane. Journal of Geotechnical and Geoenvironmental Engineering 137, 1009–1018. https://doi.org/10.1061/(ASCE)GT.1943-5606.0000519
660. Indraratna, B., Rujikiatkamjorn, C., Baral, P., Ameratunga, J., 2018. Performance of marine clay stabilised with vacuum pressure: Based on Queensland experience. Journal of Rock Mechanics and Geotechnical Engineering. https://doi.org/10.1016/j.jrmge.2018.11.002
661. Ingenbleek, L., Jazet, E., Dzossa, A.D., Adebayo, S.B., Ogungbangbe, J., Dansou, S., Diallo, Z.J., Kouebou, C., Adegboye, A., Hossou, E., Coulibaly, S., Eyangoh, S., Le Bizec, B., Verger, P., Kamanzi, J., Merten, C., Leblanc, J.C., 2017. Methodology design of the regional Sub-Saharan Africa Total Diet Study in Benin, Cameroon, Mali and Nigeria. Food and Chemical Toxicology 109, 155–169. https://doi.org/10.1016/j.fct.2017.08.017
662. Ingleton, T., McMinn, A., 2012. Thermal plume effects: A multi-disciplinary approach for assessing effects of thermal pollution on estuaries using benthic diatoms and satellite imagery. Estuarine, Coastal and Shelf Science 99, 132–144. https://doi.org/10.1016/j.ecss.2011.12.024
663. Ingraham, D., Beresford, R., Kaluri, K., Ndoh, M., Srinivasan, K., 2005. Wireless sensors: Oyster habitat monitoring in the Bras d’Or Lakes, in: Prasanna, V.K., Iyengar, S., Spirakis, P., Welsh, M. (Eds.), Distributed Computing in Sensor Systems, Proceedings, Lecture Notes in Computer Science. Springer-Verlag Berlin, Berlin, pp. 399–400.
664. Intamaso, U., Boonbanjong, P., Wisunthorn, S., Ong-in, N., Keawpo, B., Poomipak, W., 2015. DETECTION BY DUPLEX RT-COUPLED NESTED PCR OF HEPATITIS A AND ROTAVIRUS IN OYSTERS FROM THAILAND EAST COAST. The Southeast Asian journal of tropical medicine and public health 46, 624–639.
665. Ionescu, M.S., Wilson, S.E., Evans, E.J., 2016. Jellyfish stranding observations around the Isle of Anglesey in the summer of 2014. Geo-Eco-Marina 2016, 109–118.
666. Iwamoto, S., Checkley, J.D.M., Trivedi, M.M., 2001. REFLICS: Real-time flow imaging and classification system. Machine Vision and Applications 13, 1–13. https://doi.org/10.1007/PL00013270
667. Ivan, I.A., Stihi, V., Ivan, M., Stihi, C., Rakotondrabe, M., Jelea, A., 2011. Battery powered cost effective TDS logger intended for water testing. Romanian Reports of Physics 56, 540–549.
668. Ivanov, A.Y., 2010. Application of the synthetic aperture radar for monitoring activity and environmental parameters during oil-platform installation. International Journal of Remote Sensing 31, 4835–4851. https://doi.org/10.1080/01431161.2010.485221
669. Iverson, S.A., Boyd, W.S., Esler, D., Mulcahy, D.M., Bowman, T.D., 2006. Comparison of the effects and performance of four types of radiotransmitters for use with scoters. Wildlife Society Bulletin 34, 656–663. https://doi.org/10.2193/0091-7648(2006)34[656:COTEAP]2.0.CO;2
670. Jabornig, S., Podmirseg, S.M., 2015. A Novel Fixed Fibre Biofilm Membrane Process for On-Site Greywater Reclamation Requiring No Fouling Control. Biotechnology and Bioengineering 112, 484–493. https://doi.org/10.1002/bit.25449
671. Jackson, C.J., Preston, N., Burford, M.A., Thompson, P.J., 2003. Managing the development of sustainable shrimp farming in Australia: The role of sedimentation ponds in treatment of farm discharge water. Aquaculture 226, 23–34. https://doi.org/10.1016/S0044-8486(03)00464-2
672. Jackson, G., Cheng, Y.W., Wakefield, C.B., 2012. An evaluation of the daily egg production method to estimate spawning biomass of snapper (Pagrus auratus) in inner Shark Bay, Western Australia, following more than a decade of surveys 1997-2007. Fisheries Research 117–118, 22–34. https://doi.org/10.1016/j.fishres.2010.12.009
673. Jackson, L.A., Hornsey, W.P., 2002. Engineering an artificial reef. Geotechnical Fabrics Report 20, 18–25.
674. Jacobson, C., Carter, R.W., Thomsen, D.C., Smith, T.F., 2014. Monitoring and evaluation for adaptive coastal management. Ocean and Coastal Management 89, 51–57. https://doi.org/10.1016/j.ocecoaman.2013.12.008
675. Javeline, D., Kijewski-Correa, T., 2019. Coastal homeowners in a changing climate. Climatic Change 152, 259–274. https://doi.org/10.1007/s10584-018-2257-4
676. Jeppesen, E., Nõges, P., Davidson, T.A., Haberman, J., Nõges, T., Blank, K., Lauridsen, T.L., Søndergaard, M., Sayer, C., Laugaste, R., Johansson, L.S., Bjerring, R., Amsinck, S.L., 2011. Zooplankton as indicators in lakes: A scientific-based plea for including zooplankton in the ecological quality assessment of lakes according to the European Water Framework Directive (WFD). Hydrobiologia 676, 279–297. https://doi.org/10.1007/s10750-011-0831-0
677. Jessop, T.S., Sumner, J., Rudiharto, H., Purwandana, D., Imansyah, M.J., Phillips, J.A., 2004. Distribution, use and selection of nest type by Komodo Dragons. Biological Conservation 117, 463–470. https://doi.org/10.1016/j.biocon.2003.08.005
678. Jeunen, G.J., Knapp, M., Spencer, H.G., Taylor, H.R., Lamare, M.D., Stat, M., Bunce, M., Gemmell, N.J., 2019. Species-level biodiversity assessment using marine environmental DNA metabarcoding requires protocol optimization and standardization. Ecology and Evolution 9, 1323–1335. https://doi.org/10.1002/ece3.4843
679. Ji, Y.Q., Li, J.Y., Luo, S.G., Wu, T., Liu, J.L., 2001. Determination of traces of Np-237 in environmental samples by ICP-MS after separation using TOA extraction chromatography. Fresenius Journal of Analytical Chemistry 371, 49–53. https://doi.org/10.1007/s002160100923
680. Jiaguo, Q., 2007. Remote sensing’s role in water quality assessment. Water 21 31.
681. Jiang, B., Li, G., Xing, Y., Zhang, D., Jia, J., Cui, Z., Luan, X., Tang, H., 2017. A whole-cell bioreporter assay for quantitative genotoxicity evaluation of environmental samples. Chemosphere 184, 384–392. https://doi.org/10.1016/j.chemosphere.2017.05.159
682. Jiang, X., Zhang, Q., Zhao, H., Geng, G., Peng, L., Guan, D., Kan, H., Huo, H., Lin, J., Brauer, M., Martin, R.V., He, K., 2015. Revealing the hidden health costs embodied in chinese exports. Environmental Science and Technology 49, 4381–4388. https://doi.org/10.1021/es506121s
683. Jiang, Y., 2009. China’s water scarcity. Journal of Environmental Management 90, 3185–3196. https://doi.org/10.1016/j.jenvman.2009.04.016
684. Jiao, C., Zheng, G., Shang, G., Sun, D., 2016. Coastal soil clay content estimation using reflectance spectroscopy. Nongye Gongcheng Xuebao/Transactions of the Chinese Society of Agricultural Engineering 32, 137–141. https://doi.org/10.11975/j.issn.1002-6819.2016.05.019
685. Johannessen, J.A., Le Traon, P.Y., Robinson, I., Nittis, K., Bell, M., Pinardi, N., Bahurel, P., Furevik, B., 2003. Marine Environment and Security for the European Area, MERSEA Strand-1, Elsevier Oceanography Series. https://doi.org/10.1016/S0422-9894(03)80045-4
686. Johnson, D.D., Rotherham, D., Gray, C.A., 2008. Sampling estuarine fish and invertebrates using demersal otter trawls: Effects of net height, tow duration and diel period. Fisheries Research 93, 315–323. https://doi.org/10.1016/j.fishres.2008.05.012
687. Johnson, E.E., Medina, M.D., Hernandez, A.C.B., Kusel, G.A., Batzer, A.N., Angelini, C., 2019. Success of concrete and crab traps in facilitating Eastern oyster recruitment and reef development. Peerj 7. https://doi.org/10.7717/peerj.6488
688. Johnson, I., Hutchings, M., Benstead, R., Thain, J., Whitehouse, P., 2004. Bioassay selection, experimental design and quality control/assurance for use in effluent assessment and control. Ecotoxicology 13, 437–447. https://doi.org/10.1023/B:ECTX.0000035294.15964.9a
689. Johnson, R.K., Angeler, D.G., Moe, S.J., Hering, D., 2014. Cross-taxon responses to elevated nutrients in European streams and lakes. Aquatic Sciences 76, 51–60. https://doi.org/10.1007/s00027-013-0311-x
690. Johnston, D.H., Laugier, B.P., 2012. Resource assessment based on 4D seismic and inversion at Ringhorne Field, Norwegian North Sea. Leading Edge 31, 1042–1048. https://doi.org/10.1190/tle31091042.1
691. Johnston, G., 2009. OmanHydro symposium. Hydro International 13.
692. Johnston, P., Baruwa, S., Wyatt, R., 2015. Remote passive acoustic monitoring: Listening for whales and dolphins from the safety of land. Leading Edge 34, 1516–1519. https://doi.org/10.1190/tle34121516.1
693. Jókai, Z., Abrankó, L., Fodor, P., 2005. SPME-GC-pyrolysis-AFS determination of methylmercury in marine fish products by alkaline sample preparation and aqueous phase phenylation derivatization. Journal of Agricultural and Food Chemistry 53, 5499–5505. https://doi.org/10.1021/jf0501140
694. Jolánkai, G., Bíró, I., 2001. On the determination of water quality targets and the allowable pollution loads. Vizugyi Kozlemenyek 317–336.
695. Jones, G.E., Glegg, G.E., 2004. Effective use of geophysical sensors for marine environmental assessment and habitat mapping, Environmental Studies.
696. Jones, M.T., Galeczka, I.M., Gkritzalis-Papadopoulos, A., Palmer, M.R., Mowlem, M.C., Vogfjord, K., Jonsson, P., Gislason, S.R., 2015. Monitoring of jokulhlaups and element fluxes in proglacial Icelandic rivers using osmotic samplers. Journal of Volcanology and Geothermal Research 291, 112–124. https://doi.org/10.1016/j.jvolgeores.2014.12.018
697. Jones, M.T., Gałeczka, I.M., Gkritzalis-Papadopoulos, A., Palmer, M.R., Mowlem, M.C., Vogfjör, K., Jónsson, T., Gislason, S.R., 2015. Monitoring of jökulhlaups and element fluxes in proglacial Icelandic rivers using osmotic samplers. Journal of Volcanology and Geothermal Research 291, 112–124. https://doi.org/10.1016/j.jvolgeores.2014.12.018
698. Jones, N., Clark, J.R.A., 2014. Social capital and the public acceptability of climate change adaptation policies: A case study in Romney Marsh, UK. Climatic Change 123, 133–145. https://doi.org/10.1007/s10584-013-1049-0
699. Jones, O.A.H., Dondero, F., Viarengo, A., Griffin, J.L., 2008. Metabolic profiling of Mytilus galloprovincialis and its potential applications for pollution assessment. Marine Ecology Progress Series 369, 169–179. https://doi.org/10.3354/meps07654
700. Jou, L.J., Lin, S.C., Chen, B.C., Chen, W.Y., Liao, C.M., 2013. Synthesis and measurement of valve activities by an improved online clam-based behavioral monitoring system. Computers and Electronics in Agriculture 90, 106–118. https://doi.org/10.1016/j.compag.2012.09.008
701. Jouvet, G., Weidmann, Y., Kneib, M., Detert, M., Seguinot, J., Sakakibara, D., Sugiyama, S., 2018. Short-lived ice speed-up and plume water flow captured by a VTOL UAV give insights into subglacial hydrological system of Bowdoin Glacier. Remote Sensing of Environment 217, 389–399. https://doi.org/10.1016/j.rse.2018.08.027
702. Joynt, J.F., Williams, R., 2000. Profiling with lightweight towed bodies - some case histories. International Ocean Systems 4, 20–23.
703. Jung, H., Park, H.M., Kim, J.H., Kim, G., Kong, J.S., 2013. Development of a probabilistic life-cycle cost model for marine structures exposed to chloride attack based on Bayesian approach using monitoring data. KSCE Journal of Civil Engineering 17, 1073–1082. https://doi.org/10.1007/s12205-013-0350-9
704. Jäger, P., Pall, K., Dumfarth, E., 2004. A method of mapping macrophytes in large lakes with regard to the requirements of the Water Framework Directive. Limnologica 34, 140–146. https://doi.org/10.1016/S0075-9511(04)80033-1
705. Jönsson, M., Abrahamson, A., Brunström, B., Brandt, I., Ingebrigtsen, K., Jørgensen, E.H., 2003. EROD activity in gill filaments of anadromous and marine fish as a biomarker of dioxin-like pollutants. Comparative Biochemistry and Physiology - C Toxicology and Pharmacology 136, 235–243. https://doi.org/10.1016/j.cca.2003.09.005
706. Kaba, E., Philpot, W., Steenhuis, T., 2014. Evaluating suitability of MODIS-terra images for reproducing historic sediment concentrations in water bodies: Lake Tana, Ethiopia. International Journal of Applied Earth Observation and Geoinformation 26, 286–297. https://doi.org/10.1016/j.jag.2013.08.001
707. Kabatereine, N.B., Standley, C.J., Sousa-Figueiredo, J.C., Fleming, F.M., Stothard, J.R., Talisuna, A., Fenwick, A., 2011. Integrated prevalence mapping of schistosomiasis, soil-transmitted helminthiasis and malaria in lakeside and island communities in Lake Victoria, Uganda. Parasites and Vectors 4. https://doi.org/10.1186/1756-3305-4-232
708. Kalaji, H.M., Sytar, O., Brestic, M., Samborska, I.A., Cetner, M.D., Carpentier, C., 2016. Risk assessment of urban lake water quality based on in-situ cyanobacterial and total chlorophyll-a monitoring. Polish Journal of Environmental Studies 25, 655–661. https://doi.org/10.15244/pjoes/60895
709. Kamali, B., Hashim, R., 2010. Bamboo foundation mat for rubble mound breakwaters on mud deposits. International Journal of Physical Sciences 5, 1406–1410.
710. Kaminski, A., Bell, K.P., Noblet, C.L., Evans, K.S., 2017. An Economic Analysis of Coastal Beach Safety Information-Seeking Behavior. Agricultural and Resource Economics Review 46, 365–387. https://doi.org/10.1017/age.2017.17
711. Kang, D.H., Hyeon, J.E., You, S.K., Kim, S.W., Han, S.O., 2014. Efficient enzymatic degradation process for hydrolysis activity of the Carrageenan from red algae in marine biomass. Journal of Biotechnology 192, 108–113. https://doi.org/10.1016/j.jbiotec.2014.09.019
712. Kang, S.Y., McGree, J.M., Drovandi, C.C., Caley, M.J., Mengersen, K.L., 2016. Bayesian adaptive design: Improving the effectiveness of monitoring of the Great Barrier Reef. Ecological Applications 26, 2635–2646. https://doi.org/10.1002/eap.1409
713. Kangoye, D.T., Noor, A., Midega, J., Mwongeli, J., Mkabili, D., Mogeni, P., Kerubo, C., Akoo, P., Mwangangi, J., Drakeley, C., Marsh, K., Bejon, P., Njuguna, P., 2016. Malaria hotspots defined by clinical malaria, asymptomatic carriage, PCR and vector numbers in a low transmission area on the Kenyan Coast. Malaria Journal 15. https://doi.org/10.1186/s12936-016-1260-3
714. Kannan, N., Hong, S.H., Oh, J.R., Yim, U.H., Li, D., Shim, W.J., 2005. PYE [2-(1-pyrenyl)ethyldimethylsilylated silica] column HPLC and HR-GC-(micro) ECD in the accurate determination of toxic co-planar PCBs and polybrominated diphenyl ethers (PBDEs). Bulletin of the Korean Chemical Society 26, 529–536. https://doi.org/10.5012/bkcs.2005.26.4.529
715. Kanninen, A., Vallinkoski, V.M., Leka, J., Marjomäki, T.J., Hellsten, S., Hämäläinen, H., 2013. A comparison of two methods for surveying aquatic macrophyte communities in boreal lakes: Implications for bioassessment. Aquatic Botany 104, 88–100. https://doi.org/10.1016/j.aquabot.2012.09.002
716. Kanoute, Y.B., Gragnon, B.G., Schindler, C., Bonfoh, B., Schelling, E., 2017a. Epidemiology of brucellosis, Q Fever and Rift Valley Fever at the human and livestock interface in northern Cote d’Ivoire. Acta Tropica 165, 66–75. https://doi.org/10.1016/j.actatropica.2016.02.012
717. Kanoute, Y.B., Gragnon, B.G., Schindler, C., Bonfoh, B., Schelling, E., 2017b. Reprint of “Epidemiology of brucellosis, Q Fever and Rift Valley Fever at the human and livestock interface in northern Cote d’Ivoire.” Acta Tropica 175, 121–129. https://doi.org/10.1016/j.actatropica.2017.08.013
718. Karagiorgas, M., Tsoutsos, T., Moiá-Pol, A., 2007. A simulation of the energy consumption monitoring in Mediterranean hotels. Application in Greece. Energy and Buildings 39, 416–426. https://doi.org/10.1016/j.enbuild.2006.07.008
719. Karamfilov, V., Berov, D., Panayotidis, P., 2019. Using Zostera noltei biometrics for evaluation of the ecological and environmental quality status of Black Sea coastal waters. Regional Studies in Marine Science 27. https://doi.org/10.1016/j.rsma.2019.100524
720. Karatayev, A.Y., Mehler, K., Burlakova, L.E., Hinchey, E.K., Warren, G.J., 2018. Benthic video image analysis facilitates monitoring of Dreissena populations across spatial scales. Journal of Great Lakes Research 44, 629–638. https://doi.org/10.1016/j.jglr.2018.05.003
721. Karczewski, K., Riss, H.W., Meyer, E.I., 2017. Comparison of DNA-fingerprinting (T-RFLP) and high-throughput sequencing (HTS) to assess the diversity and composition of microbial communities in groundwater ecosystems. Limnologica 67, 45–53. https://doi.org/10.1016/j.limno.2017.10.001
722. Kari, E., Kratzer, S., Beltrán-Abaunza, J.M., Harvey, E.T., Vaičiūtė, D., 2017. Retrieval of suspended particulate matter from turbidity–model development, validation, and application to MERIS data over the Baltic Sea. International Journal of Remote Sensing 38, 1983–2003. https://doi.org/10.1080/01431161.2016.1230289
723. Karim, M., Maanan, M., Maanan, M., Rhinane, H., Rueff, H., Baidder, L., 2019. Assessment of water body change and sedimentation rate in Moulay Bousselham wetland, Morocco, using geospatial technologies. International Journal of Sediment Research 34, 65–72. https://doi.org/10.1016/j.ijsrc.2018.08.007
724. Karimanzira, D., Jacobi, M., Pfuetzenreuter, T., Rauschenbach, T., Eichhorn, M., Taubert, R., Ament, C., 2014. First testing of an AUV mission planning and guidance system for water quality monitoring and fish behavior observation in net cage fish farming. Information Processing in Agriculture 1, 131–140. https://doi.org/10.1016/j.inpa.2014.12.001
725. Karkare, S., Bhatnagar, D., 2006. Promising nucleic acid analogs and mimics: Characteristic features and applications of PNA, LNA, and morpholino. Applied Microbiology and Biotechnology 71, 575–586. https://doi.org/10.1007/s00253-006-0434-2
726. Karki, R., Tagert, M.L.M., Paz, J.O., 2018. Evaluating the nutrient reduction and water supply benefits of an on-farm water storage (OFWS) system in East Mississippi. Agriculture Ecosystems & Environment 265, 476–487. https://doi.org/10.1016/j.agee.2018.06.024
727. Kartal, B., Van Niftrik, L., Sliekers, O., Schmid, M.C., Schmidt, I., Van De Pas-Schoonen, K., Cirpus, I., Van Der Star, W., Van Loosdrecht, M., Abma, W., Kuenen, J.G., Mulder, J.W., Jetten, M.S.M., Op Den Camp, H., Strous, M., Van De Vossenberg, J., 2004. Application, eco-physiology and biodiversity of anaerobic ammonium-oxidizing bacteria. Reviews in Environmental Science and Biotechnology 3, 255–264. https://doi.org/10.1007/s11157-004-7247-5
728. Karunanidhi, D., Vennila, G., Suresh, M., Karthikeyan, P., 2014. Geoelectrical Schlumberger investigation for characterizing the hydrogeological conditions using GIS in Omalur Taluk, Salem District, Tamil Nadu, India. Arabian Journal of Geosciences 7, 1791–1798. https://doi.org/10.1007/s12517-013-0881-x
729. Katip, A., 2018. THE USAGE OF ARTIFICIAL NEURAL NETWORKS IN MICROBIAL WATER QUALITY MODELING: A CASE STUDY FROM THE LAKE IZNIK. Applied Ecology and Environmental Research 16, 3897–3917. https://doi.org/10.15666/aeer/1604_38973917
730. Katip, A., 2018. The usage of artificial neural networks in microbial water quality modeling: A case study from the Lake İznİk. Applied Ecology and Environmental Research 16, 3897–3917. https://doi.org/10.15666/aeer/1604_38973917
731. Kay, B.H., Ryan, P.A., Russell, B.M., Holt, J.S., Lyons, S.A., Foley, P.N., 2000. The importance of subterranean mosquito habitat to arbovirus vector control strategies in north Queensland Australia. Journal of Medical Entomology 37, 846–853. https://doi.org/10.1603/0022-2585-37.6.846
732. Keck, F., Vasselon, V., Tapolczai, K., Rimet, F., Bouchez, A., 2017. Freshwater biomonitoring in the Information Age. Frontiers in Ecology and the Environment 15, 266–274. https://doi.org/10.1002/fee.1490
733. Kedzierski, M., Le Tilly, V., Cesar, G., Sire, O., Bruzaud, S., 2017. Efficient microplastics extraction from sand. A cost effective methodology based on sodium iodide recycling. Marine Pollution Bulletin 115, 120–129. https://doi.org/10.1016/j.marpolbul.2016.12.002
734. Keesstra, S., Nunes, J., Novara, A., Finger, D., Avelar, D., Kalantari, Z., Cerdà, A., 2018. The superior effect of nature based solutions in land management for enhancing ecosystem services. Science of the Total Environment 610–611, 997–1009. https://doi.org/10.1016/j.scitotenv.2017.08.077
735. Keipert, N., Weaver, D., Summers, R., Clarke, M., Neville, S., 2008. Guiding BMP adoption to improve water quality in various estuarine ecosystems in Western Australia, Water Science and Technology. https://doi.org/10.2166/wst.2008.276
736. Keizer-Vlek, H.E., Verdonschot, P.F.M., Verdonschot, R.C.M., Goedhart, P.W., 2012. Quantifying spatial and temporal variability of macroinvertebrate metrics. Ecological Indicators 23, 384–393. https://doi.org/10.1016/j.ecolind.2012.04.025
737. Keller, K., Steffe, A.S., Lowry, M., Murphy, J.J., Suthers, I.M., 2016. Monitoring boat-based recreational fishing effort at a nearshore artificial reef with a shore-based camera. Fisheries Research 181, 84–92. https://doi.org/10.1016/j.fishres.2016.03.025
738. Keller, K., Steffe, A.S., Lowry, M.B., Murphy, J.J., Smith, J.A., Suthers, I.M., 2017. Estimating the recreational harvest of fish from a nearshore designed artificial reef using a pragmatic approach. Fisheries Research 187, 158–167. https://doi.org/10.1016/j.fishres.2016.11.022
739. Kennedy, G., Mayer, T., 2002. Natural and constructed wetlands in Canada: An overview. Water Quality Research Journal of Canada 37, 295–325.
740. Kennedy, K., Schroeder, T., Shaw, M., Haynes, D., Lewis, S., Bentley, C., Paxman, C., Carter, S., Brando, V.E., Bartkow, M., Hearn, L., Mueller, J.F., 2012. Long term monitoring of photosystem II herbicides - Correlation with remotely sensed freshwater extent to monitor changes in the quality of water entering the Great Barrier Reef, Australia. Marine Pollution Bulletin 65, 292–305. https://doi.org/10.1016/j.marpolbul.2011.10.029
741. Keskin, E., Unal, E.M., Atar, H.H., 2016. Detection of rare and invasive freshwater fish species using eDNA pyrosequencirig: Lake Iznik ichthyofauna revised. Biochemical Systematics and Ecology 67, 29–36. https://doi.org/10.1016/j.bse.2016.05.020
742. Keskin, E., Unal, E.M., Atar, H.H., 2016. Detection of rare and invasive freshwater fish species using eDNA pyrosequencing: Lake Iznik ichthyofauna revised. Biochemical Systematics and Ecology 67, 29–36. https://doi.org/10.1016/j.bse.2016.05.020
743. Khalililaghab, S., Momeni, S., Farrokhnia, M., Nabipour, I., Karimi, S., 2017. Development of a new colorimetric assay for detection of bisphenol-A in aqueous media using green synthesized silver chloride nanoparticles: experimental and theoretical study. Analytical and Bioanalytical Chemistry 409, 2847–2858. https://doi.org/10.1007/s00216-017-0230-0
744. Khan, A., Amelie, V., 2015. Assessing climate change readiness in Seychelles: implications for ecosystem-based adaptation mainstreaming and marine spatial planning. Regional Environmental Change 15, 721–733. https://doi.org/10.1007/s10113-014-0662-4
745. Khan, F.A., Puls, R.W., 2002. In situ abiotic detoxification and immobilization of hexavalent chromium. Ground Water Monitoring and Remediation 23, 77–84. https://doi.org/10.1111/j.1745-6592.2003.tb00785.x
746. Khan, S.A., Rahman, R., Er Rashid, H., Rashid, S., 2010. Socio-economic effects of Tsunami on Bangladesh, in: Wind Storm and Storm Surge Mitigation. pp. 140–152. https://doi.org/10.1061/9780784410813.ch12
747. Khandeparker, L., Anil, A.C., 2016. Global concerns of ship’s ballast water mediated translocation of bacteria, in: Marine Pollution and Microbial Remediation. pp. 255–262. https://doi.org/10.1007/978-981-10-1044-6_16
748. Khanna, S., Santos, M.J., Ustin, S.L., Shapiro, K., Haverkamp, P.J., Lay, M., 2018. Comparing the potential of multispectral and hyperspectral data for monitoring oil spill impact. Sensors (Switzerland) 18. https://doi.org/10.3390/s18020558
749. Kim, D.J., Jung, J., Kang, K.M., Kim, S.H., Xu, Z., Hensley, S., Swan, A., Duersch, M., 2015. Development of a cost-effective airborne remote sensing system for coastal monitoring. Sensors (Switzerland) 15, 25366–25384. https://doi.org/10.3390/s151025366
750. Kim, M., Yim, U.H., Hong, S.H., Jung, J.H., Choi, H.W., An, J., Won, J., Shim, W.J., 2010. Hebei Spirit oil spill monitored on site by fluorometric detection of residual oil in coastal waters off Taean, Korea. Marine Pollution Bulletin 60, 383–389. https://doi.org/10.1016/j.marpolbul.2009.10.015
751. Kinds, A., Sys, K., Schotte, L., Mondelaers, K., Polet, H., 2015. VALDUVIS: An innovative approach to assess the sustainability of fishing activities. Fisheries Research 182, 158–171. https://doi.org/10.1016/j.fishres.2015.10.027
752. King, J., Essink, G.O., Karaolis, M., Siemon, B., Bierkens, M.F.P., 2018. Quantifying Geophysical Inversion Uncertainty Using Airborne Frequency Domain Electromagnetic Data-Applied at the Province of Zeeland, the Netherlands. Water Resources Research 54, 8420–8441. https://doi.org/10.1029/2018wr023165
753. Kingsford, R.T., Porter, J.L., 2009. Monitoring waterbird populations with aerial surveys - What have we learnt? Wildlife Research 36, 29–40. https://doi.org/10.1071/WR08034
754. Kinsey, J.C., Tivey, M.A., Yoerger, D.R., 2013. Dynamics and navigation of autonomous underwater vehicles for submarine gravity surveying. GEOPHYSICS 78, G55–G68. https://doi.org/10.1190/geo2012-0181.1
755. Kinzelman, J.L., Dufour, A.P., Wymer, L.J., Rees, G., Pond, K.R., Bagley, R.C., 2006. Comparison of multiple point and composite sampling for monitoring bathing water quality. Lake and Reservoir Management 22, 95–102. https://doi.org/10.1080/07438140609353887
756. Kirby, D.S., Ward, P., 2014. Standards for the effective management of fisheries bycatch. Marine Policy 44, 419–426. https://doi.org/10.1016/j.marpol.2013.10.008
757. Kirby, M.F., Gioia, R., Law, R.J., 2014. The principles of effective post-spill environmental monitoring in marine environments and their application to preparedness assessment. Marine Pollution Bulletin 82, 11–18. https://doi.org/10.1016/j.marpolbul.2014.01.038
758. Kišević, M., Smailbegović, A., Gray, K.T., Andričević, R., Craft, J.D., Petrov, V., Brajčić, D., Dragičević, I., 2011. Spectral reflectance profile of Caulerpa racemosa var. Cylindracea and Caulerpa taxifolia in the Adriatic Sea. Acta Adriatica 52, 21–28.
759. Kissinger, L., Lorenzana, R., Mittl, B., Lasrado, M., Iwenofu, S., Olivo, V., Helba, C., Capoeman, P., Williams, A.H., 2010. Development of a Computer-Assisted Personal Interview Software System for Collection of Tribal Fish Consumption Data. Risk Analysis 30, 1833–1841. https://doi.org/10.1111/j.1539-6924.2010.01461.x
760. Klanova, J., Harner, T., 2013. The challenge of producing reliable results under highly variable conditions and the role of passive air samplers in the Global Monitoring Plan. Trac-Trends in Analytical Chemistry 46, 139–149. https://doi.org/10.1016/j.trac.2012.07.021
761. Klečka, J., Boukal, D.S., 2011. Lazy ecologist’s guide to water beetle diversity: Which sampling methods are the best? Ecological Indicators 11, 500–508. https://doi.org/10.1016/j.ecolind.2010.07.005
762. Klein, H.U., Ovsyshcher, I.E., 2005. The 7th international Dead Sea symposium on cardiac arrhythmias and device therapy. Europace 7, 407–408. https://doi.org/10.1016/j.eupc.2005.03.006
763. Kleivdal, H., Kristiansen, S.I., Nilsen, M.V., Goksøyr, A., Briggs, L., Holland, P., McNabb, P., 2007. Determination of domoic acid toxins in shellfish by biosense ASP ELISA - A direct competitive enzyme-linked immunosorbent assay: Collaborative study. Journal of AOAC International 90, 1011–1027.
764. Klemas, V., 2011. Remote sensing of wetlands: Case studies comparing practical techniques. Journal of Coastal Research 27, 418–427. https://doi.org/10.2112/JCOASTRES-D-10-00174.1
765. Klemas, V., 2013. Remote Sensing of Coastal Wetland Biomass: An Overview. Journal of Coastal Research 29, 1016–1028. https://doi.org/10.2112/jcoastres-d-12-00237.1
766. Klemas, V., 2013a. Remote sensing of emergent and submerged wetlands: an overview. International Journal of Remote Sensing 34, 6286–6320. https://doi.org/10.1080/01431161.2013.800656
767. Klemas, V., 2013b. Using remote sensing to select and monitor wetland restoration sites: An overview. Journal of Coastal Research 29, 958–970. https://doi.org/10.2112/JCOASTRES-D-12-00170.1
768. Klemas, V., 2014. Remote Sensing of Riparian and Wetland Buffers: An Overview. Journal of Coastal Research 30, 869–880. https://doi.org/10.2112/jcoastres-d-14-00013.1
769. Klemas, V., Davis, G., Wang, H., Whelan, W., Tornatore, G., 2017. MONITORING ESTUARINE CIRCULATION AND OCEAN WASTE DISPERSION USING AN INTEGRATED SATELLITE-AIRCRAFT-DROGUE APPROACH 1.
770. Klinck, H., Mellinger, D.K., Klinck, K., Bogue, N.M., Luby, J.C., Jump, W.A., Shilling, G.B., Litchendorf, T., Wood, A.S., Schorr, G.S., Baird, R.W., 2012. Near-Real-Time Acoustic Monitoring of Beaked Whales and Other Cetaceans Using a Seaglider (TM). Plos One 7. https://doi.org/10.1371/journal.pone.0036128
771. Klinck, H., Mellinger, D.K., Klinck, K., Bogue, N.M., Luby, J.C., Jump, W.A., Shilling, G.B., Litchendorf, T., Wood, A.S., Schorr, G.S., Baird, R.W., 2012. Near-real-time acoustic monitoring of beaked whales and other cetaceans using a Seaglider^TM^. PLoS ONE 7. https://doi.org/10.1371/journal.pone.0036128
772. Kloser, R.J., Ryan, T.E., Macaulay, G.J., Lewis, M.E., 2011. In situ measurements of target strength with optical and model verification: A case study for blue grenadier, Macruronus novaezelandiae. ICES Journal of Marine Science 68, 1986–1995. https://doi.org/10.1093/icesjms/fsr127
773. Knight-Jones, T.J.D., Hauser, R., Matthes, D., Stärk, K.D.C., 2010. Evaluation of effectiveness and efficiency of wild bird surveillance for avian influenza. Veterinary Research 41. https://doi.org/10.1051/vetres/2010023
774. Knol, M., 2011. The uncertainties of precaution: Zero discharges in the Barents Sea. Marine Policy 35, 399–404. https://doi.org/10.1016/j.marpol.2010.10.018
775. Knowlton, N., Brainard, R.E., Fisher, R., Moews, M., Plaisance, L., Caley, M.J., 2010. Coral Reef Biodiversity, in: Life in the World’s Oceans: Diversity, Distribution, and Abundance. pp. 65–78. https://doi.org/10.1002/9781444325508.ch4
776. Knox, S.H., Dronova, I., Sturtevant, C., Oikawa, P.Y., Matthes, J.H., Verfaillie, J., Baldocchi, D., 2017. Using digital camera and Landsat imagery with eddy covariance data to model gross primary production in restored wetlands. Agricultural and Forest Meteorology 237–238, 233–245. https://doi.org/10.1016/j.agrformet.2017.02.020
777. Knutsen, J.A., Knutsen, H., Rinde, E., Christie, H., Bodvin, T., Dahl, E., 2010. Mapping biological resources in the coastal zone: An evaluation of methods in a pioneering study from Norway. Ambio 39, 148–158. https://doi.org/10.1007/s13280-010-0023-6
778. Kobryn, H.T., Wouters, K., Beckley, L.E., Heege, T., 2013. Ningaloo Reef: Shallow Marine Habitats Mapped Using a Hyperspectral Sensor. PLoS ONE 8. https://doi.org/10.1371/journal.pone.0070105
779. Koch, M., Koebsch, F., Hahn, J., Jurasinski, G., 2017. From meadow to shallow lake: Monitoring secondary succession in a coastal fen after rewetting by flooding based on aerial imagery and plot data. Mires and Peat 19. https://doi.org/10.19189/MaP.2015.OMB.188
780. Koenig, C.C., Stallings, C.D., 2015. A new compact rotating video system for rapid survey of reef fish populations. Bulletin of Marine Science 91, 365–373. https://doi.org/10.5343/bms.2015.1010
781. Koenig, S., Savage, C., Kim, J.P., 2008. Non-destructive assessment of polycyclic aromatic hydrocarbon (PAH) exposure by fluorimetric analysis of crab urine. Marine Pollution Bulletin 56, 2003–2008. https://doi.org/10.1016/j.marpolbul.2008.08.010
782. Koenigs, R.P., Bruch, R.M., Reiter, D., Pyatskowit, J., 2019. Restoration of naturally reproducing and resident riverine lake sturgeon populations through capture and transfer. Journal of Applied Ichthyology 35, 160–168. https://doi.org/10.1111/jai.13605
783. Kohnen, C., 2003. SEAmobile submersibles: A new tool for the subsea industry. Sea Technology 44, 15–21.
784. Kolukirik, M., Ince, O., Ince, B.K., 2011. Increment in Anaerobic Hydrocarbon Degradation Activity of Halic Bay Sediments via Nutrient Amendment. Microbial Ecology 61, 871–884. https://doi.org/10.1007/s00248-011-9825-8
785. Kong, R.Y.C., Lee, S.K.Y., Law, T.W.F., Law, S.H.W., Wu, R.S.S., 2002. Rapid detection of six types of bacterial pathogens in marine waters by multiplex PCR. Water Research 36, 2802–2812. https://doi.org/10.1016/S0043-1354(01)00503-6
786. Kong, R.Y.C., Mak, M.M.H., Wu, R.S.S., 2009. DNA technologies for monitoring waterborne pathogens: A revolution in water pollution monitoring. Ocean and Coastal Management 52, 355–358. https://doi.org/10.1016/j.ocecoaman.2009.04.011
787. Kong, X., Squire, K., Li, E., Leduff, P., Rorrer, G.L., Tang, S., Chen, B., McKay, C.P., Navarro-Gonzalez, R., Wang, A.X., 2016. Chemical and biological sensing using diatom photonic crystal biosilica with in-situ growth plasmonic nanoparticles. IEEE Transactions on Nanobioscience 15, 828–834. https://doi.org/10.1109/TNB.2016.2636869
788. Konovalenko, L., Bradshaw, C., Andersson, E., Lindqvist, D., Kautsky, U., 2016. Evaluation of factors influencing accumulation of stable Sr and Cs in lake and coastal fish. Journal of Environmental Radioactivity 160, 64–79. https://doi.org/10.1016/j.jenvrad.2016.04.022
789. Kopf, A., Freudenthal, T., Ratmeyer, V., Bergenthal, M., Lange, M., Fleischmann, T., Hammerschmidt, S., Seiter, C., Wefer, G., 2015. Simple, affordable, and sustainable borehole observatories for complex monitoring objectives. Geoscientific Instrumentation, Methods and Data Systems 4, 99–109. https://doi.org/10.5194/gi-4-99-2015
790. Kopf, A., Stark, N., Hanff, H., 2009. Nimrod: A tool for rapid geotechnical characterization of surface sediments. Sea Technology 50, 10–14.
791. Koskiaho, J., Tattari, S., Röman, E., 2015. Suspended solids and total phosphorus loads and their spatial differences in a lake-rich river basin as determined by automatic monitoring network. Environmental Monitoring and Assessment 187. https://doi.org/10.1007/s10661-015-4397-6
792. Kotilainen, A.T., Kaskela, A.M., 2017. Comparison of airborne LiDAR and shipboard acoustic data in complex shallow water environments: Filling in the white ribbon zone. Marine Geology 385, 250–259. https://doi.org/10.1016/j.margeo.2017.02.005
793. Koukounari, A., Donnelly, C.A., Moustaki, I., Tukahebwa, E.M., Kabatereine, N.B., Wilson, S., Webster, J.P., Deelder, A.M., Vennervald, B.J., van Dam, G.J., 2013. A Latent Markov Modelling Approach to the Evaluation of Circulating Cathodic Antigen Strips for Schistosomiasis Diagnosis Pre- and Post-Praziquantel Treatment in Uganda. Plos Computational Biology 9. https://doi.org/10.1371/journal.pcbi.1003402
794. Koundouri, P., Scarpa, R., Stithou, M., 2014. A Choice Experiment for the Estimation of the Economic Value of the River Ecosystem: Management Policies for Sustaining NATURA (2000) Species and the Coastal Environment, Global Issues in Water Policy. https://doi.org/10.1007/978-94-007-7636-4_6
795. Kovács, J., Kovács, S., Hatvani, I.G., Magyar, N., Tanos, P., Korponai, J., Blaschke, A.P., 2015. Spatial optimization of monitoring networks on the examples of a river, a Lake-Wetland system and a Sub-Surface water system. Water Resources Management 29, 5275–5294. https://doi.org/10.1007/s11269-015-1117-5
796. Kowalsky, M.B., Nakagawa, S., Moridis, G.J., 2010. Feasibility of Monitoring Gas-Hydrate Production With Time-Lapse Vertical Seismic Profiling. Spe Journal 15, 634–645. https://doi.org/10.2118/132508-pa
797. Koydemir, H.C., Feng, S., Liang, K., Nadkarni, R., Benien, P., Ozcan, A., 2017. Comparison of supervised machine learning algorithms for waterborne pathogen detection using mobile phone fluorescence microscopy. Nanophotonics 6, 731–741. https://doi.org/10.1515/nanoph-2017-0001
798. Kozma Törökné, A., László, E., Chorus, I., Fastner, J., Heinze, R., Padisák, J., Barbosa, F.A., 2000. Water quality monitoring by Thamnotoxkit F(TM) including cyanobacterial blooms, Water Science and Technology.
799. Kramer, D.B., Polasky, S., Starfield, A., Palik, B., Westphal, L., Snyder, S., Jakes, P., Hudson, R., Gustafson, E., 2006. A comparison of alternative strategies for cost-effective water quality management in lakes. Environmental Management 38, 411–425. https://doi.org/10.1007/s00267-005-0011-y
800. Krantzberg, G., Hartig, J.H., Zarull, M.A., 2000. Sediment management: Deciding when to intervene. Environmental Science and Technology 34, 22A-27A.
801. Krapivin, V.F., Varotsos, C.A., Nghia, B.Q., 2017. A Modeling System for Monitoring Water Quality in Lagoons. Water, Air, and Soil Pollution 228. https://doi.org/10.1007/s11270-017-3581-4
802. Kroll, A.J., Hayes, M.P., MacCracken, J.G., 2009. Concerns regarding the use of amphibians as metrics of critical biological thresholds: a comment on Welsh & Hodgson (2008). Freshwater Biology 54, 2364–2373. https://doi.org/10.1111/j.1365-2427.2009.02245.x
803. Krstić, S., Svirčev, Z., Levkov, Z., Nakov, T., 2007. Selecting appropriate bioindicators regarding Water Framework Directive guidelines for freshwaters - A Macedonian experience. International Journal on Algae 9, 41–63. https://doi.org/10.1615/InterJAlgae.v9.i1.30
804. Kudom, A.A., 2015. Larval ecology of Anopheles coluzzii in Cape Coast, Ghana: Water quality, nature of habitat and implication for larval control. Malaria Journal 14. https://doi.org/10.1186/s12936-015-0989-4
805. Kusche, H., Hillgruber, N., Rossner, Y., Focken, U., 2018. The effect of different fish feed compositions on delta C-13 and delta N-15 signatures of sea bass and its potential value for tracking mariculture-derived nutrients. Isotopes in Environmental and Health Studies 54, 28–40. https://doi.org/10.1080/10256016.2017.1361419
806. Kusche, H., Hillgruber, N., Rößner, Y., Focken, U., 2018. The effect of different fish feed compositions on δ13C and δ15N signatures of sea bass and its potential value for tracking mariculture-derived nutrients. Isotopes in Environmental and Health Studies 54, 28–40. https://doi.org/10.1080/10256016.2017.1361419
807. Kuyper, B., Labuschagne, C., Philibert, R., Moyo, N., Waldron, H., Reason, C., Palmer, C., 2012. Development of a simplified, cost effective GC-ECD methodology for the sensitive detection of bromoform in the troposphere. Sensors (Switzerland) 12, 13583–13597. https://doi.org/10.3390/s121013583
808. Kuzukiran, O., Yurdakok-Dikmen, B., Totan, F.E., Celik, C., Orhan, E.C., Bilir, E.K., Kara, E., Filazi, A., 2016. Analytical method development and validation for some persistent organic pollutants in water and sediments by gas chromatography mass spectrometry. International Journal of Environmental Research 10, 401–410.
809. Kvernevik, T.I., Zambri Mohd Akhir, M., Studholme, J., 2002. A low-cost procedure for automatic seafloor mapping, with particular reference to coral reef conservation in developing nations. Hydrobiologia 474, 67–79. https://doi.org/10.1023/A:1016508923403
810. LaCommare, K.S., Brault, S., Self-Sullivan, C., Hines, E.M., 2012. Trend detection in a boat-based method for monitoring sirenians: Antillean manatee case study. Biological Conservation 152, 169–177. https://doi.org/10.1016/j.biocon.2012.02.021
811. Laffaille, P., Briand, C., Fatin, D., Lafage, D., Lasne, E., 2005. Point sampling the abundance of European eel (Anguilla anguilla) in freshwater areas. Archiv fur Hydrobiologie 162, 91–98. https://doi.org/10.1127/0003-9136/2005/0162-0091
812. Laforge, M.P., Clark, D.A., Schmidt, A.L., Lankshear, J.L., Kowalchuk, S., Brook, R.K., 2017. Temporal aspects of polar bear (Ursus maritimus) occurrences at field camps in Wapusk National Park, Canada. Polar Biology 40, 1661–1670. https://doi.org/10.1007/s00300-017-2091-6
813. Lagarde, F., Jaffrezic-Renault, N., 2011. Cell-based electrochemical biosensors for water quality assessment. Analytical and Bioanalytical Chemistry 400, 947–964. https://doi.org/10.1007/s00216-011-4816-7
814. Lago, M., Boteler, B., Rouillard, J., Abhold, K., Jähnig, S.C., Iglesias-Campos, A., Delacámara, G., Piet, G.J., Hein, T., Nogueira, A.J.A., Lillebø, A.I., Strosser, P., Robinson, L.A., De Wever, A., O’Higgins, T., Schlüter, M., Török, L., Reichert, P., van Ham, C., Villa, F., Hugh, M., 2019. Introducing the H2020 AQUACROSS project: Knowledge, Assessment, and Management for AQUAtic Biodiversity and Ecosystem Services aCROSS EU policies. Science of the Total Environment 652, 320–329. https://doi.org/10.1016/j.scitotenv.2018.10.076
815. Lampadariou, N., Karakassis, I., Pearson, T.H., 2005. Cost/benefit analysis of a benthic monitoring programme of organic benthic enrichment using different sampling and analysis methods. Marine Pollution Bulletin 50, 1606–1618. https://doi.org/10.1016/j.marpolbul.2005.06.030
816. Land, L.S., 2012. Chesapeake Bay nutrient pollution: Contribution from the land application of sewage sludge in Virginia. Marine Pollution Bulletin 64, 2305–2308. https://doi.org/10.1016/j.marpolbul.2012.07.003
817. Landrø, M., Hansteen, F., Amundsen, L., 2017. Detecting gas leakage using high-frequency signals generated by air-gun arrays. Geophysics 82, A7–A12. https://doi.org/10.1190/GEO2016-0483.1
818. Langford, K.H., Øxnevad, S., Schøyen, M., Thomas, K.V., 2014. Do antiparasitic medicines used in aquaculture pose a risk to the Norwegian aquatic environment? Environmental Science and Technology 48, 7774–7780. https://doi.org/10.1021/es5005329
819. Langlois, T.J., Bellchambers, L.M., Fisher, R., Shiell, G.R., Goetze, J., Fullwood, L., Evans, S.N., Konzewitsch, N., Harvey, E.S., Pember, M.B., 2017. Investigating ecosystem processes using targeted fisheries closures: Can small-bodied invertivore fish be used as indicators for the effects of western rock lobster fishing? Marine and Freshwater Research 68, 1251–1259. https://doi.org/10.1071/MF16022
820. Langridge, R.M., Ries, W.F., Farrier, T., Barth, N.C., Khajavi, N., De Pascale, G.P., 2014. Developing sub 5-m LiDAR DEMs for forested sections of the Alpine and Hope faults, South Island, New Zealand: Implications for structural interpretations. Journal of Structural Geology 64, 53–66. https://doi.org/10.1016/j.jsg.2013.11.007
821. Lanzén, A., Lekang, K., Jonassen, I., Thompson, E.M., Troedsson, C., 2017. DNA extraction replicates improve diversity and compositional dissimilarity in metabarcoding of eukaryotes in marine sediments. PLoS ONE 12. https://doi.org/10.1371/journal.pone.0179443
822. Lanzén, A., Lekang, K., Jonassen, I., Thompson, E.M., Troedsson, C., 2016. High-throughput metabarcoding of eukaryotic diversity for environmental monitoring of offshore oil-drilling activities. Molecular ecology 25, 4392–4406. https://doi.org/10.1111/mec.13761
823. Lappalainen, A., Saks, L., Sustar, M., Heikinheimo, O., Jurgens, K., Kokkonen, E., Kurkilahti, M., Verliin, A., Vetemaa, M., 2016. Length at maturity as a potential indicator of fishing pressure effects on coastal pikeperch (Sander lucioperca) stocks in the northern Baltic Sea. Fisheries Research 174, 47–57. https://doi.org/10.1016/j.fishres.2015.08.013
824. Laran, S., Authier, M., Canneyt, O.V., Dorémus, G., Watremez, P., Ridoux, V., 2017. A comprehensive survey of pelagic megafauna: Their distribution, densities, and taxonomic richness in the tropical Southwest Indian ocean. Frontiers in Marine Science 4. https://doi.org/10.3389/fmars.2017.00139
825. Larsen, P.F., Barker, S., Wright, J., Erickson, C.B., 2004. Use of cost effective remote sensing to map and measure marine intertidal habitats in support of ecosystem modeling efforts: Cobscook Bay, Maine. Northeastern Naturalist 11, 225–242. https://doi.org/10.1656/1092-6194(2004)11[225:uocers]2.0.co;2
826. Latest technologies benefit old engines, 2001. . Shiprepair and Conversion Technology 32–35.
827. Latif, M., Licek, E., 2004. Toxicity assessment of wastewaters, river waters, and sediments in Austria using cost-effective microbiotests. Environmental Toxicology 19, 302–309. https://doi.org/10.1002/tox.20027
828. Latini, A.O., Petrere Júnior, M., 2018. Efficiency of rapid field methods for detecting non-native fish in Eastern Brazilian lakes. Hydrobiologia 817, 85–96. https://doi.org/10.1007/s10750-018-3624-x
829. Laufer, G., Gobel, N., Borteiro, C., Soutullo, A., Martínez-Debat, C., de Sá, R.O., 2018. Current status of American bullfrog, Lithobates catesbeianus, invasion in Uruguay and exploration of chytrid infection. Biological Invasions 20, 285–291. https://doi.org/10.1007/s10530-017-1540-z
830. Law, A.Y.S., Wei, X., Zhang, X., Mak, N.K., Cheung, K.C., Wong, M.H., Giesy, J.P., Wong, C.K.C., 2012. Biological analysis of endocrine-disrupting chemicals in animal meats from the Pearl River Delta, China. Journal of Exposure Science and Environmental Epidemiology 22, 93–100. https://doi.org/10.1038/jes.2011.36
831. Le Duc, M.G., Pakeman, R.J., Marrs, R.H., 2000. Vegetation development on upland and marginal land treated with herbicide, for bracken (Pteridium aquilinum) control, in Great Britain. Journal of Environmental Management 58, 147–160. https://doi.org/10.1006/jema.1999.0321
832. Le Maitre, D.C., Versfeld, D.B., Chapman, R.A., 2000. The impact of invading alien plants on surface water resources in South Africa: A preliminary assessment. Water SA 26, 397–408.
833. Le Moullec, M., Pedersen, A.O., Yoccoz, N.G., Aanes, R., Tufto, J., Hansen, B.B., 2017. Ungulate population monitoring in an open tundra landscape: Distance sampling versus total counts. Wildlife Biology 2017. https://doi.org/10.2981/wlb.00299
834. Le Reste, S., Dutreuil, V., André, X., Thierry, V., Renaut, C., Le Traon, P.Y., Maze, G., 2016. “Deep-Arvor”: A new profiling float to extend the argo observations down to 4000-m depth. Journal of Atmospheric and Oceanic Technology 33, 1039–1055. https://doi.org/10.1175/JTECH-D-15-0214.1
835. Le, T.H.G., 2019. On the use of amour block-RAKUNA IV in breakwaters and coastal protection works in Vietnam, Lecture Notes in Civil Engineering. https://doi.org/10.1007/978-981-13-2306-5_21
836. Le, T.M.H., Eiksund, G.R., Strøm, P.J., Saue, M., 2014. Geological and geotechnical characterisation for offshore wind turbine foundations: A case study of the Sheringham Shoal wind farm. Engineering Geology 177, 40–53. https://doi.org/10.1016/j.enggeo.2014.05.005
837. Leach, J.H.J., 2006. The korrong project–semi-submersible imaging for environmental mapping in shallow water. Journal of Spatial Science 51, 133–142. https://doi.org/10.1080/14498596.2006.9635069
838. League, M.T., Seliskar, D.M., Gallagher, J.L., 2007. Predicting the effectiveness of Phragmites control measures using a rhizome growth potential bioassay. Wetlands Ecology and Management 15, 27–41. https://doi.org/10.1007/s11273-006-9009-3
839. Leaman, B.M., Williams, G.H., 2004. Collaborative Pacific halibut, Hippoglossus stenolepis, bycatch control by Canada and the United States. Marine Fisheries Review 66, 31–37.
840. Leaper, R., Burt, L., Gillespie, D., Macleod, K., 2010. Comparisons of measured and estimated distances and angles from sightings surveys. Journal of Cetacean Research and Management 11, 229–237.
841. Leckie, D.G., Jay, C., Gougeon, F.A., Sturrock, R.N., Paradine, D., 2004. Detection and assessment of trees with Phellinus weirii (laminated root rot) using high resolution multi-spectral imagery. International Journal of Remote Sensing 25, 793–818. https://doi.org/10.1080/0143116031000139926
842. Lecklin, T., Ryömä, R., Kuikka, S., 2011. A Bayesian network for analyzing biological acute and long-term impacts of an oil spill in the Gulf of Finland. Marine Pollution Bulletin 62, 2822–2835. https://doi.org/10.1016/j.marpolbul.2011.08.045
843. Lee, C.S., Dong Park, J., Shin, J., Jang, J.D., 2017. Improvement of AMSR2 Soil Moisture Products over South Korea. IEEE Journal of Selected Topics in Applied Earth Observations and Remote Sensing 10, 3839–3849. https://doi.org/10.1109/JSTARS.2017.2723923
844. Lee, E., Kang, K.M., Hyun, S.P., Lee, K.Y., Yoon, H., Kim, S.H., Kim, Y., Xu, Z., Kim, D.J., Koh, D.C., Ha, K., 2016. Submarine groundwater discharge revealed by aerial thermal infrared imagery: a case study on Jeju Island, Korea. Hydrological Processes 30, 3494–3506. https://doi.org/10.1002/hyp.10868
845. Lee, H.Y., Park, K.P., Koo, N.H., Yoo, D.G., Kang, D.H., Kim, Y.G., Hwang, K.D., Kim, J.C., 2004. High-resolution shallow marine seismic surveys off Busan and Pohang, Korea, using a small-scale multichannel system. Journal of Applied Geophysics 56, 1–15. https://doi.org/10.1016/j.jappgeo.2004.03.003
846. Lee, I.C., 2016. Instantaneous shoreline mapping from Worldview-2 satellite images by using shadow analysis and spectrum matching techniques. Journal of Marine Science and Technology (Taiwan) 24, 1204–1216. https://doi.org/10.6119/JMST-016-1026-9
847. Lega, M., Persechino, G., 2014. GIS and infrared aerial view: Advanced tools for the early detection of environmental violations. WIT Transactions on Ecology and the Environment 180, 225–235. https://doi.org/10.2495/WM140191
848. Lehmann-Horn, J.A., Walbrecker, J.O., Hertrich, M., Langston, G., McClymont, A.F., Green, A.G., 2011. Imaging groundwater beneath a rugged proglacial moraine. Geophysics 76, B165–B172. https://doi.org/10.1190/geo2011-0095.1
849. Lehtiniemi, M., Ojaveer, H., David, M., Galil, B., Gollasch, S., McKenzie, C., Minchin, D., Occhipinti-Ambrogi, A., Olenin, S., Pederson, J., 2015. Dose of truth-Monitoring marine non-indigenous species to serve legislative requirements. Marine Policy 54, 26–35. https://doi.org/10.1016/j.marpol.2014.12.015
850. Lehtonen, K.K., Schiedek, D., Köhler, A., Lang, T., Vuorinen, P.J., Förlin, L., Baršiene, J., Pempkowiak, J., Gercken, J., 2006. The BEEP project in the Baltic Sea: Overview of results and outline for a regional biological effects monitoring strategy. Marine Pollution Bulletin 53, 523–537. https://doi.org/10.1016/j.marpolbul.2006.02.008
851. Lei, C., Valenta, M.M., Saripalli, K.P., Ackerman, E.J., 2007. Biosensing paraoxon in simulated environmental samples by immobilized organophosphorus hydrolase in functionalized mesoporous silica. Journal of Environmental Quality 36, 233–238. https://doi.org/10.2134/jeq2006.0216
852. Lei, R.B., Li, Z.J., Qin, J.M., Cheng, Y.F., 2009. Investigation of new technologies for in-situ ice thickness observation. Shuikexue Jinzhan/Advances in Water Science 20, 287–292.
853. Lembke, C., Grasty, S., Silverman, A., Broadbent, H., Butcher, S., Murawski, S., 2017. The Camera-Based Assessment Survey System (C-BASS): A towed camera platform for reef fish abundance surveys and benthic habitat characterization in the Gulf of Mexico. Continental Shelf Research 151, 62–71. https://doi.org/10.1016/j.csr.2017.10.010
854. Lembo, G., Bellido, J.M., Bitetto, I., Facchini, M.T., García-Jiménez, T., Stithou, M., Vassilopoulou, V.C., Spedicato, M.T., 2017. Preference modeling to support stakeholder outreach toward the common fishery policy objectives in the north Mediterranean Sea. Frontiers in Marine Science 4. https://doi.org/10.3389/fmars.2017.00328
855. Lemmens, S., 2003. Periphyton collectors as a tool to measure environmental performance of ocean outlets, Water Science and Technology.
856. Lennert-Cody, C.E., Rusin, J.D., Maunder, M.N., Everett, E.H., Largacha Delgado, E.D., Tomlinson, P.K., 2013. Studying small purse-seine vessel fishing behavior with tuna catch data: Implications for eastern Pacific Ocean dolphin conservation. Marine Mammal Science 29, 643–668. https://doi.org/10.1111/j.1748-7692.2012.00608.x
857. Lennox, R.J., Suski, C.D., Cooke, S.J., 2018. A macrophysiology approach to watershed science and management. Science of the Total Environment 626, 434–440. https://doi.org/10.1016/j.scitotenv.2018.01.069
858. Leonard, P., 2002. The role of biological research in supporting policy needs. Marine Environmental Research 54, 209–213. https://doi.org/10.1016/S0141-1136(02)00158-7
859. Leonardo, S., Toldrà, A., Rambla-Alegre, M., Fernández-Tejedor, M., Andree, K.B., Ferreres, L., Campbell, K., Elliott, C.T., O’Sullivan, C.K., Pazos, Y., Diogène, J., Campàs, M., 2018. Self-assembled monolayer-based immunoassays for okadaic acid detection in seawater as monitoring tools. Marine Environmental Research 133, 6–14. https://doi.org/10.1016/j.marenvres.2017.11.004
860. Leong, R.C., Friess, D.A., Crase, B., Lee, W.K., Webb, E.L., 2018. High-resolution pattern of mangrove species distribution is controlled by surface elevation. Estuarine, Coastal and Shelf Science 202, 185–192. https://doi.org/10.1016/j.ecss.2017.12.015
861. Léopold, M., Cakacaka, A., Meo, S., Sikolia, J., Lecchini, D., 2009. Evaluation of the effectiveness of three underwater reef fish monitoring methods in Fiji. Biodiversity and Conservation 18, 3367–3382. https://doi.org/10.1007/s10531-009-9646-y
862. Léopold, M., Ferraris, J., Labrosse, P., 2004. Assessment of the reliability of fish consumption as an indicator of reef fish catches in small Pacific islands: The example of Ouvea Island in New Caledonia. Aquatic Living Resources 17, 119–127. https://doi.org/10.1051/alr:2004020
863. Leriche, A., Boudouresque, C.F., Monestiez, P., Pasqualini, V., 2011. An improved method to monitor the health of seagrass meadows based on kriging. Aquatic Botany 95, 51–54. https://doi.org/10.1016/j.aquabot.2011.02.008
864. Lesueur, T., Boulangé-Lecomte, C., Restoux, G., Deloffre, J., Xuereb, B., Le Menach, K., Budzinski, H., Petrucciani, N., Marie, S., Petit, F., Forget-Leray, J., 2015. Toxicity of sediment-bound pollutants in the Seine estuary, France, using a Eurytemora affinis larval bioassay. Ecotoxicology and Environmental Safety 113, 169–175. https://doi.org/10.1016/j.ecoenv.2014.11.033
865. Leujak, W., Ormond, R.F.G., 2007. Comparative accuracy and efficiency of six coral community survey methods. Journal of Experimental Marine Biology and Ecology 351, 168–187. https://doi.org/10.1016/j.jembe.2007.06.028
866. Li, C., Liu, W.J., Sun, X.B., Pan, W., Wang, J.P., 2017. Multi sensing functions integrated into one carbon-dot based platform via different types of mechanisms. Sensors and Actuators B-Chemical 252, 544–553. https://doi.org/10.1016/j.snb.2017.06.036
867. Li, C., Ma, X., Zhang, X., Wang, R., Li, X., Liu, Q., 2017. Preparation of magnetic molecularly imprinted polymer nanoparticles by surface imprinting by a sol–gel process for the selective and rapid removal of di-(2-ethylhexyl) phthalate from aqueous solution. Journal of Separation Science 40, 1621–1628. https://doi.org/10.1002/jssc.201601190
868. Li, H., Chang, J., Hou, T., Ge, L., Li, F., 2016. A facile, sensitive, and highly specific trinitrophenol assay based on target-induced synergetic effects of acid induction and electron transfer towards DNA-templated copper nanoclusters. Talanta 160, 475–480. https://doi.org/10.1016/j.talanta.2016.07.030
869. Li, J., Hatton-Ellis, T.W., Lawson Handley, L.J., Kimbell, H.S., Benucci, M., Peirson, G., Hänfling, B., 2019. Ground-truthing of a fish-based environmental DNA metabarcoding method for assessing the quality of lakes. Journal of Applied Ecology. https://doi.org/10.1111/1365-2664.13352
870. Li, J., Xu, N.S., Su, W.W., 2003. Online estimation of stirred-tank microalgal photobioreactor cultures based on dissolved oxygen measurement. Biochemical Engineering Journal 14, 51–65. https://doi.org/10.1016/S1369-703X(02)00135-3
871. Li, J.L., Yu, X., 2017. LiDAR technology for wind energy potential assessment: Demonstration and validation at a site around Lake Erie. Energy Conversion and Management 144, 252–261. https://doi.org/10.1016/j.enconman.2017.04.061
872. Li, L., Song, K., 2017. Bio-optical Modeling of Phycocyanin, in: Bio-Optical Modeling and Remote Sensing of Inland Waters. pp. 233–262. https://doi.org/10.1016/B978-0-12-804644-9.00008-2
873. Li, Q., Sun, D.K., Wang, Q.F., Cai, S.R., Jia, C.Y., 2014. Cost-benefit analysis of netting cultivation to block the spread of Oncomelania snails in lake regions. Chinese Journal of Schistosomiasis Control 26, 189–191.
874. Li, W.F., Mao, J.Q., 2011. An integrated eutrophication assessment for lakes and reservoirs. Huanjing Kexue/Environmental Science 32, 3200–3206.
875. Li, Y., Shi, Z., Wu, C. f, Li, F., Li, H. y, 2007. Optimised Spatial Sampling Scheme for Soil Electriclal Conductivity Based on Variance Quad-Tree (VQT) Method. Agricultural Sciences in China 6, 1463–1471. https://doi.org/10.1016/S1671-2927(08)60009-7
876. Liao, C.M., Jou, L.J., Lin, C.M., Chiang, K.C., Yeh, C.H., Chou, B.Y.H., 2007. Predicting acute copper toxicity to valve closure behavior in the freshwater clam Corbicula fluminea supports the biotic ligand model. Environmental Toxicology 22, 295–307. https://doi.org/10.1002/tox.20263
877. Lidz, B.H., Brock, J.C., Nagle, D.B., 2008. Utility of shallow-water ATRIS images in defining biogeologic processes and self-similarity in skeletal scleractinia, Florida reefs. Journal of Coastal Research 24, 1320–1338. https://doi.org/10.2112/08-1049.1
878. Lieber, L., Nimmo-Smith, W.A.M., Waggitt, J.J., Kregting, L., 2018. Fine-scale hydrodynamic metrics underlying predator occupancy patterns in tidal stream environments. Ecological Indicators 94, 397–408. https://doi.org/10.1016/j.ecolind.2018.06.071
879. Liebig, P.M., Taylor, T.S.A., Flessa, K.W., 2003. Bones on the beach: Marine mammal taphonomy of the Colorado delta, Mexico. Palaios 18, 168–175. https://doi.org/10.1669/0883-1351(2003)18<168:BOTBMM>2.0.CO;2
880. Liermann, M., Roni, P., 2008. More sites or more years? Optimal study design for monitoring fish response to watershed restoration. North American Journal of Fisheries Management 28, 935–943. https://doi.org/10.1577/M06-175.1
881. Likens, G.E., Lindenmayer, D.B., 2011. A strategic plan for an Australian Long-Term Environmental Monitoring Network. Austral Ecology 36, 1–8. https://doi.org/10.1111/j.1442-9993.2010.02179.x
882. Lim, A., Kane, A., Arnaubec, A., Wheeler, A.J., 2018. Seabed image acquisition and survey design for cold water coral mound characterisation. Marine Geology 395, 22–32. https://doi.org/10.1016/j.margeo.2017.09.008
883. Lin, D.D., Zeng, X.J., Chen, H.G., Hong, X.L., Tao, B., Li, Y.F., Xiong, J.J., Zhou, X.N., 2009. [Cost-effectiveness and cost-benefit analysis on the integrated schistosomiasis control strategies with emphasis on infection source in Poyang Lake region]. Zhongguo ji sheng chong xue yu ji sheng chong bing za zhi = Chinese journal of parasitology & parasitic diseases 27, 297–302.
884. Lin, I.F., Tseng, I.F., Lee, C.P., Chen, G.Y., 2015. Seabed scour around a breakwater a case study in mailiao harbor. Journal of Marine Science and Technology (Taiwan) 23, 846–854. https://doi.org/10.6119/JMST-015-0610-1
885. Ling, W., Xiuqing, H., Yupeng, L., Zhizhao, L., Min, M., 2019. Selection and Characterization of Glaciers on the Tibetan Plateau as Potential Pseudoinvariant Calibration Sites. IEEE Journal of Selected Topics in Applied Earth Observations and Remote Sensing 12, 424–436. https://doi.org/10.1109/JSTARS.2018.2890672
886. Lintern, A., Leahy, P.J., Heijnis, H., Zawadzki, A., Gadd, P., Jacobsen, G., Deletic, A., McCarthy, D.T., 2016. Identifying heavy metal levels in historical flood water deposits using sediment cores. Water Research 105, 34–46. https://doi.org/10.1016/j.watres.2016.08.041
887. Lippitt, C.L., Stow, D.A., Roberts, D.A., Coulter, L.L., 2018. Multidate MESMA for monitoring vegetation growth forms in southern California shrublands. International Journal of Remote Sensing 39, 655–683. https://doi.org/10.1080/01431161.2017.1388936
888. Lirman, D., Deangelo, G., Serafy, J.E., Hazra, A., Hazra, D.S., Brown, A., 2008. Geospatial video monitoring of nearshore benthic habitats of western Biscayne Bay (Florida) using the shallow-water positioning system (SWaPS). Journal of Coastal Research 24, 135–145. https://doi.org/10.2112/04-0428.1
889. Lirman, D., Miller, M.W., 2003. Modeling and monitoring tools to assess recovery status and convergence rates between restored and undisturbed coral reef habitats. Restoration Ecology 11, 448–456. https://doi.org/10.1046/j.1526-100X.2003.rec0286.x
890. Litaker, R.W., Stewart, T.N., Eberhart, B.T.L., Wekell, J.C., Trainer, V.L., Kudela, R.M., Miller, P.E., Roberts, A., Hertz, C., Johnson, T.A., Frankfurter, G., Smith, G.J., Schnetzer, A., Schumacker, J., Bastian, J.L., Odell, A., Gentien, P., Le Gal, D., Hardison, I.R., Tester, P.A., 2008. RAPID ENZYME-LINKED IMMUNOSORBENT ASSAY FOR DETECTION OF THE ALGAL TOXIN DOMOIC ACID. Journal of Shellfish Research 27, 1301–1310. https://doi.org/10.2983/0730-8000-27.5.1301
891. Liti, D.M., Waidbacher, H., Straif, M., Mbaluka, R.K., Munguti, J.M., Kyenze, M.M., 2006. Effects of partial and complete replacement of freshwater shrimp meal (Caridinea niloticus Roux) with a mixture of plant protein sources on growth performance of Nile tilapia (Oreochromis niloticus L.) in fertilized ponds. Aquaculture Research 37, 477–483. https://doi.org/10.1111/j.1365-2109.2006.01450.x
892. Liu, B., Liu, H., Zhang, B., Bi, J., 2013. Modeling nutrient release in the tai lake basin of china: Source identification and policy implications. Environmental Management 51, 724–737. https://doi.org/10.1007/s00267-012-9999-y
893. Liu, J., Shi, Y., Sarpe, D., Neculita, M., Zhang, X., 2011. Stakeholder perception for sustainable development and pollution of coastal aquaculture. Journal of Environmental Protection and Ecology 12, 1424–1432.
894. Liu, J.Y., Jiang, G.B., Zhou, Q.F., Yang, K.W., 2001. Headspace solid-phase microextraction of butyltin species in sediments and their gas chromatographic determination. Journal of Separation Science 24, 459–464. https://doi.org/10.1002/1615-9314(20010601)24:6<459::AID-JSSC459>3.0.CO;2-I
895. Liu, S., Persson, K.M., 2013. Situations of water reuse in China. Water Policy 15, 705–727. https://doi.org/10.2166/wp.2013.275
896. Liu, X., 2017. The identification of nutrient limitations on eutrophication in Dianchi Lake, China. Water and Environment Journal 31, 592–597. https://doi.org/10.1111/wej.12284
897. Liu, X., Liu, H., Gong, H., Lin, Z., Lv, S., 2017. Appling the one-class classification method of Maxent to detect an invasive plant Spartina alterniflora with time-series analysis. Remote Sensing 9. https://doi.org/10.3390/rs9111120
898. Liu, X., Wirtz, K.W., 2010. Managing coastal area resources by stated choice experiments. Estuarine, Coastal and Shelf Science 86, 512–517. https://doi.org/10.1016/j.ecss.2009.02.020
899. Liu, Y., Engel, B.A., Collingsworth, P.D., Pijanowski, B.C., 2017. Optimal implementation of green infrastructure practices to minimize influences of land use change and climate change on hydrology and water quality: Case study in Spy Run Creek watershed, Indiana. Science of the Total Environment 601–602, 1400–1411. https://doi.org/10.1016/j.scitotenv.2017.06.015
900. Liu, Y., Fei, T., Bian, M., Corsi, F., 2010. Assessment of underwater light climate for Lake Dahuchi using field spectral data and Landsat TM. International Journal of Remote Sensing 31, 1625–1643. https://doi.org/10.1080/01431160903475282
901. Liu, Y., Wang, Z., Guo, H., Yu, S., Sheng, H., 2013. Modelling the Effect of Weather Conditions on Cyanobacterial Bloom Outbreaks in Lake Dianchi: A Rough Decision-Adjusted Logistic Regression Model. Environmental Modeling and Assessment 18, 199–207. https://doi.org/10.1007/s10666-012-9333-3
902. Liu, Y.Z., Shen, Y.L., Lv, X.Q., Liu, Q., 2017. Numeric modelling and risk assessment of pollutions in the Chinese Bohai Sea. Science China Earth Sciences 60, 1546–1557. https://doi.org/10.1007/s11430-016-9062-y
903. Ljung, K., Maley, F., Cook, A., 2010. Canal estate development in an acid sulfate soil-Implications for human metal exposure. Landscape and Urban Planning 97, 123–131. https://doi.org/10.1016/j.landurbplan.2010.05.003
904. LoBuglio, J.N., Characklis, G.W., Serre, M.L., 2007. Cost-effective water quality assessment through the integration of monitoring data and modeling results. Water Resources Research 43. https://doi.org/10.1029/2006wr005020
905. Locascio, J., Mann, D., Wilcox, K., Luther, M., 2018. Incorporation of acoustic sensors on a coastal ocean monitoring platform for measurements of biological activity. Marine Technology Society Journal 52, 64–70. https://doi.org/10.4031/MTSJ.52.3.9
906. Locker, S.D., Armstrong, R.A., Battista, T.A., Rooney, J.J., Sherman, C., Zawada, D.G., 2010. Geomorphology of mesophotic coral ecosystems: Current perspectives on morphology, distribution, and mapping strategies. Coral Reefs 29, 329–345. https://doi.org/10.1007/s00338-010-0613-6
907. Loftis, J.D., Forrest, D., Katragadda, S., Spencer, K., Organski, T., Nguyen, C., Rhee, S., 2018. StormSense: A new integrated network of IoT water level sensors in the smart cities of Hampton roads, VA. Marine Technology Society Journal 52, 56–67. https://doi.org/10.4031/MTSJ.52.2.7
908. Logan, K., Inozu, B., Roy, P., Hetet, J.F., Chesse, P., Tauzia, X., 2002. Real-time marine diesel engine simulation for fault diagnosis. Marine Technology and Sname News 39, 21–28.
909. Loh, P.C., 2012. Indigenous mollusks: Sentinels of human waste pollution of coastal marine waters, in: Mollusks: Morphology, Behavior and Ecology. pp. 253–267.
910. Loher, T., Woods, M.A., Jimenez-Hidalgo, I., Hauser, L., 2016. Variance in age-specific sex composition of Pacific halibut catches, and comparison of statistical and genetic methods for reconstructing sex ratios. Journal of Sea Research 107, 90–99. https://doi.org/10.1016/j.seares.2015.06.004
911. Lointier, M., 2002. Use of spatialized indicators in hydrological process. Application on wetland of F. Guiana. A ≪ Digital Earth ≫ : Stake for hydrology. Houille Blanche 70–75.
912. Loo, S.E., Mac Nally, R., O’Dowd, D.J., Lake, P.S., 2009. Secondary Invasions: Implications of Riparian Restoration for In-Stream Invasion by an Aquatic Grass. Restoration Ecology 17, 378–385. https://doi.org/10.1111/j.1526-100X.2008.00378.x
913. Lopatin, J., Dolos, K., Hernandez, H.J., Galleguillos, M., Fassnacht, F.E., 2016. Comparing Generalized Linear Models and random forest to model vascular plant species richness using LiDAR data in a natural forest in central Chile. Remote Sensing of Environment 173, 200–210. https://doi.org/10.1016/j.rse.2015.11.029
914. Lopez y Royo, C., Casazza, G., Pergent-Martini, C., Pergent, G., 2010. A biotic index using the seagrass Posidonia oceanica (BiPo), to evaluate ecological status of coastal waters. Ecological Indicators 10, 380–389. https://doi.org/10.1016/j.ecolind.2009.07.005
915. Lopez y Royo, C., Silvestri, C., Pergent, G., Casazza, G., 2009. Assessing human-induced pressures on coastal areas with publicly available data. Journal of Environmental Management 90, 1494–1501. https://doi.org/10.1016/j.jenvman.2008.10.007
916. Lorenz, A.W., Korte, T., Sundermann, A., Januschke, K., Haase, P., 2012. Macrophytes respond to reach-scale river restorations. Journal of Applied Ecology 49, 202–212. https://doi.org/10.1111/j.1365-2664.2011.02082.x
917. Losso, C., Ghirardini, A.V., 2010. Overview of ecotoxicological studies performed in the Venice Lagoon (Italy). Environment International 36, 92–121. https://doi.org/10.1016/j.envint.2009.07.017
918. Lounsberry, Z.T., Forrester, T.D., Olegario, M.T., Brazeal, J.L., Wittmer, H.U., Sacks, B.N., 2015. Estimating sex-specific abundance in fawning areas of a high-density Columbian black-tailed deer population using fecal DNA. Journal of Wildlife Management 79, 39–49. https://doi.org/10.1002/jwmg.817
919. Lowry, D., Stick, K., Lindquist, A., Cheng, Y.W., 2015. Evaluation of Creel Survey Methods to Estimate Recreational Harvest of Surf Smelt in Puget Sound, Washington. North American Journal of Fisheries Management 35, 403–417. https://doi.org/10.1080/02755947.2015.1009658
920. Lowry, T., Horn, C., 2010. Innovation. Jules Verne and the field of the future. Oil and Gas Journal Latinoamerica 16, 26–27.
921. Lu, Y., Liu, J., Li, J., Bruesehoff, P.J., Pavot, C.M.B., Brown, A.K., 2003. New highly sensitive and selective catalytic DNA biosensors for metal ions. Biosensors and Bioelectronics 18, 529–540. https://doi.org/10.1016/S0956-5663(03)00013-7
922. Lugg, W.H., Griffiths, J., van Rooyen, A.R., Weeks, A.R., Tingley, R., 2018. Optimal survey designs for environmental DNA sampling. Methods in Ecology and Evolution 9, 1049–1059. https://doi.org/10.1111/2041-210X.12951
923. Lumb, A., 2001. Preface. Environmental Monitoring and Assessment 67, 1–2. https://doi.org/10.1023/A:1006413714367
924. Lunt, S., 2011. Recent developments in online oil condition monitoring sensors and alignment with astm methods and practices. Journal of ASTM International 8. https://doi.org/10.1520/JAI103632
925. Luo, L., Jiang, J., Zhang, G., Wang, L., Wang, Z., Yang, J., Yu, C., 2017. Stroke mortality attributable to ambient particulate matter pollution from 1990 to 2015 in China: An age-period-cohort and spatial autocorrelation analysis. International Journal of Environmental Research and Public Health 14. https://doi.org/10.3390/ijerph14070772
926. Luo, X., Kwok, K.L., Liu, Y., Jiao, J., 2017. A Permanent Multilevel Monitoring and Sampling System in the Coastal Groundwater Mixing Zones. Groundwater 55, 577–587. https://doi.org/10.1111/gwat.12510
927. Lydersen, C., Nost, O.A., Lovell, P., McConnell, B.J., Gammelsrod, T., Hunter, C., Fedak, M.A., Kovacs, K.M., 2002. Salinity and temperature structure of a freezing Arctic fjord - monitored by white whales (Delphinapterus leucas). Geophysical Research Letters 29. https://doi.org/10.1029/2002gl015462
928. Macário, I.P.E., Castro, B.B., Nunes, I.M.S., Pizarro, C., Coelho, C., Gonçalves, F., de Figueiredo, D.R., 2017. Stepwise strategy for monitoring toxic cyanobacterial blooms in lentic water bodies. Environmental Monitoring and Assessment 189. https://doi.org/10.1007/s10661-017-6292-9
929. MacDonald, A., 2005. New developments increase use of airborne LIDAR bathymetry. Sea Technology 46, 46–48.
930. MacHiwal, D., Jha, M.K., Mal, B.C., 2011. GIS-based assessment and characterization of groundwater quality in a hard-rock hilly terrain of Western India. Environmental Monitoring and Assessment 174, 645–663. https://doi.org/10.1007/s10661-010-1485-5
931. Mackay, M., Jennings, S., van Putten, E.I., Sibly, H., Yamazaki, S., 2018. When push comes to shove in recreational fishing compliance, think “nudge.” Marine Policy 95, 256–266. https://doi.org/10.1016/j.marpol.2018.05.026
932. Mackinson, S., Freeman, S., Flatt, R., Meadows, B., 2004. Improved acoustic surveys that save time and money: integrating fisheries and ground-discrimination acoustic technologies. Journal of Experimental Marine Biology and Ecology 305, 129–140. https://doi.org/10.1016/j.jembe.2003.10.020
933. MacNeil, C., 2012. Freshwater pollution and biological water quality monitoring: The increasing problem of invasive species and accurate ecological assessment, in: Invasive Species: Threats, Ecological Impact and Control Methods. pp. 141–153.
934. Madricardo, F., Foglini, F., Kruss, A., Ferrarin, C., Pizzeghello, N.M., Murri, C., Rossi, M., Bajo, M., Bellafiore, D., Campiani, E., Fogarin, S., Grande, V., Janowski, L., Keppel, E., Leidi, E., Lorenzetti, G., Maicu, F., Maselli, V., Mercorella, A., Gavazzi, G.M., Minuzzo, T., Pellegrini, C., Petrizzo, A., Prampolini, M., Remia, A., Rizzetto, F., Rovere, M., Sarretta, A., Sigovini, M., Sinapi, L., Umgiesser, G., Trincardi, F., 2017. Data Descriptor: High resolution multibeam and hydrodynamic datasets of tidal channels and inlets of the Venice Lagoon. Scientific Data 4. https://doi.org/10.1038/sdata.2017.121
935. Madricardo, F., Foglini, F., Kruss, A., Ferrarin, C., Pizzeghello, N.M., Murri, C., Rossi, M., Bajo, M., Bellafiore, D., Campiani, E., Fogarin, S., Grande, V., Janowski, L., Keppel, E., Leidi, E., Lorenzetti, G., Maicu, F., Maselli, V., Mercorella, A., Montereale Gavazzi, G., Minuzzo, T., Pellegrini, C., Petrizzo, A., Prampolini, M., Remia, A., Rizzetto, F., Rovere, M., Sarretta, A., Sigovini, M., Sinapi, L., Umgiesser, G., Trincardi, F., 2017. High resolution multibeam and hydrodynamic datasets of tidal channels and inlets of the Venice Lagoon. Scientific Data 4. https://doi.org/10.1038/sdata.2017.121
936. Mahapatra, D.M., Chanakya, H.N., Ramachandra, T.V., 2013. Treatment efficacy of algae-based sewage treatment plants. Environmental Monitoring and Assessment 185, 7145–7164. https://doi.org/10.1007/s10661-013-3090-x
937. Mahmood, A., Bennamoun, M., An, S.J., Sohel, F.A., Boussaid, F., Hovey, R., Kendrick, G.A., Fisher, R.B., 2019. Deep Image Representations for Coral Image Classification. Ieee Journal of Oceanic Engineering 44, 121–131. https://doi.org/10.1109/joe.2017.2786878
938. Maichomo, M.W., Kosura, W.O., Gathuma, J.M., Gitau, G.K., Ndung’u, J.M., Nyamwaro, S.O., 2009. Economic assessment of the performance of trypanotolerant cattle breeds in a pastoral production system in Kenya. Journal of the South African Veterinary Association 80, 157–162.
939. Maiti, S., 2013. Interpretation of coastal morphodynamics of subarnarekha estuary using integrated cartographic and field techniques. Current Science 104, 1709–1714.
940. Maiti, S., Bhattacharya, A.K., 2009. Shoreline change analysis and its application to prediction: A remote sensing and statistics based approach. Marine Geology 257, 11–23. https://doi.org/10.1016/j.margeo.2008.10.006
941. Maleki, S., Soffianian, A., Koupaei, S.S., Saatchi, S., Pourmanafi, S., 2018. Application of Remote Sensing in Monitoring Unsustainable Wetlands: Case Study Hamun Wetland. Journal of the Indian Society of Remote Sensing 46, 1871–1879. https://doi.org/10.1007/s12524-018-0842-7
942. Mallet, D., Pelletier, D., 2014. Underwater video techniques for observing coastal marine biodiversity: A review of sixty years of publications (1952-2012). Fisheries Research 154, 44–62. https://doi.org/10.1016/j.fishres.2014.01.019
943. Malley, D.F., Williams, P., 2014. Analysis of sediments and suspended material in lake ecosystems using near-infrared spectroscopy: A review. Aquatic Ecosystem Health and Management 17, 447–453. https://doi.org/10.1080/14634988.2014.979311
944. Malone, T.C., 2003. The coastal component of the U.S. integrated ocean observing system. Environmental Monitoring and Assessment 81, 51–62. https://doi.org/10.1023/A:1021352319046
945. Malve, O., Hjerppe, T., Tattari, S., Väisänen, S., Huttunen, I., Kotamäki, N., Kallio, K., Taskinen, A., Kauppila, P., 2016. Participatory operations model for cost-efficient monitoring and modeling of river basins - A systematic approach. Science of the Total Environment 540, 79–89. https://doi.org/10.1016/j.scitotenv.2015.06.105
946. Malve, O., Tattari, S., Riihimäki, J., Jaakkola, E., Vo, A., Williams, R., Bärlund, I., 2012. Estimation of diffuse pollution loads in Europe for continental scale modelling of loads and in-stream river water quality. Hydrological Processes 26, 2385–2394. https://doi.org/10.1002/hyp.9344
947. Mancia, A., Abelli, L., Kucklick, J.R., Rowles, T.K., Wells, R.S., Balmer, B.C., Hohn, A.A., Baatz, J.E., Ryan, J.C., 2015. Microarray applications to understand the impact of exposure to environmental contaminants in wild dolphins (Tursiops truncatus). Marine Genomics 19, 47–57. https://doi.org/10.1016/j.margen.2014.11.002
948. Mancini, A., Elsadek, I., Madon, B., 2015. When simple is better: Comparing two sampling methods to estimate green turtles abundance at coastal feeding grounds. Journal of Experimental Marine Biology and Ecology 465, 113–120. https://doi.org/10.1016/j.jembe.2015.01.004
949. Mancini, E.R., Steen, A., Rausina, G.A., Wong, D.C.L., Arnold, W.R., Gostomski, F.E., Davies, T., Hockett, J.R., Stubblefield, W.A., Drottar, K.R., Springer, T.A., Errico, P., 2002. MTBE ambient water quality criteria development: A public/private partnership. Environmental Science & Technology 36, 125–129. https://doi.org/10.1021/es002059b
950. Mandujano, S., 2005. Track count calibration to estimate density of white-tailed deer (Odocoileus virginianus) in Mexican dry tropical forest. Southwestern Naturalist 50, 223–229. https://doi.org/10.1894/0038-4909(2005)050[0223:TCCTED]2.0.CO;2
951. Mangadze, T., Dalu, T., William Froneman, P., 2019. Biological monitoring in southern Africa: A review of the current status, challenges and future prospects. Science of the Total Environment 648, 1492–1499. https://doi.org/10.1016/j.scitotenv.2018.08.252
952. Manna, S., Nandy, S., Chanda, A., Akhand, A., Hazra, S., Dadhwal, V.K., 2014. Estimating aboveground biomass in Avicennia marina plantation in Indian Sundarbans using high-resolution satellite data. Journal of Applied Remote Sensing 8. https://doi.org/10.1117/1.JRS.8.083638
953. Mannocci, A., La Torre, G., Chiaradia, G., De Waure, C., Mainelli, M.T., Cernigliaro, A., Bruno, S., Ricciardi, W., 2007. Epidemiology and direct medical costs of human Leishmaniasis in Italy. Journal of Preventive Medicine and Hygiene 48, 27–36.
954. Marancik, K.E., Richardson, D.E., Lyczkowski-Shultz, J., Konieczna, M., Cowen, R.K., 2010. Evaluation of morphological characters to identify grouper (serranidae: Epinephelini) larvae in the Gulf of Mexico using genetically identified specimens. Bulletin of Marine Science 86, 571–624.
955. Marconi, G., Landucci, F., Rosellini, D., Venanzoni, R., Albertini, E., 2018. DNA barcoding as a tool for early warning and monitoring alien duckweeds (Lemna sp.pl.): the case of Central Italy. Plant Biosystems. https://doi.org/10.1080/11263504.2018.1536087
956. Mariani, P., Quincoces, I., Haugholt, K.H., Chardard, Y., Visser, A.W., Yates, C., Piccinno, G., Reali, G., Risholm, P., Thielemann, J.T., 2018. Range-gated imaging system for underwater monitoring in ocean environment. Sustainability (Switzerland) 11. https://doi.org/10.3390/su11010162
957. Marquette, P., Dereims, A., Hugon, M., Esnault, G., Pickett, A., Karagiannis, D., Gkinosatis, A., 2014. Simulation based solutions for industrial manufacture of large infusion composite parts. SAE Technical Papers 1. https://doi.org/10.4271/2014-01-0965
958. Martin, C.S., Giannoulaki, M., De Leo, F., Scardi, M., Salomidi, M., Knitweiss, L., Pace, M.L., Garofalo, G., Gristina, M., Ballesteros, E., Bavestrello, G., Belluscio, A., Cebrian, E., Gerakaris, V., Pergent, G., Pergent-Martini, C., Schembri, P.J., Terribile, K., Rizzo, L., Ben Souissi, J., Bonacorsi, M., Guarnieri, G., Krzelj, M., Macic, V., Punzo, E., Valavanis, V., Fraschetti, S., 2014. Coralligenous and maerl habitats: predictive modelling to identify their spatial distributions across the Mediterranean Sea. Scientific Reports 4. https://doi.org/10.1038/srep05073
959. Martin, C.S., Giannoulaki, M., De Leo, F., Scardi, M., Salomidi, M., Knitweiss, L., Pace, M.L., Garofalo, G., Gristina, M., Ballesteros, E., Bavestrello, G., Belluscio, A., Cebrian, E., Gerakaris, V., Pergent, G., Pergent-Martini, C., Schembri, P.J., Terribile, K., Rizzo, L., Ben Souissi, J., Bonacorsi, M., Guarnieri, G., Krzelj, M., Macic, V., Punzo, E., Valavanis, V., Fraschetti, S., 2014. Coralligenous and maërl habitats: Predictive modelling to identify their spatial distributions across the mediterranean sea. Scientific Reports 4. https://doi.org/10.1038/srep05073
960. Martin, P., McDonald, A., Munday, E., 2015. Robust data in sensitive environments. Hydro International 19, 24–27.
961. Martínez-Gómez, C., Robinson, C.D., Burgeot, T., Gubbins, M., Halldorsson, H.P., Albentosa, M., Bignell, J.P., Hylland, K., Vethaak, A.D., 2017. Biomarkers of general stress in mussels as common indicators for marine biomonitoring programmes in Europe: The ICON experience. Marine Environmental Research 124, 70–80. https://doi.org/10.1016/j.marenvres.2015.10.012
962. Martinez-Haro, M., Acevedo, P., Pais-Costa, A.J., Taggart, M.A., Martins, I., Ribeiro, R., Marques, J.C., 2016a. Assessing estuarine quality: A cost-effective in situ assay with amphipods. Environmental Pollution 212, 382–391. https://doi.org/10.1016/j.envpol.2016.01.071
963. Martinez-Haro, M., Moreira-Santos, M., Marques, J.C., Ribeiro, R., 2014. A short-term laboratory and in situ sediment assay based on the postexposure feeding of the estuarine isopod Cyathura carinata. Environmental Research 134, 242–250. https://doi.org/10.1016/j.envres.2014.07.013
964. Martinez-Haro, M., Pais-Costa, A.J., Verdelhos, T., Marques, J.C., Acevedo, P., 2016b. Optimising a clearance index based on neutral red as an indicator of physiological stress for bivalves. Ecological Indicators 71, 514–521. https://doi.org/10.1016/j.ecolind.2016.07.025
965. Martinis, E.M., Escudero, L.B., Salvarezza, R., Calderón, M.F., Ibañez, F.J., Wuilloud, R.G., 2013. Liquid-liquid microextraction based on a dispersion of Pd nanoparticles combined with ETAAS for sensitive Hg determination in water samples. Talanta 108, 46–52. https://doi.org/10.1016/j.talanta.2013.02.067
966. Marttunen, M., Vehanen, T., 2004. Toward adaptive management: The impacts of different management strategies on fish stocks and fisheries in a large regulated lake. Environmental Management 33, 840–854. https://doi.org/10.1007/s00267-003-3021-7
967. Marugán, J., Bru, D., Pablos, C., Catalá, M., 2012. Comparative evaluation of acute toxicity by Vibrio fischeri and fern spore based bioassays in the follow-up of toxic chemicals degradation by photocatalysis. Journal of Hazardous Materials 213–214, 117–122. https://doi.org/10.1016/j.jhazmat.2012.01.075
968. Masanabo, N.M., Zinyemba, O., Mketo, N., 2019. A greener microwave-assisted extraction method for rapid spectroscopic determination of selected metals in river and freshwater sediment certified reference materials. International Journal of Environmental Analytical Chemistry 99, 33–46. https://doi.org/10.1080/03067319.2018.1559306
969. Masese, F.O., Omukoto, J.O., Nyakeya, K., 2013. Biomonitoring as a prerequisite for sustainable water resources: A review of current status, opportunities and challenges to scaling up in East Africa. Ecohydrology and Hydrobiology 13, 173–191. https://doi.org/10.1016/j.ecohyd.2013.06.004
970. Maslin, E., 2017. E&P goes green. Offshore Engineer 42, 18–21.
971. Masri, M.A., Jurkowski, W., Shaigani, P., Haack, M., Mehlmer, N., Brück, T., 2018. A waste-free, microbial oil centered cyclic bio-refinery approach based on flexible macroalgae biomass. Applied Energy 224, 1–12. https://doi.org/10.1016/j.apenergy.2018.04.089
972. Masuda, S., Hosoda, S., 2014. Effective design of profiling float network for oceanic heat-content monitoring. The Scientific World Journal 2014. https://doi.org/10.1155/2014/340518
973. Matthes, U., Gerrath, J.A., Larson, D.W., 2003. Experimental restoration of disturbed cliff-edge forests in Bruce Peninsula National Park, Ontario, Canada. Restoration Ecology 11, 174–184. https://doi.org/10.1046/j.1526-100X.2003.00140.x
974. Matthews, W.J., Marsh-Matthews, E., 2011. An invasive fish species within its native range: Community effects and population dynamics of Gambusia affinis in the central United States. Freshwater Biology 56, 2609–2619. https://doi.org/10.1111/j.1365-2427.2011.02691.x
975. Mauffret, A., Rottiers, A., Federle, T., Gillan, D.C., Hampel, M., Blasco, J., Temara, A., 2009. Colonized beads as inoculum for marine biodegradability assessment: Application to Linear Alkylbenzene Sulfonate. Environment International 35, 885–892. https://doi.org/10.1016/j.envint.2009.03.007
976. Mauvisseau, Q., Burian, A., Gibson, C., Brys, R., Ramsey, A., Sweet, M., 2019. Influence of accuracy, repeatability and detection probability in the reliability of species-specific eDNA based approaches. Scientific Reports 9. https://doi.org/10.1038/s41598-018-37001-y
977. Maxwell, S.M., Ban, N.C., Morgan, L.E., 2014. Pragmatic approaches for effective management of pelagic marine protected areas. Endangered Species Research 26, 59–74. https://doi.org/10.3354/esr00617
978. May, L., Taylor, P., Spears, B., Pitt, J.A., Collins, A.L., Corkley, I., Anthony, S., Skirvin, D., Lee, D., Naden, P., 2019. Decision support framework to identify lakes that are likely to meet water quality targets if external inputs of phosphorus from agriculture are reduced. Limnetica 38, 489–501. https://doi.org/10.23818/limn.38.28
979. Maynou, X., Martín, R., Aranda, D., 2017. The role of small secondary biotopes in a highly fragmented landscape as habitat and connectivity providers for dragonflies (Insecta: Odonata). Journal of Insect Conservation 21, 517–530. https://doi.org/10.1007/s10841-017-9992-0
980. Mazurkiewicz, M., Górska, B., Jankowska, E., Włodarska-Kowalczuk, M., 2016. Assessment of nematode biomass in marine sediments: A semi-automated image analysis method. Limnology and Oceanography: Methods 14, 816–827. https://doi.org/10.1002/lom3.10128
981. McBarnet, A., 2003. Seismic’s scant reward for technology success. Petroleum Review 57, 12–14.
982. McBride, R.S., 2014. Managing a Marine Stock Portfolio: Stock Identification, Structure, and Management of 25 Fishery Species along the Atlantic Coast of the United States. North American Journal of Fisheries Management 34, 710–734. https://doi.org/10.1080/02755947.2014.902408
983. McCaul, M., McNamara, E., Diamond, D., 2016. Development of cost effective sensors for the in-situ monitoring of eutrophication in marine waters. Abstracts of Papers of the American Chemical Society 251.
984. McClain, C.R., Feldman, G.C., Hooker, S.B., 2004. An overview of the SeaWiFS project and strategies for producing a climate research quality global ocean bio-optical time series. Deep-Sea Research Part Ii-Topical Studies in Oceanography 51, 5–42. https://doi.org/10.1016/j.dsr2.2003.11.001
985. McCormick, J.L., 2017. Evaluation of Methods to Estimate Salmon Harvest Using Angler Harvest Permits Available on a Smartphone Application. North American Journal of Fisheries Management 37, 1–8. https://doi.org/10.1080/02755947.2016.1221005
986. McDonald, M.E., 2000. EMAP overview: Objectives, approaches, and achievements. Environmental Monitoring and Assessment 64, 3–8.
987. McLamore, E.S., Garland, J.L., Mackowiak, C., Desaunay, A., Garland, N., Chaturvedi, P., Taguchi, M., Dreaden, K., Catechis, J., Ullman, J.L., 2014. Development and validation of an open source O-2-sensitive gel for physiological profiling of soil microbial communities. Journal of Microbiological Methods 96, 62–67. https://doi.org/10.1016/j.mimet.2013.10.016
988. McLean, W.R., Goldingay, R.L., Westcott, D.A., 2017. Visual lures increase camera-trap detection of the southern cassowary (Casuarius casuarius johnsonii). Wildlife Research 44, 230–237. https://doi.org/10.1071/WR16025
989. McPartlin, D.A., Lochhead, M.J., Connell, L.B., Doucette, G.J., O’Kennedy, R.J., 2016. Use of biosensors for the detection of marine toxins. Essays in Biochemistry 60, 49–58. https://doi.org/10.1042/EBC20150006
990. McVittie, A., Cole, L., Wreford, A., Sgobbi, A., Yordi, B., 2018. Ecosystem-based solutions for disaster risk reduction: Lessons from European applications of ecosystem-based adaptation measures. International Journal of Disaster Risk Reduction 32, 42–54. https://doi.org/10.1016/j.ijdrr.2017.12.014
991. Meals, D.W., 2001. Water quality response to riparian restoration in an agricultural watershed in Vermont, USA, Water Science and Technology.
992. Meals, D.W., Hopkins, R.B., 2002. Phosphorus reductions following riparian restoration in two agricultural watersheds in Vermont, USA, Water Science and Technology.
993. Mehta, R., 2006. Testing the waters. Materials World 14, 5.
994. Meleder, V., Populus, J., Guillaumont, B., Perrot, T., Mouquet, P., 2010. Predictive modelling of seabed habitats: case study of subtidal kelp forests on the coast of Brittany, France. Marine Biology 157, 1525–1541. https://doi.org/10.1007/s00227-010-1426-4
995. Mellin, C., Parrott, L., Andréfouët, S., Bradshaw, C.J.A., MacNeil, M.A., Caley, M.J., 2012. Multi-scale marine biodiversity patterns inferred efficiently from habitat image processing. Ecological Applications 22, 792–803. https://doi.org/10.1890/11-2105.1
996. Melnik, S., Neumann, A.C., Karongo, R., Dirndorfer, S., Stübler, M., Ibl, V., Niessner, R., Knopp, D., Stoger, E., 2018. Cloning and plant-based production of antibody MC10E7 for a lateral flow immunoassay to detect [4-arginine]microcystin in freshwater. Plant Biotechnology Journal 16, 27–38. https://doi.org/10.1111/pbi.12746
997. Melo, A., Ferreira, I., Mansilha, C., 2015. Application of a fast and cost-effective in situ derivatization method prior to gas chromatography with mass spectrometry to monitor endocrine disruptors in water matrices. Journal of Separation Science 38, 1983–1989. https://doi.org/10.1002/jssc.201401463
998. Merkel, F.R., 2010. Evidence of Recent Population Recovery in Common Eiders Breeding in Western Greenland. Journal of Wildlife Management 74, 1869–1874. https://doi.org/10.2193/2009-189
999. Merrill, R.M., Hyatt, B., Aldana, S.G., Kinnersley, D., 2011. Lowering employee health care costs through the healthy lifestyle incentive program. Journal of Public Health Management and Practice 17, 225–232. https://doi.org/10.1097/PHH.0b013e3181f54128
1000. Merten, W., Rivera, R., Appeldoorn, R., Serrano, K., Collazo, O., Jimenez, N., 2018. Use of video monitoring to quantify spatial and temporal patterns in fishing activity across sectors. Scientia Marina 82, 107–117. https://doi.org/10.3989/scimar.04730.09A
1001. Merten, W., Rivera, R., Appeldoorn, R., Serrano, K., Collazo, O., Jimenez, N., 2018. Use of video monitoring to quantify spatial and temporal patterns in fishing activity across sectors at moored fish aggregating devices off Puerto Rico. Scientia Marina 82, 107–117. https://doi.org/10.3989/scimar.04730.09A
1002. Michailova, P., Sella, G., Petrova, N., 2012. Chironomids (Diptera) and their salivary gland chromosomes as indicators of trace-metal genotoxicity. Italian Journal of Zoology 79, 218–230. https://doi.org/10.1080/11250003.2011.622084
1003. Migaszewski, Z.M., Galuszka, A., Dolegowska, S., 2018. Arsenic in the Wisniowka acid mine drainage area (south-central Poland) Mineralogy, hydrogeochemistry, remediation. Chemical Geology 493, 491–503. https://doi.org/10.1016/j.chemgeo.2018.06.027
1004. Miller, A.D., Taylor, B.J., Johnson, B.D., 2013. Energy expenditure and intensity levels during a 6170-m summit in the karakoram mountains. Wilderness and Environmental Medicine 24, 337–344. https://doi.org/10.1016/j.wem.2013.06.009
1005. Miller, J.F., Kelley, A.R., Kelley, J.T., Belknap, D.F., Spiess, A.E., 2018. Ground-Penetratina Radar as a Cultural Resource Management Tool for Assessment of Eroding SheII Middens. Conservation and Management of Archaeological Sites 20, 199–214. https://doi.org/10.1080/13505033.2018.1516446
1006. Miller, J.F., Kelley, A.R., Kelley, J.T., Belknap, D.F., Spiess, A.E., 2018. Ground-Penetrating Radar as a Cultural Resource Management Tool for Assessment of Eroding Shell Middens. Conservation and Management of Archaeological Sites 20, 199–214. https://doi.org/10.1080/13505033.2018.1516446
1007. Mills, D.K., Keeble, K., Foden, J., Forster, R.M., Rees, J., Keeble, S., Malcolm, S.J., 2011. EMECO datatool: A regional scale data integration and assessment system for marine environmental policy needs. Underwater Technology 30, 71–78. https://doi.org/10.3723/ut.30.071
1008. Mills, K.L., Laidig, T., Ralston, S., Sydeman, W.J., 2007. Diets of top predators indicate pelagic juvenile rockfish (Sebastes spp.) abundance in the California Current System. Fisheries Oceanography 16, 273–283. https://doi.org/10.1111/j.1365-2419.2007.00429.x
1009. Minamoto, T., Fukuda, M., Katsuhara, K.R., Fujiwara, A., Hidaka, S., Yamamoto, S., Takahashi, K., Masuda, R., 2017. Environmental DNA reflects spatial and temporal jellyfish distribution. PLoS ONE 12. https://doi.org/10.1371/journal.pone.0173073
1010. Minas, K., Karunakaran, E., Bond, T., Gandy, C., Honsbein, A., Madsen, M., Amezaga, J., Amtmann, A., Templeton, M.R., Biggs, C.A., Lawton, L., 2015. Biodesalination: an emerging technology for targeted removal of Na+ and Cl− from seawater by cyanobacteria. Desalination and Water Treatment 55, 2647–2668. https://doi.org/10.1080/19443994.2014.940647
1011. Minchin, D., Olenin, S., Liu, T.K., Cheng, M., Huang, S.C., 2016. Rapid assessment of target species: Byssate bivalves in a large tropical port. Marine Pollution Bulletin 112, 177–182. https://doi.org/10.1016/j.marpolbul.2016.08.023
1012. Minoli, D., 2015. Innovations in Satellite Communication and Satellite Technology: The Industry Implications of DVB-S2X, High Throughput Satellites, Ultra HD, M2M, and IP, Innovations in Satellite Communication and Satellite Technology: The Industry Implications of DVB-S2X, High Throughput Satellites, Ultra HD, M2M, and IP. https://doi.org/10.1002/9781118984086
1013. Minu, P., Shaju, S.S., Souda, V.P., Usha, B., Ashraf, P.M., Meenakumari, B., 2015. Hyperspectral Variability of Phytoplankton Blooms in Coastal Waters off Kochi, South-eastern Arabian Sea. Fishery Technology 52, 218–222.
1014. Misra, A., Balaji, R., 2017. Simple Approaches to Oil Spill Detection Using Sentinel Application Platform (SNAP)-Ocean Application Tools and Texture Analysis: A Comparative Study. Journal of the Indian Society of Remote Sensing 45, 1065–1075. https://doi.org/10.1007/s12524-016-0658-2
1015. Missiaen, T., 2005. VHR marine 3D seismics for shallow water investigations: Some practical guidelines. Marine Geophysical Research 26, 145–155. https://doi.org/10.1007/s11001-005-3708-7
1016. Missiaen, T., Verhegge, J., Heirman, K., Crombé, P., 2015. Potential of cone penetrating testing for mapping deeply buried palaeolandscapes in the context of archaeological surveys in polder areas. Journal of Archaeological Science 55, 174–187. https://doi.org/10.1016/j.jas.2015.01.003
1017. Miteva, D.A., Murray, B.C., Pattanayak, S.K., 2015. Do protected areas reduce blue carbon emissions? A quasi-experimental evaluation of mangroves in Indonesia. Ecological Economics 119, 127–135. https://doi.org/10.1016/j.ecolecon.2015.08.005
1018. Miya, M., Sato, Y., Fukunaga, T., Sado, T., Poulsen, J.Y., Sato, K., Minamoto, T., Yamamoto, S., Yamanaka, H., Araki, H., Kondoh, M., Iwasaki, W., 2015. MiFish, a set of universal PCR primers for metabarcoding environmental DNA from fishes: Detection of more than 230 subtropical marine species. Royal Society Open Science 2. https://doi.org/10.1098/rsos.150088
1019. Mkwara, L., Marsh, D., Scarpa, R., 2015. The effect of within-season variability on estimates of recreational value for trout anglers in New Zealand. Ecological Economics 119, 338–345. https://doi.org/10.1016/j.ecolecon.2015.09.012
1020. Mogollón, B., Villamagna, A.M., 2015. Updating the managers toolbox: Mapping spatio-temporal trends in freshwater fishing. Journal of Outdoor Recreation and Tourism 7–8, 89–95. https://doi.org/10.1016/j.jort.2014.09.009
1021. Molenaar, E.J., Tsamenyi, M., 2000. Satellite-based vessel monitoring systems for fisheries management: International legal aspects. International Journal of Marine and Coastal Law 15, 65–109. https://doi.org/10.1023/A:1008706319665
1022. Molina, M., Hunter, S., Cyterski, M., Peed, L.A., Kelty, C.A., Sivaganesan, M., Mooney, T., Prieto, L., Shanks, O.C., 2014. Factors affecting the presence of human-associated and fecal indicator real-time quantitative PCR genetic markers in urban-impacted recreational beaches. Water Research 64, 196–208. https://doi.org/10.1016/j.watres.2014.06.036
1023. Molinet, C., Niklitschek, E., Seguel, M., Díaz, P., 2010. Trends of natural accumulation and detoxification of paralytic shellfish poison in two bivalves from the Northwest Patagonian inland sea. Revista de Biologia Marina y Oceanografia 45, 195–204. https://doi.org/10.4067/S0718-19572010000200001
1024. Molognoni, L., Dos Santos, J.N., Kleemann, C.R., Costa, A.C.O., Hoff, R.B., Daguer, H., 2019. Cost-Effective and High-Reliability Analytical Approach for Multitoxin Screening in Bivalve Mollusks by Liquid Chromatography Coupled to Tandem Mass Spectrometry. Journal of Agricultural and Food Chemistry 67, 2691–2699. https://doi.org/10.1021/acs.jafc.8b06600
1025. Monitoring system approved for US fisheries, 2007. . Ship and Boat International 30.
1026. Monk, J., Ierodiaconou, D., Bellgrove, A., Laurenson, L., 2008. Using community-based monitoring with GIS to create habitat maps for a marine protected area in Australia. Journal of the Marine Biological Association of the United Kingdom 88, 865–871. https://doi.org/10.1017/S0025315408001835
1027. Moody, A.T., Neeson, T.M., Wangen, S., Dischler, J., Diebel, M.W., Milt, A., Herbert, M., Khoury, M., Yacobson, E., Doran, P.J., Ferris, M.C., O’Hanley, J.R., McIntyre, P.B., 2017. Pet Project or Best Project? Online Decision Support Tools for Prioritizing Barrier Removals in the Great Lakes and Beyond. Fisheries 42, 57–65. https://doi.org/10.1080/03632415.2016.1263195
1028. Moomaw, W.R., Birch, M.B., 2005. Cascading costs: an economic nitrogen cycle. Science in China. Series C, Life sciences / Chinese Academy of Sciences 48 Spec No, 678–696.
1029. Moon, W.M., Won, J.S., 2002. Polarimetric Synthetic Aperture Radar (SAR) and geodynamic applications: An overview of a new Earth system observation concept. Geosciences Journal 6, 341–346. https://doi.org/10.1007/BF03020618
1030. Moore, C.D., Mason, A.R., 2012. Demonstration Survey of Prehistoric Reef-Net Sites with Sidescan Sonar, near Becher Bay, British Columbia, Canada. International Journal of Nautical Archaeology 41, 179–189. https://doi.org/10.1111/j.1095-9270.2011.00324.x
1031. Moore, M.N., Depledge, M.H., Readman, J.W., Paul Leonard, D.R., 2004. An integrated biomarker-based strategy for ecotoxicological evaluation of risk in environmental management. Mutation Research - Fundamental and Molecular Mechanisms of Mutagenesis 552, 247–268. https://doi.org/10.1016/j.mrfmmm.2004.06.028
1032. Moreira, S.M., Guilhermino, L., Ribeiro, R., 2006. An in situ assay with the microalga Phaeodactylum tricornutum for sediment-overlying water toxicity evaluations in estuaries. Environmental Toxicology and Chemistry 25, 2272–2279. https://doi.org/10.1897/05-266R.1
1033. Moreira, S.M., Moreira-Santos, M., Ribeiro, R., Guilhermino, L., 2004. The “Coral Bulker” fuel oil spill on the north coast of portugal: Spatial and temporal biomarker responses in Mytilus galloprovincialis. Ecotoxicology 13, 619–630. https://doi.org/10.1007/s10646-003-4422-3
1034. Moreira-Santos, M., Soares, A.M.V.M., Ribeiro, R., 2004. A phytoplankton growth assay for routine in situ environmental assessments. Environmental Toxicology and Chemistry 23, 1549–1560. https://doi.org/10.1897/03-389
1035. Moriarty, K.M., Linnell, M.A., Thornton, J.E., Watts, G.W., III, 2018. Seeking efficiency with carnivore survey methods: A case study with elusive martens. Wildlife Society Bulletin 42, 403–413. https://doi.org/10.1002/wsb.896
1036. Moriasi, D.N., Steiner, J.L., Arnold, J.G., 2011. Sediment measurement and transport modeling: Impact of riparian and filter strip buffers. Journal of Environmental Quality 40, 807–814. https://doi.org/10.2134/jeq2010.0066
1037. Morris, R.L., Konlechner, T.M., Ghisalberti, M., Swearer, S., 2018. From grey to green: Efficacy of eco-engineering solutions for nature-based coastal defence. Global Change Biology 24, 1827–1842. https://doi.org/10.1111/gcb.14063
1038. Morrisey, D.J., Cole, R.G., Bell, J., Lane, I., Read, G.B., 2003. Low abundances and diversities of benthic faunas of shallow, coastal sediments in the Solomon Islands and their implications for assessing environmental impacts of logging. Pacific Conservation Biology 9, 215–227.
1039. Mortensen, L.O., Ulrich, C., Olesen, H.J., Bergsson, H., Berg, C.W., Tzamouranis, N., Dalskov, J., 2017. Effectiveness of fully documented fisheries to estimate discards in a participatory research scheme. Fisheries Research 187, 150–157. https://doi.org/10.1016/j.fishres.2016.11.010
1040. Mosindy, T.E., Duffy, M.J., 2007. The use of angler diary surveys to evaluate long-term changes in muskellunge populations on Lake of the Woods, Ontario. Environmental Biology of Fishes 79, 71–83. https://doi.org/10.1007/s10641-006-9167-4
1041. Moustafa, K., 2017. Greening Drylands with Seawater Easily and Naturally. Trends in Biotechnology 35, 189–191. https://doi.org/10.1016/j.tibtech.2016.09.005
1042. Moxley, J.H., Bogomolni, A., Hammill, M.O., Moore, K.M.T., Polito, M.J., Sette, L., Sharp, W.B., Waring, G.T., Gilbert, J.R., Halpin, P.N., Johnston, D.W., 2017. Google haul out: Earth observation imagery and digital aerial surveys in coastal wildlife management and abundance estimation. BioScience 67, 760–768. https://doi.org/10.1093/biosci/bix059
1043. Mu, W., van Middelaar, C.E., Bloemhof, J.M., Engel, B., de Boer, I.J.M., 2017. Benchmarking the environmental performance of specialized milk production systems: selection of a set of indicators. Ecological Indicators 72, 91–98. https://doi.org/10.1016/j.ecolind.2016.08.009
1044. Mueller, K.W., 2003. A comparison of electrofishing and scuba diving to sample black bass in western Washington lakes. North American Journal of Fisheries Management 23, 632–639. https://doi.org/10.1577/1548-8675(2003)023<0632:acoeas>2.0.co;2
1045. Muenich, R.L., Kalcic, M.M., Winsten, J., Fisher, K., Day, M., O’Neil, G., Wang, Y.C., Scavia, D., 2017. Pay-for-performance conservation using swat highlights need for field-level agricultural conservation. Transactions of the ASABE 60, 1925–1937.
1046. Muhammad Tahir, H., Akhtar, S., 2016. Services of DNA barcoding in different fields. Mitochondrial DNA Part A: DNA Mapping, Sequencing, and Analysis 27, 4463–4474. https://doi.org/10.3109/19401736.2015.1089572
1047. Multiphase metering in Machar and Monan fields, 2000. . Journal of Petroleum Technology 52, 37–38.
1048. Mumby, P.J., Edwards, A.J., 2002. Mapping marine environments with IKONOS imagery: enhanced spatial resolution can deliver greater thematic accuracy. Remote Sensing of Environment 82, 248–257. https://doi.org/10.1016/s0034-4257(02)00041-x
1049. Munday, E., 2009. Using sonar to investigate interactions between marine life, subsea turbines. Sea Technology 50, 15–18.
1050. Munksgaard, N.C., Moir, C.M., Parry, D.L., 2002. Bio-monitoring using lead isotope ratios in seagrass and oysters. Marine Technology Society Journal 36, 52–54. https://doi.org/10.4031/002533202787914269
1051. Munoz-Perez, J.J., Mas, J.M.G., Naranjo, J.M., Torres, E., Fages, L., 2000. Position and monitoring of anti-trawling reefs in the Cape of Trafalgar (Gulf of Cadiz, sw Spain). Bulletin of Marine Science 67, 761–772.
1052. Murat, D., Faye, S.C., Escudier, P., Richard, J., 2006. The contribution of space radar altimetry to ocean monitoring. Alcatel Telecommunications Review 129–135.
1053. Murphy, H.M., Jenkins, G.P., 2010. Observational methods used in marine spatial monitoring of fishes and associated habitats: A review. Marine and Freshwater Research 61, 236–252. https://doi.org/10.1071/MF09068
1054. Murray, C., Sohngen, B., Pendleton, L., 2001. Valuing water quality advisories and beach amenities in the Great Lakes. Water Resources Research 37, 2583–2590. https://doi.org/10.1029/2001WR000409
1055. Murray, S.A., Wiese, M., Stüken, A., Brett, S., Kellmann, R., Hallegraeff, G., Neilan, B.A., 2011. SxtA-based quantitative molecular assay to identify saxitoxin-producing harmful algal blooms in marine waters. Applied and Environmental Microbiology 77, 7050–7057. https://doi.org/10.1128/AEM.05308-11
1056. Musco, L., Terlizzi, A., Licciano, M., Giangrande, A., 2009. Taxonomic structure and the effectiveness of surrogates in environmental monitoring: a lesson from polychaetes. Marine Ecology Progress Series 383, 199–210. https://doi.org/10.3354/meps07989
1057. Musil, C.F., Milton, S.J., Davis, G.W., 2005. The threat of alien invasive grasses to lowland Cape floral diversity: an empirical appraisal of the effectiveness of practical control strategies. South African Journal of Science 101, 337–344.
1058. Mustajärvi, L., Eriksson-Wiklund, A.K., Gorokhova, E., Jahnke, A., Sobek, A., 2017. Transferring mixtures of chemicals from sediment to a bioassay using silicone-based passive sampling and dosing. Environmental Science: Processes and Impacts 19, 1404–1413. https://doi.org/10.1039/c7em00228a
1059. Muthivhi, R., Parani, S., May, B., Oluwafemi, O.S., 2018. Green synthesis of gelatin-noble metal polymer nanocomposites for sensing of Hg2+ ions in aqueous media. Nano-Structures and Nano-Objects 13, 132–138. https://doi.org/10.1016/j.nanoso.2017.12.008
1060. Mutiti, S., Levy, J., Mutiti, C., Gaturu, N.S., 2010. Assessing ground water development potential using Landsat imagery. Ground Water 48, 295–305. https://doi.org/10.1111/j.1745-6584.2008.00524.x
1061. Myers, J.T., Stockwell, J.D., Yule, D.L., Black, J.A., 2008. Evaluating sampling strategies for larval cisco (Coregonus artedi). Journal of Great Lakes Research 34, 245–252. https://doi.org/10.3394/0380-1330(2008)34[245:ESSFLC]2.0.CO;2
1062. Müller-Petke, M., Walbrecker, J.O., Knight, R., 2013. The inversion of surface-NMR T1 data for improved aquifer characterization. Geophysics 78, EN83–EN94. https://doi.org/10.1190/GEO2013-0035.1
1063. Naess, O.E., 2006. Repeatability and 4D seismic acquisition, SEG Technical Program Expanded Abstracts. https://doi.org/10.1190/1.2370217
1064. Naftz, D.L., Yahnke, J., Miller, J., Noyes, S., 2005. Selenium mobilization during a flood experiment in a contaminated wetland: Stewart Lake Waterfowl Management Area, Utah. Applied Geochemistry 20, 569–585. https://doi.org/10.1016/j.apgeochem.2004.09.009
1065. Nagai, S., Itakura, S., 2012. Specific detection of the toxic dinoflagellates Alexandrium tamarense and Alexandrium catenella from single vegetative cells by a loop-mediated isothermal amplification method. Marine Genomics 7, 43–49. https://doi.org/10.1016/j.margen.2012.03.001
1066. Nage, D.D., 2016. Thermal spray aluminum coatings for splash zone structures-Part II. Advanced Materials and Processes 174, 36–39.
1067. Narayana, A.C., 2016. Shoreline changes, Encyclopedia of Earth Sciences Series. https://doi.org/10.1007/978-94-017-8801-4_118
1068. Nauen, C.E., 2006. Implementing the WSSD decision of restoring marine ecosystems by 2015 - scientific information support in the public domain. Marine Policy 30, 455–461. https://doi.org/10.1016/j.marpol.2004.11.003
1069. Nayak, B.K., Anitha, K., 2015. Comparative study of antibiotic potency of AgNPs synthesized from two species of aspergilli with the drug; Ampicillin. Der Pharmacia Lettre 7, 198–201.
1070. Negi, H.R., 2001. Diversity and dominance of liverworts of Chopta-Tunganath in the Garhwal Himalaya. International Journal of Ecology and Environmental Sciences 27, 13–21.
1071. Neher, D.A., Wu, J., Barbercheck, M.E., Anas, O., 2005. Ecosystem type affects interpretation of soil nematode community measures. Applied Soil Ecology 30, 47–64. https://doi.org/10.1016/j.apsoil.2005.01.002
1072. Nelson, E.C., Batalden, P.B., Homa, K., Godfrey, M.M., Campbell, C., Headrick, L.A., Huber, T.P., Mohr, J.J., Wasson, J.H., 2003. Microsystems in health care: Part 2. Creating a rich information environment. Joint Commission journal on quality and safety 29, 5–15. https://doi.org/10.1016/S1549-3741(03)29002-X
1073. Nendza, M., 2002. Inventory of marine biotest methods for the evaluation of dredged material and sediments. Chemosphere 48, 865–883. https://doi.org/10.1016/S0045-6535(02)00003-6
1074. Neris, J., Doerr, S.H., Tejedor, M., Jiménez, C., Hernández-Moreno, J.M., 2014. Thermal analysis as a predictor for hydrological parameters of fire-affected soils. Geoderma 235–236, 240–249. https://doi.org/10.1016/j.geoderma.2014.07.018
1075. Neto, A.A., Mota, B.B., Belem, A.L., Albuquerque, A.L., Capilla, R., 2016. Seismic peak amplitude as a predictor of TOC content in shallow marine sediments. Geo-Marine Letters 36, 395–403. https://doi.org/10.1007/s00367-016-0449-3
1076. Nevers, M.B., Byappanahalli, M.N., Whitman, R.L., 2013. Choices in recreational water quality monitoring: New opportunities and health risk trade-offs. Environmental Science and Technology 47, 3073–3081. https://doi.org/10.1021/es304408y
1077. Ng, C., Malig, B., Hasheminassab, S., Sioutas, C., Basu, R., Ebisu, K., 2017. Source apportionment of fine particulate matter and risk of term low birth weight in California: Exploring modification by region and maternal characteristics. Science of the Total Environment 605–606, 647–654. https://doi.org/10.1016/j.scitotenv.2017.06.053
1078. Ngugi, H.N., Mutuku, F.M., Ndenga, B.A., Musunzaji, P.S., Mbakaya, J.O., Aswani, P., Irungu, L.W., Mukoko, D., Vulule, J., Kitron, U., LaBeaud, A.D., 2017. Characterization and productivity profiles of Aedes aegypti (L.) breeding habitats across rural and urban landscapes in western and coastal Kenya. Parasites and Vectors 10. https://doi.org/10.1186/s13071-017-2271-9
1079. Ni, C., Ma, X., 2018. Prediction of wave power generation using a Convolutional Neural Network with multiple inputs. Energies 11. https://doi.org/10.3390/en11082097
1080. Nicholls-Lee, R.F., Turnock, S.R., 2008. Tidal energy extraction: Renewable, sustainable and predictable. Science Progress 91, 81–111. https://doi.org/10.3184/003685008X285582
1081. Nichols, O.C., Lind, H., Baldwin, J., Jackett, A.R., Borrelli, M., Small Jr, P.A., 2011. Site selection for sustainable shellfish aquaculture development: A practical mapping approach. Journal of Ocean Technology 6, 59–70.
1082. Nicholson, S., 2001. Ecocytological and toxicological responses to copper in Perna viridis (L.) (Bivalvia: Mytilidae) haemocyte lysosomal membranes. Chemosphere 45, 399–407. https://doi.org/10.1016/S0045-6535(01)00039-X
1083. Nielsen, S.S., Kjeldsen, P., Jakobsen, R., 2016. Full scale amendment of a contaminated wood impregnation site with iron water treatment residues. Frontiers of Environmental Science & Engineering 10. https://doi.org/10.1007/s11783-016-0842-1
1084. Nieminen, E., Hyytiainen, K., Lindroos, M., 2017. Economic and policy considerations regarding hydropower and migratory fish. Fish and Fisheries 18, 54–78. https://doi.org/10.1111/faf.12167
1085. Nipper, M., 2000. Current approaches and future directions for contaminant-related impact assessments in coastal environments: Brazsian perspective. Aquatic Ecosystem Health and Management 3, 433–447. https://doi.org/10.1080/14634980008650680
1086. Nirmala, R., 2015. Road crash - analysis and alleviation measures. International Journal of Engineering and Technology 7, 997–1002.
1087. Niu, Z.G., Gong, P., Cheng, X., Guo, J.H., Wang, L., Huang, H.B., Shen, S.Q., Wu, Y.Z., Wang, X.F., Wang, X.W., Ying, Q., Liang, L., Zhang, L.N., Wang, L., Yao, Q., Yang, Z.Z., Guo, Z.Q., Dai, Y.J., 2009. Geographical characteristics of China’s wetlands derived from remotely sensed data. Science in China, Series D: Earth Sciences 52, 723–738. https://doi.org/10.1007/s11430-009-0075-2
1088. Noël, J.J., Ahluwalia, G.K., 2016. Electrochemical sensors, in: Applications of Chalcogenides: S, Se, and Te. pp. 235–261. https://doi.org/10.1007/978-3-319-41190-3_6
1089. Norris, P.M., da Silva, A.M., 2016. Monte Carlo Bayesian inference on a statistical model of sub-gridcolumn moisture variability using high-resolution cloud observations. Part 2: Sensitivity tests and results. Quarterly Journal of the Royal Meteorological Society 142, 2528–2540. https://doi.org/10.1002/qj.2844
1090. Norton, J.W., 2009. Decentralized systems. Water Environment Research 81, 1440–1450. https://doi.org/10.2175/106143009X12445568399893
1091. Novák, P., Witteveen, C., 2013. Reconfiguration of large-scale surveillance systems, Lecture Notes in Computer Science (including subseries Lecture Notes in Artificial Intelligence and Lecture Notes in Bioinformatics). https://doi.org/10.1007/978-3-642-40624-9_20
1092. Novikmec, M., Hamerlik, L., Kocicky, D., Hrivnak, R., Kochjarova, J., Ot’ahel’ova, H., Pal’ove-Balang, P., Svitok, M., 2016. Ponds and their catchments: size relationships and influence of land use across multiple spatial scales. Hydrobiologia 774, 155–166. https://doi.org/10.1007/s10750-015-2514-8
1093. Novoa, S., Chust, G., Froidefond, J.M., Petus, C., Franco, J., Orive, E., Seoane, S., Borja, A., 2012. Water quality monitoring in Basque coastal areas using local chlorophyll- a algorithm and MERIS images. Journal of Applied Remote Sensing 6. https://doi.org/10.1117/1.JRS.6.063519
1094. Noyer, C., Abot, A., Trouilh, L., Leberre, V.A., Dreanno, C., 2015. Phytochip: Development of a DNA-microarray for rapid and accurate identification of Pseudo-nitzschia spp and other harmful algal species. Journal of Microbiological Methods 112, 55–66. https://doi.org/10.1016/j.mimet.2015.03.002
1095. Nygård, H., Oinonen, S., Hällfors, H.A., Lehtiniemi, M., Rantajärvi, E., Uusitalo, L., 2016. Price vs. value of marine monitoring. Frontiers in Marine Science 3. https://doi.org/10.3389/fmars.2016.00205
1096. Nyman, M.T., Korhola, A.A., 2005. Chironomid-based classification of lakes in western Finnish Lapland. Boreal Environment Research 10, 239–254.
1097. O’Driscoll, R.L., Macaulay, G.J., 2005. Using fish-processing time to carry out acoustic surveys from commercial vessels. ICES Journal of Marine Science 62, 295–305. https://doi.org/10.1016/j.icesjms.2004.11.013
1098. Oddy, D.M., Stolen, E.D., Gann, S.L., Legare, S.A., Weiss, S.K., Holloway-Adkins, K.G., Oddy, D.M., Stolen, E.D., Legare, S.A., Weiss, S.K., Holloway-Adkins, K.G., Gann, S.L., 2018. Increasing detection by reducing disturbance and excluding nontarget small mammal species: An occupancy study approach. Journal of Fish and Wildlife Management 9, 383–392. https://doi.org/10.3996/072017-JFWM-057
1099. Oinonen, S., Hyytiäinen, K., Ahlvik, L., Laamanen, M., Lehtoranta, V., Salojärvi, J., Virtanen, J., 2016. Cost-effective marine protection - A pragmatic approach. PLoS ONE 11. https://doi.org/10.1371/journal.pone.0147085
1100. Okes, N.C., O’Riain, M.J., 2017. Otter occupancy in the Cape Peninsula: Estimating the probability of river habitat use by Cape clawless otters, Aonyx capensis, across a gradient of human influence. Aquatic Conservation: Marine and Freshwater Ecosystems 27, 706–716. https://doi.org/10.1002/aqc.2738
1101. Oliveira, J.M., Ferreira, M.T., Pinheiro, A.N., Bochechas, J.H., 2004. A simple method for assessing minimum flows in regulated rivers: The case of sea lamprey reproduction. Aquatic Conservation: Marine and Freshwater Ecosystems 14, 481–489. https://doi.org/10.1002/aqc.634
1102. Oloo, O.J., 2017. ASSESSING THE ACCURACY OF REMOTE SENSING TECHNIQUES IN VEGETATION FRACTIONS ESTIMATION. South African Journal of Geomatics 6, 106–129. https://doi.org/10.4314/sajg.v6i1.7
1103. Omrane, F., Gargouri, I., Khadhraoui, M., Elleuch, B., Zmirou-Navier, D., 2018. Risk assessment of occupational exposure to heavy metal mixtures: a study protocol. Bmc Public Health 18. https://doi.org/10.1186/s12889-018-5191-5
1104. Orfanidis, S., Pinna, M., Sabetta, L., Stamatis, N., Nakou, K., 2008. Variation of structural and functional metrics in macrophyte communities within two habitats of eastern Mediterranean coastal lagoons: Natural versus human effects. Aquatic Conservation: Marine and Freshwater Ecosystems 18, S45–S61. https://doi.org/10.1002/aqc.957
1105. Orlando-Bonaca, M., Lipej, L., Francé, J., 2016. The most suitable time and depth to sample cymodocea nodosa (Ucria) ascherson meadows in the shallow coastal area. Experiences from the northern adriatic sea. Acta Adriatica 57, 251–262.
1106. Orvik, K.A., Skagseth, Ø., 2003. Monitoring the Norwegian Atlantic slope current using a single moored current meter. Continental Shelf Research 23, 159–176. https://doi.org/10.1016/S0278-4343(02)00172-3
1107. Otter, D., Joy, R., Jones, M., Maal, L., 2012. Need for bridge monitoring systems to counter railroad bridge service interruptions, Transportation Research Record. https://doi.org/10.3141/2313-15
1108. Ou-Yang, H., Hua, L., Mo, Q.H., Xu, X.M., 2004. Rapid, accurate genotyping of the common -alpha(4.2) thalassaemia deletion based on the use of denaturing HPLC. Journal of Clinical Pathology 57, 159–163. https://doi.org/10.1136/jcp.2003.011130
1109. Ou-Yang, H., Hua, L., Mo, Q.H., Xu, X.M., 2004. Rapid, accurate genotyping of the common -α4.2 thalassaemia deletion based on the use of denaturing HPLC. Journal of Clinical Pathology 57, 159–163. https://doi.org/10.1136/jcp.2003.011130
1110. Ouyang, H., Shu, Q., Wang, W., Wang, Z., Yang, S., Wang, L., Fu, Z., 2016. An ultra-facile and label-free immunoassay strategy for detection of copper (II) utilizing chemiluminescence self-enhancement of Cu (II)-ethylenediaminetetraacetate chelate. Biosensors and Bioelectronics 85, 157–163. https://doi.org/10.1016/j.bios.2016.05.007
1111. Ouyang, Y., 2012. Designing a dynamic data-driven application system for estimating real-time load of dissolved organic carbon in a river. Water, Air, and Soil Pollution 223, 5289–5296. https://doi.org/10.1007/s11270-012-1279-1
1112. Ouyang, Y., Leininger, T.D., Hatten, J., 2013. Real-time estimation of TP load in a Mississippi Delta stream using a dynamic data driven application system. Journal of Environmental Management 122, 37–41. https://doi.org/10.1016/j.jenvman.2013.02.047
1113. Ouyang, Y., Luo, S.M., Cui, L.H., Wang, Q., Zhang, J.E., 2011. Estimation of real-time N load in surface water using dynamic data-driven application system. Ecological Engineering 37, 616–621. https://doi.org/10.1016/j.ecoleng.2010.12.023
1114. Owens, G.M., 2007. Analyzing impacts of bioenergy expansion in China using strategic environmental assessment. Management of Environmental Quality: An International Journal 18, 396–412. https://doi.org/10.1108/14777830710753802
1115. Ozsoy-Cicek, B., 2014. OIL SPILL DETECTION FROM RADARSAT-1 SYNTHETIC APERTURE RADAR IMAGERY AT NORTHERN ENTRY OF BOSPORUS STRAIT, TURKEY. Fresenius Environmental Bulletin 23, 2909–2918.
1116. Paerl, H.W., 2006. Assessing and managing nutrient-enhanced eutrophication in estuarine and coastal waters: Interactive effects of human and climatic perturbations. Ecological Engineering 26, 40–54. https://doi.org/10.1016/j.ecoleng.2005.09.006
1117. Pagnucco, K.S., Paszkowski, C.A., Scrimgeour, G.J., 2011. Using cameras to monitor tunnel use by Long-toed Salamanders (Ambystoma macrodactylum): An informative, cost-efficient technique. Herpetological Conservation and Biology 6, 277–286.
1118. Pagola-Carte, S., Saiz-Salinas, J.I., 2000. A pilot study for monitoring the zoobenthic communities on the rocky shores of Abra de Bilbao (northern Spain). Journal of the Marine Biological Association of the United Kingdom 80, 395–406. https://doi.org/10.1017/s0025315400002095
1119. Pagola-Carte, S., Saiz-Salinas, J.I., 2001. Changes in the sublittoral faunal biomass induced by the discharge of a polluted river along the adjacent rocky coast (N. Spain). Marine Ecology Progress Series 212, 13–27. https://doi.org/10.3354/meps212013
1120. Palas, S., Villasante, S., Pita, P., 2017. Combining fishers’ knowledge and cost-effective monitoring tools in the management of marine recreational fisheries: A case study of the squid and cuttlefish fishery of the Ria of Vigo (NW Spain). Fisheries Management and Ecology 24, 469–477. https://doi.org/10.1111/fme.12255
1121. Palas, S., Villasante, S., Pita, P., 2017. Combining fishers’ knowledge and cost-effective monitoring tools in the management of marine recreational fisheries: A case study of the squid and cuttlefish fishery of the Ría of Vigo (NW Spain). Fisheries Management and Ecology 24, 469–477. https://doi.org/10.1111/fme.12255
1122. Palm-Forster, L.H., Swinton, S.M., Lupi, F., Shupp, R.S., 2016. Too burdensome to bid: Transaction costs and pay-for-performance conservation. American Journal of Agricultural Economics 98, 1314–1333. https://doi.org/10.1093/ajae/aaw071
1123. Palomeras, N., Vallicrosa, G., Mallios, A., Bosch, J., Vidal, E., Hurtos, N., Carreras, M., Ridao, P., 2018. AUV homing and docking for remote operations. Ocean Engineering 154, 106–120. https://doi.org/10.1016/j.oceaneng.2018.01.114
1124. Pan, H.F., Long, G.F., Li, Q., Feng, Y.N., Lei, Z.Y., Wei, H.W., Huang, Y.Y., Huang, J.H., Lin, N., Xu, Q.Q., Ling, S.Y., Chen, X.J., Huang, T., 2007. Current status of thalassemia in minority populations in Guangxi, China. Clinical Genetics 71, 419–426. https://doi.org/10.1111/j.1399-0004.2007.00791.x
1125. Pan, Y., Wang, H., Gu, Z., Xiong, G., Yi, F., 2010. Accumulation and translocation of heavy metals by macrophytes. Shengtai Xuebao/ Acta Ecologica Sinica 30, 6430–6441.
1126. Panahbehagh, B., Smith, D.R., 2017. Group inverse sampling: An economical approach to inverse sampling. Environmetrics 28. https://doi.org/10.1002/env.2459
1127. Panda, S.S., Garg, V., Chaubey, I., 2004. Artificial Neural Networks Application in Lake Water Quality Estimation Using Satellite Imagery. Journal of Environmental Informatics 4, 65–74. https://doi.org/10.3808/jei.200400038
1128. Pandey, J.S., Joseph, V., Shanker, R., Ghosh, T.K., 2001. Watershed management and vegetative strips for sediment load reduction - Impact of farmland runoff. International Water and Irrigation 21, 34–37.
1129. Pandit, S.A., Natarajan, V., Raju, R.D., 2002. Exploration for uranium in the bhima BASIN in parts of Karnataka, India. Exploration and Research for Atomic Minerals 14, 59–78.
1130. Pannwitz, G., Wolf, C., Harder, T., 2009. Active surveillance for avian influenza virus infection in wild birds by analysis of avian fecal samples from the environment. Journal of Wildlife Diseases 45, 512–518. https://doi.org/10.7589/0090-3558-45.2.512
1131. Pantazis, P.A., Kelly, M.S., Connolly, J.G., Black, K.D., 2000. Effect of artificial diets on growth, lipid utilization, and gonad biochemistry in the adult sea urchin Psammechinus miliaris. Journal of Shellfish Research 19, 995–1001.
1132. Panteleev, G., Yaremchuk, M., Stroh, J., Posey, P., Hebert, D., Nechaev, D.A., 2015. Optimization of the high-frequency radar sites in the Bering Strait region. Journal of Atmospheric and Oceanic Technology 32, 297–309. https://doi.org/10.1175/JTECH-D-14-00071.1
1133. Papa, M.N., Sarno, L., Vitiello, F.S., Medina, V., 2018. Application of the 2D depth-averaged model, FLATModel, to pumiceous debris flows in the Amalfi Coast. Water (Switzerland) 10. https://doi.org/10.3390/w10091159
1134. Pardos, M., Benninghoff, C., Thomas, R.L., Dobrowolski, J., Dominik, J., 2000. Water ecotoxicity studies in Cracow (Poland) using Hydra attenuata, Selenastrum capricornutum, and Microtox® toxicity tests. Lakes and Reservoirs: Research and Management 5, 75–81. https://doi.org/10.1046/j.1440-1770.2000.00099.x
1135. Park, T., Bowker, J.M., Leeworthy, V.R., 2002. Valuing snorkeling visits to the Florida Keys with stated and revealed preference models. Journal of Environmental Management 64, 301–312. https://doi.org/10.1006/jema.2002.0552
1136. Park, Y.S., Engel, B.A., Kim, J., Theller, L., Chaubey, I., Merwade, V., Lim, K.J., 2015. A web tool for STORET/WQX water quality data retrieval and Best Management Practice scenario suggestion. Journal of Environmental Management 150, 21–27. https://doi.org/10.1016/j.jenvman.2014.11.006
1137. Parker, R., Bolam, T., Barry, J., Mason, C., Kröger, S., Warford, L., Silburn, B., Sivyer, D., Birchenough, S., Mayes, A., Fones, G.R., 2017. The application of Diffusive Gradients in Thin Films (DGT) for improved understanding of metal behaviour at marine disposal sites. Science of the Total Environment 575, 1074–1086. https://doi.org/10.1016/j.scitotenv.2016.09.183
1138. Partridge, G.J., Ginbey, B.M., Woolley, L.D., Fairclough, D.V., Crisafulli, B., Chaplin, J., Prokop, N., Dias, J., Bertram, A., Jenkins, G.I., 2017. Development of techniques for the collection and culture of wild-caught fertilised snapper (Chrysophrys auratus) eggs for stock enhancement purposes. Fisheries Research 186, 524–530. https://doi.org/10.1016/j.fishres.2016.08.025
1139. Pascual, M., Borja, A., Franco, J., Burdon, D., Atkins, J.P., Elliott, M., 2012. What are the costs and benefits of biodiversity recovery in a highly polluted estuary? Water Research 46, 205–217. https://doi.org/10.1016/j.watres.2011.10.053
1140. Pasquaud, S., Costa, J.L., Costa, M.J., Cabral, H., 2012. Concordance between expert judgment and fish-based multimetric indices in the assessment of estuarine waters ecological quality. Ocean and Coastal Management 69, 143–150. https://doi.org/10.1016/j.ocecoaman.2012.07.009
1141. Pearlman, J., Zielinski, O., 2017. A new generation of optical systems for ocean monitoring matrix fluorescence for multifunctional ocean sensing. Sea Technology 58, 30–33.
1142. Peay, S., 2004. Keynote - A cost-led evaluation of survey methods and monitoring for white-clawed crayfish - Lessons from the UK. Bulletin Francais De La Peche Et De La Pisciculture 335–352. https://doi.org/10.1051/kmae:2004008
1143. Pei, H., Jiang, L., 2018. Mixing Seawater with a Little Wastewater to Produce Bioenergy from Limnetic Algae. Trends in Biotechnology 36, 480–483. https://doi.org/10.1016/j.tibtech.2017.12.002
1144. Peirce, C., Day, A.J., 2002. Ocean-bottom seismograph tomographic experiments - A consideration of acquisition geometries vs. resources. Geophysical Journal International 151, 543–565. https://doi.org/10.1046/j.1365-246X.2002.01783.x
1145. Pelage, L., Domalain, G., Lira, A.S., Travassos, P., Fredou, T., 2019. Coastal Land Use in Northeast Brazil: Mangrove Coverage Evolution Over Three Decades. Tropical Conservation Science 12. https://doi.org/10.1177/1940082918822411
1146. Pelland, N.A., Eriksen, C.C., Emerson, S.R., Cronin, M.F., 2018. Seaglider Surveys at Ocean Station Papa: Oxygen Kinematics and Upper-Ocean Metabolism. Journal of Geophysical Research-Oceans 123, 6408–6427. https://doi.org/10.1029/2018jc014091
1147. Pelletier, D., Leleu, K., Mou-Tham, G., Guillemot, N., Chabanet, P., 2011. Comparison of visual census and high definition video transects for monitoring coral reef fish assemblages. Fisheries Research 107, 84–93. https://doi.org/10.1016/j.fishres.2010.10.011
1148. Peltier, H., Baagøe, H.J., Camphuysen, K.C.J., Czeck, R., Dabin, W., Daniel, P., Deaville, R., Haelters, J., Jauniaux, T., Jensen, L.F., Jepson, P.D., Keijl, G.O., Siebert, U., Van Canneyt, O., Ridoux, V., 2013. The Stranding Anomaly as Population Indicator: The Case of Harbour Porpoise Phocoena phocoena in North-Western Europe. PLoS ONE 8. https://doi.org/10.1371/journal.pone.0062180
1149. Peltier, H., Ridoux, V., 2015. Marine megavertebrates adrift: A framework for the interpretation of stranding data in perspective of the European Marine Strategy Framework Directive and other regional agreements. Environmental Science and Policy 54, 240–247. https://doi.org/10.1016/j.envsci.2015.07.013
1150. Peñaloza, C.L., Kendall, W.L., Langtimm, C.A., 2014. Reducing bias in survival under nonrandom temporary emigration. Ecological Applications 24, 1155–1166. https://doi.org/10.1890/13-0558.1
1151. Peng, J.T., Zhu, X.D., Sun, X., Song, X.W., 2018. Identifying external nutrient reduction requirements and potential in the hypereutrophic Lake Taihu Basin, China. Environmental Science and Pollution Research 25, 10014–10028. https://doi.org/10.1007/s11356-018-1250-9
1152. Pepper, M.A., Herrmann, V., Hines, J.E., Nichols, J.D., Kendrot, S.R., 2017. Evaluation of nutria (Myocastor coypus) detection methods in Maryland, USA. Biological Invasions 19, 831–841. https://doi.org/10.1007/s10530-016-1312-1
1153. Pereira, C.D.S., Martin-Diaz, M.L., Zanette, J., Cesar, A., Choueri, R.B., Abessa, D.M.D., Catharino, M.G.M., Vasconcellos, M.B.A., Bainy, A.C.D., de Sousa, E., Del Valls, T.A., 2011. Integrated biomarker responses as environmental status descriptors of a coastal zone (Sao Paulo, Brazil). Ecotoxicology and Environmental Safety 74, 1257–1264. https://doi.org/10.1016/j.ecoenv.2011.02.019
1154. Pereira, C.D.S., Martin-Díaz, M.L., Zanette, J., Cesar, A., Choueri, R.B., Abessa, D.M.D.S., Catharino, M.G.M., Vasconcellos, M.B.A., Bainy, A.C.D., de Sousa, E.C.P.M., Del Valls, T.A., 2011. Integrated biomarker responses as environmental status descriptors of a coastal zone (São Paulo, Brazil). Ecotoxicology and Environmental Safety 74, 1257–1264. https://doi.org/10.1016/j.ecoenv.2011.02.019
1155. Perelo, L.W., 2010. Review: In situ and bioremediation of organic pollutants in aquatic sediments. Journal of Hazardous Materials 177, 81–89. https://doi.org/10.1016/j.jhazmat.2009.12.090
1156. Perez, J.C., Alvarez, M.A., Heikkonen, J., Guillen, J., Barbas, T., 2013. The efficiency of using remote sensing for fisheries enforcement: Application to the Mediterranean bluefin tuna fishery. Fisheries Research 147, 24–31. https://doi.org/10.1016/j.fishres.2013.04.008
1157. Pergent, G., Monnier, B., Clabaut, P., Gascon, G., Pergent-Martini, C., Valette-Sansevin, A., 2017. Innovative method for optimizing Side-Scan Sonar mapping: The blind band unveiled. Estuarine, Coastal and Shelf Science 194, 77–83. https://doi.org/10.1016/j.ecss.2017.05.016
1158. Perini, F., Bastianini, M., Capellacci, S., Pugliese, L., DiPoi, E., Cabrini, M., Buratti, S., Marini, M., Penna, A., 2018. Molecular methods for cost-efficient monitoring of HAB (harmful algal bloom) dinoflagellate resting cysts. Marine Pollution Bulletin. https://doi.org/10.1016/j.marpolbul.2018.06.013
1159. Perkol-Finkel, S., Airoldi, L., 2010. Loss and recovery potential of marine habitats: An experimental study of factors maintaining resilience in subtidal algal forests at the Adriatic Sea. PLoS ONE 5. https://doi.org/10.1371/journal.pone.0010791
1160. Persson, J., Nilsson, M., Bigler, C., Brooks, S.J., Renberg, I., 2007. Near-infrared spectroscopy (NIRS) of epilithic material in streams has a potential for monitoring impact from mining. Environmental Science and Technology 41, 2874–2880. https://doi.org/10.1021/es062329b
1161. Petelet-Giraud, E., Casanova, J., Chery, L., Négrel, P., Bushaert, S., 2005. Attempt of isotopic characterisation (δ18O and δ 2H) of present rainwater signature using lakes and reservoirs: Application to south-western France. Houille Blanche 57–62.
1162. Petelet-Giraudi, E., Casanova, J., Chery, L., Negrel, P., Bushaert, S., 2005. Attempt of isotopic characterisation (delta O-18 and delta H-2) of present rainwater signature using lakes and reservoirs: application to south-western France. Houille Blanche-Revue Internationale De L Eau 57–62. https://doi.org/10.1051/lhb:200502008
1163. Peters, L., Spatharis, S., Dario, M.A., Dwyer, T., Roca, I.J.T., Kintner, A., Kanstad-Hanssen, Ø., Llewellyn, M.S., Praebel, K., 2018. Environmental DNA: A new low-cost monitoring tool for pathogens in salmonid aquaculture. Frontiers in Microbiology 9. https://doi.org/10.3389/fmicb.2018.03009
1164. Petersen, J.D., 2005. Speech on occasion of the 2004 Swedish Baltic Sea Water Award, Water Science and Technology.
1165. Petersen, J.K., Hasler, B., Timmermann, K., Nielsen, P., Torring, D.B., Larsen, M.M., Holmer, M., 2014. Mussels as a tool for mitigation of nutrients in the marine environment. Marine Pollution Bulletin 82, 137–143. https://doi.org/10.1016/j.marpolbul.2014.03.006
1166. Petersen, W., Schroeder, F., Engelke, C., 2006. FerryBox: A mature system for operational monitoring. Sea Technology 47, 53–57.
1167. Peterson, E.E., Urquhart, N.S., 2006. Predicting water quality impaired stream segments using landscape-scale data and a regional geostatistical model: A case study in maryland. Environmental Monitoring and Assessment 121, 615–638. https://doi.org/10.1007/s10661-005-9163-8
1168. Pethybridge, H.R., Nichols, P.D., Virtue, P., Jackson, G.D., 2013. The foraging ecology of an oceanic squid, Todarodes filippovae: The use of signature lipid profiling to monitor ecosystem change. Deep-Sea Research Part II: Topical Studies in Oceanography 95, 119–128. https://doi.org/10.1016/j.dsr2.2012.07.025
1169. Petihakis, G., Perivoliotis, L., Korres, G., Ballas, D., Frangoulis, C., Pagonis, P., Ntoumas, M., Pettas, M., Chalkiopoulos, A., Sotiropoulou, M., Bekiari, M., Kalampokis, A., Ravdas, M., Bourma, E., Christodoulaki, S., Zacharioudaki, A., Kassis, D., Potiris, E., Triantafyllou, G., Tsiaras, K., Krasakopoulou, E., Velanas, S., Zisis, N., 2018. An integrated open-coastal biogeochemistry, ecosystem and biodiversity observatory of the eastern Mediterranean - The Cretan Sea component of the POSEIDON system. Ocean Science 14, 1223–1245. https://doi.org/10.5194/os-14-1223-2018
1170. Piacenza, S.E., Richards, P.M., Heppell, S.S., 2017. An agent-based model to evaluate recovery times and monitoring strategies to increase accuracy of sea turtle population assessments. Ecological Modelling 358, 25–39. https://doi.org/10.1016/j.ecolmodel.2017.05.013
1171. Picard, G., Arnaud, L., Panel, J.M., Morin, S., 2016. Design of a scanning laser meter for monitoring the spatio-temporal evolution of snow depth and its application in the Alps and in Antarctica. Cryosphere 10, 1495–1511. https://doi.org/10.5194/tc-10-1495-2016
1172. Pickerell, C.H., Schott, S., Wyllie-Echeverria, S., 2005. Buoy-deployed seeding: Demonstration of a new eelgrass (Zostera marina L.) planting method. Ecological Engineering 25, 127–136. https://doi.org/10.1016/j.ecoleng.2005.03.005
1173. Piercy, J.J.B., Codling, E.A., Hill, A.J., Smith, D.J., Simpson, S.D., 2014. Habitat quality affects sound production and likely distance of detection on coral reefs. Marine Ecology Progress Series 516, 35–47. https://doi.org/10.3354/meps10986
1174. Piermattei, V., Madonia, A., Bonamano, S., Martellucci, R., Bruzzone, G., Ferretti, R., Odetti, A., Azzaro, M., Zappalà, G., Marcelli, M., 2018. Cost-effective technologies to study the arctic ocean environment†. Sensors (Switzerland) 18. https://doi.org/10.3390/s18072257
1175. Pierpoint, C., Allan, L., Arnold, H., Evans, P., Perry, S., Wilberforce, L., Baxter, J., 2009. Monitoring important coastal sites for bottlenose dolphin in Cardigan bay, UK. Journal of the Marine Biological Association of the United Kingdom 89, 1033–1043. https://doi.org/10.1017/S0025315409000885
1176. Piñeiro-Corbeira, C., Barreiro, R., Cremades, J., 2016. Decadal changes in the distribution of common intertidal seaweeds in Galicia (NW Iberia). Marine Environmental Research 113, 106–115. https://doi.org/10.1016/j.marenvres.2015.11.012
1177. Pinna, M., Marini, G., Mancinelli, G., Basset, A., 2014. Influence of sampling effort on ecological descriptors and indicators in perturbed and unperturbed conditions: A study case using benthic macroinvertebrates in Mediterranean transitional waters. Ecological Indicators 37, 27–39. https://doi.org/10.1016/j.ecolind.2013.09.038
1178. Pinna, M., Marini, G., Rosati, I., Neto, J.M., Patrício, J., Marques, J.C., Basset, A., 2013. The usefulness of large body-size macroinvertebrates in the rapid ecological assessment of Mediterranean lagoons. Ecological Indicators 29, 48–61. https://doi.org/10.1016/j.ecolind.2012.12.011
1179. Pirotta, V., Smith, A., Ostrowski, M., Russell, D., Jonsen, I.D., Grech, A., Harcourt, R., 2017. An economical Custom-Built drone for assessing whale health. Frontiers in Marine Science 4. https://doi.org/10.3389/fmars.2017.00425
1180. Pitois, S.G., Tilbury, J., Bouch, P., Close, H., Barnett, S., Culverhouse, P.F., 2018. Comparison of a Cost-Effective Integrated Plankton Sampling and Imaging Instrument with Traditiona Systems for Mesozooplankton Sampling in the Celtic Sea. Frontiers in Marine Science 5. https://doi.org/10.3389/fmars.2018.00005
1181. Pitois, S.G., Tilbury, J., Bouch, P., Close, H., Barnett, S., Culverhouse, P.F., 2018. Comparison of a cost-effective integrated plankton sampling and imaging instrument with traditional systems for mesozooplankton sampling in the Celtic Sea. Frontiers in Marine Science 5. https://doi.org/10.3389/fmars.2018.00005
1182. Pittman, S.J., Brown, K.A., 2011. Multi-scale approach for predicting fish species distributions across coral reef seascapes. PLoS ONE 6. https://doi.org/10.1371/journal.pone.0020583
1183. Pla, P., 2014. Cover story: SeaExplorer: Bags of potential. International Ocean Systems 18.
1184. Polak-Juszczak, L., 2012. Bioaccumulation of mercury in the trophic chain of flatfish from the Baltic Sea. Chemosphere 89, 585–591. https://doi.org/10.1016/j.chemosphere.2012.05.057
1185. Polemio, M., Dragone, V., Limoni, P.P., 2009. Monitoring and methods to analyse the groundwater quality degradation risk in coastal karstic aquifers (Apulia, Southern Italy). Environmental Geology 58, 299–312. https://doi.org/10.1007/s00254-008-1582-8
1186. Polisar, L., 2004. Tundra to jungle. Control (Chicago, Ill) 17, 51–52.
1187. Pollard, E., Robertson, P., Littlewood, M., Geddes, G., 2014. Insights from archaeological analysis and interpretation of marine data sets to inform marine cultural heritage management and planning of wave and tidal energy development for Orkney Waters and the Pentland Firth, NE Scotland. Ocean & Coastal Management 99, 39–51. https://doi.org/10.1016/j.ocecoaman.2014.05.012
1188. Pollard, P.C., 2012. Fluorescence instrument for in situ monitoring of viral abundance in water, wastewater and recycled water. Journal of Virological Methods 181, 97–102. https://doi.org/10.1016/j.jviromet.2012.01.021
1189. Pollom, R.A., Rose, G.A., 2015. Size-based hydroacoustic measures of within-season fish abundance in a boreal freshwater ecosystem. PLoS ONE 10. https://doi.org/10.1371/journal.pone.0124799
1190. Ponti, M., Abbiati, M., 2004. Quality assessment of transitional waters using a benthic biotic index: The case study of the Pialassa Baiona (northern Adriatic Sea). Aquatic Conservation: Marine and Freshwater Ecosystems 14, S31–S41. https://doi.org/10.1002/aqc.648
1191. Popescu, G., Iordan, D., 2018. AN OVERALL VIEW OF LIDAR AND SONAR SYSTEMS USED IN GEOMATICS APPLICATIONS FOR HYDROLOGY. Scientific Papers-Series E-Land Reclamation Earth Observation & Surveying Environmental Engineering 7, 174–181.
1192. Porst, G., Bader, S., Münch, E., Pusch, M., 2012. Sampling approaches for the assessment of shoreline development based on littoral macroinvertebrates: The case of Lake Werbellin, Germany. Fundamental and Applied Limnology 180, 123–131. https://doi.org/10.1127/1863-9135/2012/0193
1193. Porst, G., Irvine, K., 2009. Distinctiveness of macroinvertebrate communities in turloughs (temporary ponds) and their response to environmental variables. Aquatic Conservation-Marine and Freshwater Ecosystems 19, 456–465. https://doi.org/10.1002/aqc.1016
1194. Porst, G., Miler, O., Donohue, L., Jurca, T., Pilotto, F., Brauns, M., Solimini, A., Pusch, M., 2016. Efficient sampling methodologies for lake littoral invertebrates in compliance with the European Water Framework Directive. Hydrobiologia 767, 207–220. https://doi.org/10.1007/s10750-015-2500-1
1195. Potter, C., 2014. Monitoring the production of Central California coastal rangelands using satellite remote sensing. Journal of Coastal Conservation 18, 213–220. https://doi.org/10.1007/s11852-014-0308-1
1196. Potts, W.M., Childs, A.R., Sauer, W.H.H., Duarte, A.D.C., 2009. Characteristics and economic contribution of a developing recreational fishery in southern Angola. Fisheries Management and Ecology 16, 14–20. https://doi.org/10.1111/j.1365-2400.2008.00617.x
1197. Poussin, J.K., Wouter Botzen, W.J., Aerts, J.C.J.H., 2015. Effectiveness of flood damage mitigation measures: Empirical evidence from French flood disasters. Global Environmental Change 31, 74–84. https://doi.org/10.1016/j.gloenvcha.2014.12.007
1198. Powers, S.P., Hightower, C.L., Marcus Drymon, J., Johnson, M.W., 2012. Age composition and distribution of red drum (Sciaenops ocellatus) in offshore waters of the north central Gulf of Mexico: An evaluation of a stock under a federal harvest moratorium. Fishery Bulletin 110, 283–292.
1199. Prabhudesai, R.G., Joseph, A., Agarvadekar, Y., Dabholkar, N., Mehra, P., Gouveia, A., Tengali, S., Vijaykumar, Parab, A., 2006. Development and implementation of cellular-based real-time reporting and Internet accessible coastal sea-level gauge - A vital tool for monitoring storm surge and tsunami. Current Science 90, 1413–1418.
1200. Prakash, N., Prakash, J., Prakash, A., 2016. Dengue fever: Diagnosis may be skin deep. Indian Journal of Medical Specialities 7, 142–144. https://doi.org/10.1016/j.injms.2016.08.002
1201. Preissler, K., Watzal, A.D., Vences, M., Steinfartz, S., 2019. Detection of elusive fire salamander larvae (Salamandra salamandra) in streams via environmental DNA. Amphibia-Reptilia 40, 55–64. https://doi.org/10.1163/15685381-18000007
1202. Puangpila, C., Jakmunee, J., Pencharee, S., Pensrisirikul, W., 2018. Mobile-phone-based colourimetric analysis for determining nitrite content in water. Environmental Chemistry 15, 403–410. https://doi.org/10.1071/EN18072
1203. Puente, A., Juanes, J.A., 2008. Testing taxonomic resolution, data transformation and selection of species for monitoring macroalgae communities. Estuarine, Coastal and Shelf Science 78, 327–340. https://doi.org/10.1016/j.ecss.2007.12.006
1204. Puhr, K., Schultz, S., Pikelj, K., Petricioli, D., Bakran-Petricioli, T., 2014. The performance, application and integration of various seabed classification systems suitable for mapping Posidonia oceanica (L.) Delile meadows. Science of the Total Environment 470–471, 364–378. https://doi.org/10.1016/j.scitotenv.2013.09.103
1205. Puky, M., 2006. A new, volunteer-based, cost effective method for zoological mapping: The photo identification of freshwater crayfish (Crustacea : Decapoda) species and the importance of volunteers in crayfish research. Bulletin Francais De La Peche Et De La Pisciculture 927–936. https://doi.org/10.1051/kmae:2006032
1206. Puky, M., 2006. Technical note - A new, volunteer-based, cost effective method for zoological mapping: The photo identification of freshwater crayfish (Crustacea: Decapoda) species and the importance of volunteers in crayfish research, BFPP - Bulletin Francais de la Peche et de la Protection des Milieux Aquatiques.
1207. Purcell, S.W., Cheng, Y.W., 2010. Experimental restocking and seasonal visibility of a coral reef gastropod assessed by temporal modelling. Aquatic Biology 9, 227–238. https://doi.org/10.3354/ab00253
1208. Pöthig, R., Behrendt, H., Opitz, D., Furrer, G., 2010. A universal method to assess the potential of phosphorus loss from soil to aquatic ecosystems. Environmental Science and Pollution Research 17, 497–504. https://doi.org/10.1007/s11356-009-0230-5
1209. Pöyhönen, M., 2000. POLSSS: Surveying stakeholders about acceptability of risks and system changes. Safety Science 35, 123–137. https://doi.org/10.1016/S0925-7535(00)00027-8
1210. Qi, Y., Xiu, F.R., Yu, G., Huang, L., Li, B., 2017. Simple and rapid chemiluminescence aptasensor for Hg2+ in contaminated samples: A new signal amplification mechanism. Biosensors and Bioelectronics 87, 439–446. https://doi.org/10.1016/j.bios.2016.08.022
1211. Qing, Z., He, X., Wang, K., Zou, Z., Yang, X., Huang, J., Yan, G., 2012. Colorimetric multiplexed analysis of mercury and silver ions by using a unimolecular DNA probe and unmodified gold nanoparticles. Analytical Methods 4, 3320–3325. https://doi.org/10.1039/c2ay25521a
1212. Quadros, N.D., 2017. Technology in focus: Bathymetric lidar. Hydro International 21, 38–39.
1213. Quinn, T., Hayes, M.P., Dugger, D.J., Hicks, T.L., Hoffmann, A., 2007. Comparison of two techniques for surveying headwater stream amphibians. Journal of Wildlife Management 71, 282–288. https://doi.org/10.2193/2006-342
1214. Raburu, P.O., Masese, F.O., 2012. Development of a fish-based index of biotic integrity (FIBI) for monitoring riverine ecosystems in the Lake Victoria drainage Basin, Kenya. River Research and Applications 28, 23–38. https://doi.org/10.1002/rra.1428
1215. Raburu, P.O., Masese, F.O., 2012. Development of a fish-based index of biotic integrity (FIBI) for monitoring riverine ecosystems in the Lake Victoria drainage Bas Kenya. River Research and Applications 28, 23–38. https://doi.org/10.1002/rra.1428
1216. Rahman, M.R., Thakur, P.K., 2018. Detecting, mapping and analysing of flood water propagation using synthetic aperture radar (SAR) satellite data and GIS: A case study from the Kendrapara District of Orissa State of India. Egyptian Journal of Remote Sensing and Space Science 21, S37–S41. https://doi.org/10.1016/j.ejrs.2017.10.002
1217. Rai, P.K., 2013. Environmental magnetic studies of particulates with special reference to biomagnetic monitoring using roadside plant leaves. Atmospheric Environment 72, 113–129. https://doi.org/10.1016/j.atmosenv.2013.02.041
1218. Raimonet, M., Vilmin, L., Flipo, N., Rocher, V., Laverman, A.M., 2015. Modelling the fate of nitrite in an urbanized river using experimentally obtained nitrifier growth parameters. Water Research 73, 373–387. https://doi.org/10.1016/j.watres.2015.01.026
1219. Rajamani, L., Marsh, H., 2015. Mapping seagrass cost-effectively in the Coral Triangle: Sabah, Malaysia as a case study. Pacific Conservation Biology 21, 113–121. https://doi.org/10.1071/PC14908
1220. Rajendran, V., Murugesan, R., 2011. Performance of the sacrificial galvanic anodes in rehabilitation of marine structure at port blair, Andaman and Nichobar Islands, India. ARPN Journal of Engineering and Applied Sciences 6, 45–55.
1221. Ramakrishna, B., Chang, C.I., Trou, B., Henqemihle, J., 2007. Chesapeake bay water quality monitoring using satellite imagery. International Journal of High Speed Electronics and Systems 17, 681–688. https://doi.org/10.1142/S0129156407004886
1222. Ramanathan, A.A., Aqra, M.W., Al-Rawajfeh, A.E., 2018. Recent advances in 2D nanopores for desalination. Environmental Chemistry Letters 16, 1217–1231. https://doi.org/10.1007/s10311-018-0745-4
1223. Ramírez-Pérez, M., Gonçalves-Araujo, R., Wiegmann, S., Torrecilla, E., Bardaji, R., Röttgers, R., Bracher, A., Piera, J., 2017. Towards cost-effective operational monitoring systems for complex waters: Analyzing small-scale coastal processes with optical transmissometry. PLoS ONE 12. https://doi.org/10.1371/journal.pone.0170706
1224. Ramkilowan, A., Chetty, N., Lysko, M., Griffith, D., 2013. Optical Detectors for Integration into a Low Cost Radiometric Device for In-Water Applications: A Feasibility Study. Journal of the Indian Society of Remote Sensing 41, 531–538. https://doi.org/10.1007/s12524-013-0263-6
1225. Ramsay, P., Murrell, D., Wilkinson, D., 2016. Using multibeam bathymetry, topographic laser scanning & UAS photogrammetry: Marine asset integrity mapping. Hydro International 20, 24–26.
1226. Ramsey, C.L., Griffiths, P.A., Stokes, T.R., 2014. Multi-rotor unmanned aerial vehicles (UAVS) and high-resolution compact digital cameras: A promising new method for monitoring changes to surface karst resources. Acta Carsologica 43, 269–286.
1227. Ransome, E., Geller, J.B., Timmers, M., Leray, M., Mahardini, A., Sembiring, A., Collins, A.G., Meyer, C.P., 2017. The importance of standardization for biodiversity comparisons: A case study using autonomous reef monitoring structures (ARMS) and metabarcoding to measure cryptic diversity on Mo’orea coral reefs, French Polynesia. PLoS ONE 12. https://doi.org/10.1371/journal.pone.0175066
1228. Rashed, M., Atef, A., 2015. Mapping underground utilities within conductive soil using multi-frequency electromagnetic induction and ground penetrating radar. Arabian Journal of Geosciences 8, 2341–2346. https://doi.org/10.1007/s12517-014-1358-2
1229. Rasheed, T., Bilal, M., Nabeel, F., Adeel, M., Iqbal, H.M.N., 2019. Environmentally-related contaminants of high concern: Potential sources and analytical modalities for detection, quantification, and treatment. Environment International 122, 52–66. https://doi.org/10.1016/j.envint.2018.11.038
1230. Rech, S., Borrell Pichs, Y.J., García-Vazquez, E., 2018. Anthropogenic marine litter composition in coastal areas may be a predictor of potentially invasive rafting fauna. PLoS ONE 13. https://doi.org/10.1371/journal.pone.0191859
1231. Reddy, D.V., Bolivar, J.C., Sobhan, K., 2013. Durability-based ranking of typical structural repairs for corrosion-damaged marine piles. Practice Periodical on Structural Design and Construction 18, 225–237. https://doi.org/10.1061/(ASCE)SC.1943-5576.0000157
1232. Redmond, K.J., Berry, M., Pampanin, D.M., Andersen, O.K., 2017. Valve gape behaviour of mussels (Mytilus edulis) exposed to dispersed crude oil as an environmental monitoring endpoint. Marine Pollution Bulletin 117, 330–339. https://doi.org/10.1016/j.marpolbul.2017.02.005
1233. Rees, M.J., Jordan, A., Price, O.F., Coleman, M.A., Davis, A.R., 2014. Abiotic surrogates for temperate rocky reef biodiversity: Implications for marine protected areas. Diversity and Distributions 20, 284–296. https://doi.org/10.1111/ddi.12134
1234. Refsgaard, A., Jacobsen, T., Jacobsen, B., Ørum, J.E., 2007. Integrated modelling of nitrate loads to coastal waters and land rent applied to catchment-scale water management, Water Science and Technology. https://doi.org/10.2166/wst.2007.434
1235. Regmi, G., Indraratna, B., Nghiem, L.D., 2009. Long-term performance of a permeable reactive barrier in acid sulphate soil terrain. Water, Air, and Soil Pollution: Focus 9, 409–419. https://doi.org/10.1007/s11267-009-9230-1
1236. Rehmann, C., 2004. Special report: Plant safety and environment: Consider dual magnetic hermetic bearing housing seals to protect both assets and the environment. Hydrocarbon Processing 83, 58–60.
1237. Rehnstam-Holm, A.S., Hernroth, B., 2005. Shellfish and public health: A Swedish perspective. Ambio 34, 139–144. https://doi.org/10.1639/0044-7447(2005)034[0139:saphas]2.0.co;2
1238. Reitsma, T.W., 2007. To spec or not to spec. Diesel and Gas Turbine Worldwide 39, 84–87.
1239. Renaud, L., Agarwal, N., Richards, D.J., Falcinelli, S., Hazard, E.S., Carnevali, O., Hyde, J., Hardiman, G., 2019. Transcriptomic analysis of short-term 17 alpha-ethynylestradiol exposure in two Californian sentinel fish species sardine (Sardinops sagax) and mackerel (Scomber japonicus). Environmental Pollution 244, 926–937. https://doi.org/10.1016/j.envpol.2018.10.058
1240. Renaud, L., Agarwal, N., Richards, D.J., Falcinelli, S., Hazard, E.S., Carnevali, O., Hyde, J., Hardiman, G., 2019. Transcriptomic analysis of short-term 17Α-ethynylestradiol exposure in two Californian sentinel fish species sardine (Sardinops sagax) and mackerel (Scomber japonicus). Environmental Pollution 926–937. https://doi.org/10.1016/j.envpol.2018.10.058
1241. Reoyo-Prats, B., Aubert, D., Sellier, A., Roig, B., Palacios, C., 2018. Dynamics and sources of pharmaceutically active compounds in a coastal Mediterranean river during heavy rains. Environmental Science and Pollution Research 25, 6107–6121. https://doi.org/10.1007/s11356-017-0880-7
1242. Revollo, N.V., Delrieux, C.A., Perillo, G.M.E., 2016. Automatic methodology for mapping of coastal zones in video sequences. Marine Geology 381, 87–101. https://doi.org/10.1016/j.margeo.2016.08.005
1243. Rey, A., Basurko, O.C., Rodriguez-Ezpeleta, N., 2018. The challenges and promises of genetic approaches for ballast water management. Journal of Sea Research 133, 134–145. https://doi.org/10.1016/j.seares.2017.06.001
1244. Rich, V.I., Pham, V.D., Eppley, J., Shi, Y., DeLong, E.F., 2011. Time-series analyses of Monterey Bay coastal microbial picoplankton using a “genome proxy” microarray. Environmental Microbiology 13, 116–134. https://doi.org/10.1111/j.1462-2920.2010.02314.x
1245. Richards, V., Gregory, D., MacLeod, I., Matthiesen, H., 2012. Reburial and analyses of archaeological remains in the marine environment - Investigations into the effects on metals. Conservation and Management of Archaeological Sites 14, 35–47. https://doi.org/10.1179/1350503312Z.0000000004
1246. Richards, V.L., 2011. In situ preservation and reburial of the ex-slave ship James Matthews. AICCM Bulletin 32, 33–43. https://doi.org/10.1179/bac.2011.32.1.006
1247. Richardson, E.A., Kaiser, M.J., Edwards-Jones, G., Possingham, H.P., 2006. Sensitivity of marine-reserve design to the spatial resolution of socioeconomic data. Conservation Biology 20, 1191–1202. https://doi.org/10.1111/j.1523-1739.2006.00426.x
1248. Richman, N.I., Gibbons, J.M., Turvey, S.T., Akamatsu, T., Ahmed, B., Mahabub, E., Smith, B.D., Jones, J.P.G., 2014. To see or not to see: Investigating detectability of ganges river dolphins using a combined visual-acoustic survey. PLoS ONE 9. https://doi.org/10.1371/journal.pone.0096811
1249. Richmond, S., Stevens, T., 2014. Classifying benthic biotopes on sub-tropical continental shelf reefs: How useful are abiotic surrogates? Estuarine, Coastal and Shelf Science 138, 79–89. https://doi.org/10.1016/j.ecss.2013.12.012
1250. Rickerby, D.G., 2009. Potental application of biosensor networks for monitoring aquatic sytems in support of the water framework directive. Bollettino Di Geofisica Teorica Ed Applicata 50, 341–360.
1251. Ridgway, J., Breward, N., Langston, W.J., Lister, R., Rees, J.G., Rowlatt, S.M., 2003. Distinguishing between natural and anthropogenic sources of metals entering the Irish Sea. Applied Geochemistry 18, 283–309. https://doi.org/10.1016/S0883-2927(02)00126-9
1252. Rihouey, D., Dailloux, D., Nader, J.R., Morichon, D., 2009. Mapping inter-tidal topography from video measurements. Houille Blanche-Revue Internationale De L Eau 32–37. https://doi.org/10.1051/lhb/2009011
1253. Rihouey, D., Dailloux, D., Nader, J.R., Morichon, D., 2009. Mapping inter-tidal topography from video measurments. Houille Blanche 32–37. https://doi.org/10.1051/lhb:2009011
1254. Ringvall, A., Kruys, N., 2005. Sampling of sparse species with probability proportional to prediction. Environmental Monitoring and Assessment 104, 131–146. https://doi.org/10.1007/s10661-005-1599-3
1255. Rishworth, G.M., Tremblay, Y., Green, D.B., Pistorius, P.A., 2014. An automated approach towards measuring time-activity budgets in colonial seabirds. Methods in Ecology and Evolution 5, 854–863. https://doi.org/10.1111/2041-210x.12213
1256. Risk, M.J., Lapointe, B.E., Sherwood, O.A., Bedford, B.J., 2009. The use of δ15N in assessing sewage stress on coral reefs. Marine Pollution Bulletin 58, 793–802. https://doi.org/10.1016/j.marpolbul.2009.02.008
1257. Rivett, M.O., Ellis, P.A., Greswell, R.B., Ward, R.S., Roche, R.S., Cleverly, M.G., Walker, C., Conran, D., Fitzgerald, P.J., Willcox, T., Dowle, J., 2008. Cost-effective mini drive-point piezometers and multilevel samplers for monitoring the hyporheic zone. Quarterly Journal of Engineering Geology and Hydrogeology 41, 49–60. https://doi.org/10.1144/1470-9236/07-012
1258. Robert, K., Jones, D.O.B., Tyler, P.A., Van Rooij, D., Huvenne, V.A.I., 2015. Finding the hotspots within a biodiversity hotspot: Fine-scale biological predictions within a submarine canyon using high-resolution acoustic mapping techniques. Marine Ecology 36, 1256–1276. https://doi.org/10.1111/maec.12228
1259. Robinson, L.M., Gledhill, D.C., Moltschaniwskyj, N.A., Hobday, A.J., Frusher, S., N.Barrett, Stuart-Smith, J., Pecl, G.T., 2015. Rapid assessment of an ocean warming hotspot reveals “high” confidence in potential species’ range extensions. Global Environmental Change 31, 28–37. https://doi.org/10.1016/j.gloenvcha.2014.12.003
1260. Robinson, O.J., Ruiz-Gutierrez, V., Fink, D., 2018. Correcting for bias in distribution modelling for rare species using citizen science data. Diversity and Distributions 24, 460–472. https://doi.org/10.1111/ddi.12698
1261. Robinson, T.P., Wardell-Johnson, G.W., Pracilio, G., Brown, C., Corner, R., van Klinken, R.D., 2016. Testing the discrimination and detection limits of WorldView-2 imagery on a challenging invasive plant target. International Journal of Applied Earth Observation and Geoinformation 44, 23–30. https://doi.org/10.1016/j.jag.2015.07.004
1262. Rodgveller, C.J., 2018. A comparison of methods for classifying female sablefish maturity and skip spawning outside the spawning season. Marine and Coastal Fisheries 10, 563–576. https://doi.org/10.1002/mcf2.10053
1263. Rodriguez, H.G., Popp, J., Maringanti, C., Chaubey, I., 2011. Selection and placement of best management practices used to reduce water quality degradation in Lincoln Lake watershed. Water Resources Research 47. https://doi.org/10.1029/2009WR008549
1264. Rodríguez-Rodríguez, D., Rees, S.E., Rodwell, L.D., Attrill, M.J., 2015. IMPASEA: A methodological framework to monitor and assess the socioeconomic effects of marine protected areas. An English Channel case study. Environmental Science and Policy 54, 44–51. https://doi.org/10.1016/j.envsci.2015.05.019
1265. Roelfsema, C.M., Phinn, S.R., Dennison, W.C., Dekker, A.G., Brando, V.E., 2006. Monitoring toxic cyanobacteria Lyngbya majuscula (Gomont) in Moreton Bay, Australia by integrating satellite image data and field mapping. Harmful Algae 5, 45–56. https://doi.org/10.1016/j.hal.2005.05.001
1266. Rogers, P.J., Dake, N., Dussart, G.B.J., 2004. Ulva problem in Kent: 1973-2003. Marine Pollution Bulletin 49, 145–146. https://doi.org/10.1016/j.marpolbul.2004.04.013
1267. Rogowski, P.A., Terrill, E., Schiff, K., Kim, S.Y., 2015. An assessment of the transport of southern California stormwater ocean discharges. Marine Pollution Bulletin 90, 135–142. https://doi.org/10.1016/j.marpolbul.2014.11.004
1268. Romagnan, J.B., Aldamman, L., Gasparini, S., Nival, P., Aubert, A., Jamet, J.L., Stemmann, L., 2016. High frequency mesozooplankton monitoring: Can imaging systems and automated sample analysis help us describe and interpret changes in zooplankton community composition and size structure — An example from a coastal site. Journal of Marine Systems 162, 18–28. https://doi.org/10.1016/j.jmarsys.2016.03.013
1269. Rona, R.J., Burdett, H., Khondoker, M., Chesnokov, M., Green, K., Pernet, D., Jones, N., Greenberg, N., Wessely, S., Fear, N.T., 2017. Post-deployment screening for mental disorders and tailored advice about help-seeking in the UK military: a cluster randomised controlled trial. The Lancet 389, 1410–1423. https://doi.org/10.1016/S0140-6736(16)32398-4
1270. Ronen, S., Fontana, P., 2006. Wide- and multi-azimuth acquisition: Issues and answers. World Oil 227, 75–80.
1271. Roosevelt, C.H., 2015. Mapping site-level microtopography with Real- Time Kinematic Global Navigation Satellite Systems (RTK GNSS) and Unmanned Aerial Vehicle Photogrammetry (UAVP). Open Archaeology 1, 29–53. https://doi.org/10.2478/opar-2014-0003
1272. Rose, K.A., Adamack, A.T., Murphy, C.A., Sable, S.E., Kolesar, S.E., Craig, J.K., Breitburg, D.L., Thomas, P., Brouwer, M.H., Cerco, C.F., Diamond, S., 2009. Does hypoxia have population-level effects on coastal fish? Musings from the virtual world. Journal of Experimental Marine Biology and Ecology 381, S188–S203. https://doi.org/10.1016/j.jembe.2009.07.022
1273. Rose, V.J., Forney, W.M., Norton, R.A., Harrison, J.A., 2019. Catchment characteristics, water quality, and cyanobacterial blooms in Washington and Oregon Lakes. Lake and Reservoir Management. https://doi.org/10.1080/10402381.2018.1518940
1274. Rosén, P., Vogel, H., Cunningham, L., Hahn, A., Hausmann, S., Pienitz, R., Zolitschka, B., Wagner, B., Persson, P., 2011. Universally applicable model for the quantitative determination of lake sediment composition using fourier transform infrared spectroscopy. Environmental Science and Technology 45, 8858–8865. https://doi.org/10.1021/es200203z
1275. Rosser, N.J., Petley, D.N., Lim, M., Dunning, S.A., Allison, R.J., 2005. Terrestrial laser scanning for monitoring the process of hard rock coastal cliff erosion. Quarterly Journal of Engineering Geology and Hydrogeology 38, 363–375. https://doi.org/10.1144/1470-9236/05-008
1276. Rotherham, D., Gray, C.A., Johnson, D.D., Lokys, P., 2008. Effects of diel period and tow duration on estuarine fauna sampled with a beam trawl over bare sediment: Consequences for designing more reliable and efficient surveys. Estuarine, Coastal and Shelf Science 78, 179–189. https://doi.org/10.1016/j.ecss.2007.11.019
1277. Rotherham, D., Underwood, A.J., Chapman, M.G., Gray, C.A., 2007. A strategy for developing scientific sampling tools for fishery-independent surveys of estuarine fish in New South Wales, Australia. ICES Journal of Marine Science 64, 1512–1516. https://doi.org/10.1093/icesjms/fsm096
1278. Ruessink, B.G., Bell, P.S., Van Enckevort, I.M.J., Aarninkhof, S.G.J., 2002. Nearshore bar crest location quantified from time-averaged X-band radar images. Coastal Engineering 45, 19–32. https://doi.org/10.1016/S0378-3839(01)00042-4
1279. Ruhí, A., Batzer, D.P., 2014. Assessing Congruence and Surrogacy Among Wetland Macroinvertebrate Taxa Towards Efficiently Measuring Biodiversity. Wetlands 34, 1061–1071. https://doi.org/10.1007/s13157-014-0566-6
1280. Ruiz, J., Batty, A., Chavance, P., McElderry, H., Restrepo, V., Sharples, P., Santos, J., Urtizberea, A., 2014. Electronic monitoring trials on in the tropical tuna purse-seine fishery. ICES Journal of Marine Science 72, 1201–1213. https://doi.org/10.1093/icesjms/fsu224
1281. Rundberget, T., Gustad, E., Samdal, I.A., Sandvik, M., Miles, C.O., 2009. A convenient and cost-effective method for monitoring marine algal toxins with passive samplers. Toxicon 53, 543–550. https://doi.org/10.1016/j.toxicon.2009.01.010
1282. Ruse, L., 2010. Classification of nutrient impact on lakes using the chironomid pupal exuvial technique. Ecological Indicators 10, 594–601. https://doi.org/10.1016/j.ecolind.2009.10.002
1283. Ruse, L., 2011. Lake acidification assessed using chironomid pupal exuviae. Fundamental and Applied Limnology 178, 267–286. https://doi.org/10.1127/1863-9135/2011/0178-0267
1284. Russell, K.L., Baker, C.I., Hansen, C., Poland, G.A., Ryan, M.A.K., Merrill, M.M., Gray, G.C., 2015. Lack of effectiveness of the 23-valent polysaccharide pneumococcal vaccine in reducing all-cause pneumonias among healthy young military recruits: A randomized, double-blind, placebo-controlled trial. Vaccine 33, 1182–1187. https://doi.org/10.1016/j.vaccine.2014.12.058
1285. Ryan, J.C., Hubbard, A.L., Box, J.E., Todd, J., Christoffersen, P., Carr, J.R., Holt, T.O., Snooke, N., 2015. UAV photogrammetry and structure from motion to assess calving dynamics at Store Glacier, a large outlet draining the Greenland ice sheet. Cryosphere 9, 1–11. https://doi.org/10.5194/tc-9-1-2015
1286. Ryan, K.L., Trinnie, F.I., Jones, R., Hart, A.M., Wise, B.S., 2016. Recreational fisheries data requirements for monitoring catch shares. Fisheries Management and Ecology 23, 218–233. https://doi.org/10.1111/fme.12151
1287. Ryan, P.G., Moore, C.J., Van Franeker, J.A., Moloney, C.L., 2009. Monitoring the abundance of plastic debris in the marine environment. Philosophical Transactions of the Royal Society B: Biological Sciences 364, 1999–2012. https://doi.org/10.1098/rstb.2008.0207
1288. Ränäk, M., Saari, L., Hario, M., Hnninen, J., Lehikoinen, E., 2011. Breeding success and breeding population trends of waterfowl: Implications for monitoring. Wildlife Biology 17, 225–239. https://doi.org/10.2981/09-064
1289. Saannen, I., 2011. Opportunities for process automation to save energy. Wochenblatt fuer Papierfabrikation 139, 549–551.
1290. Sabol, B.M., Kannenberg, J., Skogerboe, J.G., 2009. Integrating acoustic mapping into operational aquatic plant management: A case study in Wisconsin. Journal of Aquatic Plant Management 47, 44–52.
1291. Saco-Álvarez, L., Durán, I., Ignacio Lorenzo, J., Beiras, R., 2010. Methodological basis for the optimization of a marine sea-urchin embryo test (SET) for the ecological assessment of coastal water quality. Ecotoxicology and Environmental Safety 73, 491–499. https://doi.org/10.1016/j.ecoenv.2010.01.018
1292. Sadeghi, S.H., Kazemi Kia, S., Erfanian, M., Movahed, S.M.S., 2019. Identifying representative watershed for the Urmia Lake Basin, Iran. Environmental Monitoring and Assessment 191. https://doi.org/10.1007/s10661-018-7147-8
1293. Saenger, A., Cécillon, L., Poulenard, J., Bureau, F., De Daniéli, S., Gonzalez, J.M., Brun, J.J., 2015. Surveying the carbon pools of mountain soils: A comparison of physical fractionation and Rock-Eval pyrolysis. Geoderma 241–242, 279–288. https://doi.org/10.1016/j.geoderma.2014.12.001
1294. Salonen, J.K., Taskinen, J., 2017. Electrofishing as a new method to search for unknown populations of the endangered freshwater pearl mussel Margaritifera margaritifera. Aquatic Conservation: Marine and Freshwater Ecosystems 27, 115–127. https://doi.org/10.1002/aqc.2667
1295. Sambuelli, L., Fiorucci, A., Dabove, P., Pascal, I., Colombero, C., Comina, C., 2017. Case history: A 5 km long waterborne geophysical survey along the Po river within the city of Turin (northwest Italy). Geophysics 82, B189–B199. https://doi.org/10.1190/geo2017-0071.1
1296. Sánchez-Fernández, D., Abellán, P., Mellado, A., Velasco, J., Millán, A., 2006. Are water beetles good indicators of biodiversity in Mediterranean aquatic ecosystems? The case of the Segura river basin (SE Spain). Biodiversity and Conservation 15, 4507–4520. https://doi.org/10.1007/s10531-005-5101-x
1297. Sánchez-Gendriz, I., Padovese, L.R., 2017. Temporal and spectral patterns of fish choruses in two protected areas in southern Atlantic. Ecological Informatics 38, 31–38. https://doi.org/10.1016/j.ecoinf.2017.01.003
1298. Sandin, M., Piikki, K., Jarvis, N., Larsbo, M., Bishop, K., Kreuger, J., 2018. Spatial and temporal patterns of pesticide concentrations in streamflow, drainage and runoff in a small Swedish agricultural catchment. Science of the Total Environment 610, 623–634. https://doi.org/10.1016/j.scitotenv.2017.08.068
1299. Sang, Z., Jiang, Y., Tsoi, Y.K., Leung, K.S.Y., 2014. Evaluating the environmental impact of artificial sweeteners: A study of their distributions, photodegradation and toxicities. Water Research 52, 260–274. https://doi.org/10.1016/j.watres.2013.11.002
1300. Santos, M.M., Jorge, P.A.S., Coimbra, J., Vale, C., Caetano, M., Bastos, L., Iglesias, I., Guimarães, L., Reis-Henriques, M.A., Teles, L.O., Vieira, M.N., Raimundo, J., Pinheiro, M., Nogueira, V., Pereira, R., Neuparth, T., Ribeiro, M.C., Silva, E., Castro, L.F.C., 2018. The last frontier: Coupling technological developments with scientific challenges to improve hazard assessment of deep-sea mining. Science of the Total Environment 627, 1505–1514. https://doi.org/10.1016/j.scitotenv.2018.01.221
1301. Santos, M.M., Solé, M., Lima, D., Hambach, B., Ferreira, A.M., Reis-Henriques, M.A., 2010. Validating a multi-biomarker approach with the shanny Lipophrys pholis to monitor oil spills in European marine ecosystems. Chemosphere 81, 685–691. https://doi.org/10.1016/j.chemosphere.2010.07.065
1302. Santos, R., Schröter-Schlaack, C., Antunes, P., Ring, I., Clemente, P., 2014. Reviewing the role of habitat banking and tradable development rights in the conservation policy mix. Environmental Conservation 42, 294–305. https://doi.org/10.1017/S0376892915000089
1303. Sasikala, K., Harikrishna, P., Thamarai Selvi, S., 2016. Cloud technology module for aggregation of survey data of housing and buildings in coastal villages as per IS:15499 towards cyclone disaster mitigation. Journal of Structural Engineering (India) 43, 199–211.
1304. Saunders, M.I., Atkinson, S., Klein, C.J., Weber, T., Possingham, H.P., 2017. Increased sediment loads cause non-linear decreases in seagrass suitable habitat extent. PLoS ONE 12. https://doi.org/10.1371/journal.pone.0187284
1305. Saunders, T., Xuereb, S., 2016. Optimising the monitoring of tropical aquatic resources through the development of Indigenous scientific capability. Reviews in Fish Biology and Fisheries 26, 727–736. https://doi.org/10.1007/s11160-016-9451-0
1306. Sayed, M.E., Nemitz, M.P., Aracri, S., McConnell, A.C., McKenzie, R.M., Stokes, A.A., 2018. The limpet: A ROS-enabled multi-sensing platform for the ORCA hub. Sensors (Switzerland) 18. https://doi.org/10.3390/s18103487
1307. Sayer, M.D.J., Küpper, F.C., van West, P., Wilson, C.M., Brown, H., Azzopardi, E., 2013. Managing scientific diving operations in a remote location: The Canadian high Arctic. Diving and Hyperbaric Medicine 43, 239–243.
1308. Scemama, P., Levrel, H., 2016. Using Habitat Equivalency Analysis to Assess the Cost Effectiveness of Restoration Outcomes in Four Institutional Contexts. Environmental Management 57, 109–122. https://doi.org/10.1007/s00267-015-0598-6
1309. Schaeffer, B.A., Bailey, S.W., Conmy, R.N., Galvin, M., Ignatius, A.R., Johnston, J.M., Keith, D.J., Lunetta, R.S., Parmar, R., Stumpf, R.P., Urquhart, E.A., Werdell, P.J., Wolfe, K., 2018. Mobile device application for monitoring cyanobacteria harmful algal blooms using Sentinel-3 satellite Ocean and Land Colour Instruments. Environmental Modelling and Software 109, 93–103. https://doi.org/10.1016/j.envsoft.2018.08.015
1310. Schintu, M., Buosi, C., Galgani, F., Marrucci, A., Marras, B., Ibba, A., Cherchi, A., 2015. Interpretation of coastal sediment quality based on trace metal and PAH analysis, benthic foraminifera, and toxicity tests (Sardinia, Western Mediterranean). Marine Pollution Bulletin 94, 72–83. https://doi.org/10.1016/j.marpolbul.2015.03.007
1311. Schmidt, D.J., Espinoza, T., Real, K., Dunlop, A., Kennard, M., Hughes, J.M., 2018. Improved genetic markers for monitoring recruitment dynamics in the endangered Mary River cod (Maccullochella mariensis). Journal of Applied Ichthyology 34, 633–637. https://doi.org/10.1111/jai.13633
1312. Schmieder, K., Lehmann, A., 2004. A spatio-temporal framework for efficient inventories of natural resources: A case study with submersed macrophytes. Journal of Vegetation Science 15, 807–816. https://doi.org/10.1111/j.1654-1103.2004.tb02324.x
1313. Schofield, O., Kohut, J., Glenn, S., Morell, J., Capella, J., Corredor, J., Orcutt, J., Arrott, M., Krueger, I., Meisinger, M., Peach, C., Vernon, F., Chave, A., Chao, Y., Chien, S., Thompson, D., Brown, W., Oliver, M., Boicourt, W., 2010. A regional slocum glider network in the Mid-Atlantic Bight leverages broad community engagement. Marine Technology Society Journal 44, 185–195. https://doi.org/10.4031/MTSJ.44.6.20
1314. Scholes, M.C., De Villiers, S., Scholes, R.J., Feig, G., 2007. Integrated approach to nutrient cycling monitoring. South African Journal of Science 103, 323–328.
1315. Schouten, P.W., Parisi, A.V., 2012. Underwater deployment of the polyphenylene oxide dosimeter combined with a neutral density filter to measure long-term solar UVB exposures. Journal of Photochemistry and Photobiology B: Biology 112, 31–36. https://doi.org/10.1016/j.jphotobiol.2012.04.004
1316. Schreinemachers, P., Balasubramaniam, S., Boopathi, N.M., Ha, C.V., Kenyon, L., Praneetvatakul, S., Sirijinda, A., Le, N.T., Srinivasan, R., Wu, M.H., 2015. Farmers’ perceptions and management of plant viruses in vegetables and legumes in tropical and subtropical Asia. Crop Protection 75, 115–123. https://doi.org/10.1016/j.cropro.2015.05.012
1317. Schröter, I., Paasche, H., Doktor, D., Xu, X., Dietrich, P., Wollschläger, U., 2017. Estimating soil moisture patterns with remote sensing and terrain data at the small catchment scale. Vadose Zone Journal 16. https://doi.org/10.2136/vzj2017.01.0012
1318. Schwörer, T., Federer, R.N., Ferren Ii, H.J., 2014. Invasive species management programs in Alaska: A survey of statewide expenditures, 2007 - 11. Arctic 67, 20–27. https://doi.org/10.14430/arctic4359
1319. Seak, S., Schmidt-Vogt, D., Thapa, G.B., 2011. A comparison between biodiversity monitoring systems to improve natural resource management in Tonle Sap Biosphere Reserve, Cambodia. International Journal of Biodiversity Science, Ecosystem Services and Management 7, 258–272. https://doi.org/10.1080/21513732.2011.649301
1320. Seekamp, E., McCreary, A., Mayer, J., Zack, S., Charlebois, P., Pasternak, L., 2016. Exploring the efficacy of an aquatic invasive species prevention campaign among water recreationists. Biological Invasions 18, 1745–1758. https://doi.org/10.1007/s10530-016-1117-2
1321. Sefrioui, S.B., Chergui, H., 2002. Application of kits microbiotests (Toxkits) in toxicity characterization of wastewater’s of the city of Fes (Morocco). Journal Europeen d’Hydrologie 33, 101–114.
1322. Segal, J.J., 2016. Perspective: Medical Malpractice Roller Coaster. World Neurosurgery 86, 61–62. https://doi.org/10.1016/j.wneu.2015.09.064
1323. Segurado, P., Almeida, C., Neves, R., Ferreira, M.T., Branco, P., 2018. Understanding multiple stressors in a Mediterranean basin: Combined effects of land use, water scarcity and nutrient enrichment. Science of the Total Environment 624, 1221–1233. https://doi.org/10.1016/j.scitotenv.2017.12.201
1324. Seiler, J., Friedman, A., Steinberg, D., Barrett, N., Williams, A., Holbrook, N.J., 2012. Image-based continental shelf habitat mapping using novel automated data extraction techniques. Continental Shelf Research 45, 87–97. https://doi.org/10.1016/j.csr.2012.06.003
1325. Sen, S., Haggard, B.E., Chaubey, I., Brye, K.R., Costello, T.A., Matlock, M.D., 2007. Sediment phosphorus release at Beaver Reservoir, northwest Arkansas, USA, 2002-2003: A preliminary investigation. Water, Air, and Soil Pollution 179, 67–77. https://doi.org/10.1007/s11270-006-9214-y
1326. Seoane, S., Garmendia, M., Revilla, M., Borja, Á., Franco, J., Orive, E., Valencia, V., 2011. Phytoplankton pigments and epifluorescence microscopy as tools for ecological status assessment in coastal and estuarine waters, within the Water Framework Directive. Marine Pollution Bulletin 62, 1484–1497. https://doi.org/10.1016/j.marpolbul.2011.04.010
1327. Serrana, J.M., Miyake, Y., Gamboa, M., Watanabe, K., 2019. Comparison of DNA metabarcoding and morphological identification for stream macroinvertebrate biodiversity assessment and monitoring. Ecological Indicators 101, 963–972. https://doi.org/10.1016/j.ecolind.2019.02.008
1328. Sevilla, E., Yuste, L., Rojo, F., 2015. Marine hydrocarbonoclastic bacteria as whole-cell biosensors for n-alkanes. Microbial Biotechnology 8, 693–706. https://doi.org/10.1111/1751-7915.12286
1329. Shabangu, F.W., Findlay, K.P., Yemane, D., Stafford, K.M., van den Berg, M., Blows, B., Andrew, R.K., 2019. Seasonal occurrence and diel calling behaviour of Antarctic blue whales and fin whales in relation to environmental conditions off the west coast of Check for South Africa. Journal of Marine Systems 190, 25–39. https://doi.org/10.1016/j.jmarsys.2018.11.002
1330. Shabangu, F.W., Findlay, K.P., Yemane, D., Stafford, K.M., van den Berg, M., Blows, B., Andrew, R.K., 2019. Seasonal occurrence and diel calling behaviour of Antarctic blue whales and fin whales in relation to environmental conditions off the west coast of South Africa. Journal of Marine Systems 190, 25–39. https://doi.org/10.1016/j.jmarsys.2018.11.002
1331. Shah, J., 2010. Hyperbaric oxygen therapy. Journal of the American College of Certified Wound Specialists 2, 9–13. https://doi.org/10.1016/j.jcws.2010.04.001
1332. Shahid, H., Singh, J.A., 2015. Investigational drugs for hyperuricemia. Expert Opinion on Investigational Drugs 24, 1013–1030. https://doi.org/10.1517/13543784.2015.1051617
1333. Shanmugam, P., Ahn, Y.H., 2007. New atmospheric correction technique to retrieve the ocean colour from SeaWiFS imagery in complex coastal waters. Journal of Optics A: Pure and Applied Optics 9. https://doi.org/10.1088/1464-4258/9/5/016
1334. Sharma, R., Kumar, D., 2018. Nanoadsorbents: An approach towards wastewater treatment, in: Nanotechnology for Sustainable Water Resources. pp. 371–405. https://doi.org/10.1002/9781119323655.ch12
1335. Shaw, J.L.A., Clarke, L.J., Wedderburn, S.D., Barnes, T.C., Weyrich, L.S., Cooper, A., 2016. Comparison of environmental DNA metabarcoding and conventional fish survey methods in a river system. Biological Conservation 197, 131–138. https://doi.org/10.1016/j.biocon.2016.03.010
1336. Sheehan, E.V., Stevens, T.F., Attrill, M.J., 2010. A quantitative, non-destructive methodology for habitat characterisation and benthic monitoring at offshore renewable energy developments. PLoS ONE 5. https://doi.org/10.1371/journal.pone.0014461
1337. Shen, L., Fischer, J., Martin, J., Hoque, M.E., Telgmann, L., Hintelmann, H., Metcalfe, C.D., Yargeau, V., 2016. Carbon Nanotube Integrative Sampler (CNIS) for passive sampling of nanosilver in the aquatic environment. Science of the Total Environment 569–570, 223–233. https://doi.org/10.1016/j.scitotenv.2016.06.095
1338. Sheng, H., Guo, H., Liu, H., Yang, Y., 2012. Reversion and analysis on cyanobacteria bloom in Waihai of Lake Dianchi. Shengtai Xuebao/ Acta Ecologica Sinica 32, 0056–0063. https://doi.org/10.5846/stxb201011261680
1339. Sheng, H., Li, N., Guo, H., Yang, Y., Liu, H., Zhou, F., He, C., Wang, C., 2010. Analysis of total amount allocation and emission trading potential in a watershed. Huanjing Kexue Xuebao/Acta Scientiae Circumstantiae 30, 655–663.
1340. Shephard, S., van Hal, R., de Boois, I., Birchenough, S.N.R., Foden, J., O’Connor, J., Geelhoed, S.C.V., Van Hoey, G., Marco-Rius, F., Reid, D.G., Schaber, M., 2015. Making progress towards integration of existing sampling activities to establish Joint Monitoring Programmes in support of the MSFD. Marine Policy 59, 105–111. https://doi.org/10.1016/j.marpol.2015.06.004
1341. Sherman, D., Constable, S.C., 2018. Permafrost Extent on the Alaskan Beaufort Shelf From Surface-Towed Controlled-Source Electromagnetic Surveys. Journal of Geophysical Research: Solid Earth 123, 7253–7265. https://doi.org/10.1029/2018JB015859
1342. Shi, W., Huang, J.C., 2018. Correcting on-site sampling bias: A new method with application to recreation demand analysis. Land Economics 94, 459–474. https://doi.org/10.3368/le.94.3.459
1343. Shin, H.J., 2011. Genetically engineered microbial biosensors for in situ monitoring of environmental pollution. Applied Microbiology and Biotechnology 89, 867–877. https://doi.org/10.1007/s00253-010-2990-8
1344. Shinohara, M., Uchida, K., Shimada, S., Tomioka, K., Suzuki, N., Minegishi, T., Kawahashi, S., Yoshikawa, Y., Ohashi, N., 2011. Novel concentration method for the detection of norovirus and sapovirus from water using minute particles of amorphous calcium phosphate. Journal of Medical Microbiology 60, 780–786. https://doi.org/10.1099/jmm.0.026260-0
1345. Shulman, A., David, I., Gelman, E., Priel, M., 2011. Control and management of brine disposal for inland desalination plants. Desalination and Water Treatment 31, 71–81. https://doi.org/10.5004/dwt.2011.2342
1346. Shuttleworth, E.L., Evans, M.G., Hutchinson, S.M., Rothwell, J.J., 2014. Assessment of Lead Contamination in Peatlands Using Field Portable XRF. Water Air and Soil Pollution 225. https://doi.org/10.1007/s11270-013-1844-2
1347. Sidike, P., Sagan, V., Maimaitijiang, M., Maimaitiyiming, M., Shakoor, N., Burken, J., Mockler, T., Fritschi, F.B., 2019. dPEN: deep Progressively Expanded Network for mapping heterogeneous agricultural landscape using WorldView-3 satellite imagery. Remote Sensing of Environment 221, 756–772. https://doi.org/10.1016/j.rse.2018.11.031
1348. Siegenthaler, A., Wangensteen, O.S., Soto, A.Z., Benvenuto, C., Corrigan, L., Mariani, S., 2019. Metabarcoding of shrimp stomach content: Harnessing a natural sampler for fish biodiversity monitoring. Molecular Ecology Resources 19, 206–220. https://doi.org/10.1111/1755-0998.12956
1349. Sigua, G.C., Tweedale, W.A., 2004. Assessing redesigned effectiveness of the water quality monitoring program in the Indian River Lagoon, Florida. Aquatic Conservation: Marine and Freshwater Ecosystems 14, 49–64. https://doi.org/10.1002/aqc.580
1350. Silva, D.R.O., Herlihy, A.T., Hughes, R.M., Callisto, M., 2017. An improved macroinvertebrate multimetric index for the assessment of wadeable streams in the neotropical savanna. Ecological Indicators 81, 514–525. https://doi.org/10.1016/j.ecolind.2017.06.017
1351. Silva, R., Veloso-Gomes, F., Pais-Barbosa, J., 2013. Morphological behaviour of costa da caparica beaches monitored during nourishment operations. Journal of Coastal Research 1862–1867. https://doi.org/10.2112/SI65-315
1352. Silva, S., Vieira-Lanero, R., Barca, S., Servia, M.J., Sánchez-Hernández, J., Cobo, F., 2014. Single pass electrofishing method for assessment and monitoring of larval lamprey populations. Limnetica 33, 217–226.
1353. Silvergieter, M.P., Lank, D.B., 2011. Patch scale nest-site selection by marbled murrelets (Brachyramphus marmoratus). Avian Conservation and Ecology 6. https://doi.org/10.5751/ACE-00483-060206
1354. Simoniello, C., Watson, S., Kirkpatrick, B., Spranger, M., Jochens, A.E., Kobara, S., Howard, M.K., 2015. One System, Many Societal Benefits: Building an Efficient, Cost-Effective Ocean Observing System for the Gulf of Mexico, in: Coastal Ocean Observing Systems. pp. 430–451. https://doi.org/10.1016/B978-0-12-802022-7.00023-7
1355. Simpson, J.M., Santo Domingo, J.W., Reasoner, D.J., 2002. Microbial source tracking: State of the science. Environmental Science and Technology 36, 5279–5288. https://doi.org/10.1021/es026000b
1356. Sinclair, M., 2002. Airborne laser surveys for resources development. Sea Technology 43, 51–56.
1357. Singh, P.K., Kumar, S., Singh, U.C., 2011. Groundwater resource evaluation in the Gwalior area, India, using satellite data: An integrated geomorphological and geophysical approach. Hydrogeology Journal 19, 1421–1429. https://doi.org/10.1007/s10040-011-0758-6
1358. Sioen, I., Leblanc, J.C., Volatier, J.L., Henauw, S.D., Camp, J.V., 2008. Evaluation of the exposure methodology for risk-benefit assessment of seafood consumption. Chemosphere 73, 1582–1588. https://doi.org/10.1016/j.chemosphere.2008.08.036
1359. Siontorou, C.G., Georgopoulos, K.N., Nalantzi, M.M.E., 2017. Designing biosensor networks for the environmental risk assessment of aquatic systems. Critical Reviews in Environmental Science and Technology 47, 40–63. https://doi.org/10.1080/10643389.2016.1278141
1360. Sips, R.J., Van Der Vlis, A., Nagel, R., Havers, B., 2013. A case for evidence-based levee management using sensor technology. IBM Journal of Research and Development 57. https://doi.org/10.1147/JRD.2013.2261213
1361. Skov, C., Hansen, J.H., Baktoft, H., Brodersen, J., Bronmark, C., Hansson, L.A., Hulthen, K., Chapman, B.B., Nilsson, P.A., 2019. Biomanipulating streams: a supplementary tool in lake restoration. Hydrobiologia 829, 205–216. https://doi.org/10.1007/s10750-018-3832-4
1362. Sleight, N., Neeson, T.M., 2018. Opportunities for collaboration between infrastructure agencies and conservation groups: Road-stream crossings in Oklahoma. Transportation Research Part D: Transport and Environment 63, 622–631. https://doi.org/10.1016/j.trd.2018.07.002
1363. Slimani, N., Sánchez-Fernández, D., Guilbert, E., Boumaïza, M., Guareschi, S., Thioulouse, J., 2019. Assessing potential surrogates of macroinvertebrate diversity in North-African Mediterranean aquatic ecosystems. Ecological Indicators 101, 324–329. https://doi.org/10.1016/j.ecolind.2019.01.017
1364. Smale, D.A., 2010. Monitoring marine macroalgae: The influence of spatial scale on the usefulness of biodiversity surrogates. Diversity and Distributions 16, 985–995. https://doi.org/10.1111/j.1472-4642.2010.00709.x
1365. Smale, D.A., Kendrick, G.A., Wernberg, T., 2010. Assemblage turnover and taxonomic sufficiency of subtidal macroalgae at multiple spatial scales. Journal of Experimental Marine Biology and Ecology 384, 76–86. https://doi.org/10.1016/j.jembe.2009.11.013
1366. Smale, D.A., Langlois, T.J., Kendrick, G.A., Meeuwig, J.J., Harvey, E.S., 2011. From fronds to fish: The use of indicators for ecological monitoring in marine benthic ecosystems, with case studies from temperate Western Australia. Reviews in Fish Biology and Fisheries 21, 311–337. https://doi.org/10.1007/s11160-010-9173-7
1367. Smallwood, C.B., Pollock, K.H., Wise, B.S., Hall, N.G., Gaughan, D.J., 2012. Expanding aerial-roving surveys to include counts of shore-based recreational fishers from remotely operated cameras: Benefits, limitations, and cost effectiveness. North American Journal of Fisheries Management 32, 1265–1276. https://doi.org/10.1080/02755947.2012.728181
1368. Smital, T., Terzic, S., Loncar, J., Senta, I., Zaja, R., Popovic, M., Mikac, I., Tollefsen, K.E., Thomas, K.V., Ahel, M., 2013. Prioritisation of organic contaminants in a river basin using chemical analyses and bioassays. Environmental Science and Pollution Research 20, 1384–1395. https://doi.org/10.1007/s11356-012-1059-x
1369. Smith, D., 2012. Determining location and design of cost-effective wildlife crossing structures along US-64 in North Carolina, Transportation Research Record. https://doi.org/10.3141/2270-05
1370. Smith, D.R., Villella, R.F., Lemarié, D.P., 2003. Application of adaptive cluster sampling to low-density populations of freshwater mussels. Environmental and Ecological Statistics 10, 7–15. https://doi.org/10.1023/A:1021956617984
1371. Smith, G.H., Owens, E.H., Reading, I., 2005. Water fraction measurement in marine fuel emulsions. Proceedings of the Institution of Mechanical Engineers Part M: Journal of Engineering for the Maritime Environment 219, 149–160. https://doi.org/10.1243/147509005X10549
1372. Smith, P.A., 2003. A cost-effective survey of fish occurring in a linear waterbody. Water and Environment Journal 17, 181–186. https://doi.org/10.1111/j.1747-6593.2003.tb00459.x
1373. Smith, R.N., Das, J., Heidarsson, H., Pereira, A.M., Arrichiello, F., Cetnić, I., Darjany, L., Garneau, M.E., Howard, M.D., Oberg, C., Ragan, M., Seubert, E., Smith, E.C., Stauffer, B.A., Schnetzer, A., Toro-Farmer, G., Caron, D.A., Jones, B.H., Sukhatme, G.S., 2010. USC CINAPS builds bridges: Observing and monitoring the Southern California bight. IEEE Robotics and Automation Magazine 17, 20–30. https://doi.org/10.1109/MRA.2010.935795
1374. Smith, S.D.A., 2005. Rapid assessment of invertebrate biodiversity on rocky shores: Where there’s a whelk there’s a way. Biodiversity and Conservation 14, 3565–3576. https://doi.org/10.1007/s10531-004-0828-3
1375. Smith, S.L., Cunniff, S.E., Peyronnin, N.S., Kritzer, J.P., 2017. Prioritizing coastal ecosystem stressors in the Northeast United States under increasing climate change. Environmental Science and Policy 78, 49–57. https://doi.org/10.1016/j.envsci.2017.09.009
1376. Smith, T.A., Osmond, D.L., Moorman, C.E., Stucky, J.M., Gilliam, J.W., 2008. Effect of vegetation management on bird habitat in Riparian buffer zones. Southeastern Naturalist 7, 277–288. https://doi.org/10.1656/1528-7092(2008)7[277:eovmob]2.0.co;2
1377. Smolarz, K., Bradtke, K., 2011. Bioindicative potential of shell abnormalities occurring in the clam Macoma balthica (L.) from the Baltic Sea. Marine Pollution Bulletin 62, 1421–1426. https://doi.org/10.1016/j.marpolbul.2011.04.031
1378. Snape, I., Riddle, M.J., Filler, D.M., Williams, P.J., 2003. Contaminants in freezing ground and associated ecosystems: Key issues at the beginning of the new millennium. Polar Record 39, 291–300.
1379. Snider, G., Weagle, C.L., Murdymootoo, K.K., Ring, A., Ritchie, Y., Stone, E., Walsh, A., Akoshile, C., Anh, N.X., Balasubramanian, R., Brook, J., Qonitan, F.D., Dong, J., Griffith, D., He, K., Holben, B.N., Kahn, R., Lagrosas, N., Lestari, P., Ma, Z., Misra, A., Norford, L.K., Quel, E.J., Salam, A., Schichtel, B., Segev, L., Tripathi, S., Wang, C., Yu, C., Zhang, Q., Zhang, Y., Brauer, M., Cohen, A., Gibson, M.D., Liu, Y., Martins, J.V., Rudich, Y., Martin, R.V., 2016. Variation in global chemical composition of PM 2.5 : emerging results from SPARTAN. Atmospheric Chemistry and Physics 16, 9629–9653. https://doi.org/10.5194/acp-16-9629-2016
1380. Soininen, J., 2015. Are catchment properties useful proxies for freshwater biodiversity?, in: Advances in Environmental Research. pp. 29–39.
1381. Sokolov, S., King, B.A., Rintoul, S.R., Rojas, R.L., 2004. Upper ocean temperature and the baroclinic transport stream function relationship in Drake Passage. Journal of Geophysical Research C: Oceans 109, C05001 1-14-C. https://doi.org/10.1029/2003JC002010
1382. Sokulski, G.S., 2006. Louisville doin’s. RT and S: Railway Track and Structures 102, 44.
1383. Solberg, A.H.S., 2012. Remote sensing of ocean oil-spill pollution. Proceedings of the IEEE 100, 2931–2945. https://doi.org/10.1109/JPROC.2012.2196250
1384. Soldal, E., Bekkby, T., Rinde, E., Bakkestuen, V., Erikstad, L., Longva, O., Isæus, M., 2009. Predictive Probability Modelling of Marine Habitats - A Case Study from the West Coast of Norway, in: Integrated Coastal Zone Management. pp. 57–65. https://doi.org/10.1002/9781444316285.ch5
1385. Solsten, B.L., Aitken, A.E., 2006. An application of GIS techniques to assess the risk of disturbance of archaeological sites by mass movement and marine flooding in Auyuittuq National Park Reserve, Nunavut. Geographie Physique et Quaternaire 60, 81–92. https://doi.org/10.7202/016366ar
1386. Soltani, A., Allan, A., Nguyen, H.A., Berry, S., 2019. Students’ commuting pattern from the viewpoint of environmentalism: comparing Australia with China. International Journal of Sustainability in Higher Education 20, 91–114. https://doi.org/10.1108/IJSHE-08-2018-0146
1387. Song, F., An, P.E., Folleco, A., 2003. Modeling and simulation of autonomous underwater vehicles: Design and implementation. IEEE Journal of Oceanic Engineering 28, 283–296. https://doi.org/10.1109/JOE.2003.811893
1388. Song, X., Li, H., Lin, X., Chen, X., Guo, X., Tian, J., 2009. Sea experiments of the underway conductivity-temperature-depth prototype made in China. Journal of Ocean University of China 8, 409–415. https://doi.org/10.1007/s11802-009-0409-x
1389. Soucémarianadin, L., Cécillon, L., Chenu, C., Baudin, F., Nicolas, M., Girardin, C., Barré, P., 2018. Is Rock-Eval 6 thermal analysis a good indicator of soil organic carbon lability? – A method-comparison study in forest soils. Soil Biology and Biochemistry 117, 108–116. https://doi.org/10.1016/j.soilbio.2017.10.025
1390. Southwell, C., Emmerson, L., 2015. Remotely-operating camera network expands Antarctic seabird observations of key breeding parameters for ecosystem monitoring and management. Journal for Nature Conservation 23, 1–8. https://doi.org/10.1016/j.jnc.2014.11.002
1391. Southwell, C., McKinlay, J., Low, M., Wilson, D., Newbery, K., Lieser, J.L., Emmerson, L., 2013. New methods and technologies for regional-scale abundance estimation of land-breeding marine animals: Application to Adélie penguin populations in East Antarctica. Polar Biology 36, 843–856. https://doi.org/10.1007/s00300-013-1310-z
1392. Souza, G.B.G., Barros, F., 2015. Analysis of sampling methods of estuarine benthic macrofaunal assemblages: sampling gear, mesh size, and taxonomic resolution. Hydrobiologia 743, 157–174. https://doi.org/10.1007/s10750-014-2033-z
1393. Spagnoli, G., Hannington, M., Bairlein, K., Hordt, A., Jegen, M., Petersen, S., Laurila, T., 2016. Electrical properties of seafloor massive sulfides. Geo-Marine Letters 36, 235–245. https://doi.org/10.1007/s00367-016-0439-5
1394. Spagnolo, A.M., Cristina, M.L., Casini, B., Perdelli, F., 2013. Legionella pneumophila in healthcare facilities. Reviews in Medical Microbiology 24, 70–80. https://doi.org/10.1097/MRM.0b013e328362fe66
1395. Spelling the end of the unexplained marine incident?, 2000. . Ship and Boat International 0, 35–39.
1396. Spencer, R.G.M., Baker, A., Ahad, J.M.E., Cowie, G.L., Ganeshram, R., Upstill-Goddard, R.C., Uher, G., 2007. Discriminatory classification of natural and anthropogenic waters in two U.K. estuaries. Science of the Total Environment 373, 305–323. https://doi.org/10.1016/j.scitotenv.2006.10.052
1397. Spratt, A., 2006. Meeting report: Emerging technologies for monitoring the coastal zone. Proceedings of the Institution of Civil Engineers: Maritime Engineering 159, 167–168. https://doi.org/10.1680/maen.2006.159.4.167
1398. Sreekanth, J., Datta, B., 2015. Review: Simulation-optimization models for the management and monitoring of coastal aquifers. Hydrogeology Journal 23, 1155–1166. https://doi.org/10.1007/s10040-015-1272-z
1399. Srinivasa Rao, C., Gopinath, K.A., Prasad, J.V.N.S., Prasannakumar, Singh, A.K., 2016. Climate Resilient Villages for Sustainable Food Security in Tropical India: Concept, Process, Technologies, Institutions, and Impacts, Advances in Agronomy. https://doi.org/10.1016/bs.agron.2016.06.003
1400. Srivastava, H., Singh, T.P., 2010. Assessment and development of algorithms to detection of oil spills using MODIS data. Journal of the Indian Society of Remote Sensing 38, 161–167. https://doi.org/10.1007/s12524-010-0007-9
1401. Stafford, K.J., McMeekan, C.M., 2000. A survey of the methods used by farmers to castrate calves in new zealand. New Zealand Veterinary Journal 48, 16–19. https://doi.org/10.1080/00480169.2000.36151
1402. Stahr, K.J., Knudsen, R.L., 2018. Evaluating the Efficacy of Using Time-Lapse Cameras to Assess Angling Use: An Example from a High-Use Metropolitan Reservoir in Arizona. North American Journal of Fisheries Management 38, 327–333. https://doi.org/10.1002/nafm.10026
1403. Stambaugh, K., Drummen, I., Cleary, C., Sheinberg, R., Kaminski, M., 2014. Structural fatigue life assessment and sustainment implications for a new class of US coast guard cutters. Transactions - Society of Naval Architects and Marine Engineers 122, 434–444.
1404. Stark, J.D., Phillips, N., 2009. Seasonal variability in the macroinvertebrate community index: Are seasonal correction factors required? New Zealand Journal of Marine and Freshwater Research 43, 867–882. https://doi.org/10.1080/00288330909510045
1405. Stark, K.D., Van Elswyk, M.E., Higgins, M.R., Weatherford, C.A., Salem, N., 2016. Global survey of the omega-3 fatty acids, docosahexaenoic acid and eicosapentaenoic acid in the blood stream of healthy adults. Progress in Lipid Research 63, 132–152. https://doi.org/10.1016/j.plipres.2016.05.001
1406. Stauffer, D.R., Seaman, N.L., Hunter, G.K., Leidner, S.M., Lario-Gibbs, A., Tanrikulu, S., 2000. A field-coherence technique for meteorological field-program design for air quality studies. Part I: Description and interpretation. Journal of Applied Meteorology 39, 297–316. https://doi.org/10.1175/1520-0450(2000)039<0297:AFCTFM>2.0.CO;2
1407. Steffe, A.S., Murphy, J.J., Reid, D.D., 2008. Supplemented access point sampling designs: A cost-effective way of improving the accuracy and precision of fishing effort and harvest estimates derived from recreational fishing surveys. North American Journal of Fisheries Management 28, 1001–1008. https://doi.org/10.1577/M06-248.1
1408. Steffy, L.Y., Shank, M.K., 2018. Considerations for using turbidity as a surrogate for suspended sediment in small, ungaged streams: Time-series selection, streamflow estimation, and regional transferability. River Research and Applications 34, 1304–1314. https://doi.org/10.1002/rra.3373
1409. Stein, B.R., Zheng, B., Kokkinidis, L., Kayastha, N., Seigler, T., Gökkaya, K., Gopalakrishnan, R., Hwang, W.H., 2012. An efficient remote sensing solution to update the NCWI. Photogrammetric Engineering and Remote Sensing 78, 537–547.
1410. Steinbeiser, C.M., Kioko, J., Maresi, A., Kaitilia, R., Kiffner, C., 2019. Relative abundance and activity patterns explain method-related differences in mammalian species richness estimates. Journal of Mammalogy 100, 192–201. https://doi.org/10.1093/jmammal/gyy175
1411. Stern, R.F., Picard, K.T., Hamilton, K.M., Walne, A., Tarran, G.A., Mills, D., McQuatters-Gollop, A., Edwards, M., 2015. Novel lineage patterns from an automated water sampler to probe marine microbial biodiversity with ships of opportunity. Progress in Oceanography 137, 409–420. https://doi.org/10.1016/j.pocean.2015.04.015
1412. Stewart, C., Renga, A., Gaffney, V., Schiavon, G., 2016. Sentinel-1 bathymetry for North Sea palaeolandscape analysis. International Journal of Remote Sensing 37, 471–491. https://doi.org/10.1080/01431161.2015.1129563
1413. Stevens, T., 2002. Rigor and representativeness in marine protected area design. Coastal Management 30, 237–248. https://doi.org/10.1080/08920750290042183
1414. Stevens, T.F., Sheehan, E.V., Gall, S.C., Fowell, S.C., Attrill, M.J., 2014. Monitoring benthic biodiversity restoration in Lyme Bay marine protected area: Design, sampling and analysis. Marine Policy 45, 310–317. https://doi.org/10.1016/j.marpol.2013.09.006
1415. Stoeck, T., Fruhe, L., Forster, D., Cordier, T., Martins, C.I.M., Pawlowski, J., 2018. Environmental DNA metabarcoding of benthic bacterial communities indicates the benthic footprint of salmon aquaculture. Marine Pollution Bulletin 127, 139–149. https://doi.org/10.1016/j.marpolbul.2017.11.065
1416. Stokes, D.J., Bulmer, R.H., Lundquist, C.J., 2016. Addressing the mismatch between restoration objectives and monitoring needs to support mangrove management. Ocean and Coastal Management 134, 69–78. https://doi.org/10.1016/j.ocecoaman.2016.09.024
1417. Stork, C., 2011. Seismic acquisition is moving from a “CMP Fold” perspective to a “Wavefield Recording” perspective which has significant implications on acquisition design, SEG Technical Program Expanded Abstracts. https://doi.org/10.1190/1.3627504
1418. Strand, J.A., Weisner, S.E.B., 2013. Effects of wetland construction on nitrogen transport and species richness in the agricultural landscape-Experiences from Sweden. Ecological Engineering 56, 14–25. https://doi.org/10.1016/j.ecoleng.2012.12.087
1419. Stratoudakis, Y., Mateus, C.S., Quintella, B.R., Antunes, C., Raposo de Almeida, P., 2016. Exploited anadromous fish in Portugal: Suggested direction for conservation and management. Marine Policy 73, 92–99. https://doi.org/10.1016/j.marpol.2016.07.031
1420. Strindberg, S., Coleman, R.A., Perez, V.R.B., Campbell, C.L., Majil, I., Gibson, J., 2016. In-water assessments of sea turtles at Glover’s Reef Atoll, Belize. Endangered Species Research 31, 211–225. https://doi.org/10.3354/esr00765
1421. Stringell, T.B., Millar, C.P., Sanderson, W.G., Westcott, S.M., McMath, M.J., 2014. When aerial surveys will not do: Grey seal pup production in cryptic habitats of Wales. Journal of the Marine Biological Association of the United Kingdom 94, 1155–1159. https://doi.org/10.1017/S0025315413000064
1422. Strobl, R.O., Robillard, P.D., 2008. Network design for water quality monitoring of surface freshwaters: A review. Journal of Environmental Management 87, 639–648. https://doi.org/10.1016/j.jenvman.2007.03.001
1423. Stuart, G., Hollingsworth, A., Thomsen, F., Szylkarski, S., Khan, S., Tomlinson, R., Kirkpatrick, S., Catterall, K., Capati, B., 2009. Gold coast seaway smartrelease decision support system: Optimising recycled water release in a sub tropical estuarine environment, Water Science and Technology. https://doi.org/10.2166/wst.2009.630
1424. Stuart-Smith, R.D., Edgar, G.J., Barrett, N.S., Bates, A.E., Baker, S.C., Bax, N.J., Becerro, M.A., Berkhout, J., Blanchard, J.L., Brock, D.J., Clark, G.F., Cooper, A.T., Davis, T.R., Day, P.B., Emmett Duffy, J., Holmes, T.H., Howe, S.A., Jordan, A., Kininmonth, S., Knott, N.A., Jonathan, L.S., Ling, S.D., Parr, A., Strain, E., Hugh, S., Russell, T., 2017. Assessing national biodiversity trends for rocky and coral reefs through the integration of citizen science and scientific monitoring programs. BioScience 67, 134–146. https://doi.org/10.1093/biosci/biw180
1425. Styan, C.A., Strzelecki, J., 2002. Small scale spatial distribution patterns and monitoring strategies for the introduced marine worm, Sabella spallanzanii (Polychaeta : Sabellidae). Transactions of the Royal Society of South Australia 126, 117–124.
1426. Styan, C.A., Strzelecki, J., 2002. Small scale spatial distribution patterns and monitoring strategies for the introduced marine worm, Sabella spallanzanii (Ploychaeta: Sabellidae). Transactions of the Royal Society of South Australia 126, 117–124.
1427. Su, H., Liu, H., Wang, L., Filippi, A.M., Heyman, W.D., Beck, R.A., 2014. Geographically adaptive inversion model for improving bathymetric retrieval from satellite multispectral imagery. IEEE Transactions on Geoscience and Remote Sensing 52, 465–476. https://doi.org/10.1109/TGRS.2013.2241772
1428. Su, H., Liu, H., Wu, Q., 2015. Prediction of Water Depth From Multispectral Satellite Imagery - The Regression Kriging Alternative. IEEE Geoscience and Remote Sensing Letters 12, 2511–2515. https://doi.org/10.1109/LGRS.2015.2489678
1429. Sun, C.H.J., Fine, L., 2016. A cost-effective discards-proportional at-sea monitoring allocation scheme for the groundfish fishery in New England. Marine Policy 66, 75–82. https://doi.org/10.1016/j.marpol.2015.12.029
1430. Sun, Y., Hodgart, S., Sweeting, M.N., 2000. Improved sea state monitoring using two-mode radar altimeter. Electronics Letters 36, 1813–1815. https://doi.org/10.1049/el:20001250
1431. Sundar, R., Venkatesan, R., Muthiah, M.A., Vedachalam, N., Atmanand, M.A., 2016. Performance assessment of indian meteorological ocean buoys with INSAT telemetry. Marine Technology Society Journal 50, 33–39. https://doi.org/10.4031/MTSJ.50.6.7
1432. Supply chain management, 2004. . Shipping World and Shipbuilder 205, 34.
1433. Sutherland, R.A., Tack, F.M.G., 2008. Extraction of labile metals from solid media by dilute hydrochloric acid. Environmental Monitoring and Assessment 138, 119–130. https://doi.org/10.1007/s10661-007-9748-5
1434. Swift, N., 2014. Pixel-perfect imaging. Sea Technology 55, 15–17.
1435. Swuste, P., Gulijk, C.V., Zwaard, W., 2010. Safety metaphors and theories, a review of the occupational safety literature of the US, UK and The Netherlands, till the first part of the 20th century. Safety Science 48, 1000–1018. https://doi.org/10.1016/j.ssci.2010.01.020
1436. Sykora-Bodie, S.T., Bezy, V., Johnston, D.W., Newton, E., Lohmann, K.J., 2017. Quantifying Nearshore Sea Turtle Densities: Applications of Unmanned Aerial Systems for Population Assessments. Scientific Reports 7. https://doi.org/10.1038/s41598-017-17719-x
1437. Szabó, Z., Gál, N.E., Kun, É., Szőcs, T., Falus, G., 2018. Accessing effects and signals of leakage from a CO2 reservoir to a shallow freshwater aquifer by reactive transport modelling. Environmental Earth Sciences 77. https://doi.org/10.1007/s12665-018-7637-6
1438. Szuster, B.W., Steckler, C., Kullavanijaya, B., 2008. Detecting and managing coastal fisheries and aquaculture gear using satellite radar imagery. Coastal Management 36, 318–329. https://doi.org/10.1080/08920750801968330
1439. Søndergaard, M., Jeppesen, E., 2007. Anthropogenic impacts on lake and stream ecosystems, and approaches to restoration. Journal of Applied Ecology 44, 1089–1094. https://doi.org/10.1111/j.1365-2664.2007.01426.x
1440. Sørensen, K.B., Thomsen, U.S., Juhler, S., Larsen, J., 2012. Cost efficient MIC management system based on molecular microbiological methods, NACE - International Corrosion Conference Series.
1441. Tagliapietra, D., Cornello, M., Ghirardini, A.V., 2005. Monitoring transitional waters using reduced benthic assemblages. Environment International 31, 1089–1093. https://doi.org/10.1016/j.envint.2005.05.019
1442. Takougang, I., Barbazan, P., Tchounwou, P.B., Noumi, E., 2008. The value of the freshwater snail dip scoop sampling method in macroinvertebrates bioassessment of sugar mill wastewater pollution in Mbandjock, Cameroon. International Journal of Environmental Research and Public Health 5, 68–75. https://doi.org/10.3390/ijerph5020068
1443. Tam, H.Y., Liu, S.Y., Ho, S.L., Ho, T.K., 2011. Fiber bragg grating sensors for railway systems, in: Fiber Bragg Grating Sensors: Recent Advancements, Industrial Applications and Market Exploitation. pp. 197–217. https://doi.org/10.2174/978160805084011101010197
1444. Tang, S., Chang, Y., Chia, G.H., Lee, H.K., 2015. Selective extraction and release using (EDTA-Ni)-layered double hydroxide coupled with catalytic oxidation of 3,3’,5,5’-tetramethylbenzidine for sensitive detection of copper ion. Analytica Chimica Acta 885, 106–113. https://doi.org/10.1016/j.aca.2015.05.029
1445. Tanos, P., Kovács, J., Kovács, S., Anda, A., Hatvani, I.G., 2015. Optimization of the monitoring network on the River Tisza (Central Europe, Hungary) using combined cluster and discriminant analysis, taking seasonality into account. Environmental Monitoring and Assessment 187. https://doi.org/10.1007/s10661-015-4777-y
1446. Taylor, S.M., Blight, S.J., Desfosses, C.J., Steffe, A.S., Ryan, K.L., Denham, A.M., Wise, B.S., 2018. Thermographic cameras reveal high levels of crepuscular and nocturnal shore-based recreational fishing effort in an Australian estuary. Ices Journal of Marine Science 75, 2107–2116. https://doi.org/10.1093/icesjms/fsy066
1447. Teatini, P., Tosi, L., Viezzoli, A., Baradello, L., Zecchin, M., Silvestri, S., 2011. Understanding the hydrogeology of the Venice Lagoon subsurface with airborne electromagnetics. Journal of Hydrology 411, 342–354. https://doi.org/10.1016/j.jhydrol.2011.10.017
1448. Teixeira, J.B., Martins, A.S., Pinheiro, H.T., Secchin, N.A., Leão de Moura, R., Bastos, A.C., 2013. Traditional Ecological Knowledge and the mapping of benthic marine habitats. Journal of Environmental Management 115, 241–250. https://doi.org/10.1016/j.jenvman.2012.11.020
1449. Telesca, L., Belluscio, A., Criscoli, A., Ardizzone, G., Apostolaki, E.T., Fraschetti, S., Gristina, M., Knittweis, L., Martin, C.S., Pergent, G., Alagna, A., Badalamenti, F., Garofalo, G., Gerakaris, V., Louise Pace, M., Pergent-Martini, C., Salomidi, M., 2015. Seagrass meadows (Posidonia oceanica) distribution and trajectories of change. Scientific Reports 5. https://doi.org/10.1038/srep12505
1450. Temmerman, S., Kirwan, M.L., 2015. Building land with a rising sea. Science 349, 588–589. https://doi.org/10.1126/science.aac8312
1451. Terán-Baamonde, J., Carlosena, A., Soto-Ferreiro, R.M., Andrade, J.M., Prada, D., 2017. Fast assessment of bioaccessible metallic contamination in marine sediments. Marine Pollution Bulletin 125, 310–317. https://doi.org/10.1016/j.marpolbul.2017.08.033
1452. Tercier-Waeber, M.L., Confalonieri, F., Riccardi, G., Sina, A., Nöel, S., Buffle, J., Graziottin, F., 2005. Multi Physical - Chemical profiler for real-time in situ monitoring of trace metal speciation and master variables: Development, validation and field applications. Marine Chemistry 97, 216–235. https://doi.org/10.1016/j.marchem.2005.03.004
1453. Tercier-Waeber, M.L., Taillefert, M., 2008. Remote in situ voltammetric techniques to characterize the biogeochemical cycling of trace metals in aquatic systems. Journal of Environmental Monitoring 10, 30–54. https://doi.org/10.1039/b714439n
1454. Tereszkiewicz, P.A., Ellis, J.T., Gould, H.A., 2019. Introducing a cost-effective method to assess beach-dune dynamics using existing infrastructure. Journal of Coastal Conservation. https://doi.org/10.1007/s11852-019-00686-y
1455. Terlizzi, A., Anderson, M.J., Bevilacqua, S., Ugland, K.I., 2014. Species-accumulation curves and taxonomic surrogates: An integrated approach for estimation of regional species richness. Diversity and Distributions 20, 356–368. https://doi.org/10.1111/ddi.12168
1456. The Italian job, 2004. . Journal of Offshore Technology 12, 28–29.
1457. Theary, C., Panagides, D., Laillou, A., Vonthanak, S., Kanarath, C., Chhorvann, C., Sambath, P., Sowath, S., Moench-Pfanner, R., 2013. Fish sauce, soy sauce, and vegetable oil fortification in Cambodia: where do we stand to date? Food and nutrition bulletin 34, S62-71.
1458. Thekisoe, O.M.M., Rambritch, N.E., Nakao, R., Bazie, R.S., Mbati, P., Namangala, B., Malele, I., Skilton, R.A., Jongejan, F., Sugimoto, C., Kawazu, S.I., Inoue, N., 2010. Loop-mediated isothermal amplification (LAMP) assays for detection of Theileria parva infections targeting the PIM and p150 genes. International Journal for Parasitology 40, 55–61. https://doi.org/10.1016/j.ijpara.2009.07.004
1459. Thiele, T., Gerber, L.R., 2017. Innovative financing for the High Seas. Aquatic Conservation: Marine and Freshwater Ecosystems 27, 89–99. https://doi.org/10.1002/aqc.2794
1460. Thies, P.R., Johanning, L., Harnois, V., Smith, H.C.M., Parish, D.N., 2014. Mooring line fatigue damage evaluation for floating marine energy converters: Field measurements and prediction. Renewable Energy 63, 133–144. https://doi.org/10.1016/j.renene.2013.08.050
1461. Thiruppathi, K., Lakshmi, P., Saravanan, N., Vinodha, S., Kirubagaran, R., 2014. On-line biofouling control in the plate heat exchanger system through osmotic shock. Indian Journal of Geo-Marine Sciences 43, 2158–2168.
1462. Thomas, F., Robinson, K., Judge, T., Eastlee, C., Frazer, E., Thomas, S.H., Romig, L., Blumen, I., Brozen, R., Williams, K., Swanson, E.R., Hartsell, S., Johnson, J., Hutton, K., Heffernan, J., North, M., Johnson, K., Petersen, P., Toews, R., Zalar, C.M., 2004. The 2003 Air Medical Leadership Congress: Findings and recommendations. Air Medical Journal 23, 20–36. https://doi.org/10.1016/j.amj.2004.03.004
1463. Thomas, K.E., Hall, R.I., Scrimgeour, G.J., 2013. Evaluating the use of algal pigments to assess the biological condition of streams. Environmental Monitoring and Assessment 185, 7895–7913. https://doi.org/10.1007/s10661-013-3143-1
1464. Thomas, P.A., 2000. Radionuclides in the terrestrial ecosystem near a Canadian uranium mill - Part II: Small mammal food chains and bioavailability. Health Physics 78, 625–632. https://doi.org/10.1097/00004032-200006000-00004
1465. Thomas, S.M., Tjaden, N.B., Frank, C., Jaeschke, A., Zipfel, L., Wagner-Wiening, C., Faber, M., Beierkuhnlein, C., Stark, K., 2018. Areas with high hazard potential for autochthonous transmission of Aedes albopictus-associated arboviruses in Germany. International Journal of Environmental Research and Public Health 15. https://doi.org/10.3390/ijerph15061270
1466. Thomassin, A., White, C.S., Stead, S.S., David, G., 2010. Social acceptability of a marine protected area: The case of Reunion Island. Ocean and Coastal Management 53, 169–179. https://doi.org/10.1016/j.ocecoaman.2010.01.008
1467. Thompson, B.W., Riddle, M.J., Stark, J.S., 2003. Cost-efficient methods for marine pollution monitoring at Casey Station, East Antarctica: The choice of sieve mesh-size and taxonomic resolution. Marine Pollution Bulletin 46, 232–243. https://doi.org/10.1016/S0025-326X(02)00366-1
1468. Thomsen, P.F., Willerslev, E., 2015. Environmental DNA - An emerging tool in conservation for monitoring past and present biodiversity. Biological Conservation 183, 4–18. https://doi.org/10.1016/j.biocon.2014.11.019
1469. Thorngren, L., Holthuis, T.D., Lindegarth, S., Lindegarth, M., 2017. Developing methods for assessing abundance and distribution of European oysters (Ostrea edulis) using towed video. Plos One 12. https://doi.org/10.1371/journal.pone.0187870
1470. Thorson, J.T., Shelton, A.O., Ward, E.J., Skaug, H.J., 2015. Geostatistical delta-generalized linear mixed models improve precision for estimated abundance indices for West Coast groundfishes. ICES Journal of Marine Science 72, 1297–1310. https://doi.org/10.1093/icesjms/fsu243
1471. Thresher, R.E., Jones, M., Drake, D.A.R., 2019. Stakeholder attitudes towards the use of recombinant technology to manage the impact of an invasive species: Sea Lamprey in the North American Great Lakes. Biological Invasions 21, 575–586. https://doi.org/10.1007/s10530-018-1848-3
1472. Tian, S.Q., Han, C., Chen, Y., Chen, X.J., 2013. Evaluating the impact of spatio-temporal scale on CPUE standardization. Chinese Journal of Oceanology and Limnology 31, 935–948. https://doi.org/10.1007/s00343-013-2285-x
1473. Tinsley, D., 2006. Measuring performance. Shipping World and Shipbuilder 207, 10–15.
1474. Toma, D.M., Masmitja, I., del Río, J., Martinez, E., Artero-Delgado, C., Casale, A., Figoli, A., Pinzani, D., Cervantes, P., Ruiz, P., Memè, S., Delory, E., 2018. Smart embedded passive acoustic devices for real-time hydroacoustic surveys. Measurement: Journal of the International Measurement Confederation 125, 592–605. https://doi.org/10.1016/j.measurement.2018.05.030
1475. Torbick, N., Becker, B., 2009. Evaluating principal components analysis for identifying Optimal bands using wetland hyperspectral measurements from the Great Lakes, USA. Remote Sensing 1, 408–417. https://doi.org/10.3390/rs1030408
1476. Torn, K., Martin, G., Suursaar, U., 2016. Beach wrack macrovegetation index for assessing coastal phytobenthic biodiversity. Proceedings of the Estonian Academy of Sciences 65, 78–87. https://doi.org/10.3176/proc.2016.1.08
1477. Tosi, L., Teatini, P., Bincoletto, L., Simonini, P., Strozzi, T., 2012. Integrating Geotechnical and Interferometric SAR Measurements for Secondary Compressibility Characterization of Coastal Soils. Surveys in Geophysics 33, 907–926. https://doi.org/10.1007/s10712-012-9186-y
1478. Traganos, D., Aggarwal, B., Poursanidis, D., Topouzelis, K., Chrysoulakis, N., Reinartz, P., 2018. Towards global-scale seagrass mapping and monitoring using Sentinel-2 on Google Earth Engine: The case study of the Aegean and Ionian Seas. Remote Sensing 10. https://doi.org/10.3390/rs10081227
1479. Traganos, D., Reinartz, P., 2018. Mapping Mediterranean seagrasses with Sentinel-2 imagery. Marine Pollution Bulletin 134, 197–209. https://doi.org/10.1016/j.marpolbul.2017.06.075
1480. Trasviña-Moreno, C.A., Blasco, R., Marco, Á., Casas, R., Trasviña-Castro, A., 2017. Unmanned aerial vehicle based wireless sensor network for marine-coastal environment monitoring. Sensors (Switzerland) 17. https://doi.org/10.3390/s17030460
1481. Traversetti, L., Del Grosso, F., Malafoglia, V., Colasanti, M., Ceschin, S., Larsen, S., Scalici, M., 2017. The Hydra regeneration assay reveals ecological risks in running waters: a new proposal to detect environmental teratogenic threats. Ecotoxicology 26, 184–195. https://doi.org/10.1007/s10646-016-1753-4
1482. Treasure, A.M., Roquet, F., Ansorge, I.J., Bester, M.N., Boehme, L., Bornemann, H., Charrassin, J.B., Chevallier, D., Costa, D.P., Fedak, M.A., Guinet, C., Hammill, M.O., Harcourt, R.G., Hindell, M.A., Kovacs, K.M., Lea, M.A., Lovell, P., Lowther, A.D., Lydersen, C., McIntyre, T., McMahon, C.R., Muelbert, M.M.C., Nicholls, K., Picard, B., Reverdin, G., Trites, A.W., Williams, G.D., de Bruyn, P.J.N., 2017. Marine Mammals Exploring the Oceans Pole to Pole A Review of the MEOP Consortium. Oceanography 30, 132–138. https://doi.org/10.5670/oceanog.2017.234
1483. Triantis, T., Tsimeli, K., Kaloudis, T., Thanassoulias, N., Lytras, E., Hiskia, A., 2010. Development of an integrated laboratory system for the monitoring of cyanotoxins in surface and drinking waters. Toxicon 55, 979–989. https://doi.org/10.1016/j.toxicon.2009.07.012
1484. Triyanti, A., Chu, E., 2018. A survey of governance approaches to ecosystem-based disaster risk reduction: Current gaps and future directions. International Journal of Disaster Risk Reduction 32, 11–21. https://doi.org/10.1016/j.ijdrr.2017.11.005
1485. Trois, G.M., Borjesson, L., 2005. Development of an immunoassay for the determination of polyaromatic hydrocarbons in plasma samples from oiled seabirds. Environmental Science and Technology 39, 3748–3755. https://doi.org/10.1021/es048935t
1486. Tsai, W.P., Huang, S.P., Cheng, S.T., Shao, K.T., Chang, F.J., 2017. A data-mining framework for exploring the multi-relation between fish species and water quality through self-organizing map. Science of the Total Environment 579, 474–483. https://doi.org/10.1016/j.scitotenv.2016.11.071
1487. Tsarpali, V., Kamilari, M., Dailianis, S., 2012. Seasonal alterations of landfill leachate composition and toxic potency in semi-arid regions. Journal of Hazardous Materials 233–234, 163–171. https://doi.org/10.1016/j.jhazmat.2012.07.007
1488. Tse, A.C.K., Lau, K.Y.T., Ge, W., Wu, R.S.S., 2013. A rapid screening test for endocrine disrupting chemicals using primary cell culture of the marine medaka. Aquatic Toxicology 144–145, 50–58. https://doi.org/10.1016/j.aquatox.2013.09.022
1489. Tsingas, C., Brizard, T., Muhaidib, A.A., 2018. Seafloor seismic acquisition using autonomous underwater vehicles. Geophysical Prospecting. https://doi.org/10.1111/1365-2478.12670
1490. Tucker, A.J., Williamson, C.E., Oris, J.T., 2012. Development and application of a UV attainment threshold for the prevention of warmwater aquatic invasive species. Biological Invasions 14, 2331–2342. https://doi.org/10.1007/s10530-012-0232-y
1491. Tugiyono, L., Gagnon, M.M., 2001. Testing the toxicity of influents to activated sludge plants with the Vibrio fischeri bioassay utilising a sludge matrix. Environmental Toxicology 16, 422–427. https://doi.org/10.1002/tox.10000
1492. Turemis, M., Silletti, S., Pezzotti, G., Sanchís, J., Farré, M., Giardi, M.T., 2018. Optical biosensor based on the microalga-paramecium symbiosis for improved marine monitoring. Sensors and Actuators, B: Chemical 270, 424–432. https://doi.org/10.1016/j.snb.2018.04.111
1493. Turner, C.R., Miller, D.J., Coyne, K.J., Corush, J., 2014. Improved methods for capture, extraction, and quantitative assay of environmental DNA from Asian bigheaded carp (hypophthalmichthys spp.). PLoS ONE 9. https://doi.org/10.1371/journal.pone.0114329
1494. Turner, I.L., Harley, M.D., Drummond, C.D., 2016. UAVs for coastal surveying. Coastal Engineering 114, 19–24. https://doi.org/10.1016/j.coastaleng.2016.03.011
1495. Turner, K., Wong, W.H., Gerstenberger, S.L., Miller, J.M., 2011. Interagency monitoring action plan (I-MAP) for quagga mussels in lake mead, Nevada-Arizona, USA. Aquatic Invasions 6, 195–204. https://doi.org/10.3391/ai.2011.6.2.08
1496. Turner, R.E., McClenachan, G., 2018. Reversing wetland death from 35,000 cuts: Opportunities to restore Louisiana’s dredged canals. PLoS ONE 13. https://doi.org/10.1371/journal.pone.0207717
1497. Tweddale, S.A., Frank, T.D., 2006. A procedure to extrapolate vegetation cover estimates over large arid and semi-arid regions using multiple spatial resolution imagery. Geocarto International 21, 13–18. https://doi.org/10.1080/10106040608542369
1498. Tweedley, J.R., Warwick, R.M., Clarke, K.R., Potter, I.C., 2014. Family-level AMBI is valid for use in the north-eastern Atlantic but not for assessing the health of microtidal Australian estuaries. Estuarine, Coastal and Shelf Science 141, 85–96. https://doi.org/10.1016/j.ecss.2014.03.002
1499. Twiss, M.R., M. Stryszowska, K., 2016. State of emerging technologies for assessing aquatic condition in the Great Lakes - St. Lawrence River system. Journal of Great Lakes Research 42, 1470–1477. https://doi.org/10.1016/j.jglr.2016.10.002
1500. Tyne, J.A., Loneragan, N.R., Johnston, D.W., Pollock, K.H., Williams, R., Bejder, L., 2016. Evaluating monitoring methods for cetaceans. Biological Conservation 201, 252–260. https://doi.org/10.1016/j.biocon.2016.07.024
1501. Töpfer, K., 2003. Balancing competing water uses - A necessity for sustainable development, Water Science and Technology.
1502. Törökné, A.K., László, E., Chorus, I., Sivonen, K., Barbosa, F.A.R., 2000. Cyanobacterial toxins detected by Thamnotoxkit (A double blind experiment). Environmental Toxicology 15, 549–553. https://doi.org/10.1002/1522-7278(2000)15:5<549::AID-TOX27>3.0.CO;2-Z
1503. Ubrihien, R.P., Taylor, A.M., Maher, W.A., 2017. Bioaccumulation, oxidative stress and cellular damage in the intertidal gastropod Bembicium nanum exposed to a metal contamination gradient. Marine and Freshwater Research 68, 922–930. https://doi.org/10.1071/MF16026
1504. Udell, B.J., Martin, J., Fletcher, R.J., Jr., Bonneau, M., Edwards, H.H., Gowan, T.A., Hardy, S.K., Gurarie, E., Calleson, C.S., Deutsch, C.J., 2018. Integrating encounter theory with decision analysis to evaluate collision risk and determine optimal protection zones for wildlife. Journal of Applied Ecology. https://doi.org/10.1111/1365-2664.13290
1505. Udy, J., Gall, M., Longstaff, B., Moore, K., Roelfsema, C., Spooner, D.R., Albert, S., 2005. Water quality monitoring: A combined approach to investigate gradients of change in the Great Barrier Reef, Australia. Marine Pollution Bulletin 51, 224–238. https://doi.org/10.1016/j.marpolbul.2004.10.048
1506. Ugalde, S.C., Preston, J., Ogier, E., Crawford, C., 2018. Analysis of farm management strategies following herpesvirus (OsHV-1) disease outbreaks in Pacific oysters in Tasmania, Australia. Aquaculture 495, 179–186. https://doi.org/10.1016/j.aquaculture.2018.05.019
1507. Ulrich, K.U., Bethge, C., Guderitz, I., Heinrich, B., Neumann, V., Nitsche, C., Benthaus, F.C., 2012. In-Lake Neutralization: Quantification and Prognoses of the Acid Load into a Conditioned Pit Lake (Lake Bockwitz, Central Germany). Mine Water and the Environment 31, 320–338. https://doi.org/10.1007/s10230-012-0206-4
1508. Unsworth, R.K.F., Peters, J.R., McCloskey, R.M., Hinder, S.L., 2014. Optimising stereo baited underwater video for sampling fish and invertebrates in temperate coastal habitats. Estuarine, Coastal and Shelf Science 150, 281–287. https://doi.org/10.1016/j.ecss.2014.03.020
1509. Urbanič, G., 2014. A Littoral Fauna Index for assessing the impact of lakeshore alterations in Alpine lakes. Ecohydrology 7, 703–716. https://doi.org/10.1002/eco.1392
1510. Vacchi, M., Rovere, A., Schiaffino, C.F., Ferrari, M., 2012. Monitoring the effectiveness of re-establishing beaches artificially: Methodological and practical insights into the use of video transects and SCUBA-operated coring devices. Underwater Technology 30, 201–206. https://doi.org/10.3723/ut.30.201
1511. Waddington, A., 2013. A layered approach gathering data once, for multiple purposes. Hydro International 17, 20–24.
1512. Waddington, K.I., Piek, B.W., Payne, A.D., Grove, S.L., Harvey, E.S., Kendrick, G.A., Taylor, H.F., Meeuwig, J.J., 2010. Description of a remote still photography system for collection of benthic photo-quadrats. Marine Technology Society Journal 44, 56–63. https://doi.org/10.4031/MTSJ.44.2.1
1513. Vaidya, U.K., Abraham, A., Bhide, S., 2001. Affordable processing of thick section and integral multi-functional composites. Composites - Part A: Applied Science and Manufacturing 32, 1133–1142. https://doi.org/10.1016/S1359-835X(01)00033-1
1514. Wainger, L.A., 2012. Opportunities for reducing Total Maximum Daily Load (TMDL) compliance costs: Lessons from the Chesapeake Bay. Environmental Science and Technology 46, 9256–9265. https://doi.org/10.1021/es300540k
1515. Wakamiya, S.M., Roy, C.L., 2009. Use of monitoring data and population viability analysis to inform reintroduction decisions: Peregrine falcons in the Midwestern United States. Biological Conservation 142, 1767–1776. https://doi.org/10.1016/j.biocon.2009.03.015
1516. Wakefield, C.B., Santana-Garcon, J., Dorman, S.R., Blight, S., Denham, A., Wakeford, J., Molony, B.W., Newman, S.J., 2017. Performance of bycatch reduction devices varies for chondrichthyan, reptile, and cetacean mitigation in demersal fish trawls: Assimilating subsurface interactions and unaccounted mortality. ICES Journal of Marine Science 74, 343–358. https://doi.org/10.1093/icesjms/fsw143
1517. Valdor, P.F., Gómez, A.G., Ondiviela, B., Puente, A., Juanes, J.A., 2016. Prioritization maps: The integration of environmental risks to manage water quality in harbor areas. Marine Pollution Bulletin 111, 57–67. https://doi.org/10.1016/j.marpolbul.2016.07.028
1518. Walker, J.D., Dimitrova, N., Dimitrov, S., Mekenyan, O., Plewak, D., 2004a. Use of QSARs to promote more cost-effective use of chemical monitoring resources. 2. Screening chemicals for hydrolysis half-lives, Henry’s Law constants, ultimate biodegradation potential, modes of toxic action and bioavailability. Water Quality Research Journal of Canada 39, 40–49.
1519. Walker, J.D., Knaebel, D., Mayo, K., Tunkel, J., Gray, D.A., 2004b. Use of QSARs to promote more cost-effective use of chemical monitoring resources. 1. Screening industrial chemicals and pesticides, direct food additives, indirect food additives and pharmaceuticals for biodegradation, bioconcentration and aquatic toxicity potential. Water Quality Research Journal of Canada 39, 35–39.
1520. Walker, W.E., 2000. POLSSS: Overview and cost-effectiveness analysis. Safety Science 35, 105–121. https://doi.org/10.1016/S0925-7535(00)00026-6
1521. Wallace, B., Purcell, M., 2003. The benefits of nitrogen and total organic carbon (TOC) determination by high-temperature combustion. American Laboratory 35, 58–64.
1522. Wallis, R.J., Ali, N., Barnes, P., Khan, F., Whitfield, O., 2002. Redevelopment of the Brighton Marine Field, Trinidad ... the early days. Petroleum Geoscience 8, 327–337. https://doi.org/10.1144/petgeo.8.4.327
1523. Valta-Hulkkonen, K., Kanninen, A., Ilvonen, R., Leka, J., 2005. Assessment of aerial photography as a method for monitoring aquatic vegetation in lakes of varying trophic status. Boreal Environment Research 10, 57–66.
1524. Van De Weyer, K., Nienhaus, I., Tigges, P., Hussner, A., Hamann, U., 2007. A simple and cost-efficient method for the planar collection of submerse plant existence in lakes. Wasser und Abfall 9, 20–22.
1525. Van der Velden, R., 2006. Low cost or cost effective? Managing cost through the fit for purpose principle. Hydro International 10, 21–23.
1526. Van Lancker, V., Baeye, M., 2015. Wave glider monitoring of sediment transport and dredge plumes in a shallow marine sandbank environment. PLoS ONE 10. https://doi.org/10.1371/journal.pone.0128948
1527. van Opstal, H., Mallie, C., 2006. Low-budget hydrography. Hydro International 10, 50–51.
1528. van Overmeeren, R., Craeymeersch, J., van Dalfsen, J., Fey, F., van Heteren, S., Meesters, E., 2009. Acoustic habitat and shellfish mapping and monitoring in shallow coastal water - Sidescan sonar experiences in The Netherlands. Estuarine, Coastal and Shelf Science 85, 437–448. https://doi.org/10.1016/j.ecss.2009.07.016
1529. van Proosdij, D., Perrott, B., Carrol, K., 2013. Development and Application of a Geo-temporal Atlas for Climate Change Adaptation in Bay of Fundy Dykelands. Journal of Coastal Research 1069–1074. https://doi.org/10.2112/si65-181.1
1530. Van Rein, H., Schoeman, D.S., Brown, C.J., Quinn, R., Breen, J., 2012. Development of low-cost image mosaics of hard-bottom sessile communities using SCUBA: Comparisons of optical media and of proxy measures of community structure. Journal of the Marine Biological Association of the United Kingdom 92, 49–62. https://doi.org/10.1017/S0025315411000233
1531. van Rein, H., Schoeman, D.S., Brown, C.J., Quinn, R., Breen, J., 2011. Development of benthic monitoring methods using photoquadrats and scuba on heterogeneous hard-substrata: A boulder-slope community case study. Aquatic Conservation: Marine and Freshwater Ecosystems 21, 676–689. https://doi.org/10.1002/aqc.1224
1532. Van Slobbe, E., Klimkowska, A., Van Dobben, H., Wiersma, A., 2013. The soft sand engine at the Workumer Buitenwaarden. Landschap 30, 219–227.
1533. Van Urk, W., De Vries, W.A., 2000. POLSSS: Policy making for sea shipping safety. Safety Science 35, 139–150. https://doi.org/10.1016/S0925-7535(00)00028-X
1534. Vandenbrouck, T., Jones, O.A.H., Dom, N., Griffin, J.L., De Coen, W., 2010. Mixtures of similarly acting compounds in Daphnia magna: From gene to metabolite and beyond. Environment International 36, 254–268. https://doi.org/10.1016/j.envint.2009.12.006
1535. Wang, F., Chen, J., Gao, S., Tang, K., Meng, X., 2017. Development and sea trial of real-time offshore pipeline installation monitoring system. Ocean Engineering 146, 468–476. https://doi.org/10.1016/j.oceaneng.2017.09.016
1536. Wang, H., Liu, Z., Kim, S., Koo, C., Cho, Y., Jang, D.Y., Kim, Y.J., Han, A., 2014. Microfluidic acoustophoretic force based low-concentration oil separation and detection from the environment. Lab on a Chip 14, 947–956. https://doi.org/10.1039/c3lc51032h
1537. Wang, Q., Li, Y., Wang, Y., 2011. Optimizing the weight loss-on-ignition methodology to quantify organic and carbonate carbon of sediments from diverse sources. Environmental Monitoring and Assessment 174, 241–257. https://doi.org/10.1007/s10661-010-1454-z
1538. Wang, T., Liu, S., Qian, X., Shimizu, T., Dente, S.M.R., Hashimoto, S., Nakajima, J., 2017. Assessment of the municipal water cycle in China. Science of the Total Environment 607–608, 761–770. https://doi.org/10.1016/j.scitotenv.2017.07.072
1539. Wang, W., Wang, B.D., Xu, Z.J., Zhang, Y.H., Wang, Z.L., 2016. Modeling stem and branch biomass of Tamarix spp. in the marine protected area in Changyi, China. Shengtai Xuebao/ Acta Ecologica Sinica 36, 2202–2209. https://doi.org/10.5846/stxb201410272098
1540. VanGerwen-Toyne, M., Gillis, D.M., Tallman, R.F., 2014. Statistical power: an important consideration in designing community-based monitoring programs for Arctic and sub-Arctic subsistence fisheries. Polar Biology 37, 1435–1444. https://doi.org/10.1007/s00300-014-1533-7
1541. Varble, S., Secchi, S., 2013. Human consumption as an invasive species management strategy. A preliminary assessment of the marketing potential of invasive Asian carp in the US. Appetite 65, 58–67. https://doi.org/10.1016/j.appet.2013.01.022
1542. Varkitzi, I., Francé, J., Basset, A., Cozzoli, F., Stanca, E., Zervoudaki, S., Giannakourou, A., Assimakopoulou, G., Venetsanopoulou, A., Mozetič, P., Tinta, T., Skejic, S., Vidjak, O., Cadiou, J.F., Pagou, K., 2018. Pelagic habitats in the Mediterranean Sea: A review of Good Environmental Status (GES) determination for plankton components and identification of gaps and priority needs to improve coherence for the MSFD implementation. Ecological Indicators 95, 203–218. https://doi.org/10.1016/j.ecolind.2018.07.036
1543. Warner, W., Nödler, K., Farinelli, A., Blum, J., Licha, T., 2018. Integrated approach for innovative monitoring strategies of reservoirs and lakes. Environmental Engineering and Management Journal 17, 2497–2505.
[truncated: 36,659 more chars]
