# Supplementary material for: Cost-efficiency assessments of marine monitoring methods lack rigor—a systematic mapping of literature and an end-user view on optimal cost-efficiency analysis
Source: Environ Monit Assess. 2021 Jun 9;193(7):400. doi: 10.1007/s10661-021-09159-y (PMC8187199; doi:10.1007/s10661-021-09159-y)
Supplement: Supplementary file 2 — Supplementary file2 (DOCX 55 KB) [file 10661_2021_9159_MOESM2_ESM.docx]

Annex 2. A reference list of the 313 articles that included a cost-efficiency assessment method and thus, contributed to the knowledge base

1. Aarnio, K.; Mattila, J.; Bonsdorff, E. 2011. Comparison of different sampling strategies in monitoring zoobenthos and classification of archipelago areas. Boreal Environment Research. 16(5): 395-406.
2. Abdelzaher, A. M.; Solo-Gabriele, H. M.; Palmer, C. J.; Scott, T. M. 2009. Simultaneous concentration of enterococci and coliphage from marine waters using a dual layer filtration system. Journal of Environmental Quality. 38(6): 2468-2473.
3. Abdullah, A. L.; Anscelly, A. A.; Mohamed, J.; Yasin, Z. 2016. Conservation of pulau payar marine park and optical remote sensing models. Kemanusiaan. 23: 79-107.
4. Aguado-Giménez, F.; Marín, A.; Montoya, S.; Marín-Guirao, L.; Piedecausa, A.; García-García, B. 2007. Comparison between some procedures for monitoring offshore cage culture in western Mediterranean Sea: Sampling methods and impact indicators in soft substrata. Aquaculture. 271(43469): 357-370.
5. Allan, M. G.; Hamilton, D. P.; Hicks, B.; Brabyn, L. 2015. Empirical and semi-analytical chlorophyll a algorithms for multi-temporal monitoring of New Zealand lakes using Landsat. Environmental Monitoring and Assessment. 187(6).
6. Alquezar, R.; Boyd, W. 2007. Development of rapid, cost effective coral survey techniques: Tools for management and conservation planning. Journal of Coastal Conservation. 11(2): 105-119.
7. Amin, R.; Richards, B. L.; Misa, Wfxe; Taylor, J. C.; Miller, D. R.; Rollo, A. K.; Demarke, C.; Singh, H.; Young, G. C.; Childress, J.; Ossolinski, J. E.; Reardon, R. T.; Koyanagi, K. H. 2017. The Modular Optical Underwater Survey System. Sensors. 17(10).
8. Anderson, J. W.; Hartwell, S. I.; Hameed, M. J. 2005. Regional comparisons of coastal sediment contamination detected by a biomarker (P450 HRGS; EPA Method 4425). Environmental Science and Technology. 39(1): 17-23.
9. Andrade, H.; Renaud, P. E. 2011. Polychaete/amphipod ratio as an indicator of environmental impact related to offshore oil and gas production along the Norwegian continental shelf. Marine Pollution Bulletin. 62(12): 2836-2844.
10. Ashraf, S.; Brabyn, L.; Hicks, B. J.; Collier, K. 2010. Satellite remote sensing for mapping vegetation in New Zealand freshwater environments: A review. New Zealand Geographer. 66(1): 33-43.
11. Assilzadeh, H.; Levy, J. K.; Wang, X.; Gao, Y.; Zhong, Z. 2010. Geosensing systems engineering for ocean security and sustainable coastal zone management. Journal of Systems Science and Systems Engineering. 19(1): 22-35.
12. Assoumani, A.; Coquery, M.; Liger, L.; Mazzella, N.; Margoum, C. 2015. Field application of passive SBSE for the monitoring of pesticides in surface waters. Environmental Science and Pollution Research. 22(6): 3997-4008.
13. Atkinson, J.; Esteves, L. S.; Williams, J. W.; McCann, D. L.; Bell, P. S. 2018. The Application of X-Band Radar for Characterization of Nearshore Dynamics on a Mixed Sand and Gravel Beach. Journal of Coastal Research. 85: 281-285.
14. Au, D. W. T. 2004. The application of histo-cytopathological biomarkers in marine pollution monitoring: A review. Marine Pollution Bulletin. 48(43718): 817-834.
15. Aubert, A.; Antajan, E.; Lynam, C.; Pitois, S.; Pliru, A.; Vaz, S.; Thibault, D. 2018. No more reason for ignoring gelatinous zooplankton in ecosystem assessment and marine management: Concrete cost-effective methodology during routine fishery trawl surveys. Marine Policy. 89: 100-108.
16. Aykanat, T.; Lindqvist, M.; Pritchard, V. L.; Primmer, C. R. 2016. From population genomics to conservation and management: a workflow for targeted analysis of markers identified using genome-wide approaches in Atlantic salmon Salmo salar. Journal of Fish Biology. 89(6): 2658-2679.
17. Aylagas, E.; Borja, Á; Irigoien, X.; Rodríguez-Ezpeleta, N. 2016. Benchmarking DNA metabarcoding for biodiversity-based monitoring and assessment. Frontiers in Marine Science. 3(JUN).
18. Aylesworth, L.; Phoonsawat, R.; Suvanachai, P.; Vincent, A. C. J. 2017. Generating spatial data for marine conservation and management. Biodiversity and Conservation. 26(2): 383-399.
19. Bailey, H.; Clay, G.; Coates, E. A.; Lusseau, D.; Senior, B.; Thompson, P. M. 2010. Using T-PODs to assess variations in the occurrence of coastal bottlenose dolphins and harbour porpoises. Aquatic Conservation: Marine and Freshwater Ecosystems. 20(2): 150-158.
20. Baldantoni, D.; Maisto, G.; Bartoli, G.; Alfani, A. 2005. Analyses of three native aquatic plant species to assess spatial gradients of lake trace element contamination. Aquatic Botany. 83(1): 48-60.
21. Balfour, C. A. 2012. Cost-effective remote data acquisition and instrumentation management for oceanographic and environmental monitoring applications. Journal of Operational Oceanography. 5(2): 41-52.
22. Balfour, C. A.; Howarth, M. J.; Jones, D. S.; Doyle, T. 2013. The design and development of an irish sea passenger-ferry-based oceanographic measurement system. Journal of Atmospheric and Oceanic Technology. 30(6): 1226-1239.
23. Bartholomew, D. C.; Mangel, J. C.; Alfaro-Shigueto, J.; Pingo, S.; Jimenez, A.; Godley, B. J. 2018. Remote electronic monitoring as a potential alternative to on-board observers in small-scale fisheries. Biological Conservation. 219: 35-45.
24. Bastian, T.; Haberlin, D.; Purcell, J. E.; Hays, G. C.; Davenport, J.; McAllen, R.; Doyle, T. K. 2011. Large-scale sampling reveals the spatio-temporal distributions of the jellyfish Aurelia aurita and Cyanea capillata in the Irish Sea. Marine Biology. 158(12): 2639-2652.
25. Begliomini, F. N.; Maciel, D. C.; de Almeida, S. M.; Abessa, D. M.; Maranho, L. A.; Pereira, C. D. S.; Yogui, G. T.; Zanardi-Lamardo, E.; Castro, Í B. 2017. Shell alterations in limpets as putative biomarkers for multi-impacted coastal areas. Environmental Pollution. 226: 494-503.
26. Beisiegel, K.; Darr, A.; Gogina, M.; Zettler, M. L. 2017. Benefits and shortcomings of non-destructive benthic imagery for monitoring hard-bottom habitats. Marine Pollution Bulletin. 121(43467): 43600.
27. Bell, J. J.; Burton, M.; Bullimore, B.; Newman, P. B.; Lock, K. 2006. Morphological monitoring of subtidal sponge assemblages. Marine Ecology Progress Series. 311: 79-91.
28. Bellanger, M.; Levrel, H. 2017. A cost-effectiveness analysis of alternative survey methods used for the monitoring of marine recreational fishing in France. Ocean and Coastal Management. 138: 19-28.
29. Bellchambers, L. M.; Evans, S. N.; Meeuwig, J. J. 2013. Assessing the effectiveness of two methods of habitat characterisation for understanding species habitat relationships, using the western rock lobster (Panulirus cygnus George). Fisheries Research. 139: 43595.
30. Bennett, K.; Wilson, S. K.; Shedrawi, G.; McLean, D. L.; Langlois, T. J. 2016. Can diver operated stereo-video surveys for fish be used to collect meaningful data on benthic coral reef communities?. Limnology and Oceanography: Methods. 14(12): 874-885.
31. Berman, J.; Burton, M.; Gibbs, R.; Lock, K.; Newman, P.; Jones, J.; Bell, J. 2013. Testing the suitability of a morphological monitoring approach for identifying temporal variability in a temperate sponge assemblage. Journal for Nature Conservation. 21(3): 173-182.
32. Bevilacqua, S.; Mistri, M.; Terlizzi, A.; Munari, C. 2018. Assessing the effectiveness of surrogates for species over time: Evidence from decadal monitoring of a Mediterranean transitional water ecosystem. Marine Pollution Bulletin. 131: 507-514.
33. Bian, X.; Shao, Y.; Wang, S.; Tian, W.; Wang, X.; Zhang, C. 2018. Shallow Water Depth Retrieval from Multitemporal Sentinel-1 SAR Data. IEEE Journal of Selected Topics in Applied Earth Observations and Remote Sensing. 11(9): 2991-3000.
34. Boldt, J. L.; Williams, K.; Rooper, C. N.; Towler, R. H.; Gauthier, S. 2018. Development of stereo camera methodologies to improve pelagic fish biomass estimates and inform ecosystem management in marine waters. Fisheries Research. 198: 66-77.
35. Boman, E. M.; De Graaf, M.; Nagelkerke, L. A. J.; Van Rijn, J.; Schlochtern, M. M. Z.; Smaal, A. 2016. UNDERWATER TOWED VIDEO: A NOVEL METHOD TO ESTIMATE DENSITIES OF QUEEN CONCH (LOBATUS GIGAS; STROMBIDAE) ACROSS ITS DEPTH RANGE. Journal of Shellfish Research. 35(2): 493-498.
36. Bonino, G.; Burlando, M.; De Gaetano, P.; Solari, G.; Carmisciano, C.; Iafolla, L. 2015. Sea state monitoring and simulation in the “wind, ports, and sea project. Towards Green Marine Technology and Transport. 875-882.
37. Booth, C. G. 2016. Challenge of using passive acoustic monitoring in high-energy environments: UK tidal environments and other case studies. Advances in Experimental Medicine and Biology. 875: 101-108.
38. Borker, A. L.; Halbert, P.; McKown, M. W.; Tershy, B. R.; Croll, D. A. 2015. A comparison of automated and traditional monitoring techniques for marbled murrelets using passive acoustic sensors. Wildlife Society Bulletin. 39(4): 813-818.
39. Bosch, N. E.; Goncalves, J. M. S.; Erzini, K.; Tuya, F. 2017. How and what matters: Sampling method affects biodiversity estimates of reef fishes. Ecology and Evolution. 7(13): 4891-4906.
40. Bourlat, S. J.; Borja, A.; Gilbert, J.; Taylor, M. I.; Davies, N.; Weisberg, S. B.; Griffith, J. F.; Lettieri, T.; Field, D.; Benzie, J.; Glöckner, F. O.; Rodríguez-Ezpeleta, N.; Faith, D. P.; Bean, T. P.; Obst, M. 2013. Genomics in marine monitoring: New opportunities for assessing marine health status. Marine Pollution Bulletin. 74(1): 19-31.
41. Bramburger, A. J.; Stephen Brown, R.; Haley, J.; Ridal, J. J. 2015. A new, automated rapid fluorometric method for the detection of Escherichia coli in recreational waters. Journal of Great Lakes Research. 41(1): 298-302.
42. Braulik, G. T.; Kasuga, M.; Wittich, A.; Kiszka, J. J.; MacCaulay, J.; Gillespie, D.; Gordon, J.; Said, S. S.; Hammond, P. S. 2018. Cetacean rapid assessment: An approach to fill knowledge gaps and target conservation across large data deficient areas. Aquatic Conservation: Marine and Freshwater Ecosystems. 28(1): 216-230.
43. Bresciani, M.; Stroppiana, D.; Odermatt, D.; Morabito, G.; Giardino, C. 2011. Assessing remotely sensed chlorophyll-a for the implementation of the Water Framework Directive in European perialpine lakes. Science of the Total Environment. 409(17): 3083-3091.
44. Brischoux, F.; Bonnet, X.; Legagneux, P. 2009. Are sea snakes pertinent bio-indicators for coral reefs? a comparison between species and sites. Marine Biology. 156(10): 1985-1992.
45. Brodin, Y.; Ejdung, G.; Strandberg, J.; Lyrholm, T. 2013. Improving environmental and biodiversity monitoring in the Baltic Sea using DNA barcoding of Chironomidae (Diptera). Molecular Ecology Resources. 13(6): 996-1004.
46. Bui, M. P. N.; Brockgreitens, J.; Ahmed, S.; Abbas, A. 2016. Dual detection of nitrate and mercury in water using disposable electrochemical sensors. Biosensors and Bioelectronics. 85: 280-286.
47. Buzzelli, C. P.; Ramus, J.; Paerl, H. W. 2003. Ferry-based monitoring of surface water quality in North Carolina estuaries. Estuaries. 26(4 A): 975-984.
48. Byer, J. D.; Struger, J.; Sverko, E.; Klawunn, P.; Todd, A. 2011. Spatial and seasonal variations in atrazine and metolachlor surface water concentrations in Ontario (Canada) using ELISA. Chemosphere. 82(8): 1155-1160.
49. Cahalane, C.; Hanafin, J.; Monteys, X. 2017. Improving satellite-derived bathymetry. Hydro International. 21(1): 16-19.
50. Camino-Sánchez, F. J.; Zafra-Gómez, A.; Oliver-Rodríguez, B.; Ruiz-Naranjo, I.; Ruiz-García, J.; Vílchez, J. L. 2012. Validation of a method for the determination of tributyltin in seawater by stir bar sorptive extraction-liquid chromatography tandem mass spectrometry. Journal of Chromatography A. 1263: 14-20.
51. Canedo-Arguelles, M.; Boix, D.; Sanchez-Millaruelo, N.; Sala, J.; Caiola, N.; Nebra, A.; Rieradevall, M. 2012. A rapid bioassessment tool for the evaluation of the water quality of transitional waters. Estuarine Coastal and Shelf Science. 111: 129-138.
52. Carpio, J. N.; Nalunat, J. C.; Bañares, J. C. T.; Fernando, A. R.; Jurado, J. A. O.; Bayang, S. J. N.; Dizon, C. F.; Valerio, C. M. G.; Marasigan, J. S. 2018. A cost-effective fish pond monitoring and warning system using thermal probe. International Journal of Simulation: Systems, Science and Technology. 19(3): 14.1-14.4.
53. Castellote, M.; Brotons, J. M.; Chicote, C.; Gazo, M.; Cerdà, M. 2015. Long-term acoustic monitoring of bottlenose dolphins, Tursiops truncatus, in marine protected areas in the Spanish Mediterranean Sea. Ocean and Coastal Management. 113: 54-66.
54. Chai, F.; Wang, C. A.; Wang, T. T.; Li, L.; Su, Z. M. 2010. Colorimetric Detection of Pb2+ Using Glutathione Functionalized Gold Nanoparticles. Acs Applied Materials & Interfaces. 2(5): 1466-1470.
55. Chambault, P.; Vandeperre, F.; Machete, M.; Lagoa, J. C.; Pham, C. K. 2018. Distribution and composition of floating macro litter off the Azores archipelago and Madeira (NE Atlantic) using opportunistic surveys. Marine Environmental Research. 141: 225-232.
56. Cheng, C.; Chen, H. Y.; Wu, C. S.; Meena, J. S.; Simon, T.; Ko, F. H. 2016. A highly sensitive and selective cyanide detection using a gold nanoparticle-based dual fluorescence-colorimetric sensor with a wide concentration range. Sensors and Actuators, B: Chemical. 227: 283-290.
57. Chial, B.; Persoone, G. 2002. Cyst-based toxicity tests XIII - Development of a short chronic sediment toxicity test with the ostracod crustacean Heterocypris incongruents: Methodology and precision. Environmental Toxicology. 17(6): 528-532.
58. Claire, K.; Nathalie, C. M.; Noelle, B.; Frank, D. 2019. Optimizing cost-efficiency of long term monitoring programs by using for spatially balanced sampling designs: The case of manila clams in Arcachon bay. Ecological Informatics. 49: 32-39.
59. Clayton, L.; Dennison, G. 2017. Inexpensive video drop-camera for surveying sensitive benthic habitats: Applications from glass sponge (Hexactinellida) reefs in Howe Sound, British Columbia. Canadian Field-Naturalist. 131(1): 46-54.
60. Clemento, A. J.; Crandall, E. D.; Garza, J. C. 2014. Evaluation of a single nucleotide polymorphism baseline for genetic stock identification of Chinook Salmon (Oncorhynchus tshawytscha) in the California Current large marine ecosystem. Fishery Bulletin. 112(43499): 112-130.
61. Codiga, D. L. 2015. A Marine Autonomous Surface Craft for Long-Duration, Spatially Explicit, Multidisciplinary Water Column Sampling in Coastal and Estuarine Systems. Journal of Atmospheric and Oceanic Technology. 32(3): 627-641.
62. Colefax, A. P.; Butcher, P. A.; Kelaher, B. P. 2018. The potential for unmanned aerial vehicles (UAVs) to conduct marine fauna surveys in place of manned aircraft. ICES Journal of Marine Science. 75(1): 43473.
63. Collins, S. F.; Diana, M. J.; Butler, S. E.; Wahl, D. H. 2017. A Comparison of Sampling Gears for Capturing Juvenile Silver Carp in River-Floodplain Ecosystems. North American Journal of Fisheries Management. 37(1): 94-100.
64. Cooke, S. J.; Schreer, J. F. 2002. Determination of fish community composition in the untempered regions of a thermal effluent canal-the efficacy of a fixed underwater videography system. Environmental Monitoring and Assessment. 73(2): 109-129.
65. Costa, B. M.; Battista, T. A.; Pittman, S. J. 2009. Comparative evaluation of airborne LiDAR and ship-based multibeam SoNAR bathymetry and intensity for mapping coral reef ecosystems. Remote Sensing of Environment. 113(5): 1082-1100.
66. Cragg, J. L.; Burger, A. E.; Piatt, J. F. 2015. Testing the effectiveness of automated acoustic sensors for monitoring vocal activity of marbled murrelets Brachyramphus marmoratus. Marine Ornithology. 43(2): 151-160.
67. Currie, J. J.; Stack, S. H.; Kaufman, G. D. 2018. Conservation and education through ecotourism: Using citizen science to monitor cetaceans in the four-island region of Maui, Hawaii. Tourism in Marine Environments. 13(43499): 65-71.
68. Danovaro, R.; Carugati, L.; Berzano, M.; Cahill, A. E.; Carvalho, S.; Chenuil, A.; Corinaldesi, C.; Cristina, S.; David, R.; Dell'Anno, A.; Dzhembekova, N.; Garcés, E.; Gasol, J. M.; Goela, P.; Féral, J. P.; Ferrera, I.; Forster, R. M.; Kurekin, A. A.; Rastelli, E.; Marinova, V.; Miller, P. I.; Moncheva, S.; Newton, A.; Pearman, J. K.; Pitois, S. G.; Reñé, A.; Rodríguez-Ezpeleta, N.; Saggiomo, V.; Simis, S. G. H.; Stefanova, K.; Wilson, C.; Martire, M. L.; Greco, S.; Cochrane, S. K. J.; Mangoni, O.; Borja, A. 2016. Implementing and innovating marine monitoring approaches for assessing marine environmental status. Frontiers in Marine Science. 3(NOV).
69. Davidson, M. A.; Aarninkhof, S. G. J.; Van Koningsveld, M.; Holman, R. A. 2006. Developing coastal video monitoring systems in support of coastal zone management. Journal of Coastal Research. : 49-56.
70. Davies, C. H.; Ajani, P.; Armbrecht, L.; Atkins, N.; Baird, M. E.; Beard, J.; Bonham, P.; Burford, M.; Clementson, L.; Coad, P.; Crawford, C.; Dela-Cruz, J.; Doblin, M. A.; Edgar, S.; Eriksen, R.; Everett, J. D.; Furnas, M.; Harrison, D. P.; Hassler, C.; Henschke, N.; Hoenner, X.; Ingleton, T.; Jameson, I.; Keesing, J.; Leterme, S. C.; James McLaughlin, M.; Miller, M.; Moffatt, D.; Moss, A.; Nayar, S.; Patten, N. L.; Patten, R.; Pausina, S. A.; Proctor, R.; Raes, E.; Robb, M.; Rothlisberg, P.; Saeck, E. A.; Scanes, P.; Suthers, I. M.; Swadling, K. M.; Talbot, S.; Thompson, P.; Thomson, P. G.; Uribe-Palomino, J.; Van Ruth, P.; Waite, A. M.; Wright, S.; Richardson, A. J. 2018. A database of chlorophyll a in Australian waters. Scientific Data. 5.
71. Delparte, D. M.; Belt, M.; Nishioka, C.; Turner, N.; Richardson, R. T.; Ericksen, T. 2014. Monitoring tropical alpine lake levels in a culturally sensitive environment utilizing 3D technological approaches. Arctic, Antarctic, and Alpine Research. 46(4): 709-718.
72. Descamp, P.; Holon, F.; Ballesta, L.; Guilbert, A.; Guillot, M.; Boissery, P.; Raimondino, V.; Deter, J. 2011. Fast and easy method for seagrass monitoring: Application of acoustic telemetry to precision mapping of Posidonia oceanica beds. Marine Pollution Bulletin. 62(2): 284-292.
73. Deus, D.; Gloaguen, R. 2013. Remote Sensing Analysis of Lake Dynamics in Semi-Arid Regions: Implication for Water Resource Management. Lake Manyara, East African Rift, Northern Tanzania. Water. 5(2): 698-727.
74. Diana, C. M.; Jonas, J. L.; Claramunt, R. M.; Fitzsimons, J. D.; Marsden, J. E. 2006. A comparison of methods for sampling round goby in rocky littoral areas. North American Journal of Fisheries Management. 26(3): 514-522.
75. Ding, Y.; Garcia, C. D.; Rogers, K. R. 2008. Poly(dimethylsiloxane) microchip electrophoresis with contactless conductivity detection for measurement of chemical warfare agent degradation products. Analytical Letters. 41(2): 335-350.
76. Ding, Y.; Rogers, K. 2008. Measurement of nitrogen mustard degradation products by poly(dimethylsiloxane) microchip electrophoresis with contactless conductivity detection. Electroanalysis. 20(20): 2192-2198.
77. Dinsdale, E. A.; Harriott, V. J. 2004. Assessing Anchor Damage on Coral Reefs: A Case Study in Selection of Environmental Indicators. Environmental Management. 33(1): 126-139.
78. Dissanayake, A.; Galloway, T. S. 2004. Evaluation of fixed wavelength fluorescence and synchronous fluorescence spectrophotometry as a biomonitoring tool of environmental contamination. Marine Environmental Research. 58(43501): 281-285.
79. Donaldson, K. A.; Griffin, D. W.; Paul, J. H. 2002. Detection, quantitation and identification of enteroviruses from surface waters and sponge tissue from the Florida Keys using real-time RT-PCR. Water Research. 36(10): 2505-2514.
80. Dong, W.; Wang, F.; Fang, M.; Wu, J.; Wang, S.; Li, M.; Yang, J.; Chernick, M.; Hinton, D. E.; Pei, D. S.; Chen, H.; Zheng, N.; Mu, J.; Xie, L.; Dong, W. 2019. Use of biological detection methods to assess dioxin-like compounds in sediments of Bohai Bay, China. Ecotoxicology and Environmental Safety. 173: 339-346.
81. Embling, C. B.; Walters, A. E. M.; Dolman, S. J. 2015. How much effort is enough? The power of citizen science to monitor trends in coastal cetacean species. Global Ecology and Conservation. 3: 867-877.
82. Emelogu, E. S.; Pollard, P.; Robinson, C. D.; Smedes, F.; Webster, L.; Oliver, I. W.; McKenzie, C.; Seiler, T. B.; Hollert, H.; Moffat, C. F. 2013. Investigating the significance of dissolved organic contaminants in aquatic environments: Coupling passive sampling with in vitro bioassays. Chemosphere. 90(2): 210-219.
83. Evans, L. J.; Jones, T. H.; Pang, K.; Saimin, S.; Goossens, B. 2016. Spatial ecology of estuarine crocodile (Crocodylus porosus)nesting in a fragmented landscape. Sensors (Switzerland). 16(9).
84. Evans, S. N.; Abdo, D. A. 2010. A cost-effective technique for measuring relative water movement for studies of benthic organisms. Marine and Freshwater Research. 61(11): 1327-1335.
85. Ewing, G.; Frusher, S. 2015. New puerulus collector design suitable for fishery-dependent settlement monitoring. ICES Journal of Marine Science. 72: i225-i231.
86. Fairclough, D. V.; Brown, J. I.; Carlish, B. J.; Crisafulli, B. M.; Keay, I. S. 2014. Breathing life into fisheries stock assessments with citizen science. Scientific Reports. 4.
87. Fairclough, D. V.; Edmonds, J. S.; Lenanton, R. C. J.; Jackson, G.; Keay, I. S.; Crisafulli, B. M.; Newman, S. J. 2011. Rapid and cost-effective assessment of connectivity among assemblages of Choerodon rubescens (Labridae), using laser ablation ICP-MS of sagittal otoliths. Journal of Experimental Marine Biology and Ecology. 403(43467): 46-53.
88. Farré, M.; Brix, R.; Barceló, D. 2005. Screening water for pollutants using biological techniques under European Union funding during the last 10 years. TrAC - Trends in Analytical Chemistry. 24(6): 532-545.
89. Farrell, E. D.; Clarke, M. W.; Mariani, S. 2009. A simple genetic identification method for Northeast Atlantic smoothhound sharks (Mustelus spp.). ICES Journal of Marine Science. 66(3): 561-565.
90. Fernandes, M.; Benger, S.; Sharma, S. K.; Gaylard, S.; Kildea, T.; Hoare, S.; Braley, M.; Irving, A. D. 2012. The use of ?15N signatures of translocated macroalgae to map coastal nutrient plumes: Improving species selection and spatial analysis of metropolitan datasets. Journal of Environmental Monitoring. 14(9): 2399-2410.
91. Fernandes, M.; Benger, S.; Sharma, S. K.; Gaylard, S.; Kildea, T.; Hoare, S.; Braley, M.; Irving, A. D. 2012. The use of delta N-15 signatures of translocated macroalgae to map coastal nutrient plumes: improving species selection and spatial analysis of metropolitan datasets. Journal of Environmental Monitoring. 14(9): 2399-2410.
92. Florisson, J. H.; Tweedley, J. R.; Walker, T. H. E.; Chaplin, J. A. 2018. Reef vision: A citizen science program for monitoring the fish faunas of artificial reefs. Fisheries Research. 206: 296-308.
93. Flynn, D. J. H.; Lynch, T. P.; Barrett, N. S.; Wong, L. S. C.; Devine, C.; Hughes, D. 2018. Gigapixel big data movies provide cost-effective seascape scale direct measurements of open-access coastal human use such as recreational fisheries. Ecology and Evolution. 8(18): 9372-9383.
94. Fornes, A.; Basterretxea, G.; Orfila, A.; Jordi, A.; Alvarez, A.; Tintore, J. 2006. Mapping Posidonia oceanica from IKONOS. ISPRS Journal of Photogrammetry and Remote Sensing. 60(5): 315-322.
95. Franklin, N. M.; Adams, M. S.; Stauber, J. L.; Lim, R. P. 2001. Development of an improved rapid enzyme inhibition bioassay with marine and freshwater microalgae using flow cytometry. Archives of Environmental Contamination and Toxicology. 40(4): 469-480.
96. Fuhrman, J. A.; Liang, X.; Noble, R. T. 2005. Rapid detection of enteroviruses in small volumes of natural waters by real-time quantitative reverse transcriptase PCR. Applied and Environmental Microbiology. 71(8): 4523-4530.
97. G?r?cs, Z.; Tamamitsu, M.; Bianco, V.; Wolf, P.; Roy, S.; Shindo, K.; Yanny, K.; Wu, Y.; Koydemir, H. C.; Rivenson, Y.; Ozcan, A. 2018. A deep learning-enabled portable imaging flow cytometer for cost-effective, high-throughput, and label-free analysis of natural water samples. Light: Science and Applications. 7(1).
98. Gallo, A.; Boni, R.; Tosti, E. 2018. Sperm viability assessment in marine invertebrates by fluorescent staining and spectrofluorimetry: A promising tool for assessing marine pollution impact. Ecotoxicology and Environmental Safety. 147: 407-412.
99. Gardner, J. P. A.; Struthers, C. D. 2013. Comparisons among survey methodologies to test for abundance and size of a highly targeted fish species. Journal of Fish Biology. 82(1): 242-262.
100. Gera, A.; Alcoverro, T.; Mascaró, O.; Pérez, M.; Romero, J. 2012. Exploring the utility of Posidonia oceanica chlorophyll fluorescence as an indicator of water quality within the European Water Framework Directive. Environmental Monitoring and Assessment. 184(6): 3675-3686.
101. Gerovasileiou, V.; Trygonis, V.; Sini, M.; Koutsoubas, D.; Voultsiadou, E. 2013. Three-dimensional mapping of marine caves using a handheld echosounder. Marine Ecology Progress Series. 486: 13-22.
102. Gill, D. A.; Oxenford, H. A.; Turner, R. A.; Schuhmann, P. W. 2019. Making the most of data-poor fisheries: Low cost mapping of small island fisheries to inform policy. Marine Policy. 101: 198-207.
103. Goetze, J. S.; Januchowski-Hartley, F. A.; Claudet, J.; Langlois, T. J.; Wilson, S. K.; Jupiter, S. D. 2017. Fish wariness is a more sensitive indicator to changes in fishing pressure than abundance, length or biomass. Ecological Applications. 27(4): 1178-1189.
104. Gordoa, A.; Boada, J.; García-Rubies, A.; Sagué, O. 2018. Free-diving underwater fish photography contests: A complementary tool for assessing littoral fish communities. Scientia Marina. 82(2): 95-106.
105. Goreau, T. J.; Fisher, T.; Perez, F.; Lockhart, K.; Hibbert, M.; Lewin, A. 2008. Turks and Caicos Islands 2006 coral reef assessment: Large-scale environmental and ecological interactions and their management implications. Revista de Biologia Tropical. 56: 25-49.
106. Gray, C. A.; Johnson, D. D.; Reynolds, D.; Rotherham, D. 2014. Development of rapid sampling procedures for an exploited bivalve in the swash zone on exposed ocean beaches. Fisheries Research. 154: 205-212.
107. Guzman, H. M.; Condit, R. 2017. Abundance of manatees in Panama estimated from side-scan sonar. Wildlife Society Bulletin. 41(3): 556-565.
108. Harper, L. R.; Buxton, A. S.; Rees, H. C.; Bruce, K.; Brys, R.; Halfmaerten, D.; Read, D. S.; Watson, H. V.; Sayer, C. D.; Jones, E. P.; Priestley, V.; Mächler, E.; Múrria, C.; Garcés-Pastor, S.; Medupin, C.; Burgess, K.; Benson, G.; Boonham, N.; Griffiths, R. A.; Lawson Handley, L.; Hänfling, B. 2019. Prospects and challenges of environmental DNA (eDNA) monitoring in freshwater ponds. Hydrobiologia. 826(1): 25-41.
109. Harper, L. R.; Lawson Handley, L.; Hahn, C.; Boonham, N.; Rees, H. C.; Gough, K. C.; Lewis, E.; Adams, I. P.; Brotherton, P.; Phillips, S.; Hänfling, B. 2018. Needle in a haystack? A comparison of eDNA metabarcoding and targeted qPCR for detection of the great crested newt (Triturus cristatus). Ecology and Evolution. 8(12): 6330-6341.
110. Heblinski, J.; Schmieder, K.; Heege, T.; Agyemang, T. K.; Sayadyan, H.; Vardanyan, L. 2011. High-resolution satellite remote sensing of littoral vegetation of Lake Sevan (Armenia) as a basis for monitoring and assessment. Hydrobiologia. 661(1): 97-111.
111. Hedley, J. D.; Roelfsema, C. M.; Chollett, I.; Harborne, A. R.; Heron, S. F.; Weeks, S. J.; Skirving, W. J.; Strong, A. E.; Mark Eakin, C.; Christensen, T. R. L.; Ticzon, V.; Bejarano, S.; Mumby, P. J. 2016. Remote sensing of coral reefs for monitoring and management: A review. Remote Sensing. 8(2).
112. Hering, D.; Borja, A.; Jones, J. I.; Pont, D.; Boets, P.; Bouchez, A.; Bruce, K.; Drakare, S.; Hänfling, B.; Kahlert, M.; Leese, F.; Meissner, K.; Mergen, P.; Reyjol, Y.; Segurado, P.; Vogler, A.; Kelly, M. 2018. Implementation options for DNA-based identification into ecological status assessment under the European Water Framework Directive. Water Research. 138: 192-205.
113. Hicks, B. J.; Stichbury, G. A.; Brabyn, L. K.; Allan, M. G.; Ashraf, S. 2013. Hindcasting water clarity from Landsat satellite images of unmonitored shallow lakes in the Waikato region, New Zealand. Environmental Monitoring and Assessment. 185(9): 7245-7261.
114. Hitz, G.; Pomerleau, F.; Garneau, M. E.; Pradalier, C.; Posch, T.; Pernthaler, J.; Siegwart, R. Y. 2012. Autonomous inland water monitoring: Design and application of a surface vessel. IEEE Robotics and Automation Magazine. 19(1): 62-72.
115. Hodge, J.; Longstaff, B.; Steven, A.; Thornton, P.; Ellis, P.; McKelvie, I. 2005. Rapid underway profiling of water quality in Queensland estuaries. Marine Pollution Bulletin. 51(43469): 113-118.
116. Hoyer, M. V.; Wellendorf, N.; Frydenborg, R.; Bartlett, D.; Canfield Jr, D. E. 2012. A comparison between professionally (Florida department of environmental protection) and volunteer (Florida LAKEWATCH) collected trophic state chemistry data in Florida. Lake and Reservoir Management. 28(4): 277-281.
117. Huang, J.; Bennett, W. W.; Teasdale, P. R.; Kankanamge, N. R.; Welsh, D. T. 2017. A modified DGT technique for the simultaneous measurement of dissolved inorganic nitrogen and phosphorus in freshwaters. Analytica Chimica Acta. 988: 17-26.
118. Hulley, E. N.; Tharmalingam, S.; Zarnke, A.; Boreham, D. R. 2019. Development and validation of probe-based multiplex real-time PCR assays for the rapid and accurate detection of freshwater fish species. PLoS ONE. 14(1).
119. Ingleton, T.; McMinn, A. 2012. Thermal plume effects: A multi-disciplinary approach for assessing effects of thermal pollution on estuaries using benthic diatoms and satellite imagery. Estuarine, Coastal and Shelf Science. 99: 132-144.
120. Iwamoto, S.; Checkley, Jr D. M.; Trivedi, M. M. 2001. REFLICS: Real-time flow imaging and classification system. Machine Vision and Applications. 13(1): 43478.
121. Jackson, G.; Cheng, Y. W.; Wakefield, C. B. 2012. An evaluation of the daily egg production method to estimate spawning biomass of snapper (Pagrus auratus) in inner Shark Bay, Western Australia, following more than a decade of surveys 1997-2007. Fisheries Research. 117-118: 22-34.
122. Jeunen, G. J.; Knapp, M.; Spencer, H. G.; Taylor, H. R.; Lamare, M. D.; Stat, M.; Bunce, M.; Gemmell, N. J. 2019. Species-level biodiversity assessment using marine environmental DNA metabarcoding requires protocol optimization and standardization. Ecology and Evolution. 9(3): 1323-1335.
123. Jiang, B.; Li, G.; Xing, Y.; Zhang, D.; Jia, J.; Cui, Z.; Luan, X.; Tang, H. 2017. A whole-cell bioreporter assay for quantitative genotoxicity evaluation of environmental samples. Chemosphere. 184: 384-392.
124. Johnston, P.; Baruwa, S.; Wyatt, R. 2015. Remote passive acoustic monitoring: Listening for whales and dolphins from the safety of land. Leading Edge. 34(12): 1516-1519.
125. Jones, G. E.; Glegg, G. E. 2004. Effective use of geophysical sensors for marine environmental assessment and habitat mapping. Environmental Studies. 10: 43601.
126. Jones, O. A. H.; Dondero, F.; Viarengo, A.; Griffin, J. L. 2008. Metabolic profiling of Mytilus galloprovincialis and its potential applications for pollution assessment. Marine Ecology Progress Series. 369: 169-179.
127. Jouvet, G.; Weidmann, Y.; Kneib, M.; Detert, M.; Seguinot, J.; Sakakibara, D.; Sugiyama, S. 2018. Short-lived ice speed-up and plume water flow captured by a VTOL UAV give insights into subglacial hydrological system of Bowdoin Glacier. Remote Sensing of Environment. 217: 389-399.
128. Jäger, P.; Pall, K.; Dumfarth, E. 2004. A method of mapping macrophytes in large lakes with regard to the requirements of the Water Framework Directive. Limnologica. 34(43467): 140-146.
129. Jönsson, M.; Abrahamson, A.; Brunström, B.; Brandt, I.; Ingebrigtsen, K.; Jørgensen, E. H. 2003. EROD activity in gill filaments of anadromous and marine fish as a biomarker of dioxin-like pollutants. Comparative Biochemistry and Physiology - C Toxicology and Pharmacology. 136(3): 235-243.
130. Kalaji, H. M.; Sytar, O.; Brestic, M.; Samborska, I. A.; Cetner, M. D.; Carpentier, C. 2016. Risk assessment of urban lake water quality based on in-situ cyanobacterial and total chlorophyll-a monitoring. Polish Journal of Environmental Studies. 25(2): 655-661.
131. Kanninen, A.; Vallinkoski, V. M.; Leka, J.; Marjomäki, T. J.; Hellsten, S.; Hämäläinen, H. 2013. A comparison of two methods for surveying aquatic macrophyte communities in boreal lakes: Implications for bioassessment. Aquatic Botany. 104: 88-100.
132. Karatayev, A. Y.; Mehler, K.; Burlakova, L. E.; Hinchey, E. K.; Warren, G. J. 2018. Benthic video image analysis facilitates monitoring of Dreissena populations across spatial scales. Journal of Great Lakes Research. 44(4): 629-638.
133. Karczewski, K.; Riss, H. W.; Meyer, E. I. 2017. Comparison of DNA-fingerprinting (T-RFLP) and high-throughput sequencing (HTS) to assess the diversity and composition of microbial communities in groundwater ecosystems. Limnologica. 67: 45-53.
134. Keskin, E.; Unal, E. M.; Atar, H. H. 2016. Detection of rare and invasive freshwater fish species using eDNA pyrosequencing: Lake Iznik ichthyofauna revised. Biochemical Systematics and Ecology. 67: 29-36.
135. Kim, M.; Yim, U. H.; Hong, S. H.; Jung, J. H.; Choi, H. W.; An, J.; Won, J.; Shim, W. J. 2010. Hebei Spirit oil spill monitored on site by fluorometric detection of residual oil in coastal waters off Taean, Korea. Marine Pollution Bulletin. 60(3): 383-389.
136. Kinzelman, J. L.; Dufour, A. P.; Wymer, L. J.; Rees, G.; Pond, K. R.; Bagley, R. C. 2006. Comparison of multiple point and composite sampling for monitoring bathing water quality. Lake and Reservoir Management. 22(2): 95-102.
137. Kle?ka, J.; Boukal, D. S. 2011. Lazy ecologist's guide to water beetle diversity: Which sampling methods are the best?. Ecological Indicators. 11(2): 500-508.
138. Knight-Jones, T. J. D.; Hauser, R.; Matthes, D.; Stärk, K. D. C. 2010. Evaluation of effectiveness and efficiency of wild bird surveillance for avian influenza. Veterinary Research. 41(4).
139. Knutsen, J. A.; Knutsen, H.; Rinde, E.; Christie, H.; Bodvin, T.; Dahl, E. 2010. Mapping biological resources in the coastal zone: An evaluation of methods in a pioneering study from Norway. Ambio. 39(2): 148-158.
140. Kobryn, H. T.; Wouters, K.; Beckley, L. E.; Heege, T. 2013. Ningaloo Reef: Shallow Marine Habitats Mapped Using a Hyperspectral Sensor. PLoS ONE. 8(7).
141. Koenig, C. C.; Stallings, C. D. 2015. A new compact rotating video system for rapid survey of reef fish populations. Bulletin of Marine Science. 91(3): 365-373.
142. Kong, R. Y. C.; Lee, S. K. Y.; Law, T. W. F.; Law, S. H. W.; Wu, R. S. S. 2002. Rapid detection of six types of bacterial pathogens in marine waters by multiplex PCR. Water Research. 36(11): 2802-2812.
143. Kong, R. Y. C.; Mak, M. M. H.; Wu, R. S. S. 2009. DNA technologies for monitoring waterborne pathogens: A revolution in water pollution monitoring. Ocean and Coastal Management. 52(7): 355-358.
144. Kopf, A.; Freudenthal, T.; Ratmeyer, V.; Bergenthal, M.; Lange, M.; Fleischmann, T.; Hammerschmidt, S.; Seiter, C.; Wefer, G. 2015. Simple, affordable, and sustainable borehole observatories for complex monitoring objectives. Geoscientific Instrumentation, Methods and Data Systems. 4(1): 99-109.
145. Kotilainen, A. T.; Kaskela, A. M. 2017. Comparison of airborne LiDAR and shipboard acoustic data in complex shallow water environments: Filling in the white ribbon zone. Marine Geology. 385: 250-259.
146. Koydemir, H. C.; Feng, S.; Liang, K.; Nadkarni, R.; Benien, P.; Ozcan, A. 2017. Comparison of supervised machine learning algorithms for waterborne pathogen detection using mobile phone fluorescence microscopy. Nanophotonics. 6(4): 731-741.
147. Kozma Törökné, A.; László, E.; Chorus, I.; Fastner, J.; Heinze, R.; Padisák, J.; Barbosa, F. A. 2000. Water quality monitoring by Thamnotoxkit F(TM) including cyanobacterial blooms. Water Science and Technology. 42: 381-385.
148. Krsti?, S.; Svir?ev, Z.; Levkov, Z.; Nakov, T. 2007. Selecting appropriate bioindicators regarding Water Framework Directive guidelines for freshwaters - A Macedonian experience. International Journal on Algae. 9(1): 41-63.
149. Kuzukiran, O.; Yurdakok-Dikmen, B.; Totan, F. E.; Celik, C.; Orhan, E. C.; Bilir, E. K.; Kara, E.; Filazi, A. 2016. Analytical method development and validation for some persistent organic pollutants in water and sediments by gas chromatography mass spectrometry. International Journal of Environmental Research. 10(3): 401-410.
150. Kvernevik, T. I.; Zambri Mohd Akhir, M.; Studholme, J. 2002. A low-cost procedure for automatic seafloor mapping, with particular reference to coral reef conservation in developing nations. Hydrobiologia. 474: 67-79.
151. LaCommare, K. S.; Brault, S.; Self-Sullivan, C.; Hines, E. M. 2012. Trend detection in a boat-based method for monitoring sirenians: Antillean manatee case study. Biological Conservation. 152: 169-177.
152. Lagarde, F.; Jaffrezic-Renault, N. 2011. Cell-based electrochemical biosensors for water quality assessment. Analytical and Bioanalytical Chemistry. 400(4): 947-964.
153. Lampadariou, N.; Karakassis, I.; Pearson, T. H. 2005. Cost/benefit analysis of a benthic monitoring programme of organic benthic enrichment using different sampling and analysis methods. Marine Pollution Bulletin. 50(12): 1606-1618.
154. Lanzén, A.; Lekang, K.; Jonassen, I.; Thompson, E. M.; Troedsson, C. 2016. High-throughput metabarcoding of eukaryotic diversity for environmental monitoring of offshore oil-drilling activities. Molecular ecology. 25(17): 4392-4406.
155. Lanzén, A.; Lekang, K.; Jonassen, I.; Thompson, E. M.; Troedsson, C. 2017. DNA extraction replicates improve diversity and compositional dissimilarity in metabarcoding of eukaryotes in marine sediments. PLoS ONE. 12(6).
156. Laran, S.; Authier, M.; Canneyt, O. V.; Dorémus, G.; Watremez, P.; Ridoux, V. 2017. A comprehensive survey of pelagic megafauna: Their distribution, densities, and taxonomic richness in the tropical Southwest Indian ocean. Frontiers in Marine Science. 4(MAY).
157. Latini, A. O.; Petrere Júnior, M. 2018. Efficiency of rapid field methods for detecting non-native fish in Eastern Brazilian lakes. Hydrobiologia. 817(1): 85-96.
158. Le Reste, S.; Dutreuil, V.; André, X.; Thierry, V.; Renaut, C.; Le Traon, P. Y.; Maze, G. 2016. Deep-Arvor: A new profiling float to extend the argo observations down to 4000-m depth. Journal of Atmospheric and Oceanic Technology. 33(5): 1039-1055.
159. Lembke, C.; Grasty, S.; Silverman, A.; Broadbent, H.; Butcher, S.; Murawski, S. 2017. The Camera-Based Assessment Survey System (C-BASS): A towed camera platform for reef fish abundance surveys and benthic habitat characterization in the Gulf of Mexico. Continental Shelf Research. 151: 62-71.
160. Leonardo, S.; Toldrà, A.; Rambla-Alegre, M.; Fernández-Tejedor, M.; Andree, K. B.; Ferreres, L.; Campbell, K.; Elliott, C. T.; O'Sullivan, C. K.; Pazos, Y.; Diogène, J.; Campàs, M. 2018. Self-assembled monolayer-based immunoassays for okadaic acid detection in seawater as monitoring tools. Marine Environmental Research. 133: 43630.
161. Léopold, M.; Cakacaka, A.; Meo, S.; Sikolia, J.; Lecchini, D. 2009. Evaluation of the effectiveness of three underwater reef fish monitoring methods in Fiji. Biodiversity and Conservation. 18(13): 3367-3382.
162. Leujak, W.; Ormond, R. F. G. 2007. Comparative accuracy and efficiency of six coral community survey methods. Journal of Experimental Marine Biology and Ecology. 351(43467): 168-187.
163. Li, J.; Hatton-Ellis, T. W.; Lawson Handley, L. J.; Kimbell, H. S.; Benucci, M.; Peirson, G.; Hänfling, B. 2019. Ground-truthing of a fish-based environmental DNA metabarcoding method for assessing the quality of lakes. Journal of Applied Ecology. .
164. Lim, A.; Kane, A.; Arnaubec, A.; Wheeler, A. J. 2018. Seabed image acquisition and survey design for cold water coral mound characterisation. Marine Geology. 395: 22-32.
165. Lintern, A.; Leahy, P. J.; Heijnis, H.; Zawadzki, A.; Gadd, P.; Jacobsen, G.; Deletic, A.; McCarthy, D. T. 2016. Identifying heavy metal levels in historical flood water deposits using sediment cores. Water Research. 105: 34-46.
166. Litaker, R. W.; Stewart, T. N.; Eberhart, B. T. L.; Wekell, J. C.; Trainer, V. L.; Kudela, R. M.; Miller, P. E.; Roberts, A.; Hertz, C.; Johnson, T. A.; Frankfurter, G.; Smith, G. J.; Schnetzer, A.; Schumacker, J.; Bastian, J. L.; Odell, A.; Gentien, P.; Le Gal, D.; Hardison, I. R.; Tester, P. A. 2008. RAPID ENZYME-LINKED IMMUNOSORBENT ASSAY FOR DETECTION OF THE ALGAL TOXIN DOMOIC ACID. Journal of Shellfish Research. 27(5): 1301-1310.
167. Lu, Y.; Liu, J.; Li, J.; Bruesehoff, P. J.; Pavot, C. M. B.; Brown, A. K. 2003. New highly sensitive and selective catalytic DNA biosensors for metal ions. Biosensors and Bioelectronics. 18(43591): 529-540.
168. Lydersen, C.; Nost, O. A.; Lovell, P.; McConnell, B. J.; Gammelsrod, T.; Hunter, C.; Fedak, M. A.; Kovacs, K. M. 2002. Salinity and temperature structure of a freezing Arctic fjord - monitored by white whales (Delphinapterus leucas). Geophysical Research Letters. 29(23).
169. Mackinson, S.; Freeman, S.; Flatt, R.; Meadows, B. 2004. Improved acoustic surveys that save time and money: integrating fisheries and ground-discrimination acoustic technologies. Journal of Experimental Marine Biology and Ecology. 305(2): 129-140.
170. Mahmood, A.; Bennamoun, M.; An, S. J.; Sohel, F. A.; Boussaid, F.; Hovey, R.; Kendrick, G. A.; Fisher, R. B. 2019. Deep Image Representations for Coral Image Classification. Ieee Journal of Oceanic Engineering. 44(1): 121-131.
171. Mallet, D.; Pelletier, D. 2014. Underwater video techniques for observing coastal marine biodiversity: A review of sixty years of publications (1952-2012). Fisheries Research. 154: 44-62.
172. Malley, D. F.; Williams, P. 2014. Analysis of sediments and suspended material in lake ecosystems using near-infrared spectroscopy: A review. Aquatic Ecosystem Health and Management. 17(4): 447-453.
173. Mancini, A.; Elsadek, I.; Madon, B. 2015. When simple is better: Comparing two sampling methods to estimate green turtles abundance at coastal feeding grounds. Journal of Experimental Marine Biology and Ecology. 465: 113-120.
174. Martinez-Haro, M.; Acevedo, P.; Pais-Costa, A. J.; Taggart, M. A.; Martins, I.; Ribeiro, R.; Marques, J. C. 2016. Assessing estuarine quality: A cost-effective in situ assay with amphipods. Environmental Pollution. 212: 382-391.
175. Martinez-Haro, M.; Moreira-Santos, M.; Marques, J. C.; Ribeiro, R. 2014. A short-term laboratory and in situ sediment assay based on the postexposure feeding of the estuarine isopod Cyathura carinata. Environmental Research. 134: 242-250.
176. Martinis, E. M.; Escudero, L. B.; Salvarezza, R.; Calderón, M. F.; Ibañez, F. J.; Wuilloud, R. G. 2013. Liquid-liquid microextraction based on a dispersion of Pd nanoparticles combined with ETAAS for sensitive Hg determination in water samples. Talanta. 108: 46-52.
177. Mazurkiewicz, M.; Górska, B.; Jankowska, E.; W?odarska-Kowalczuk, M. 2016. Assessment of nematode biomass in marine sediments: A semi-automated image analysis method. Limnology and Oceanography: Methods. 14(12): 816-827.
178. Melnik, S.; Neumann, A. C.; Karongo, R.; Dirndorfer, S.; Stübler, M.; Ibl, V.; Niessner, R.; Knopp, D.; Stoger, E. 2018. Cloning and plant-based production of antibody MC10E7 for a lateral flow immunoassay to detect [4-arginine]microcystin in freshwater. Plant Biotechnology Journal. 16(1): 27-38.
179. Melo, A.; Ferreira, Implvo; Mansilha, C. 2015. Application of a fast and cost-effective in situ derivatization method prior to gas chromatography with mass spectrometry to monitor endocrine disruptors in water matrices. Journal of Separation Science. 38(11): 1983-1989.
180. Michailova, P.; Sella, G.; Petrova, N. 2012. Chironomids (Diptera) and their salivary gland chromosomes as indicators of trace-metal genotoxicity. Italian Journal of Zoology. 79(2): 218-230.
181. Minamoto, T.; Fukuda, M.; Katsuhara, K. R.; Fujiwara, A.; Hidaka, S.; Yamamoto, S.; Takahashi, K.; Masuda, R. 2017. Environmental DNA reflects spatial and temporal jellyfish distribution. PLoS ONE. 12(2).
182. Minchin, D.; Olenin, S.; Liu, T. K.; Cheng, M.; Huang, S. C. 2016. Rapid assessment of target species: Byssate bivalves in a large tropical port. Marine Pollution Bulletin. 112(43467): 177-182.
183. Misra, A.; Balaji, R. 2017. Simple Approaches to Oil Spill Detection Using Sentinel Application Platform (SNAP)-Ocean Application Tools and Texture Analysis: A Comparative Study. Journal of the Indian Society of Remote Sensing. 45(6): 1065-1075.
184. Miya, M.; Sato, Y.; Fukunaga, T.; Sado, T.; Poulsen, J. Y.; Sato, K.; Minamoto, T.; Yamamoto, S.; Yamanaka, H.; Araki, H.; Kondoh, M.; Iwasaki, W. 2015. MiFish, a set of universal PCR primers for metabarcoding environmental DNA from fishes: Detection of more than 230 subtropical marine species. Royal Society Open Science. 2(7).
185. Molognoni, L.; Dos Santos, J. N.; Kleemann, C. R.; Costa, A. C. O.; Hoff, R. B.; Daguer, H. 2019. Cost-Effective and High-Reliability Analytical Approach for Multitoxin Screening in Bivalve Mollusks by Liquid Chromatography Coupled to Tandem Mass Spectrometry. Journal of Agricultural and Food Chemistry. 67(9): 2691-2699.
186. Moreira, S. M.; Moreira-Santos, M.; Ribeiro, R.; Guilhermino, L. 2004. The 'Coral Bulker' fuel oil spill on the north coast of portugal: Spatial and temporal biomarker responses in Mytilus galloprovincialis. Ecotoxicology. 13(7): 619-630.
187. Moreira-Santos, M.; Soares, A. M. V. M.; Ribeiro, R. 2004. A phytoplankton growth assay for routine in situ environmental assessments. Environmental Toxicology and Chemistry. 23(6): 1549-1560.
188. Mortensen, L. O.; Ulrich, C.; Olesen, H. J.; Bergsson, H.; Berg, C. W.; Tzamouranis, N.; Dalskov, J. 2017. Effectiveness of fully documented fisheries to estimate discards in a participatory research scheme. Fisheries Research. 187: 150-157.
189. Mosindy, T. E.; Duffy, M. J. 2007. The use of angler diary surveys to evaluate long-term changes in muskellunge populations on Lake of the Woods, Ontario. Environmental Biology of Fishes. 79(43467): 71-83.
190. Moxley, J. H.; Bogomolni, A.; Hammill, M. O.; Moore, K. M. T.; Polito, M. J.; Sette, L.; Sharp, W. B.; Waring, G. T.; Gilbert, J. R.; Halpin, P. N.; Johnston, D. W. 2017. Google haul out: Earth observation imagery and digital aerial surveys in coastal wildlife management and abundance estimation. BioScience. 67(8): 760-768.
191. Mueller, K. W. 2003. A comparison of electrofishing and scuba diving to sample black bass in western Washington lakes. North American Journal of Fisheries Management. 23(2): 632-639.
192. Mumby, P. J.; Edwards, A. J. 2002. Mapping marine environments with IKONOS imagery: enhanced spatial resolution can deliver greater thematic accuracy. Remote Sensing of Environment. 82(43499): 248-257.
193. Munksgaard, N. C.; Moir, C. M.; Parry, D. L. 2002. Bio-monitoring using lead isotope ratios in seagrass and oysters. Marine Technology Society Journal. 36(1): 52-54.
194. Murphy, H. M.; Jenkins, G. P. 2010. Observational methods used in marine spatial monitoring of fishes and associated habitats: A review. Marine and Freshwater Research. 61(2): 236-252.
195. Murray, S. A.; Wiese, M.; Stüken, A.; Brett, S.; Kellmann, R.; Hallegraeff, G.; Neilan, B. A. 2011. SxtA-based quantitative molecular assay to identify saxitoxin-producing harmful algal blooms in marine waters. Applied and Environmental Microbiology. 77(19): 7050-7057.
196. Nagai, S.; Itakura, S. 2012. Specific detection of the toxic dinoflagellates Alexandrium tamarense and Alexandrium catenella from single vegetative cells by a loop-mediated isothermal amplification method. Marine Genomics. 7: 43-49.
197. Nendza, M. 2002. Inventory of marine biotest methods for the evaluation of dredged material and sediments. Chemosphere. 48(8): 865-883.
198. Neto, A. A.; Mota, B. B.; Belem, A. L.; Albuquerque, A. L.; Capilla, R. 2016. Seismic peak amplitude as a predictor of TOC content in shallow marine sediments. Geo-Marine Letters. 36(5): 395-403.
199. Noyer, C.; Abot, A.; Trouilh, L.; Leberre, V. A.; Dreanno, C. 2015. Phytochip: Development of a DNA-microarray for rapid and accurate identification of Pseudo-nitzschia spp and other harmful algal species. Journal of Microbiological Methods. 112: 55-66.
200. O'Driscoll, R. L.; Macaulay, G. J. 2005. Using fish-processing time to carry out acoustic surveys from commercial vessels. ICES Journal of Marine Science. 62(2): 295-305.
201. Ouyang, H.; Shu, Q.; Wang, W.; Wang, Z.; Yang, S.; Wang, L.; Fu, Z. 2016. An ultra-facile and label-free immunoassay strategy for detection of copper (II) utilizing chemiluminescence self-enhancement of Cu (II)-ethylenediaminetetraacetate chelate. Biosensors and Bioelectronics. 85: 157-163.
202. Ozsoy-Cicek, B. 2014. OIL SPILL DETECTION FROM RADARSAT-1 SYNTHETIC APERTURE RADAR IMAGERY AT NORTHERN ENTRY OF BOSPORUS STRAIT, TURKEY. Fresenius Environmental Bulletin. 23(11A): 2909-2918.
203. Pelletier, D.; Leleu, K.; Mou-Tham, G.; Guillemot, N.; Chabanet, P. 2011. Comparison of visual census and high definition video transects for monitoring coral reef fish assemblages. Fisheries Research. 107(43468): 84-93.
204. Pergent, G.; Monnier, B.; Clabaut, P.; Gascon, G.; Pergent-Martini, C.; Valette-Sansevin, A. 2017. Innovative method for optimizing Side-Scan Sonar mapping: The blind band unveiled. Estuarine, Coastal and Shelf Science. 194: 77-83.
205. Perini, F.; Bastianini, M.; Capellacci, S.; Pugliese, L.; DiPoi, E.; Cabrini, M.; Buratti, S.; Marini, M.; Penna, A. 2018. Molecular methods for cost-efficient monitoring of HAB (harmful algal bloom) dinoflagellate resting cysts. Marine Pollution Bulletin. .
206. Piermattei, V.; Madonia, A.; Bonamano, S.; Martellucci, R.; Bruzzone, G.; Ferretti, R.; Odetti, A.; Azzaro, M.; Zappalà, G.; Marcelli, M. 2018. Cost-effective technologies to study the arctic ocean environment†. Sensors (Switzerland). 18(7).
207. Pinna, M.; Marini, G.; Rosati, I.; Neto, J. M.; Patrício, J.; Marques, J. C.; Basset, A. 2013. The usefulness of large body-size macroinvertebrates in the rapid ecological assessment of Mediterranean lagoons. Ecological Indicators. 29: 48-61.
208. Pirotta, V.; Smith, A.; Ostrowski, M.; Russell, D.; Jonsen, I. D.; Grech, A.; Harcourt, R. 2017. An economical Custom-Built drone for assessing whale health. Frontiers in Marine Science. 4(DEC).
209. Pitois, S. G.; Tilbury, J.; Bouch, P.; Close, H.; Barnett, S.; Culverhouse, P. F. 2018. Comparison of a cost-effective integrated plankton sampling and imaging instrument with traditional systems for mesozooplankton sampling in the Celtic Sea. Frontiers in Marine Science. 5(JAN).
210. Polak-Juszczak, L. 2012. Bioaccumulation of mercury in the trophic chain of flatfish from the Baltic Sea. Chemosphere. 89(5): 585-591.
211. Pollard, P. C. 2012. Fluorescence instrument for in situ monitoring of viral abundance in water, wastewater and recycled water. Journal of Virological Methods. 181(1): 97-102.
212. Popescu, G.; Iordan, D. 2018. AN OVERALL VIEW OF LIDAR AND SONAR SYSTEMS USED IN GEOMATICS APPLICATIONS FOR HYDROLOGY. Scientific Papers-Series E-Land Reclamation Earth Observation & Surveying Environmental Engineering. 7: 174-181.
213. Porst, G.; Bader, S.; Münch, E.; Pusch, M. 2012. Sampling approaches for the assessment of shoreline development based on littoral macroinvertebrates: The case of Lake Werbellin, Germany. Fundamental and Applied Limnology. 180(2): 123-131.
214. Porst, G.; Miler, O.; Donohue, L.; Jurca, T.; Pilotto, F.; Brauns, M.; Solimini, A.; Pusch, M. 2016. Efficient sampling methodologies for lake littoral invertebrates in compliance with the European Water Framework Directive. Hydrobiologia. 767(1): 207-220.
215. Prabhudesai, R. G.; Joseph, A.; Agarvadekar, Y.; Dabholkar, N.; Mehra, P.; Gouveia, A.; Tengali, S.; Vijaykumar; Parab, A. 2006. Development and implementation of cellular-based real-time reporting and Internet accessible coastal sea-level gauge - A vital tool for monitoring storm surge and tsunami. Current Science. 90(10): 1413-1418.
216. Puhr, K.; Schultz, S.; Pikelj, K.; Petricioli, D.; Bakran-Petricioli, T. 2014. The performance, application and integration of various seabed classification systems suitable for mapping Posidonia oceanica (L.) Delile meadows. Science of the Total Environment. 470-471: 364-378.
217. Qi, Y.; Xiu, F. R.; Yu, G.; Huang, L.; Li, B. 2017. Simple and rapid chemiluminescence aptasensor for Hg2+ in contaminated samples: A new signal amplification mechanism. Biosensors and Bioelectronics. 87: 439-446.
218. Qing, Z.; He, X.; Wang, K.; Zou, Z.; Yang, X.; Huang, J.; Yan, G. 2012. Colorimetric multiplexed analysis of mercury and silver ions by using a unimolecular DNA probe and unmodified gold nanoparticles. Analytical Methods. 4(10): 3320-3325.
219. Rajamani, L.; Marsh, H. 2015. Mapping seagrass cost-effectively in the Coral Triangle: Sabah, Malaysia as a case study. Pacific Conservation Biology. 21(2): 113-121.
220. Ramkilowan, A.; Chetty, N.; Lysko, M.; Griffith, D. 2013. Optical Detectors for Integration into a Low Cost Radiometric Device for In-Water Applications: A Feasibility Study. Journal of the Indian Society of Remote Sensing. 41(3): 531-538.
221. Ransome, E.; Geller, J. B.; Timmers, M.; Leray, M.; Mahardini, A.; Sembiring, A.; Collins, A. G.; Meyer, C. P. 2017. The importance of standardization for biodiversity comparisons: A case study using autonomous reef monitoring structures (ARMS) and metabarcoding to measure cryptic diversity on Mo'orea coral reefs, French Polynesia. PLoS ONE. 12(4).
222. Rich, V. I.; Pham, V. D.; Eppley, J.; Shi, Y.; DeLong, E. F. 2011. Time-series analyses of Monterey Bay coastal microbial picoplankton using a 'genome proxy' microarray. Environmental Microbiology. 13(1): 116-134.
223. Rickerby, D. G. 2009. Potental application of biosensor networks for monitoring aquatic sytems in support of the water framework directive. Bollettino Di Geofisica Teorica Ed Applicata. 50(4): 341-360.
224. Rishworth, G. M.; Tremblay, Y.; Green, D. B.; Pistorius, P. A. 2014. An automated approach towards measuring time-activity budgets in colonial seabirds. Methods in Ecology and Evolution. 5(9): 854-863.
225. Risk, M. J.; Lapointe, B. E.; Sherwood, O. A.; Bedford, B. J. 2009. The use of ?15N in assessing sewage stress on coral reefs. Marine Pollution Bulletin. 58(6): 793-802.
226. Roelfsema, C. M.; Phinn, S. R.; Dennison, W. C.; Dekker, A. G.; Brando, V. E. 2006. Monitoring toxic cyanobacteria Lyngbya majuscula (Gomont) in Moreton Bay, Australia by integrating satellite image data and field mapping. Harmful Algae. 5(1): 45-56.
227. Romagnan, J. B.; Aldamman, L.; Gasparini, S.; Nival, P.; Aubert, A.; Jamet, J. L.; Stemmann, L. 2016. High frequency mesozooplankton monitoring: Can imaging systems and automated sample analysis help us describe and interpret changes in zooplankton community composition and size structure — An example from a coastal site. Journal of Marine Systems. 162: 18-28.
228. Rotherham, D.; Gray, C. A.; Johnson, D. D.; Lokys, P. 2008. Effects of diel period and tow duration on estuarine fauna sampled with a beam trawl over bare sediment: Consequences for designing more reliable and efficient surveys. Estuarine, Coastal and Shelf Science. 78(1): 179-189.
229. Rotherham, D.; Underwood, A. J.; Chapman, M. G.; Gray, C. A. 2007. A strategy for developing scientific sampling tools for fishery-independent surveys of estuarine fish in New South Wales, Australia. ICES Journal of Marine Science. 64(8): 1512-1516.
230. Ruiz, J.; Batty, A.; Chavance, P.; McElderry, H.; Restrepo, V.; Sharples, P.; Santos, J.; Urtizberea, A. 2014. Electronic monitoring trials on in the tropical tuna purse-seine fishery. ICES Journal of Marine Science. 72(4): 1201-1213.
231. Rundberget, T.; Gustad, E.; Samdal, I. A.; Sandvik, M.; Miles, C. O. 2009. A convenient and cost-effective method for monitoring marine algal toxins with passive samplers. Toxicon. 53(5): 543-550.
232. Ruse, L. 2010. Classification of nutrient impact on lakes using the chironomid pupal exuvial technique. Ecological Indicators. 10(3): 594-601.
233. Ruse, L. 2011. Lake acidification assessed using chironomid pupal exuviae. Fundamental and Applied Limnology. 178(4): 267-286.
234. Ryan, P. G.; Moore, C. J.; Van Franeker, J. A.; Moloney, C. L. 2009. Monitoring the abundance of plastic debris in the marine environment. Philosophical Transactions of the Royal Society B: Biological Sciences. 364(1526): 1999-2012.
235. Ränäk, M.; Saari, L.; Hario, M.; Hnninen, J.; Lehikoinen, E. 2011. Breeding success and breeding population trends of waterfowl: Implications for monitoring. Wildlife Biology. 17(3): 225-239.
236. Sánchez-Gendriz, I.; Padovese, L. R. 2017. Temporal and spectral patterns of fish choruses in two protected areas in southern Atlantic. Ecological Informatics. 38: 31-38.
237. Santos, M. M.; Solé, M.; Lima, D.; Hambach, B.; Ferreira, A. M.; Reis-Henriques, M. A. 2010. Validating a multi-biomarker approach with the shanny Lipophrys pholis to monitor oil spills in European marine ecosystems. Chemosphere. 81(6): 685-691.
238. Schaeffer, B. A.; Bailey, S. W.; Conmy, R. N.; Galvin, M.; Ignatius, A. R.; Johnston, J. M.; Keith, D. J.; Lunetta, R. S.; Parmar, R.; Stumpf, R. P.; Urquhart, E. A.; Werdell, P. J.; Wolfe, K. 2018. Mobile device application for monitoring cyanobacteria harmful algal blooms using Sentinel-3 satellite Ocean and Land Colour Instruments. Environmental Modelling and Software. 109: 93-103.
239. Schmidt, D. J.; Espinoza, T.; Real, K.; Dunlop, A.; Kennard, M.; Hughes, J. M. 2018. Improved genetic markers for monitoring recruitment dynamics in the endangered Mary River cod (Maccullochella mariensis). Journal of Applied Ichthyology. 34(3): 633-637.
240. Schouten, P. W.; Parisi, A. V. 2012. Underwater deployment of the polyphenylene oxide dosimeter combined with a neutral density filter to measure long-term solar UVB exposures. Journal of Photochemistry and Photobiology B: Biology. 112: 31-36.
241. Seoane, S.; Garmendia, M.; Revilla, M.; Borja, Á; Franco, J.; Orive, E.; Valencia, V. 2011. Phytoplankton pigments and epifluorescence microscopy as tools for ecological status assessment in coastal and estuarine waters, within the Water Framework Directive. Marine Pollution Bulletin. 62(7): 1484-1497.
242. Sevilla, E.; Yuste, L.; Rojo, F. 2015. Marine hydrocarbonoclastic bacteria as whole-cell biosensors for n-alkanes. Microbial Biotechnology. 8(4): 693-706.
243. Sheehan, E. V.; Stevens, T. F.; Attrill, M. J. 2010. A quantitative, non-destructive methodology for habitat characterisation and benthic monitoring at offshore renewable energy developments. PLoS ONE. 5(12).
244. Shin, H. J. 2011. Genetically engineered microbial biosensors for in situ monitoring of environmental pollution. Applied Microbiology and Biotechnology. 89(4): 867-877.
245. Shinohara, M.; Uchida, K.; Shimada, S.; Tomioka, K.; Suzuki, N.; Minegishi, T.; Kawahashi, S.; Yoshikawa, Y.; Ohashi, N. 2011. Novel concentration method for the detection of norovirus and sapovirus from water using minute particles of amorphous calcium phosphate. Journal of Medical Microbiology. 60(6): 780-786.
246. Siegenthaler, A.; Wangensteen, O. S.; Soto, A. Z.; Benvenuto, C.; Corrigan, L.; Mariani, S. 2019. Metabarcoding of shrimp stomach content: Harnessing a natural sampler for fish biodiversity monitoring. Molecular Ecology Resources. 19(1): 206-220.
247. Silva, S.; Vieira-Lanero, R.; Barca, S.; Servia, M. J.; Sánchez-Hernández, J.; Cobo, F. 2014. Single pass electrofishing method for assessment and monitoring of larval lamprey populations. Limnetica. 33(2): 217-226.
248. Slimani, N.; Sánchez-Fernández, D.; Guilbert, E.; Boumaïza, M.; Guareschi, S.; Thioulouse, J. 2019. Assessing potential surrogates of macroinvertebrate diversity in North-African Mediterranean aquatic ecosystems. Ecological Indicators. 101: 324-329.
249. Smale, D. A. 2010. Monitoring marine macroalgae: The influence of spatial scale on the usefulness of biodiversity surrogates. Diversity and Distributions. 16(6): 985-995.
250. Smale, D. A.; Langlois, T. J.; Kendrick, G. A.; Meeuwig, J. J.; Harvey, E. S. 2011. From fronds to fish: The use of indicators for ecological monitoring in marine benthic ecosystems, with case studies from temperate Western Australia. Reviews in Fish Biology and Fisheries. 21(3): 311-337.
251. Smith, P. A. 2003. A cost-effective survey of fish occurring in a linear waterbody. Water and Environment Journal. 17(3): 181-186.
252. Smith, R. N.; Das, J.; Heidarsson, H.; Pereira, A. M.; Arrichiello, F.; Cetni?, I.; Darjany, L.; Garneau, M. E.; Howard, M. D.; Oberg, C.; Ragan, M.; Seubert, E.; Smith, E. C.; Stauffer, B. A.; Schnetzer, A.; Toro-Farmer, G.; Caron, D. A.; Jones, B. H.; Sukhatme, G. S. 2010. USC CINAPS builds bridges: Observing and monitoring the Southern California bight. IEEE Robotics and Automation Magazine. 17(1): 20-30.
253. Solberg, A. H. S. 2012. Remote sensing of ocean oil-spill pollution. Proceedings of the IEEE. 100(10): 2931-2945.
254. Song, X.; Li, H.; Lin, X.; Chen, X.; Guo, X.; Tian, J. 2009. Sea experiments of the underway conductivity-temperature-depth prototype made in China. Journal of Ocean University of China. 8(4): 409-415.
255. Southwell, C.; Emmerson, L. 2015. Remotely-operating camera network expands Antarctic seabird observations of key breeding parameters for ecosystem monitoring and management. Journal for Nature Conservation. 23: 43473.
256. Souza, G. B. G.; Barros, F. 2015. Analysis of sampling methods of estuarine benthic macrofaunal assemblages: sampling gear, mesh size, and taxonomic resolution. Hydrobiologia. 743(1): 157-174.
257. Stahr, K. J.; Knudsen, R. L. 2018. Evaluating the Efficacy of Using Time-Lapse Cameras to Assess Angling Use: An Example from a High-Use Metropolitan Reservoir in Arizona. North American Journal of Fisheries Management. 38(2): 327-333.
258. Stern, R. F.; Picard, K. T.; Hamilton, K. M.; Walne, A.; Tarran, G. A.; Mills, D.; McQuatters-Gollop, A.; Edwards, M. 2015. Novel lineage patterns from an automated water sampler to probe marine microbial biodiversity with ships of opportunity. Progress in Oceanography. 137: 409-420.
259. Stoeck, T.; Fruhe, L.; Forster, D.; Cordier, T.; Martins, C. I. M.; Pawlowski, J. 2018. Environmental DNA metabarcoding of benthic bacterial communities indicates the benthic footprint of salmon aquaculture. Marine Pollution Bulletin. 127: 139-149.
260. Strindberg, S.; Coleman, R. A.; Perez, V. R. B.; Campbell, C. L.; Majil, I.; Gibson, J. 2016. In-water assessments of sea turtles at Glover's Reef Atoll, Belize. Endangered Species Research. 31(1): 211-225.
261. Stringell, T. B.; Millar, C. P.; Sanderson, W. G.; Westcott, S. M.; McMath, M. J. 2014. When aerial surveys will not do: Grey seal pup production in cryptic habitats of Wales. Journal of the Marine Biological Association of the United Kingdom. 94(6): 1155-1159.
262. Sun, C. H. J.; Fine, L. 2016. A cost-effective discards-proportional at-sea monitoring allocation scheme for the groundfish fishery in New England. Marine Policy. 66: 75-82.
263. Sykora-Bodie, S. T.; Bezy, V.; Johnston, D. W.; Newton, E.; Lohmann, K. J. 2017. Quantifying Nearshore Sea Turtle Densities: Applications of Unmanned Aerial Systems for Population Assessments. Scientific Reports. 7(1).
264. Tagliapietra, D.; Cornello, M.; Ghirardini, A. V. 2005. Monitoring transitional waters using reduced benthic assemblages. Environment International. 31(7): 1089-1093.
265. Teixeira, J. B.; Martins, A. S.; Pinheiro, H. T.; Secchin, N. A.; Leão de Moura, R.; Bastos, A. C. 2013. Traditional Ecological Knowledge and the mapping of benthic marine habitats. Journal of Environmental Management. 115: 241-250.
266. Terán-Baamonde, J.; Carlosena, A.; Soto-Ferreiro, R. M.; Andrade, J. M.; Prada, D. 2017. Fast assessment of bioaccessible metallic contamination in marine sediments. Marine Pollution Bulletin. 125(43467): 310-317.
267. Tercier-Waeber, M. L.; Confalonieri, F.; Riccardi, G.; Sina, A.; Nöel, S.; Buffle, J.; Graziottin, F. 2005. Multi Physical - Chemical profiler for real-time in situ monitoring of trace metal speciation and master variables: Development, validation and field applications. Marine Chemistry. 97(43528): 216-235.
268. Tercier-Waeber, M. L.; Taillefert, M. 2008. Remote in situ voltammetric techniques to characterize the biogeochemical cycling of trace metals in aquatic systems. Journal of Environmental Monitoring. 10(1): 30-54.
269. Thompson, B. W.; Riddle, M. J.; Stark, J. S. 2003. Cost-efficient methods for marine pollution monitoring at Casey Station, East Antarctica: The choice of sieve mesh-size and taxonomic resolution. Marine Pollution Bulletin. 46(2): 232-243.
270. Thomsen, P. F.; Willerslev, E. 2015. Environmental DNA - An emerging tool in conservation for monitoring past and present biodiversity. Biological Conservation. 183: 43573.
271. Toma, D. M.; Masmitja, I.; del Río, J.; Martinez, E.; Artero-Delgado, C.; Casale, A.; Figoli, A.; Pinzani, D.; Cervantes, P.; Ruiz, P.; Memè, S.; Delory, E. 2018. Smart embedded passive acoustic devices for real-time hydroacoustic surveys. Measurement: Journal of the International Measurement Confederation. 125: 592-605.
272. Trasviña-Moreno, C. A.; Blasco, R.; Marco, Á; Casas, R.; Trasviña-Castro, A. 2017. Unmanned aerial vehicle based wireless sensor network for marine-coastal environment monitoring. Sensors (Switzerland). 17(3).
273. Turemis, M.; Silletti, S.; Pezzotti, G.; Sanchís, J.; Farré, M.; Giardi, M. T. 2018. Optical biosensor based on the microalga-paramecium symbiosis for improved marine monitoring. Sensors and Actuators, B: Chemical. 270: 424-432.
274. Turner, C. R.; Miller, D. J.; Coyne, K. J.; Corush, J. 2014. Improved methods for capture, extraction, and quantitative assay of environmental DNA from Asian bigheaded carp (hypophthalmichthys spp.). PLoS ONE. 9(12).
275. Turner, I. L.; Harley, M. D.; Drummond, C. D. 2016. UAVs for coastal surveying. Coastal Engineering. 114: 19-24.
276. Unsworth, R. K. F.; Peters, J. R.; McCloskey, R. M.; Hinder, S. L. 2014. Optimising stereo baited underwater video for sampling fish and invertebrates in temperate coastal habitats. Estuarine, Coastal and Shelf Science. 150(PB): 281-287.
277. Waddington, K. I.; Piek, B. W.; Payne, A. D.; Grove, S. L.; Harvey, E. S.; Kendrick, G. A.; Taylor, H. F.; Meeuwig, J. J. 2010. Description of a remote still photography system for collection of benthic photo-quadrats. Marine Technology Society Journal. 44(2): 56-63.
278. Valta-Hulkkonen, K.; Kanninen, A.; Ilvonen, R.; Leka, J. 2005. Assessment of aerial photography as a method for monitoring aquatic vegetation in lakes of varying trophic status. Boreal Environment Research. 10(1): 57-66.
279. Van Lancker, V.; Baeye, M. 2015. Wave glider monitoring of sediment transport and dredge plumes in a shallow marine sandbank environment. PLoS ONE. 10(6).
280. van Overmeeren, R.; Craeymeersch, J.; van Dalfsen, J.; Fey, F.; van Heteren, S.; Meesters, E. 2009. Acoustic habitat and shellfish mapping and monitoring in shallow coastal water - Sidescan sonar experiences in The Netherlands. Estuarine, Coastal and Shelf Science. 85(3): 437-448.
281. van Rein, H.; Schoeman, D. S.; Brown, C. J.; Quinn, R.; Breen, J. 2011. Development of benthic monitoring methods using photoquadrats and scuba on heterogeneous hard-substrata: A boulder-slope community case study. Aquatic Conservation: Marine and Freshwater Ecosystems. 21(7): 676-689.
282. Van Rein, H.; Schoeman, D. S.; Brown, C. J.; Quinn, R.; Breen, J. 2012. Development of low-cost image mosaics of hard-bottom sessile communities using SCUBA: Comparisons of optical media and of proxy measures of community structure. Journal of the Marine Biological Association of the United Kingdom. 92(1): 49-62.
283. Varkitzi, I.; Francé, J.; Basset, A.; Cozzoli, F.; Stanca, E.; Zervoudaki, S.; Giannakourou, A.; Assimakopoulou, G.; Venetsanopoulou, A.; Mozeti?, P.; Tinta, T.; Skejic, S.; Vidjak, O.; Cadiou, J. F.; Pagou, K. 2018. Pelagic habitats in the Mediterranean Sea: A review of Good Environmental Status (GES) determination for plankton components and identification of gaps and priority needs to improve coherence for the MSFD implementation. Ecological Indicators. 95: 203-218.
284. Warren, J. K.; Vlahos, P.; Smith, R.; Tobias, C. 2018. Investigation of a new passive sampler for the detection of munitions compounds in marine and freshwater systems. Environmental Toxicology and Chemistry. 37(7): 1990-1997.
285. Warren-Myers, F.; Ingram, B. A.; Dempster, T.; Swearer, S. E. 2018. Enriched stable isotope marking of hatchery trout via immersion: A method to monitor restocking success. Fisheries Research. 197: 78-83.
286. Waseem, M. H.; Alamzeb, M.; Mustafa, B.; Malik, F.; Shakir, M.; Khan, M. A. 2013. Design of a low-cost underwater wireless sensor network for water quality monitoring. IETE Journal of Research. 59(5): 523-534.
287. Vasimalai, N.; Fernández-Argüelles, M. T.; Espiña, B. 2018. Detection of Sulfide Using Mercapto Tetrazine-Protected Fluorescent Gold Nanodots: Preparation of Paper-Based Testing Kit for On-Site Monitoring. ACS Applied Materials and Interfaces. 10(2): 1634-1645.
288. Watson, J. L.; Huntington, B. E. 2016. Assessing the performance of a cost-effective video lander for estimating relative abundance and diversity of nearshore fish assemblages. Journal of Experimental Marine Biology and Ecology. 483: 104-111.
289. Watson, S. B.; Zastepa, A.; Boyer, G. L.; Matthews, E. 2017. Algal bloom response and risk management: On-site response tools. Toxicon. 129: 144-152.
290. Ventura, D.; Bonifazi, A.; Gravina, M. F.; Belluscio, A.; Ardizzone, G. 2018. Mapping and classification of ecologically sensitive marine habitats using unmanned aerial vehicle (UAV) imagery and Object-Based Image Analysis (OBIA). Remote Sensing. 10(9).
291. Ventura, D.; Bruno, M.; Jona Lasinio, G.; Belluscio, A.; Ardizzone, G. 2016. A low-cost drone based application for identifying and mapping of coastal fish nursery grounds. Estuarine, Coastal and Shelf Science. 171: 85-98.
292. Vianna, G. M. S.; Meekan, M. G.; Bornovski, T. H.; Meeuwig, J. J. 2014. Acoustic telemetry validates a citizen science approach for monitoring sharks on coral reefs. PLoS ONE. 9(4).
293. Wight, N. A.; Suzuki, J.; Vadopalas, B.; Friedman, C. S. 2009. DEVELOPMENT AND OPTIMIZATION OF QUANTITATIVE PCR ASSAYS TO AID OSTREA LURIDA CARPENTER 1864 RESTORATION EFFORTS. Journal of Shellfish Research. 28(1): 33-41.
294. Williams, J. L.; Pierce, S. J.; Fuentes, M. M. P. B.; Hamann, M. 2015. Effectiveness of recreational divers for monitoring sea turtle populations. Endangered Species Research. 26(3): 209-219.
295. Williams, R.; Thomas, L. 2009. Cost-effective abundance estimation of rare animals: Testing performance of small-boat surveys for killer whales in British Columbia. Biological Conservation. 142(7): 1542-1547.
296. Vilmi, A.; Karjalainen, S. M.; Nokela, T.; Tolonen, K.; Heino, J. 2016. Unravelling the drivers of aquatic communities using disparate organismal groups and different taxonomic levels. Ecological Indicators. 60: 108-118.
297. Wittwer, C.; Stoll, S.; Strand, D.; Vrålstad, T.; Nowak, C.; Thines, M. 2018. eDNA-based crayfish plague monitoring is superior to conventional trap-based assessments in year-round detection probability. Hydrobiologia. 807(1): 87-97.
298. Wong, K.; Fong, T. T.; Bibby, K.; Molina, M. 2012. Application of enteric viruses for fecal pollution source tracking in environmental waters. Environment International. 45(1): 151-164.
299. Wood, G.; Lynch, T. P.; Devine, C.; Keller, K.; Figueira, W. 2016. High-resolution photo-mosaic time-series imagery for monitoring human use of an artificial reef. Ecology and Evolution. 6(19): 6963-6968.
300. Xu, G.; Wang, Z.; Yang, Z.; Xu, H. 2015. Congruency analysis of biofilm-dwelling ciliates as a surrogate of eukaryotic microperiphyton for marine bioassessment. Marine Pollution Bulletin. 101(2): 600-604.
301. Xu, K.; Du, Y.; Lei, Y.; Dai, R. 2010. A practical method of Ludox density gradient centrifugation combined with protargol staining for extracting and estimating ciliates in marine sediments. European Journal of Protistology. 46(4): 263-270.
302. Xu, M.; Cao, H.; Xie, P.; Deng, D.; Feng, W.; Xu, J. 2005. Use of PFU protozoan community structural and functional characteristics in assessment of water quality in a large, highly polluted freshwater lake in China. Journal of Environmental Monitoring. 7(7): 670-674.
303. Xu, T.; Close, D.; Smartt, A.; Ripp, S.; Sayler, G. 2014. Detection of organic compounds with whole-cell bioluminescent bioassays. Advances in Biochemical Engineering/Biotechnology. 144: 111-151.
304. Yamanaka, H.; Minamoto, T. 2016. The use of environmental DNA of fishes as an efficient method of determining habitat connectivity. Ecological Indicators. 62: 147-153.
305. Yamanaka, H.; Takao, D.; Maruyama, A.; Imamura, A. 2018. Species-specific detection of the endangered piscivorous cyprinid fish Opsariichthys uncirostris uncirostris, three-lips, using environmental DNA analysis. Ecological Research. 33(5): 1075-1078.
306. Yamano, H.; Shimazaki, H.; Matsunaga, T.; Ishoda, A.; McClennen, C.; Yokoki, H.; Fujita, K.; Osawa, Y.; Kayanne, H. 2006. Evaluation of various satellite sensors for waterline extraction in a coral reef environment: Majuro Atoll, Marshall Islands. Geomorphology. 82(43528): 398-411.
307. Yang, C. H.; You, J. I.; Lin, C. P. 2002. Delineating lake bottom structure by resistivity image profiling on water surface. Terrestrial, Atmospheric and Oceanic Sciences. 13(1): 39-52.
308. Yin, G.; Danielsson, S.; Dahlberg, A. K.; Zhou, Y.; Qiu, Y.; Nyberg, E.; Bignert, A. 2017. Sampling designs for contaminant temporal trend analyses using sedentary species exemplified by the snails Bellamya aeruginosa and Viviparus viviparus. Chemosphere. 185: 431-438.
309. Yoon, T. H.; Kang, H. E.; Kang, C. K.; Lee, S. H.; Ahn, D. H.; Park, H.; Kim, H. W. 2016. Development of a cost-effective metabarcoding strategy for analysis of the marine phytoplankton community. PeerJ. 2016(6).
310. Zappalà, G.; Marcelli, M.; Piermattei, V. 2008. Development of a sliding device for extended measurements in coastal waters. WIT Transactions on Ecology and the Environment. 111: 187-195.
311. Zeh, D. R.; Heupel, M. R.; Limpus, C. J.; Hamann, M.; Fuentes, M. M. P. B.; Babcock, R. C.; Pillans, R. D.; Townsend, K. A.; Marsh, H. 2015. Is acoustic tracking appropriate for air-breathing marine animals? Dugongs as a case study. Journal of Experimental Marine Biology and Ecology. 464: 43475.
312. Zhang, J.; Hanner, R. 2012. Molecular approach to the identification of fish in the South China Sea. PLoS ONE. 7(2).
313. Zhou, C. Y.; Guan, D. X.; Williams, P. N.; Luo, J.; Ma, L. Q. 2016. Novel DGT method with tri-metal oxide adsorbent for in situ spatiotemporal flux measurement of fluoride in waters and sediments. Water Research. 99: 200-208.
